# Large-scale Identification of N-linked Intact Glycopeptides in Human Serum using HILIC Enrichment and Spectral Library Search*[Fn FN2]

**DOI:** 10.1074/mcp.RA119.001791

**Published:** 2020-02-26

**Authors:** Qingbo Shu, Mengjie Li, Lian Shu, Zhiwu An, Jifeng Wang, Hao Lv, Ming Yang, Tanxi Cai, Tony Hu, Yan Fu, Fuquan Yang

**Affiliations:** ‡ Laboratory of Protein and Peptide Pharmaceuticals & Proteomics Laboratory, Institute of Biophysics, Chinese Academy of Sciences, Beijing 100101, China; § National Center for Mathematics and Interdisciplinary Sciences, Key Laboratory of Random Complex Structures and Data Science, Academy of Mathematics and Systems Science, Chinese Academy of Sciences, Beijing 100190, China; ¶ Center for Cellular and Molecular Diagnostics, Department of Biochemistry and Molecular Biology, School of Medicine, Tulane University, New Orleans, Louisiana 70112; ‖ Computer Network Information Center, Chinese Academy of Sciences, Beijing 100101, China; ** University of Chinese Academy of Sciences, Beijing 100049, China; ‡‡ Research Center for Basic Sciences of Medicine, Basic Medical College, Guizhou Medical University, Guiyang 550025, China

**Keywords:** Glycomics, glycoproteomics, bioinformatics software, mass spectrometry, glycoprotein pathways, glycoproteins, co-elution, human serum, isotopic distribution, N-linked intact glycopeptide, spectral library search

## Abstract

Large-scale identification of *N*-linked intact glycopeptides by liquid chromatography coupled with tandem mass spectrometry (LC-MS/MS) in human serum is challenging because of the wide dynamic range of serum protein abundances, the lack of a complete serum N-glycan database and the existence of proteoforms. In this regard, a spectral library search method was presented for the identification of *N*-linked intact glycopeptides from *N*-linked glycoproteins in human serum with target-decoy and motif-specific false discovery rate (FDR) control. Serum proteins were firstly separated into low-abundance and high-abundance proteins by acetonitrile (ACN) precipitation. After digestion, the *N*-linked intact glycopeptides were enriched by hydrophilic interaction liquid chromatography (HILIC) and a portion of the enriched *N*-linked intact glycopeptides were processed by Peptide-N-Glycosidase F (PNGase F) to generate *N*-linked deglycopeptides. Both *N*-linked intact glycopeptides and deglycopeptides were analyzed by LC-MS/MS. From *N*-linked deglycopeptides data sets, 764 *N*-linked glycoproteins, 1699 *N*-linked glycosites and 3328 unique *N*-linked deglycopeptides were identified. Four types of *N*-linked glycosylation motifs (NXS/T/C/V, X≠P) were used to recognize the *N*-linked deglycopeptides. The spectra of these *N*-linked deglycopeptides were utilized for *N*-linked deglycopeptides library construction and identification of *N*-linked intact glycopeptides. A database containing 739 N-glycan masses was constructed and utilized during spectral library search for the identification of *N*-linked intact glycopeptides. In total, 526 *N*-linked glycoproteins, 1036 *N*-linked glycosites, 22,677 *N*-linked intact glycopeptides and 738 N-glycan masses were identified under 1% FDR, representing the most in-depth serum N-glycoproteome identified by LC-MS/MS at *N*-linked intact glycopeptide level.

## Abstract (precis)

The human serum N-linked glycoproteome has been determined through LC-MS/MS. The intact glycopeptides were identified through a spectral library search method embedded in the pMatchGlyco software. Four types of known N-glycosylation motifs, prevalent variable modifications and semi-tryptic digestion were considered during searching and the identified intact glycopeptides were validated through target-decoy and motif-specific false discovery rate (FDR) control. The results reveal site-specific glycosylation of serum glycoproteins and provide high-quality tandem mass spectra of 22,677 serum N-linked intact glycopeptides.

## Graphical Abstract

**Figure:**
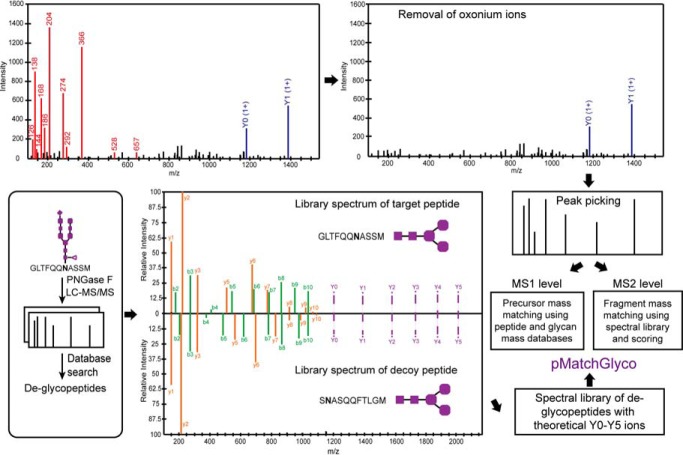

**Highlights**

- This study proposed a spectral library search method to accurately identify N-linked glycopeptides in human serum through LC-MS/MS with pMatchGlyco software.
- The identification depth of serum N-linked intact glycopeptides and glycoproteins was increased by combination of acetonitrile precipitation, HILIC enrichment and high-pH RPLC fractionation.
- 22,677 unique serum N-linked intact glycopeptides corresponding to 526 N-linked glycoproteins were identified with N-glycosylation motif-specific FDR control.
- This study revealed the great microheterogeneity of N-linked glycoproteins in serum.

N-linked glycoproteins in human serum have been utilized in diagnosing of diseases for decades, such as prostate specific antigen (PSA)^1^ and cancer antigen 125 (CA-125) (1). Accurate identification of *N*-linked intact glycopeptides is thus a prerequisite to monitor their changes under different disease status. LC-MS/MS has been widely applied to identifying and quantifying *N*-linked glycopeptides. In this field, data-dependent acquisition (DDA) is usually utilized. In DDA mode, precursor ions are selected for MS/MS acquisition according to their relative intensities on MS1. To isolate a precursor ion, its *m*/*z* value serves as the center point of an isolation window, and all ions in this window are fragmented at the same time. Hence, a list of precursor *m*/*z* recorded by the instrument will include the most intense isotopic peak of this precursor ion in its elution profile. A lot of software tools are developed to extract MS/MS spectra of different formats of raw data produced by different mass spectrometers. They inspect the isolation windows corresponding to each MS/MS spectrum and export the most possible precursor, such as MSConvert (2), and infer precursor *m*/*z* simply by exporting the *m*/*z* value recorded on each MS/MS spectrum. If the monoisotopic peak is selected for fragmentation during DDA and recorded as the precursor m/z on MS/MS spectrum, this streamline strategy can find the monoisotopic peak easily. However, the monoisotopic peak of *N*-linked intact glycopeptide is rarely the most intense peak among its isotope cluster, which is less likely selected as precursor *m*/*z* in DDA compared to its other isotopic peaks. Therefore, a software tool that can precisely recognize the monoisotopic peaks of *N*-linked intact glycopeptides is usually needed in the glycoproteomic study. In addition, conjugation with glycan increases hydrophilicity of peptides. They are more difficult to be separated by commonly used reversed-phase liquid chromatography (RPLC) than non-glycosylated peptides. Hence, co-elution of *N*-linked intact glycopeptides in LC and co-fragmentation of their precursor ions in MS are not uncommon in LC-MS analysis of glycopeptides. This could distort isotopic distribution of each precursor ion and make it difficult to determine its respective monoisotopic peak or precursor m/z. Software tools that enable accurate determination of precursor *m*/*z* of *N*-linked intact glycopeptides in MS1 spectra are still under developing. To improve the accuracy of precursor *m*/*z*, a correction step called precursor ion selection was employed in a recent study after data conversion from vendor-specific binary files to open-format files (3). In that study, a software tool called GPQuest with spectral library search strategy was utilized in *N*-linked intact glycopeptide identification from human cell lysates. Instead of post-correction of precursor *m*/*z*, another tool pParse aims to find the precursor *m*/*z* precisely and further separate co-eluted peptides based on machine learning (4). It was embedded into pGlyco 2.0 and was successfully applied to mouse tissue *N*-linked intact glycopeptide identification through database search (5). Both studies achieved in-depth identification of *N*-linked intact glycopeptides in biological samples, though they were different in their identification strategies. Different from the proteomes of cell and tissue lysates, human serum proteome has a wider dynamic range in protein abundance. Hence, it is a big challenge to deeply identify the *N*-linked intact glycopeptides in human serum. To date, the largest spectral library of plasma N-glycopeptides contains 4347 *N*-linked deglycopeptides corresponding to 1151 plasma glycoproteins (6). This was achieved by sophisticate fractionation of plasma proteins and hydrazide chemistry enrichment of *N*-linked intact glycopeptides, followed by hydrolyzing of N-glycans using PNGase F to generate *N*-linked deglycopeptides and LC-MS/MS identification. Because of the covalent binding of glycan to hydrazide beads in hydrazide chemistry enrichment, this enrichment strategy is more suitable to large-scale glycosite mapping instead of *N*-linked intact glycopeptide identification. Notably, there are some false positive *N*-linked glycosites caused by spontaneous deamidation of asparagine. To decrease the false positive identification of glycosites, a two-step enzymatic cleavage strategy was recently developed to specifically identify *N*-linked glycosites and deglycopeptides, in which carboxyl groups of aspartic acid (D) were modified with aniline before *N*-linked glycans were released by PNGase F digestion, and the generated *N*-linked deglycopeptides contained new aspartic residues which were able to be cleaved by Asp-N (3). Together with the software GPQuest, this two-step cleavage strategy was applied to human serum and 331 *N*-linked glycoproteins were identified (7). By ignoring the difference between plasma and serum proteome, the coverage of serum N-glycoproteome achieved by this two-step cleavage strategy was 28.8% (331/1151).

In this study, we proposed a complete workflow to increase the depth of serum N-glycoproteome through LC-MS/MS. First, serum proteins were separated into low-abundance and high-abundance ones by ACN precipitation. After tryptic digestion, HILIC was used to enrich glycopeptides, considering its high specificity in glycopeptide enrichment (7–9). Second, PNGase F was used to produce *N*-linked deglycopeptides from a portion of the enriched *N*-linked intact glycopeptide samples. The *N*-linked deglycopeptides were further fractionated through high-pH RPLC, analyzed using LC-MS/MS and identified through protein sequence database search using pFind 2.8.8 (10). From *N*-linked deglycopeptides data sets, 764 *N*-linked glycoproteins, 1699 *N*-linked glycosites and 3328 unique *N*-linked deglycopeptides were identified. The coverage of serum N-glycoproteome identified by this strategy was increased to 66.4% (764/1151). A spectral library of identified deglycopeptides with theoretical Y0–Y5 ions was generated by pMatchGlyco (version 1.2) (11). *N*-linked intact glycopeptides were also analyzed using LC-MS/MS. To reduce co-elution of *N*-linked intact glycopeptides on LC column, high-pH RPLC was used for *N*-linked intact glycopeptide fractionation. Furthermore, pParse's co-elution function was activated to accurately determine the precursor *m*/*z* of *N*-linked intact glycopeptides. Through open spectral library search and target-decoy FDR control, 526 *N*-linked glycoproteins were identified in serum using pMatchGlyco. Furthermore, 1036 *N*-linked glycosites, 22,677 *N*-linked intact glycopeptides and 738 N-glycan masses were identified under 1% FDR, representing the most in-depth serum N-glycoproteome identified by LC-MS/MS at both intact *N*-linked glycopeptide level and *N*-linked glycoprotein level.

## EXPERIMENTAL PROCEDURES

### 

#### 

##### Experimental Design and Statistical Rationale

**Fig. 1.**
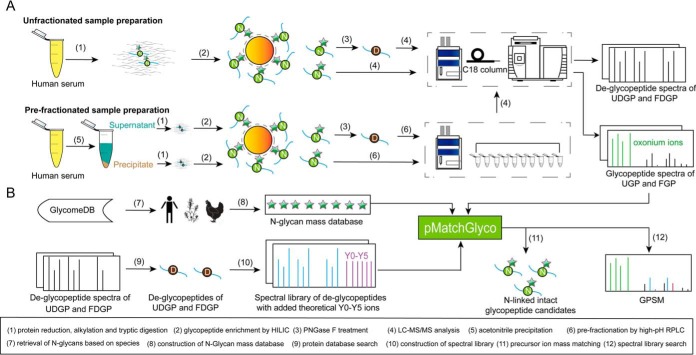
**Experimental design of *N*-linked intact glycopeptide identification in human serum.** *A*, Serum is processed with or without ACN precipitation and high pH RPLC fractionation. N-glycan is presented as dark green star and peptide backbone is presented as line. *B*, Data analysis workflow for N-linked intact glycopeptide identification.

In this study, one serum specimen from single healthy donor and one pooled serum specimen from 50 healthy donors were used. A detailed scheme of experimental design for N-glycoproteomics of human serum is shown in Fig. 1.

For the single donor serum specimen, after tryptic digestion and HILIC enrichment, one portion of enriched *N*-linked intact glycopeptide sample was analyzed by LC-MS/MS for four times and generated *N*-linked intact glycopeptides (unfractionated *N*-linked intact glycopeptides, UGP). Another portion of enriched *N*-linked intact glycopeptide sample was treated with PNGase F and then analyzed by LC-MS/MS for four times and generated *N*-linked deglycopeptides (unfractionated *N*-linked deglycopeptides, UDGP).

For pooled serum specimen, proteins were first separated into low and high abundant protein fractions in the supernatant and the precipitate by ACN precipitation. Proteins in the supernatant and the precipitate were processed with tryptic digestion and HILIC enrichment respectively to generate two *N*-linked intact glycopeptide samples. One portion of each enriched *N*-linked intact glycopeptides (40 μg) was fractionated into 10 fractions by high-pH RPLC, and then analyzed by LC-MS/MS, respectively. Another portion of each enriched *N*-linked intact glycopeptide sample (40 μg) was treated with PNGase F, fractionated into 10 fractions by high-pH RPLC, and then analyzed by LC-MS/MS respectively. The LC-MS/MS analyses finally generated two data sets, fractionated *N*-linked intact glycopeptides (FGP) and fractionated *N*-linked deglycopeptides (FDGP). FGP consisted with *N*-linked intact glycopeptides from supernatant (SGP) and *N*-linked intact glycopeptides from precipitate (PGP). FDGP consisted with *N*-linked deglycopeptides from supernatant (SDGP) and deglycopeptides from precipitate (PDGP).

The rationale for the LC-MS/MS analysis with four technical replicates for the single donor specimen is that the overlap of *N*-linked deglycopeptides identified between two single LC-MS/MS runs is 80% and the number of *N*-linked deglycopeptides identified will be saturated after four technical replicates (12). On the other side, it allows us to evaluate the MS instrumental variation by comparing retention times and peak areas of identified *N*-linked intact glycopeptides among four LC-MS/MS runs. It is important because a quantitative and high-throughput serum glycoproteome assay relies on a robust LC-MS/MS setting by knowing instrumental variation. There are two reasons for pooling sera from 50 healthy donors. First, the biological variance will be reduced by pooling sera from 50 different subjects. As a proof, a previous study used 12–45 subjects to construct 12 standard serum sets for evaluating biomarkers for ovarian, breast, or endometrial cancer (13). Four glycoprotein biomarkers, *i.e.* CA 125, CA 19-9, CA 15-3, and CEA were evaluated in that study. Repeated measurement of these markers among 20 aliquots of standard serum sets showed no more than 2.6% coefficients of variation (CV) of the four glycoproteins, which is acceptable in clinical diagnosis. Therefore, a pooled sera specimen from 50 healthy donors was employed in this work to represent the potential biological variations of *N*-linked glycoproteome. Second, it allows repeatable pre-fractionation of serum specimen to 40 fractions using our current workflow and analyzing by LC-MS/MS in a reasonable instrumental time. Because both specimens are from healthy donors and undergo distinct sample preparation workflows, *i.e.* un-fractionation *versus* pre-fractionation. This study compared the number of *N*-linked glycoproteins, glycosites, deglycopeptides, intact glycopeptides, and glycans identified in the un-fractionated and pre-fractionated specimens and showed the powerful role of pre-fractionation in increasing the depth of serum *N*-linked glycoproteome. Further evaluation of biological variation of *N*-linked glycoproteome in serum is critical in translating this method into clinical usage. However, it requires quantitative experimental design and customized MS data analysis software, which is not in the scope of this study.

##### Sample Collection

This research project was reviewed and approved by the Institutional Review Board (IRB) at Institute of Biophysics, Chinese Academy of Sciences before study initiation. Serum samples were obtained from healthy donors enrolled in IRB-approved protocols at the clinical laboratory, No.306 hospital of the Chinese People's Liberation Army. All donors were fasted for 12 h before blood collection. Sera were collected invacutainer blood collection tubes with blood coagulation accelerator and inertia separating gel after centrifugation at 2000 × *g* for 8 min. Two aliquots of 20 μl serum sample from single healthy donor were used for glycopeptide enrichment and identification without pre-fractionation. 20 equal aliquots of 200 μl pooled sera sample from 50 healthy subjects were used for pre-fractionated sample preparation workflow.

##### Glycoprotein Standards and Related Sample Preparation

Horseradish peroxidase (HRP, Catalogue #31490) from *Armoracia rusticana* was purchased from Thermo Scientific. Ovalbumin (Catalogue #A5503) and human holo-transferrin (Catalogue #T4132) were purchased from Sigma-Aldrich. Two milligrams HRP, transferrin, and ovalbumin were processed using the procedure used above to generate their respective *N*-linked deglycopeptides and *N*-linked intact glycopeptides. Additionally, 6 μg HRP intact glycopeptides and 6 μg ovalbumin intact glycopeptides were mixed with 6 μg human serum intact glycopeptides. The spiked sample was equally divided into two portions, one portion was treated with PNGase F to generate the *N*-linked deglycopeptides followed by LC-MS/MS analysis with four replicates, and another portion was analyzed by LC-MS/MS with four replicates too.

##### Serum Protein Precipitation

In the pre-fractionated sample preparation workflow, 20 equal aliquots of 200 μl pooled serum samples (protein concentration: 68 μg/μL) then added 1200 μl ACN and mixed at 4 °C for 30 min. Each sample solution was then centrifuged at 16,000 × *g* at 4 °C for 30 min. The supernatant and precipitate were collected separately and dried by refrigerated centrifugal vacuum concentrator (LABCONCO, Kansas City, MO).

##### Protein Reduction, Alkylation and Tryptic Digestion

The serum protein and commercial glycoprotein samples were denatured in 8 m urea/0.1 M Tris buffer (pH 8.5, adjusted by adding HCl) respectively, then reduced by 10 mm dithiothreitol (DTT) at 37 °C for 1 h and alkylated by 40 mm iodoacetamide at room temperature in the dark for 40 min. The excess iodoacetamide was reduced by 10 mm DTT at 37 °C for 30 min. The sample solution was diluted with 50 mm NH_4_HCO_3_to a final concentration of urea less than 2 m, the sequencing grade trypsin (Promega, Madison, WI; protein: enzyme, 50:1, w/w) was then added in and incubated at 37 °C overnight with shaking on Thermo mixer (Thermo Fisher Scientific, Waltham, MA). Digestion was terminated by adding formic acid (FA) to a final concentration of 0.1%. The tryptic peptide sample was then desalted using Oasis HLB cartridge (Waters, MA) with 0.1% TFA. The peptides were eluted with 1 mL20% ACN/0.1% TFA and 1 mL40% ACN/0.1% TFA. The eluted peptide solutions were combined and concentrated to 100 μl by refrigerated centrifugal vacuum concentrator. Peptide concentration was measured by spectrophotometer of nanodrop 2000 (Thermo Fisher Scientific).

##### N-linked Intact Glycopeptides Enrichment by HILIC

Serum N-linked intact glycopeptides were enriched by HILIC using a previously reported method (14). The enrichment of *N*-linked intact glycopeptides was performed on a RIGOL l-3000 Series HPLC system coupling with an Ultimate HILIC amphion column (4.6 × 100 mm, 5 μm, 120 Å, Welch Materials, Inc., Shanghai, China), a manual injection valve and a UV detector. The enrichment of *N*-linked intact glycopeptides from all kinds of samples in this study was performed using the same method as following.

One aliquot of tryptic peptide solution, which contains about 400 μg lyophilized tryptic peptides was taken, and dried by refrigerated centrifugal vacuum concentrator, then re-dissolved in 20 μl 0.1% TFA followed by adding 80 μl ACN/0.1% TFA slowly for further HILIC separation. The amphion HILIC column was first equilibrated with 80% ACN/0.1% TFA aqueous solution for 20 min. About 100 μl peptide solution was injected onto the HILIC column through a manual injection valve, and peptides were eluted with buffer A (0.1% TFA in deionized water) and buffer B (0.1% TFA in ACN) at a 38 min gradient: 80% buffer B (0.1 min)-80% buffer B (20 min)-2% buffer B (21 min)-2% buffer B (30 min)-80% buffer B (31 min)-80% buffer B (38 min), and at a flow-rate of 0.7 ml/min. The eluate was detected at 214 nm. The *N*-linked intact glycopeptides containing fractions were collected from 23 to 26 min according to the chromatogram, and then dried by refrigerated centrifugal vacuum concentrator. After several times of enrichment by HILIC, the collected fractions of glycopeptide were combined to be one sample for further enrichment to improve the purity of glycopeptide. After that, one portion of *N*-linked intact glycopeptide sample was re-dissolved in 0.1% FA for further LC-MS/MS analysis. Another portion was treated with PNGase F to generate deglycopeptides.

##### PNGase F Treatment

For one aliquot of the enriched *N*-linked intact glycopeptides (4 μg) from unfractionated serum proteins, 1 μl of PNGase F (P0705S, 500,000 units/ml, NEB, MA) was added into 10 μl of *N*-linked intact glycopeptide solution in 50 mm NH_4_HCO_3_ and incubated 4 h at 37 °C to release N-glycan. After PNGase F treatment, the deglycopeptide samples were desalted by ZipTip C18 (ZTC18S960, Merck KGaA, Darmstadt, Germany) with 0.1%TFA and eluted successively with 10 μl 20% ACN/0.1%TFA, 10 μl 40% ACN/0.1%TFA, 10 μl 60% ACN/0.1%TFA. The eluted fractions were combined and dried by refrigerated centrifugal vacuum concentrator and re-dissolved in 20 μl 0.1% FA for LC-MS/MS analysis.

For one aliquot of enriched *N*-linked intact glycopeptides (40 μg) from fractionated sera proteins, 10 μl of PNGase F was added into 40 μl of *N*-linked intact glycopeptide solution in 50 mm NH_4_HCO_3_ and incubated 4 h at 37 °C to release N-glycan. After PNGase F treatment, the deglycopeptide samples were desalted by C18 Tips (87784, Pierce, Rockford) with 0.1%TFA and eluted successively with 100 μl 20% ACN/0.1%TFA, 100 μl 40% ACN/0.1%TFA, 100 μl 60% ACN/0.1%TFA. The eluted fractions were combined and dried by refrigerated centrifugal vacuum concentrator and re-dissolved in 20 μl 0.1% FA for LC-MS/MS analysis.

##### Fractionation of Peptides Using High-pH RPLC

The fractionation of enriched *N*-linked intact glycopeptide and deglycopeptide samples were performed on a RIGOL l-3000 Series HPLC system coupling with a Xbridge^®^ Peptide BEH C18 column (2.1 mm × 150 mm, 3.5 μm, 130 Å), a manual injection valve and a UV detector. The fractionation of *N*-linked intact glycopeptides and deglycopeptides in this study were performed using the same method as following.

The enriched *N*-linked intact glycopeptides (40 μg) or deglycopeptides (40 μg) from supernatant and precipitate were re-dissolved in 100 μl 0.1% ammonium hydroxide solution (pH 10), respectively, then injected into a Xbridge Peptide BEH C18 column (2.1 mm×150 mm, 3.5 μm, 130 Å), fractionated with buffer A (2%ACN/98%H_2_O/0.1%NH_3_·H_2_O) and buffer B (98%ACN/2%H_2_O/0.1%NH_3_·H_2_O) at a 76 min gradient: 5% buffer B (0 min)-8% buffer B (5 min)-18% buffer B (40 min)-32% buffer B (62 min)-95% buffer B(64 min)-95% buffer B(68 min)-5% buffer B (69min)-5% buffer B (76 min), and at a flowrate of 0.23 ml/min. The eluate was detected at 214 nm. 93 fractions of *N*-linked intact glycopeptides or deglycopeptides were collected in a time-based method start from 5 to 66 min and then concatenated into 12 fractions by combining fractions 1,13, 25, 37, 49, 61, 73; 2, 14, 26, 38, 50, 63, 75, etc. The fractions were then dried by refrigerated centrifugal vacuum concentrator and stored at −80 °C until use.

##### LC-MS/MS Analysis

LC-MS/MS analysis of deglycopeptides from human sera and standard glycoproteins HRP, ovalbumin, transferrin, and serum spiked with HRP and ovalbumin were performed on an EASY-nLC 1000 HPLC coupled with Q Exactive mass spectrometer (Thermo Fisher Scientific, Bremen, Germany). Deglycopeptides were re-dissolved in 0.1% FA and injected into a reversed-phase C18 trap column (100 μm × 2 cm, 5 μm, Reprosil-Pur C18 AQ, Dr. Maisch GmbH, Germany) and then separated on a self-packed reversed-phase C18 analytical column (75 μm × 20 cm, 3 μm, Reprosil-Pur C18 AQ) with mobile phase A (0.1% FA) and mobile phase B (ACN/0.1% FA) at a 78 min gradient: 4% buffer B (0 min)-8% buffer B (5 min)-22% buffer B (58 min)-32% buffer B (70 min)-90% buffer B (71 min)- 90% buffer B (78 min), and at a flowrate of 310 nL/min.

The mass spectrometer was operated in DDA mode. Full scan MS spectra (mass range from *m*/*z* 300 to 1600) were acquired in the Orbitrap with resolution of 70,000 at *m*/*z* 200. The automatic gain control (AGC) was set as 3 × 10^6^ and the maximum injection time (IT) was set as 60 ms. The top 20 precursor ions were selected from each MS full scan with isolation width of *m*/*z* 2. They were fragmented in the HCD collisional cell with normalized collision energy (NCE) of 27%. Subsequently, MS/MS spectra were acquired in the Orbitrap with resolution of 17,500 at *m*/*z* 200. The AGC was 5 × 10^4^ and the maximum injection time was 80 ms. Ions selected for MS/MS were dynamically excluded for a duration of 40s. The charges of precursor ions were set as 2–5, and the default charge was 2, peptide match was switched on. The spray voltage was 2.0 kV and the heated capillary temperature was 320 °C.

LC-MS/MS analyses of UGP were performed using the method mentioned above with modification of the mass range to *m*/*z* 400–2400.

For the *N*-linked intact glycopeptides derived from pre-fractionated human sera, commercial HRP, ovalbumin and transferrin, the sera sample spiked with HRP and ovalbumin, LC-MS/MS analyses were performed using the method mentioned above with modification of the mass range to *m*/*z* 700–2000 and NCE to 33%. The charges of precursor ions were set as 2–6 and the default charge was 3, and peptide match was switched off.

##### Identification of N-linked Deglycopeptides

All LC-MS/MS spectra of *N*-linked deglycopeptides in UDGP, FDGP, commercial HRP, ovalbumin and transferrin as well as the sera spiked with HRP and ovalbumin were converted to .mgf files using pParse2.0 with the *co-elute* parameter set as “0”. After conversion, UDGP and FDGP spectra were searched against the Uniprot *Homo sapiens* protein database (downloaded from Uniprot website on May 20, 2018, consisting of 73,112 entries) by pFind (v2.8.8). The search parameters were set as follows: up to 2 missed cleavages, 10 ppm precursor mass tolerance and 0.02 Da fragment mass tolerance; carbamidomethylation (C) was a fixed modification. Semi-tryptic digestion and 16 variable modifications were considered. To reduce the search time caused by the large number of variable modifications, the spectra were searched 7 times separately by setting the enzyme digestion and variable modifications as follows: (1) semi-tryptic digestion with deamidated (N), oxidation (M) and Gln->pyro-Glu (Any N-term Q); (2) tryptic digestion with deamidated (N), oxidation (M) and Gln->pyro-Glu (Any N-term Q); (3) tryptic digestion with deamidated (N,Q), oxidation (M), Gln->pyro-Glu (Any N-term Q) and amidine (Any N-term); (4) tryptic digestion with deamidated (N), oxidation (M,H,W), Gln->pyro-Glu (Any N-term Q) and dioxidation (C, M, W); (5) tryptic digestion with deamidated (N), oxidation (M), Gln->pyro-Glu (Any N-term Q) and carbamidomethyl (Any N-term, K, H); (6) tryptic digestion with deamidated (N), oxidation (M), Gln->pyro-Glu (Any N-term Q) andcarbamyl (K); (7) tryptic digestion with deamidated (N), oxidation (M), Gln->pyro-Glu (Any N-term Q), formyl (Any N-term) and sulfide (C).

For identification of *N*-linked deglycopeptides from commercial ovalbumin, the deglycopeptide spectra were searched against the protein database of *Gallus gallus* available in Uniprot (downloaded from Uniprot website on July 19, 2019, consisting of 27,804 entries) with semi-tryptic digestion. Gln->pyro-Glu (Any N-term Q), oxidation (M, H, W), deamidated (N), deamidated (Q), amidine (Any N-term), carbamidomethyl (Any N-term), carbamidomethyl (H), carbamidomethyl (K), carbamyl (K), dioxidation (C, M, W), formyl (Any N-term), and sulfide (C) were set as variable modifications. Note that we specified these modifications in one search instead of dividing them into groups because the size of data was relatively small.

For identification of *N*-linked deglycopeptides from commercial HRP, the deglycopeptide spectra were searched against the protein database of *Armoracia rusticana* available in Uniprot (8 entries) with semi-tryptic digestion. Gln->pyro-Glu (Any N-term Q), oxidation (M, H, W), deamidated (N), deamidated (Q), amidine (Any N-term), carbamidomethyl (Any N-term, H, K), carbamyl (K), dioxidation (C, M, W), formyl (Any N-term), and sulfide (C) were set as variable modifications.

For identification of *N*-linked deglycopeptides of transferrin, the deglycopeptide spectra were searched against the protein database of *Homo sapiens* available in Uniprot. The spectra were searched 2 times separately by setting 1) semi-tryptic digestion with deamidated (N), oxidation (M) and Gln->pyro-Glu (Any N-term Q); 2) tryptic digestion with deamidated (N, Q), oxidation (M, H, W) and Gln->pyro-Glu (Any N-term Q), amidine (Any N-term), carbamidomethyl (Any N-term, H, K), carbamyl (K), dioxidation (C, M, W), formyl (Any N-term), and sulfide (C).

For identification of *N*-linked deglycopeptides of serum spiked with glycoprotein standards (HRP and ovalbumin), the spectra were searched against the protein database of *Homo sapiens* together with 5 proteins from *Armoracia rusticana* and 16 proteins from *Gallus gallus* (73,152 entries). The variable modification setting was the same as that for serotransferrin.

All other parameters were set as default. Finally, for each search result, identified peptides that have “deamidated[N]” modification were filtered with 1% FDR at the peptide level by the pBuild software. Spectra file named X.spectra.txt of identified *N*-linked deglycopeptides were exported for further processing.

##### Construction of N-glycan Mass Database

Three N-glycan databases were retrieved from GlycomeDB (15), resulting in 194, 7, and 51 distinct glycan masses annotated with *Homo sapiens*, *Armoracia rusticana* and *Gallus gallus* after removing the redundant masses from isomeric glycan structures. Another N-glycan database with 701 N-glycan masses annotated with *Homo sapiens* and unknown species from GlycomeDB has been used in the development of pGlyco2.0 (5). Then the two databases with 194 and 701 N-glycan masses were combined to construct a new one, which contained 739 N-glycan masses and was mainly made of four kinds of common monosaccharides, *i.e.* hexose (H), N-acetylhexosamine (N), fucose (F), and N-acetylneuraminic acid or sialic acid (NANA, S) and less contained N-glycolylneuraminic acid (NGNA,G). The format of 194, 701, 739, and 51 N-glycan masses used in pMatchGlyco is H-N-S-G-F. N-glycan masses associated with *Armoracia rusticana* also contained xylose, specifically pentose(P). The format of 7 N-glycan masses includes H-N-S-G-F and H-N-S-G-F-P.

##### Identification of N-linked Intact Glycopeptides

The pMatchGlyco software (11) was used to identify *N*-linked intact glycopeptides. Briefly, data sets from *N*-linked intact glycopeptides were converted from “raw” to “mgf” format using pParse 2.0 with the co-elute parameter switched on (co-elute = 1) and converted files with the '_HCDFT' suffix remained. A spectral library of *N*-linked deglycopeptides was constructed using pMatchGlyco from identified *N*-linked deglycopeptides in UDGP and FDGP data sets. The identified *N*-linked deglycopeptides were first filtered with the N-glycosylation sequon of N-X-S/T/C/V (X≠P), which are the well-established *N*-linked glycosylation motifs. HILIC enrichment and filtration with a sequon (N-X-S/T/C/V, X≠P) could effectively remove the peptides with deamidation of this asparagine residue generated during high-pH RP-LC fractionation. The spectral library of *N*-linked deglycopeptides was finally constructed by adding theoretical Y0-Y5 ions to each of MS/MS spectra of the identified *N*-linked deglycopeptides. Ten ppm precursor mass tolerance and 20 ppm fragment mass tolerance were used during library construction; parameter “theta” was set as 0; introduced modification was set as “deamidated[N].” Relative intensities of added Y ions were set as 0.2. The oxonium ion-containing MS/MS spectra were extracted using oxonium ion 138.055 Da. For fragment ions, 0.02 Da mass tolerance was used during library construction, and 10 ppm precursor mass tolerance and 20 ppm fragment mass tolerance were used during searching of *N*-linked intact glycopeptides.

N-linked deglycopeptides identified in UDGP and FDGP through setting different enzymatic digestion or variable modifications and searching them seven times were combined for serum *N*-linked deglycopeptides library construction.

The parameter of minimal number of matched peptide ions was set as “3” and “4” for UGP and FGP data sets, respectively. Three N-glycan mass databases containing 194, 701, and 739 N-glycan masses respectively were used.

For each glycoprotein standard, a spectral library was generated using its identified *N*-linked deglycopeptides. A database with 7 N-glycan masses retrieved from GlycomeDB annotated as species of *Armoracia rusticana* (16) was used for identifying HRP *N*-linked intact glycopeptides. A database with 51 *Gallus gallus* N-glycan masses retrieved from GlycomeDB was used for identifying ovalbumin *N*-linked intact glycopeptides. A database with 739 N-glycan masses was used for identifying transferrin *N*-linked intact glycopeptides. Relative intensities of added Y ions were set as 0.2. Minimal number of peptide ions was set as 4. The four *N*-linked Glycan motifs, *i.e.* N-X-S/T/C/V(X≠P) were selected. The oxonium ion-containing MS/MS spectra were extracted using oxonium ion 138.055 Da from the UGP and FGP data sets.

##### FDR Estimation of N-linked Intact Glycopeptide Identification

pMatchGlyco uses the target-decoy strategy to estimate the FDR of *N*-linked intact glycopeptide identifications. Briefly, in the library construction step, for each target spectrum, a decoy spectrum was generated with the same precursor mass and charge status. The decoy sequence was obtained by reversing the target sequence but maintaining the sequon of N-X-S/T/C/V(X≠P), by which means the *N*-linked intact glycopeptide feature was reserved. Then, the corresponding decoy spectrum was generated with explained peaks (b/y ions) moved to the new *m*/*z* positions determined by its reversed sequence, and other peaks including Y0-Y5 ions were unchanged. Search results were filtered by their pMatchGlyco scores and the FDR was estimated using the formula FDR = D/T, where D and T represent the numbers of matches to the decoy and target spectra, respectively (17). To estimate the FDR accurately, glycopeptide spectrum matches (GPSMs) were separated into four classes according to their N-glycosylation motifs (N-X-S/T/C/V), and 1% FDR was applied within each class. Before FDR estimation, the motif of each glycosite in each glycopeptide's peptide backbone was determined for each GPSM by pMatchGlyco. Based on the motif found on each glycosite, the FDRs of GPSMs were separately estimated for the four motif classes using target-decoy approach, which was called separate-FDR (18). After FDR estimation, the pMatchGlyco score that represented 1% separate-FDR (18) was set as a cutoff to remove low-quality GPSMs. The remaining high-quality GPSMs were further filtered using the in-house matlab code. This filtering step minimized precursor m/z once a spectrum was considered as a co-elution case by pParse 2.0. In this way, if a spectrum was determined by pParse 2.0 to have more than one precursor m/z and was identified by pMatchGlyco as more than one GPSMs under 1% separate-FDR control, the results needed to be filtered if the GPSMs had their precursor m/z in the fashion of isotopic peaks, *i.e.* they had the same charge state but were 1-Da different in their masses. According to the peptide backbone match, these GPSMs derived from isotopic peaks were filtered in two ways, that is (1) the GPSM that had the smallest precursor m/z was kept when the peptide backbone was identical, and (2) the GPSM that had the highest pMatchGlyco score was kept when the peptide backbone was different. Except for these isotopic peak derived GPSMs, multiple GPSMs identified from one spectrum were kept and regarded as true co-elution.

Identified *N*-linked intact glycopetides were manually imported into Skyline (version 4.1, MacCoss Lab, University of Washington). The precursor m/z of a glycopeptide as well as its second to fifth isotopic peaks were used to extract ion chromatograph with a 10 ppm of mass error tolerance. Peak areas were calculated using the integration function embedded in Skyline. A similarity-based idotp value given by Skyline based on the theoretical isotopic distribution was used to determine the correct peak. The theoretical isotopic distribution was also obtained by inputting the chemical composition of a glycopeptide into a web tool (https://www.envipat.eawag.ch/index.php). Chemical composition of a glycan was obtained using an excel template downloaded from http://www.ionsource.com/Card/carbo/carbstr.htm.

##### Validation of Identified N-glycan Masses Using Entrapment Masses

To generate entrapment masses, we used random numbers from 1.5–14.5 Da with 1-Da interval as the mass shifts, and all entrapment masses were required to have at least 1-Da mass difference from any of the original N-glycan masses to be distinguishable from the latter in the defined mass tolerance.

##### Validation of the Method Using Glycoprotein Standards

To validate the accuracy of this strategy, a set of non-human glycoproteins including HRP, ovomucoid (IOVO), alpha-1-acid glycoprotein (Ogchi), ovalbumin (OVAL) and sulfhydryl oxidase 1(QSOX1) were spiked into serum and the change of identified N-glycan masses were analyzed.

## RESULTS

### 

#### 

##### Identification of N-linked Deglycopeptides

The two data sets FDGP and UDGP underwent protein sequence database search for *N*-linked deglycopeptide identification. During database search, the naturally existed or artificial post-translational modifications (PTMs) of serum proteome makes it necessary to set variable modifications in order to increase the depth of *N*-linked glycoproteome. Semi-tryptic cleavage was selected as enzymatic digestion because the truncated forms of proteins widely exist in human serum. It is time-consuming if too many variable modifications are set for searching spectra from dozens of LC-MS/MS runs against the human proteome database, and therefore it is favorable to know which modifications truly exist in the sample. The development of open mass search algorithm addresses this issue to some extent. The algorithm is embedded in software tools such as ProteinPilot (Sciex), Proteome Discoverer (Thermo Scientific), Peaks Studio (Bioinfomatics Solutions), pFind and so forth. To find the prevalent PTMs in human serum proteome, we used the recently published tool PTMiner (19) to identify the possible modifications in the UDGP data set after open mass searching using pFind v2.8.8 (supplemental Table S1). From this result, 16 common variable modifications resulted from 9 types of mass shifts were observed, including deamidated (N, Q), oxidation (M, H, W) and Gln->pyro-Glu (Any N-term Q), amidine (Any N-term), carbamidomethyl (Any N-term, H, K), carbamyl (K), dioxidation (C, M, W), formyl (Any N-term), and sulfide (C). Fifteen kinds of these modifications showed considerable frequencies in serum *N*-linked deglycopeptides (supplemental Fig. S1). N-terminal glutamate to pyro-glutamate conversion was among them, because TFA buffer was used during HILIC enrichment, which might cause this modification (20). Accordingly, some *N*-linked deglycopeptides with this modification were identified in both UDGP and FDGP data sets (supplemental Table S2). Notably, 51.04% (467/915) and 42.22% (1150/2724) of identified *N*-linked deglycopeptides were semi-tryptic peptides in UDGP and FDGP, respectively (supplemental Table S2). It is necessary to consider semi-tryptic digestion in database search for the identification of serum *N*-linked deglycopeptides.

Under 1% FDR, 915 and 2,724 *N*-linked deglycopeptides corresponding to 444 and 1536 *N*-linked glycosites from 219 and 724 *N*-linked glycoproteins were identified in UDGP and FDGP, respectively (Table I, supplemental Table S2). The number of *N*-linked glycoproteins, glycosites and deglycopeptides were increased by 249%, 283%, and 264%, respectively with the combination of ACN precipitation and high pH RP-LC fractionation. These results revealed that the low and high abundant protein fractionation and *N*-linked deglycopeptide fractionation together significantly increased the depth of serum N-glycoproteome. A total of 3328 *N*-linked deglycopeptides have been identified in human sera after combining the results from UDGP and FDGP. They were from 764 *N*-linked glycoproteins carrying 1699 *N*-linked glycosites. The coverage of serum N-glycoproteome identified by this strategy was increased to 66.4% (764/1151).

**Table I:** Statistics of identifications from N-linked deglycopeptide samples

Indicator	Data set		Combined	% of identifications increased by pre-fractionation
	UDGP	FDGP		
Glycoproteins	219	724	764	249
Glycosites	444	1536	1699	283
Deglycopeptides	915	2724	3328	264

##### Identification of N-linked Intact Glycopeptides by pMatchGlyco with Three N-glycan Databases

**Fig. 2.**
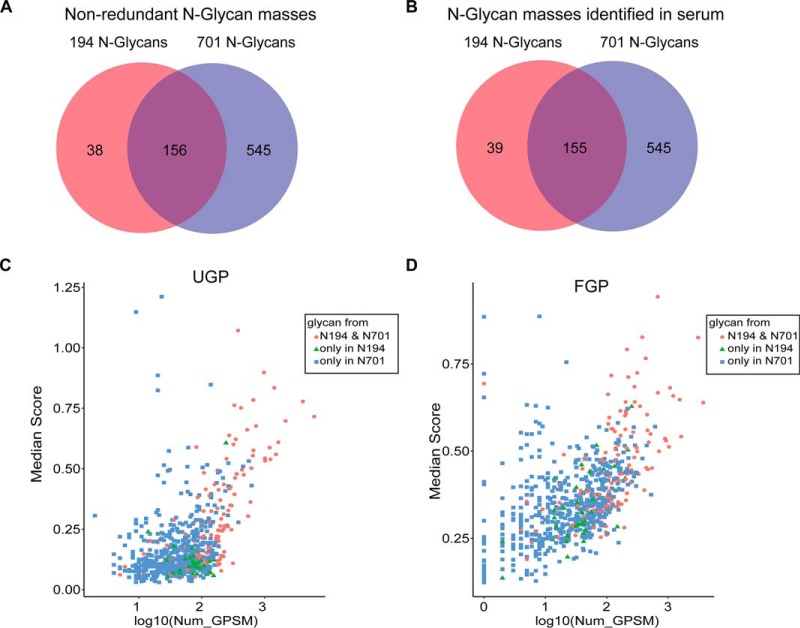
**Overlap analysis of the two glycan mass databases used and the N-glycan masses identified.** *A*, Shared and unique N-glycan masses between 194 N-glycan masses database and 701 N-glycan masses database. *B*, Commonly identified N-glycan masses using 194 and 701 N-glycan masses databases in FGP and UGP data set. *C*, *D*, The scattering plot of median pMatchGlyco score and number of GPSM for each identified N-glycan mass in the two serum data sets using the 739 N-glycan masses.

It is a very important step to establish a relatively complete human-specific N-glycan composite database for identification of N-glycoproteome by pMatchGlyco. One thousand four hundred seventy-nine kinds of human-specific N-glycans were first retrieved from GlycomeDB, resulting in a new database with 194 distinct N-glycan masses after removing the redundant masses from isomeric glycan structures. In this N-glycan mass database, 27 kinds of masses were from N-glycans containing sulfate, amino, phosphate group, and other rare monosaccharides such as N-glycolylneuraminic acid (Neu5Gc, NGNA) and 2-keto-3-deoxy-d-glycero-d-galacto-nononic acid (KDN). They were defined as modified N-glycans (supplemental Table S3), and the other 167 masses were non-modified N-glycans, which were made of four kinds of common monosaccharides, *i.e.* hexose (H), N-acetylhexosamine (N), fucose (F), and N-acetylneuraminic acid or sialic acid (NANA or S). Another N-glycan database with 701 N-glycans annotated with human and unknown species from GlycomeDB has been used in the development of pGlyco2.0 (5) by considering N-glycans from human and other unknown species (supplemental Table S4). The two N-glycan masses databases shared with 156 non-modified N-glycan masses, and were first applied in this study for comparison (Fig. 2*A*).

**Fig. 3.**
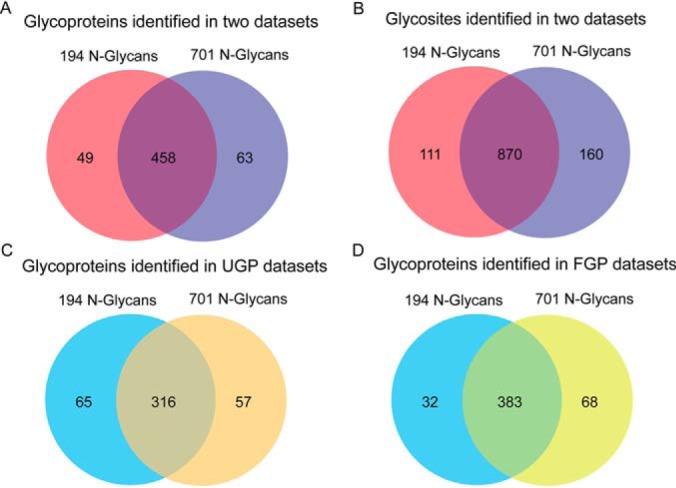
**Overlap of the identified serum glycoproteins and glycosites using the two glycan mass databases.**

Like GPQuest, open mass spectral library search was employed in pMatchGlyco to find suitable glycan composites from the N-glycan mass database, which could explain the precursor ion mass shift between a query spectrum and a library spectrum. Once a suitable glycan was found, this GPSM underwent scoring and 1% FDR filtering through target-decoy approach. To increase the sensitivity of 1% FDR filtering, all GPSMs were grouped according to their N-glycosylation motifs observed on their peptide backbones. There are four known motifs (NXS/T/C/V, X≠P) included in pMatchGlyco, therefore, 1% separate-FDR filtering was applied based on four groups. After 1% separate-FDR filtering, we observed that a subset of identified glycopeptide precursor ions determined by pParse software were prone to be isotopic peaks, because their charge states were the same, and their precursor ion masses differed by 1 Da. When these glycopeptides have identical peptide backbone, and their precursor ions are from isotopic peaks, their pMatchGlyco score sometimes are identical. In this situation, the 1-Da mass difference of their precursor ions are explained by two glycan masses with 1-Da mass difference. Therefore, these GPSMs from isotopic peaks should be deleted and their monoisotopic peak represents the unique identity. The challenge of recognizing these isotopic peaks by manual check results from their poor isotopic distribution. This is called monoisotopic peak assignment error, which is not uncommon when the abundance of precursor ion is low, and its ionization is suppressed by the high-abundance ions. To increase the confidence of GPSMs identified by pMatchGlyco, multiple GPSMs from one spectrum that have the same charges were further filtered according to their peptide backbones. That is, if their peptide backbones are identical, the GPSM with the smallest *m*/*z* is kept. If their peptide backbones are different, the GPSM that has the highest pMatchGlyco score is kept. Based on the above strategy, 1030 *N*-linked glycosites and 22,194 *N*-linked intact glycopeptides from 521 *N*-linked glycoproteins were identified from UGP and FGP data sets when using the database of 701 N-glycan masses (Table II, supplemental Table S5, S6, and S7). In comparison, 981 *N*-linked glycosites and 13,874 *N*-linked intact glycopeptides from 507 *N*-linked glycoproteins were identified when using the database of 194 N-glycan masses (Table III, supplemental Table S8, S9, and S10). One hundred fifty-five N-glycan masses were commonly identified using 194 and 701 N-glycan masses databases in FGP and UGP data set (Fig. 2*B*). The 155 N-glycan masses showed higher median pMatchGlyco scores and more GPSMs compared to other N-glycan masses (Fig. 2*C* and *D*). There were 80.4% and 76.2% overlaps of identifications at glycoprotein and glycosite levels respectively (Fig. 3). This comparison revealed that the constructed *N*-linked deglycopeptide library covers most of the occupied serum *N*-linked glycosites. The number of *N*-linked intact glycopeptides increased by 8320 when using 701 *N*-linked glycan masses database compared to that using 194 N-glycan masses database, indicating that the number of human N-glycans in GlycomeDB database is underestimated. A comprehensive human N-glycome is needed to facilitate the in-depth study of human serum *N*-linked glycoproteome. The 194 and 701 N-glycan masses were then combined. The new database contained 739 N-glycan masses and was used to re-analyze the two serum data sets UGP and FGP. In total, 526 *N*-linked glycoproteins, 1036 *N*-linked glycosites, 22,677 *N*-linked intact glycopeptides and 738 N-glycan masses were identified, which represent the most in-depth N-glycoproteome in serum identified by LC-MS/MS at *N*-linked intact glycopeptide level (Table IV, supplemental Table S11, S12, and S13). Compared to the result obtained using the 701 N-glycan masses, the number of identifications increased by 2.3% (12/521), 2.9% (30/1,030), and 5% (1109/22,194) at the glycoprotein, glycosite and glycopeptide levels when using the combined glycan mass database (supplemental Table S7 and supplemental Table S13).

**Table II:** Statistics of identifications from N-linked intact glycopeptide samples using 701 N-glycan masses

Indicator	Data set		Combined	Identifications increased by pre-fractionation
	UGP	FGP		
Glycoprotein	373	451	521	148
Glycosite	676	856	1030	354
Glycopeptide	7141	17,482	22,194	15,053

**Table III:** Statistics of identifications from N-linked intact glycopeptide samples using 194 N-glycan masses

Indicator	Data set		Combined	Identifications increased by pre-fractionation
	UGP	FGP		
Glycoprotein	381	415	507	126
Glycosite	690	767	981	291
Glycopeptide	5393	10,448	13,874	8481

**Table IV:** Statistics of Identifications from N-linked glycopeptide samples using 739 N-glycan masses

Indicator	Data set		Combined	Identifications increased by pre-fractionation
	UGP	FGP		
Glycoprotein	378	446	526	148
Glycosite	685	847	1036	351
Glycopeptide	7254	17,854	22,677	15,423

This strategy enables elucidating the micro-heterogeneity of N-glycan in serum glycoproteins, as exemplified by MMRN1, LRP1 and APOB. They are the top three proteins with the most glycosites identified in deglycopeptide samples including UDGP and FDGP. 20, 18 and 18 glycosites were identified from them, respectively (supplemental Table S2). In the intact glycopeptide data sets UGP and FGP, 11, 7, and 17 glycosites, 46, 40 and 281 glycopeptides, 45, 40, and 165 N-glycans were identified from MMRN1, LRP1 and APOB, respectively (supplemental Table S13).

##### Validation of Identified N-glycan Masses Using Entrapment Masses

**Fig. 4.**
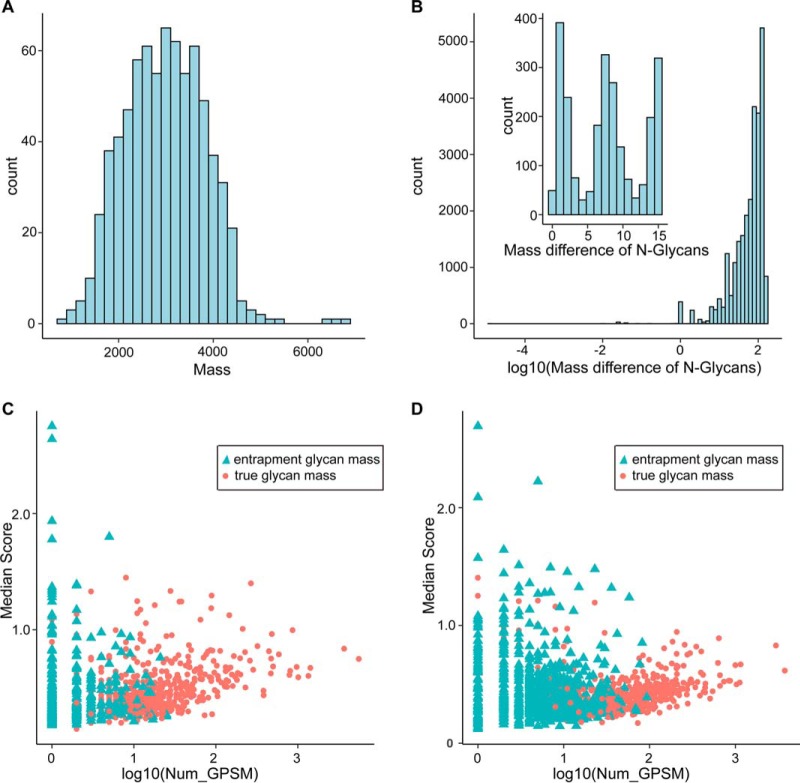
**Validation of the proposed method using entrapment glycan masses.** *A*, The distribution of 739 N-glycan masses used for serum *N*-linked intact glycopeptide identification. *B*, The distribution of mass difference in the range of 0–150 Da among the 739 glycan masses. Insert shows the frequencies of mass difference in the range of 0–15 Da. *C*, *D*, Scattering plots of all matched glycan masses after including entrapment masses. All identified GPSMs in UGP (*C*) and FGP (*D*) using the 739 true N-glycan masses and 739 entrapment N-glycan masses are combined for plotting.

The distribution of 739 N-glycan masses used for serum *N*-linked intact glycopeptide identification is shown in Fig. 4*A*, which is close to a normal distribution. The distribution of mass difference in the range of 0–150 Da among the 739 glycan masses is shown in Fig. 4*B* and the insert figure shows the distribution of mass difference in the range of 0–15 Da, which is non-normal distribution. The number of PSMs and the score of each PSM are established indicators of identification confidence. To evaluate the identification confidence of N-glycan masses, scattering plot of the GPSM number and the corresponding median pMatchGlyco score was used for all matched glycan masses including entrapment masses. All identified GPSMs in UGP (Fig. 4*C*) and FGP (Fig. 4*D*) using the 739 true N-glycan masses and 739 entrapment N-glycan masses were combined for scattering plotting. The results showed that identified entrapment N-glycan masses corresponded to less GPSMs and lower pMatchGlyco median score in both UGP and FGP. In addition, 685 (92.8%) shared N-glycan masses were identified in UGP and FGP (supplemental Table S14). Moreover, the N-glycan composites Hex_5_HexNAc_4_NANA_2_ and Hex_5_HexNAc_4_NANA_1_ are reported to be the most abundant ones carried by human serum glycoproteins. In consistence, these two glycan composites ranked the first and second one based on the number of GPSMs in the two serum data sets.

After combining the original masses with the entrapment masses and analyzing the two serum data sets, only 2.6% (969/37,087) and 8.6% (5,512/64,454) of GPSMs in UGP and FGP matched to the entrapment glycan masses (supplemental Table S15), suggesting that the likelihood of incorrect glycan identification was acceptable. Because some of the entrapment masses may be truly existed ones, this may explain why some entrapment masses were matched with high scores or many GPSMs. In this case, the error rate is overestimated. Therefore, we cannot use the entrapment glycan masses as a rigorous strategy to estimate and control the FDR of glycan identification. Instead, we used this strategy as an assistant method to validate the identification result.

##### Validation of the Method Using Glycoprotein Standards

We then used commercial glycoprotein standards (HRP, ovalbumin, serotransferrin) as well as the serum spiked with these proteins (HRP and ovalbumin) to validate our identification strategy. The classical serum glycoprotein serotransferrin was used as a benchmark protein, as it was identified with thousands of GPSMs in the human serum data sets. From the commercial serotransferrin protein standard, we identified five glycosites and varied number of glycan masses on each site (supplemental Table S16). This indicates it is feasible to use the proposed workflow in identifying *N*-linked intact glycopeptides in commercial protein samples. However, we identified 54 other proteins in the deglycosylated serotransferrin protein standard sample (supplemental Table S17), suggesting that its purity is not 100%. Moreover, 16 proteins were identified from the deglycosylated ovalbumin standard sample (supplemental Table S18). Among the 16 proteins, there are four glycoproteins including IOVO, Ogchi, OVAL and QSOX1, and most of the GPSMs identified from this sample were from IOVO instead of OVAL (supplemental Table S19). This reflected the difficulty in obtaining a glycoprotein standard with high purity.

**Fig. 5.**
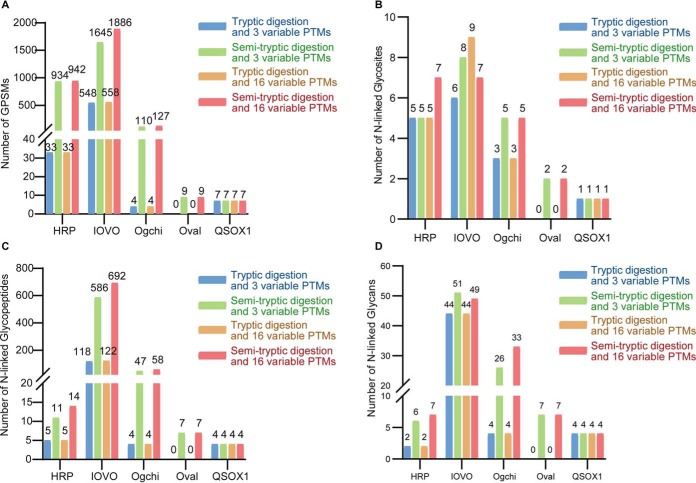
**Validation of semi-tryptic digestion and 16 PTMs setting using glycoprotein standards and serum spiked with glycoprotein standards.** Four different set of search parameters are used to identify glycopeptides from five non-human glycoprotein standards and their GPSMs (*A*), *N*-linked glycosites (*B*), *N*-linked intact glycopeptides(*C*) and number of N-glycans (D) are compared. HRP, horseradish peroxidase; IOVO, ovomucoid; Oval, ovalbumin; Ogchi, alpha-1-acid glycoprotein; QSOX1, sulfhydryl oxidase 1.

The plant glycoprotein HRP and the four chicken glycoproteins including IOVO, OVAL, Ogchi and QSOX1 were used to validate the setting of database search parameters. 7 N-glycan masses from *Armoracia rusticana* and 51 N-glycan masses from *Gallus gallus* based on species annotation in GlycomeDB were used to identify corresponding *N*-linked intact glycopeptides. Four different sets of search parameters were compared, and it showed semi-tryptic digestion and 16 PTMs returned the most GPSMs from the five proteins (Fig. 5*A*, supplemental Table S20). Notably, the two glycosites of ovalbumin, *i.e.* N293 and N312 were identified only by setting semi-tryptic digestion (Fig. 5*A*–5*D*, supplemental Table S20). Compared to the result of setting tryptic digestion and 3 variable PTMs, the major increase of GPSMs and N-glycan masses identified resulted from setting semi-tryptic digestion instead of setting 16 variable PTMs (Fig. 5*A*–5*D*, supplemental Table S20). Manual check of these novel GPSMs by setting semi-tryptic digestion confirmed that they were true identifications, supporting the setting of semi-tryptic digestion and 16 variable PTMs in the proposed workflow.

To validate the accuracy of this strategy, we spiked a set of non-human glycoproteins including HRP, IOVO, Ogchi, OVAL, and QSOX1 into serum and analyzed the change of identified N-glycan masses. After spiking them into serum, the number of identified N-glycan masses increased by 8 and 1 on the *N*-linked glycosites N293 and N312 of OVAL, the number of identified N-glycan masses increased by 2, 5, 5, 5, and 6 on the *N*-linked glycosites N99, N93,N77, N34, N199, of IOVO, the number of identified N-glycan masses increased by 5, 3, and 2 on the *N*-linked glycosites N36, N90 and N82 of Ogchi, the number of identified N-glycan masses didn't increased in QSOX1 though glycosite of N371 was identified after spiking it into serum (supplemental Table S21), and the number of identified N-glycan masses in HRP didn't increased (supplemental TableS 22). These results reflect the effectiveness of this strategy. An alternative way of evaluating the FDR is by searching the intact glycopeptides' spectra from a glycoprotein standard against the protein and glycan mass database from another unrelated species. When searching the *N*-linked intact glycopeptide data sets from ovalbumin standard against human serum *N*-linked deglycopeptide library and the 739 N-glycan masses library, only 12 GPSMs were returned. Compared to the 2041 GPSMs obtained using the *N*-linked deglycopeptide library and N-glycan masses database derived from Gallus gallus, it suggested the FDR of the proposed workflow was 0.59%.

HRP contains Hex_3_HexNAc_2_Pent_1_Fuc_1_ as the major N-glycan composite, which is unable to be processed by PNGase F (21, 22). However, some other N-glycans that can be processed by PNGase F are reported to be carried by HRP. Therefore, the *N*-linked intact glycopeptides carrying this major N-glycan in HRP may be identified using the *N*-linked deglycopeptides carrying other N-glycans that can be processed by PNGase F. We identified this major glycan on 5 glycosites including N87, N216, N228, N285, and N298 in commercial HRP (supplemental Table S22).

##### Complement and Coagulation Cascades as the Most Enriched Pathway for Serum Protein Glycosylation

**Fig. 6.**
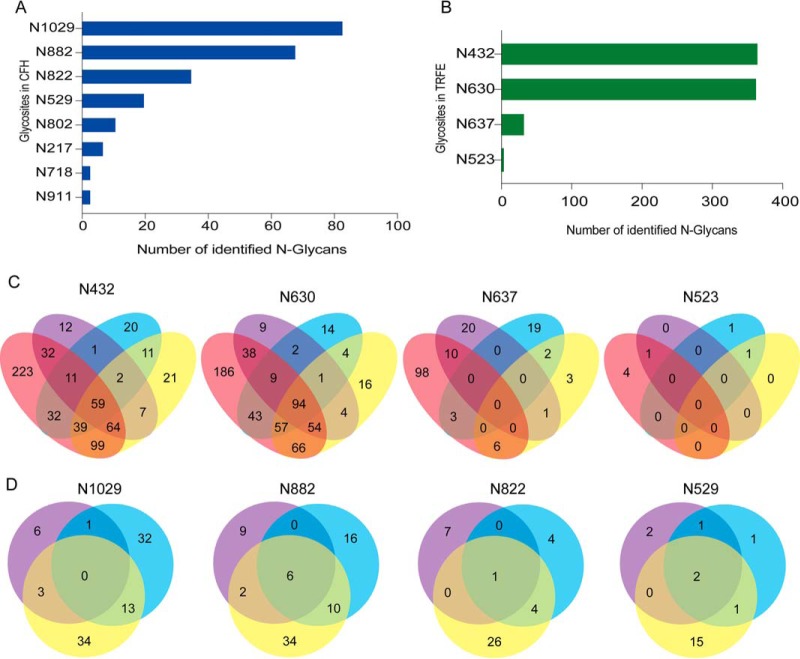
**Site-specific glycosylation of serum glycoproteins.** The number of identified N-glycan masses on each of the eight glycosites of complement factor H (*A*) and on each of the four glycosites of serotransferrin (*B*) are shown. *C*, *D*, The overlap of N-glycan masses identified on the four glycosites N432, N630 and N637 of serotransferrin and on the four glycosites N529, N822, N882 and N1029 of complement factor H in different samples including commercial transferrin, serum spiked with HRP and ovalbumin, UGP and FGP data set. Pink ellipse represents the N-glycan masses from commercial serotransferrin. Purple ellipse represents the N-glycan masses from human serum spiked with HRP and ovalbumin. Cyan ellipse represents the N-glycan masses from the UGP data set. Yellow ellipse represents the N-glycan masses from FGP data set. *CFH*, complement factor H; *TRFE*, serotransferrin.

The identified 526 serum N-glycoproteins were mapped to biological pathways using the KEGG mapping tool (23), and the significance of each pathway enriched given by the STRING database (24). It showed that complement and coagulation cascades was the most significantly enriched pathway (supplementary Document S1, supplemental Table S23), followed by PI3K-Akt signaling pathway. The complement and coagulation cascades pathway rank as the third significantly enriched pathway when using Reactome Pathway Database (25) (supplementary Document S2). Complement augments the opsonization of bacteria by antibodies and allows antibodies to kill some bacteria. The complement and coagulation cascades are thus a critical component of human innate immune system. Hence, most entities in this pathway are shared in Staphylococcus aureus infection pathway. The most enriched molecular function of the 526 serum glycoproteins is calcium ion binding and the second one is glycosaminoglycan (GAG) binding. Complement factor H (CFH) is a representative one which binds to heparan sulfate proteoglycan. CFH negatively regulates the activation of complement C3 in plasma (supplementary Document S1). It also functions by binding to host cell surface through interaction with heparan sulfate (26). There are nine known N-glycosites in CFH, from which 8 N-glycosites were identified in this study. However, the number of glycans identified on each N-glycosite varied (Fig. 6*A*, supplemental Table S24). This suggested that N-glycosylation of CFH was site-specific. Because the two sites N882 and N911 are close to each other and both are located on the surface of the fifteenth sushi repeat domain in the CFH protein structure, it is possible that N911 is less accessible to OSTs than N882. The fine three-dimensional structures of CFH and its complex with heparin sulfate that are still lacking may help to elucidate this.

##### Enrichment of Cell Adhesion Molecules and Hematopoietic Cell Linage Markers in Serum Glycoproteins

Except for the component of complement and coagulation cascades, cell adhesion molecules and hematopoietic cell linage markers (CD molecules) were also enriched in the identified serum glycoproteins (supplementary Document S1). Most of these proteins are blood cell or neural cell surface proteins, and they may shed or secreted from cells, such as CD44 protein. Once shedding from cell surface, the soluble CD44 plays versatile biological functions which are different from cell adhesion (27). The N-glycosite N25 of CD44 locates at its extracellular part. One glycopeptide containing this site were identified using the 739 N-glycan masses. (supplemental Table S13). It is known that elevated levels of soluble CD44 in the serum of patients is a marker of tumor burden and metastasis in several cancers (28), hence, the glycans on soluble CD44 may provide more information regarding to disease status. CD44 is also a marker for T cell and erythrocyte lineage differentiation. It is reasonable to detect those CD markers in serum once they shed from progenitor cells into circulation during cell differentiation. The alteration of N-glycosylation on these CD markers may provide information of aberrant cell differentiation.

##### Modified N-glycans Carried by Serum N-linked Glycoproteins

The proposed method will achieve the highest accuracy by using a complete database of N-glycans as well as *N*-linked deglycopeptides derived from human serum. However, this database is not established to date. More than 140 N-glycans in serum was observed in a previous study (28). In another serum glycoproteomic study, about 200 kind of N-glycans were used for *N*-linked glycopeptide identification (7). To figure out the influence of size of glycan mass database, we compared the GPSM results using two distinct size of glycan mass databases, one with 701 N-glycan masses and the other with 194 N-glycan masses. The results indicated that the size of N-glycan mass database significantly influenced the number of *N*-linked intact glycopeptides. It also influenced the number of *N*-linked glycoprotein or *N*-linked glycosite but was insignificant. There are 21 kind of N-glycan masses with sulfonation or phosphorylation in the 194 N-glycan masses database. Sulfated N-glycans on IgG were reported to be biomarkers for rheumatoid arthritis recently (29). The two glycan mass databases were thus combined and used to analyze the two serum *N*-linked intact glycopeptide data sets. In total, 138 serum *N*-linked glycoproteins with 192 *N*-linked glycosites were identified to carry these modified N-glycans (supplemental Table S25). Consistent with the previous study, we also identified sulfated N-glycans on the heavy chain of IgG. Besides IgG, these sulfated N-glycans were identified on the heavy chain of IgM, IgA, and in other serum proteins such as prothrombin and heparin cofactor 2. Representative structures of modified N-glycans identified are present in the supplementary Document S3. It is thus critical to include modified N-glycans when identifying serum *N*-linked intact glycopeptides.

##### Site-specific Glycoform and Its Functional Insight

Glycoproteins that differ only in their N-glycan composition are termed glycoforms (30). Because N-glycosylation is site specific, it is reasonable to study glycoforms in a site-specific fashion. The identified *N*-linked intact glycopeptides using 739 N-glycan masses database were used to construct glycoform for a given glycosite. Serotransferrin and complement factor H were selected as examples. There were four *N*-linked glycosites of serotransferrin identified, *i.e.* N432, N630, N637, and N523 (Fig. 6*B*, supplemental Table S26). The number of N-glycans on those glycosites of serotransferrin varied among commercial serotransferrin, serum spiked with glycoproteins, UGP and FGP data set (Fig. 6*C*). The number of N-glycans on N529, N822, N882, and N1029 of complement factor H also varied among serum spiked with glycoproteins, UGP and FGP data set (Fig. 6*D*).

Among the mentioned glycosites of serotransferrin, N523 and N637 are not reported in Uniprot. Both are located on an uncanonical NXC motif. A recent study showed that N523 and N637 were both occupied by N-glycans (31). Notably, the three glycosites N432, N630 and N637 were reported to locating at the interface of serotransferrin and pathogenic *Neisseria* transferrin-binding protein A (TbpA) (supplemental Fig. S2) (32). The two N-glycan conjugates located on N432 and N630 hold TbpA and may stabilize their binding. More than three hundred N-glycan masses were identified on the two sites, whereas two N-glycan masses were identified on N523. The two glycan composites are Hex_5_HexNAc_4_NANA_2_ and Hex_5_HexNAc_4_NANA_1_. The latter one was initially identified as Hex_5_HexNAc_4_Fuc_2_ by pMatchGlyco but was corrected to Hex_5_HexNAc_4_NANA_1_ after manually checking its isotopic distribution (supplemental Fig. S3). The glycosite N523 is distant from the binding interface, hence it has the least number of N-glycan masses identified. In this regard, the number of N-glycan mass identified on the four N-glycosites of serotransferrin might indicate the accessibility of each site to OSTs and their functional importance. From the site-specific glycoform result of serotransferrin, it is easy to obtain a structure-function perspective of N-glycosylation of serotransferrin in pathogen-host interaction. However, its roles in binding to its cognate receptor and other human proteins remain elusive. As shown in supplemental Fig. S4, the two N-glycosites of serotransferrin and the two N-glycosites of transferrin receptor 1 identified in our work don't approach to each other in their protein complex's structure. A possible role of N-glycans in their binding is to form a lattice first on the cell surface in a head-to-head fashion. After anchoring by N-glycans, serotransferrin moves to cell surface and binds to its receptor side by side. This, however, needs further study to validate.

##### Quantitation of Site-specific Glycoforms

Carbohydrate-deficient transferrin (CDT) is a laboratory test that is used widely in detecting abusive alcohol consumption (33). It also indicates defects in N-glycan synthesis that is characterized by the ratio of mono-oligosaccharide/di-oligosaccharide serotransferrin, a-oligosaccharide/di-oligosaccharide serotransferrin and tri-sialo/di-oligosaccharide serotransferrin (34). These ratios can be accurately estimated by quantitative analysis of each glycan in a site-specific manner, because the intact protein is digested into tryptic peptides.

**Fig. 7.**
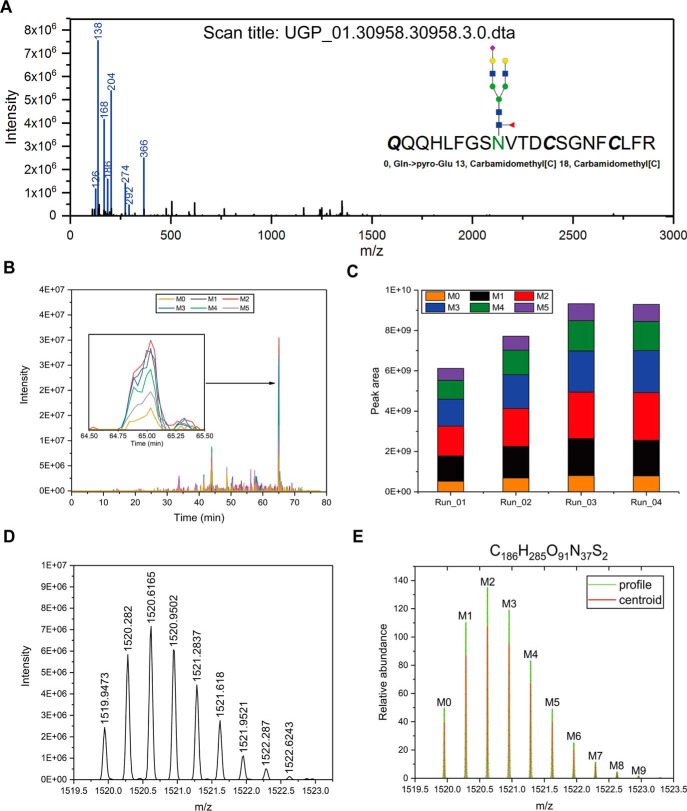
**A glycopeptide spectrum identified by pMatchGlyco in UGP data set.** *A*, The spectrum is shown in the m/z range of 0–3000. Eight commonly observed oxonium ions are colored in blue and labeled with their m/z values. *B*, Extracted ion chromatographs of the first six isotopic peaks of this glycopeptide's precursor ion. *C*, Peak areas of the first six isotopic peaks in the four replicate LC runs in UGP data set. *D*, Experimentally observed isotopic distribution of the glycopeptide precursor ion in Run_01. *E*, Theoretical isotopic distribution of the same precursor ion predicted based on its chemical composition.

To obtain the quantitative information of these glycans identified on the glycosite N630 of serotransferrin, the peak area of each precursor ion was integrated by Skyline (35). Two hundred twenty-four N-glycan masses were identified with the peptide at position 622–642 of serotransferrin (supplemental Table S26). A representative GPSM of this tryptic peptide was shown in Fig. 7*A*. In total, 363 N-glycan masses were identified on N630 of serotransferrin from UGP and FGP data sets. In general, precursor m/z and retention time are the two parameters to define a peak. Ideally a GPSM will be identified among all replicate LC-MS/MS runs (supplemental Fig. S5), enabling accurate peak extraction by the retention time of GPSM. However, in DDA employed here, it is not uncommon that a given GPSM fails to be identified in some runs, and it was difficult to keep the retention time of a given glycopeptide constant among multiple LC runs without retention time correction using standard peptides. For example, the spectrum in Fig. 7*A* was collected at 65.05 min in UGP_01, whereas the retention time of this glycopeptide shifted in other three LC-MS/MS runs (supplemental Fig. S6). This was confirmed by the extracted ion chromatograms of the spectrum's precursor ion (Fig. 7*B*). The peak area of this N-linked intact glycopeptide was exported by Skyline (Fig. 7*C*). Isotopic distribution is another inherent character of analyte in LC-MS/MS analysis except for its retention time. Therefore, the elution profile of isotopic peaks M0-M6 of this precursor ion on MS1 (Fig. 7*D*) was compared with its theoretical distribution (Fig. 7*E*). An idotp value was further given by Skyline, suggesting a high similarity (0.99) between observed profile and theoretical profile in all four runs (supplemental Fig. S6). We thus used idotp value together with retention time of GPSM to define the boundaries of elution profile in multiple runs. For all other *N*-linked intact glycopeptide carrying N630, a spectrum sequence list (.ssl) was generated and exported as a spectral library for MS1 peak area-based quantification using Skyline. For quantitative study of site-specific glycosylation in human serum using peak areas on LC-MS/MS, it needs to consider the numerous variants of *N*-linked intact glycopeptides containing the same N-glycosite, such as N630 of serotransferrin. These variants include semi-tryptic peptides resulting from un-specific digestions, modifications and distinct charge states of precursor ions. If the peak area of all glycopeptide variants carrying the same glycosite and glycan is summed and compared among samples, it will reflect the site-specific abundance change of this glycan composite among samples in an unbiased way. This, however, needs a high-throughput software tool to recognize all glycopeptide variants and sum their peak areas, which is not achieved in this study.

## DISCUSSION

Compared with published workflows for *N*-linked intact glycopeptide identification, the widespread presence of truncated proteins or variable proteoforms in serum has been taken into account in the proposed workflow. Semi-tryptic digestion and multiple variable PTMs were set during database search. To the best of our knowledge, these settings have not been considered simultaneously in published workflows, as they will increase the searching space. We used a set of glycoprotein standards to validate the setting of semi-tryptic digestion, and the results showed semi-tryptic digestion dramatically increased the number of confident GPSMs of these protein standards' glycopeptides. In the case of OVAL, it was necessary to set semi-tryptic digestion in order to identify its *N*-linked deglycopeptides. In addition, 16 kinds of variable PTMs were considered when setting tryptic digestion. By comparing the GPSMs, glycosites and N-glycan masses obtained from these standards and the two human serum data sets under four different sets of search parameters, it showed more than 80% GPSMs were shared by setting 16 variable PTMs and by setting 3 variable PTMs (supplemental Fig. S7). Therefore, setting 16 variable PTMs didn't introduce excessive false positive ones. We combined the database search results from setting semi-tryptic digestion plus 3 variable PTMs and from setting tryptic-digestion plus 16 variable PTMs that were introduced as five subgroups. The combined results were filtered with 1% FDR at peptide level based on the peptides identified as having an Asn deamidation modification, and the remaining peptides were used to construct serum *N*-linked deglycopeptide spectral library.

In this regard, the FDR of deglycopeptide identifications was effectively controlled, and the tryptic peptides with some of the 16 PTMs and their unmodified versions will undergo matching and scoring in parallel. This reduces the chance of mismatching because of the lack of really existed peptides containing the 16 PTMs.

It is critical to validate the GPSMs in large-scale *N*-linked intact glycopeptide identification. By filtering the GPSMs that have their precursor m/z assigned as isotopic peaks, the major part of false positive GPSMs caused by inaccurate determination of precursor m/z during data format conversion was removed. Through introducing entrapment glycan masses into search space, the accuracy of our method was validated. The degree of separation between matched true and entrapment glycan masses in the scattering plot is a good indicator of our data quality. It also helps to recognize those low-confidence N-glycan masses and glycopeptides identified, which is critical in completing the human serum N-glycan database.

Target-decoy method was used to perform FDR control at the peptide level for deglycopeptide identification. In theory, if the peptide sequence is correct, the glycan mass should be correct. In the proposed method, the target-decoy strategy was applied for FDR control of peptide identifications of GPSMs. Therefore, the peptides of GPSMs after FDR filtration should be reliable. If the peptide part (including sequence and PTMs) of a GPSM is correct, the mass of glycan part should also be correct if the precursor mass of the *N*-linked intact glycopeptide is accurate. However, there are three conditions that can lead to incorrect identification of the glycan part of a GPSM. Firstly, the identified peptide has the correct sequence but incorrect PTMs because its PTMs are unusual and not included in database search. The mass shift introduced by these unusual PTMs may be incorrectly explained as part of a glycan's mass, which leads to incorrect identification of the glycan. However, we have detected 16 abundant PTMs and included them into database search, the proportion of *N*-linked intact glycopeptides with unusual PTMs is expected to be small, and the incorrect glycan identification caused by this way is neglectable. Secondly, the precursor mass assignment is incorrect, and the mass error causes incorrect glycan identification. Finally, the glycan may carry some modifications, resulting in the change of the glycan mass, and there happens to be a glycan mass that equals to the changed glycan mass. To evaluate the rate of glycan mass errors caused by the above three conditions, we generated entrapment glycan masses and incorporated them into the glycan database (See supplementary Discussion for more details).

However, because of the presence of incorrect precursor masses and unknown PTMs, even if the peptide sequence is correct, the glycan mass may be erroneous. At GPSM level, the proportion of incorrect glycan mass identification was estimated to be less than 10%. Due to the limited knowledge of human serum N-glycome, this estimation is not strict because it is possible to include truly existed glycan mass into entrapment glycan masses. Therefore, the entrapment glycan masses were used for method validation instead of FDR control.

N-glycosylation happened at the non-canonical motif NXV(X≠P) were reported in human serum recently (7). However, stringent FDR estimation is needed in order to decrease false positives caused by including this newly identified motif, which is not addressed by employing database search strategy in *N*-linked intact glycopeptide identification to date. Here we included this motif in *N*-linked intact glycopeptide identification and adopted group-FDR strategy to separately estimate FDR in different motif subclasses. In the UDGP and FDGP data sets, we also identified 143 deglycopeptides with NXV motif. By including them into the spectral library, 26 *N*-linked glycoproteins carrying 29 *N*-linked glycosites were identified with NXV motif (supplemental Table S27). They included the previously reported glycosite N63 of alpha-1B-glycoprotein and N68 of albumin (7), suggesting that our method was reliable. However, it is unclear how this newly identified motif is different from the canonical N-glycosylation motifs in terms of its recognition and processing by OSTs.

It is well established that rare genetic defects in the assembly, attachment, and processing of glycans will produce disorders that affect multiple systems and lead to congenital disorders of glycosylation (CDG). Many serum glycoproteins are affected in CDG. We suggest that the proposed method can be used for CDG diagnosis and phenotyping. Currently, serotransferrin is used as the analyte marker to diagnose CDG because of its relatively high abundance (36). In the serum of patient with CDG-1a, the loss of an entire oligosaccharide moiety, *i.e.* Hex_5_HexNAc_4_NANA_2_ from serotransferrin was identified by MS. In our result, this glycan is identified on all the five glycosites of serotransferrin. Compared with the established methods based on immuno-capture and MS, our method avoids immuno-capture and provides more details of serotransferrin glycoform in a site-specific manner. With the glycopetides identified here, a spectral library of serotransferrin glycopeptide can be constructed and applied to DIA workflow for its glycoform quantification. This will contribute to precise diagnostics of CDG.

As part of human innate immune system, serotransferrin sequesters irons and restricts the iron-dependent growth of pathogenic bacteria. However, pathogenic *Neisseria* membrane protein TbpA can bind to serotransferrin, and obtain iron from it. The N-glycans on the surface of serotransferrin provides binding surface for TbpA. As a result, distinct N-glycan structures on the interacting interface may vary in their binding affinity with TbpA. Further study on the role of serotransferrin carried N-glycans in the pathogenesis of *Neisseria* will help us elucidating the biological role of N-glycosylation in host-pathogen interaction. This can be achieved by in-depth analysis of serotransferrin glycoform and the binding affinity of each glycan to TbpA.

Accurate identification of each N-glycan composite is a prerequisite for functional investigation of site-specific glycoform of serum glycoproteins. The proposed LC-MS/MS method for *N*-linked glycopeptide identification and site-specific glycoform construction was validated in human serum sample. It enables quantitative analysis of site-specific glycoform of serum glycoprotein, which helps to develop better approaches for diagnosis and treatment of CDG as well as other human diseases.

## DATA AVAILABILITY

The mass spectrometry proteomics data (UDGP, FDGP, UGP, FGP and data from commercial glycoproteins as well as serum spiked with HRP and Ovalbumin) and annotated *N*-linked intact glycopeptide spectra have been deposited to the ProteomeXchange Consortium via the PRIDE (37) partner repository with the data set identifier PXD015622 [http://www.ebi.ac.uk/pride]. The data set OVCAR3 was previously published (3) and can be found with the data set identifier PXD001571. The pMatchGlyco software (version 1.2) and matlab code named “SeparateFDR_MiniMax” for separate-FDR estimation and precursor m/z related GPSM filtering can be downloaded at http://fugroup.amss.ac.cn/software/pMatchGlyco/pMatchGlyco.html.

## Supplementary Material

Supplementary Discusion

Supplementary Document 1

Supplementary Figures

Supplementary Table S1

Supplementary Table S2

Supplementary Table S3

Supplementary Table S4

Supplementary Table S5

Supplementary Table S6

Supplementary Table S7

Supplementary Table S8

Supplementary Table S9

Supplementary Table S10

Supplementary Table S11

Supplementary Table S12

Supplementary Table S13

Supplementary Table S14

Supplementary Table S15

Supplementary Table S16

Supplementary Table S17

Supplementary Table S18

Supplementary Table S19

Supplementary Table S20

Supplementary Table S21

Supplementary Table S22

Supplementary Table S23

Supplementary Table S24

Supplementary Table S25

Supplementary Table S26

Supplementary Table S27

Supplementary Document 2

Supplementary Document 3

## Footnotes

^1^ The abbreviations used are:

PSA: prostate specific antigen
ACN: acetonitrile
CA-125: cancer antigen 125
CD: cluster of differentiation
CDG: congenital disorders of glycosylation
CDT: carbohydrate-deficient transferrin
CFH: complement factor H
DDA: data-dependent acquisition
DIA: data-independent acquisition
DTT: 1, 4-dithiothreitol
FA: formic acid
FDGP: fractionated deglycopeptides
FGP: fractionated glycopeptides
FDR: false discovery rate
GAG: glycosaminoglycan
GPSM: glycopeptidespectrum match
HILIC: hydrophilic interaction liquid chromatography
IAM: 2-iodoacetamide
LC: liquid chromatography
LC-MS/MS: liquid chromatography coupled tandem mass spectrometry
MS: mass spectrometry
OSTs: oligosaccharyltransferases
PDGP: deglycopeptides from precipitate
PGP: glycopeptides from precipitate
PTMs: post-translational modifications
RPLC: reversed-phase liquid chromatography
SDGP: deglycopeptides from supernatant
SGP: glycopeptides from supernatant
TFA: trifluoroacetic acid
UDGP: deglycopeptides from un-fractionated serum sample
UGP: glycopeptides from un-fractionated serum sample.
