# Supplementary material for: Large-scale Identification of N-linked Intact Glycopeptides in Human Serum using HILIC Enrichment and Spectral Library Search
Source: Mol Cell Proteomics. 2020 Feb 26;19(4):672–89. doi: 10.1074/mcp.RA119.001791 (PMC7124471; doi:10.1074/mcp.RA119.001791)
Supplement: Supplementary Discusion [file 156056_1_supp_471916_q5c98v.docx]

**Supplementary Discussion**

*Determination of precursor m/z by pParse*

Prior to spectral library search, the precursor m/z of each spectrum needs to be determined. Accurate determination of precursor m/z depends on the quality of isotopic distribution and peak recognition algorithm. In this regard, the four datasets were processed by two tools, i.e., pParse and RawConverter (1), both of which aim to better determine precursor m/z. A co-elution option in pParse enables assignment of multiple precursor m/z values to one MS/MS spectrum, which will increase the number of MS/MS spectra after data conversion. To keep the number of spectra comparable between the two tools, the co-elution option in pParse was deactivated initially. It showed that the proportions of inconsistent precursor m/z obtained by the two tools were 8% (28,914/359,898) in UDGP and FDGP, and 26.5% (206,565/778,973) in UGP and FGP. Manual check of 30 inconsistent results that were randomly selected in UGP indicated the accuracy of pParse was higher than that of RawConverter (**Supplementary Table S12**). We thus used pParse in data conversion. Furthermore, we compared the distribution of the most intense peak between glycopeptides and their de-glycosylated counterparts from human serum. In de-glycosylated peptides, 54.11% and 41.03% of precursor m/z had M0 and M1 as the most intense peak, respectively. Where as in glycopeptides the proportion was 0.03% and 17.75%, respectively (**Supplementary** **Figure 8**). In opposite, 80.53% of precursor m/z in glycopeptide had M2 or M3 isotopic peak as the most intense peak, suggesting a trend of shift of the most intense isotopic peak from M0/M1 to M2/M3 between de-glycosylated peptides and glycopeptides. Therefore, determination of precursor m/z according to the recorded m/z on glycopeptide MS/MS spectrum, which is the way that MSConvert determined precursor m/z, will often hit its M2/M3 instead of M0/M1. As expected, the m/z of most intense peak showed a positive correlation with the mass of its precursor ion. This correlation pointed out the necessity of a specific algorithm to accurately determine precursor m/z of intact glycopeptide. This may explain the less accuracy of RawConverter in determination of glycopeptide precursor m/z, since RawConverter was not trained and optimized using glycopeptide MS datasets.

Co-elution of precursor ions is another factor that challenges accurate determination of precursor m/z. If both precursor ions are selected for fragmentation by mass spectrometry, which is called co-isolation and co-fragmentation, a mixed MS/MS spectrum will be generated. An isolation window of 2 m/z was applied in this work to collect MS/MS spectra of glycopeptides (see **Experimental Procedures**). Therefore, co-isolation of precursor ions was not uncommon. The co-elution option in pParse was activated to investigate the mixed spectra from glycopeptides. One example was shown in Supplementary Figure 8. In this case, the resolution power allowed differentiating the two co-isolated precursor ions and determining each precursor m/z by its isotopic distribution in the elution profile. However, the MS/MS spectrum will contain fragments from each of the co-isolated precursor ions. During this elution profile, there were three MS/MS spectra with scan number 11156, 11159 and 11178 generated by the same set of co-isolated precursor ions (**Supplementary Figure 9** and **10**). Once the co-elution option in pParse was activated, it converted each of the three mixed MS/MS spectra into two spectra, which contained same fragment peaks but with two distinct precursor m/z values as well as charge states. After search by pMatchGlyco, two glycopeptide-spectrum matches (GPSMs) named GPSM1 and GPSM2 were identified from the mixed spectrum. Each of the two GPSMs explained a subset of observed peaks. Without activation of co-elution option in pParse, all of the three MS/MS spectra were determined with single precursor m/z and identified as GPSM2 only. Therefore, the co-elution option in pParse achieved identification of co-eluted and co-fragmented glycopeptide precursor ions. We thus kept activating the co-elution option when converting the data format of UGP and FGP dataset using pParse.

Notably, a subclass of precursor ions determined by pParse as co-eluted precursor ions differed by 1 Da and had the same charge (**Supplementary Figure 11**). As a result, they were identified as two glycopeptides with same peptide backbone but two 1-Da-different glycan masses. It is difficult to determine which one explained the MS/MS spectrum better by pMatchGlyco score, because all of their matched ions are same in theory. Regarding to this, the GPSM with smaller precursor m/z was kept in the final result. Though this filtering step can reduce false positives, some precursor m/z remained to be incorrect due to their poor isotopic distribution. As an example, incorrect determination of precursor m/z of one GPSM from the N523 glyosite of serotransferrin was manually corrected (**Supplementary Figure 3**). Therefore, all GPSM results need to be validated manually to confirm its precursor m/z, which is labor intensive. In this regard, we proposed a way for method evaluation by generating a decoy glycan masses database based on the 739 N-Glycan masses and combining the two glycan masses for further searching using pMatchGlyco.

*Evaluating the proposed identification method using decoy glycan masses*

According to the nature of glycopeptide MS spectra, there are two sources of false positive matches in the serum data. They are 1) the incorrect precursor m/z at MS1 level resulting in mis-matches between two glycopeptides with close masses, and 2) the ambiguous matches between peptide and glycan mass combinations, i.e., peptide proteoforms or the same sequence with different PTMs. If the precursor m/z is correctly determined, the true positive matches and false positive matches can be discriminated by their pMatchGlyco scores at MS2 level, as the scoring algorithm aims to find the best match based on the number of peak hits and their intensities, though a small set of false positive matches may exist due to the poor quality of glycopeptide MS/MS spectra. We therefore focus on the first kind of false positive matches because they are difficult to be corrected through improving the scoring algorithm. Since we already reversed the peptide sequence to generate the decoy library spectra for FDR control, the false positive matches remained after 1% FDR filtering will be caused by close glycan masses. When a suitable decoy glycan mass database is generated and applied for glycopeptide searching, matches to the decoy glycan masses can represent the extent of the fist type of false positive ones, as they don’t exist in the sample. Therefore, three ways of generating decoy mass database are compared here, they are (1) shifting the 739 N-glycan masses by 1 Da, (2) shifting the 739 N-glycan masses by 2 Da, and (3) shifting the 739 N-glycan masses with a number from 1.5, 2.5, 3.5, 4.5, 5.5, 6.5, 7.5, 8.5, 9.5, 10.5, 11.5, 12.5, 13.5 and 14.5 Da to ensure the smallest mass difference between any decoy mass and target mass is not less than 1 Da. The first two ways reflect mis-matches caused by incorrect monoisotopic peak assignment, but they are ineffective once the target and decoy matches once their masses are too close to be discriminated by precursor mass error tolerance. Therefore, we selected non-integral mass units in the third way and prevented the extremely close masses generated by the first two ways. Furthermore, mis-matches caused by variable modifications were prevented by limiting the maximal mass shift as 14.5 Da, since the smallest PTM mass except for deamidation is 14.016 Da, which is from peptide methylation. The reason of setting the smallest mass difference as 1 Da is because the mass error tolerance of precursor ion is set to 10 ppm., which means the 1 Da mass shift cannot be caused by instrument considering that most glycopeptides have their masses less than 10,000 Da. On the other side, there is no possible PTM that can cause a mass shift within 1 Da except for deamidation. Therefore, the only ambiguity between a target and a decoy mass match is due to deamidation on peptide backbone. This can be solved by comparing their pMatchGlyco scores. After searching the two datasets using a combined glycan mass database with both target masses and decoy masses, one can expect that the really existed glycan masses show better matches than the decoy glycan masses. As expected, the decoy glycan masses matches showed less GPSMs and lower pMatchGlyco scores comparing to those of the target glycan masses (**Figure 3**), suggesting that most of the targeted glycan masses identified by our method are truly existed in serum. Though a few target glycan masses showed low confidence based on the number of their GPSMs, i.e., they are less than 10 GPSMs, we can further validate these GPSMs manually.

*Setting of theta, relative intensities of added Y ions and minimal number of matched peptide ions*

In this work, higher-energy collisional dissociation (HCD) was used to generate MS/MS spectra of glycopeptides. Compared to peptide bond, glyosidic bond has a lower energy and is prior to be fragmented under HCD. Therefore, intensities of oxonium ions are typically higher than those of peptide ions. Taking UGP dataset as an example, more than 80% spectra contained at least one oxonium ion, and the summed intensity of 25 oxonium ions accounted for at least 50% of the total peak intensity in 66.7% spectra (**Supplementary Figure 12**). In our previous work, a strategy for library spectra processing was proposed and validated in two published datasets from recombinant protein mixture and OVCAR3 cell lysate respectively (2). It removes the 25 oxonium ions from glycopeptide spectra and add N-glycan core related Y ions into library spectra. Here, the same strategy was applied in the human serum datasets, while several parameters were adjusted considering the difference of sample types and normalized collision energy (NCE) used for MS fragmentation. To investigate the influence of oxonium ions in identifying glycopeptides, we used UDGP, UGP and the previously published OVCAR3 cell line dataset to compare the number of GPSMs between deletion and remaining of the 25 oxonium ions in query spectra. It showed that deletion of 25 oxonium ions from query spectra resulted in 90% increase of glycopeptide-spectrum matches (GPSMs) in UGP dataset and 50% increase of GPSMs in OVCAR3 dataset, compared to the GPSMs obtained by remaining of 25 oxonium ions. This result suggested that intense oxonium ions impeded identification of N-linked glycopeptides. Hence, it is feasible to delete 25 oxonium ions from query spectra regardless of sample type or MS fragmentation energy.

As mentioned above, insufficient fragmentation of peptide backbone often leads to absence of peptide ions on glycopeptide spectrum. Compared to its de-glycosylated peptide, less peptide ions could be observed from a glycopeptide. Therefore, only including those peptide ions observed on de-glycosylated peptide spectrum, which is used for spectral library construction, is assumed to cover all peptide ions that will be observed on glycopeptide spectrum. To test this hypothesis, a parameter theta that was inherited from pMatch was adjusted in pMatchGlyco, enabling addition of absent peptide ions into library spectra with customized relative intensities. It showed that the most GPSMs was achieved by setting theta as 0, supporting that all peptide ions observed on de-glycosylated peptide spectrum were enough for glycopeptide inferring. A threshold of minimal number of matched peptide ions was also introduced to filter out random matches. It showed that the most GPSMs was achieved by setting the threshold of minimal number of peptide ion matches as three in UGP or four in FGP (**Supplementary Figure 13**). Notably, different collision energy settings lead to heterogeneity of glycopeptide fragmentation. Since we used different collision energy setting when obtaining UGP (NCE 27) and FGP (NCE 33) (see **Experimental Procedures**), this might explain the suitable threshold of minimal number of matched peptide ions were different between them.

On the other side, Y ions were added into library spectra to increase the matched peaks between library spectrum and query spectrum. The number as well as relative intensities of added Y ions were fine-adjusted, and it showed that addition of all six Y ions with relative intensity of 0.2 to library spectra achieved the most GPSMs. The optimal setting of theta as 0, relative intensities of Y ions as 0.2 and the minimal number of matched peptide ions as 3 or 4 were thus applied in the two datasets.

1. He, L., Diedrich, J., Chu, Y. Y., and Yates, J. R., 3rd (2015) Extracting Accurate Precursor Information for Tandem Mass Spectra by RawConverter. *Anal Chem* 87, 11361-11367

2. An, Z. W., Shu, Q. B., Lv, H., Shu, L., Wang, J. F., Yang, F. Q., and Fu, Y. (2018) N-Linked Glycopeptide Identification Based on Open Mass Spectral Library Search. *Biomed Res Int*
