# Supplementary material for: Large-scale Identification of N-linked Intact Glycopeptides in Human Serum using HILIC Enrichment and Spectral Library Search
Source: Mol Cell Proteomics. 2020 Feb 26;19(4):672–89. doi: 10.1074/mcp.RA119.001791 (PMC7124471; doi:10.1074/mcp.RA119.001791)
Supplement: Supplementary Document 1 [file 156056_1_supp_471918_q5c98v.pptx]

## Slide 1
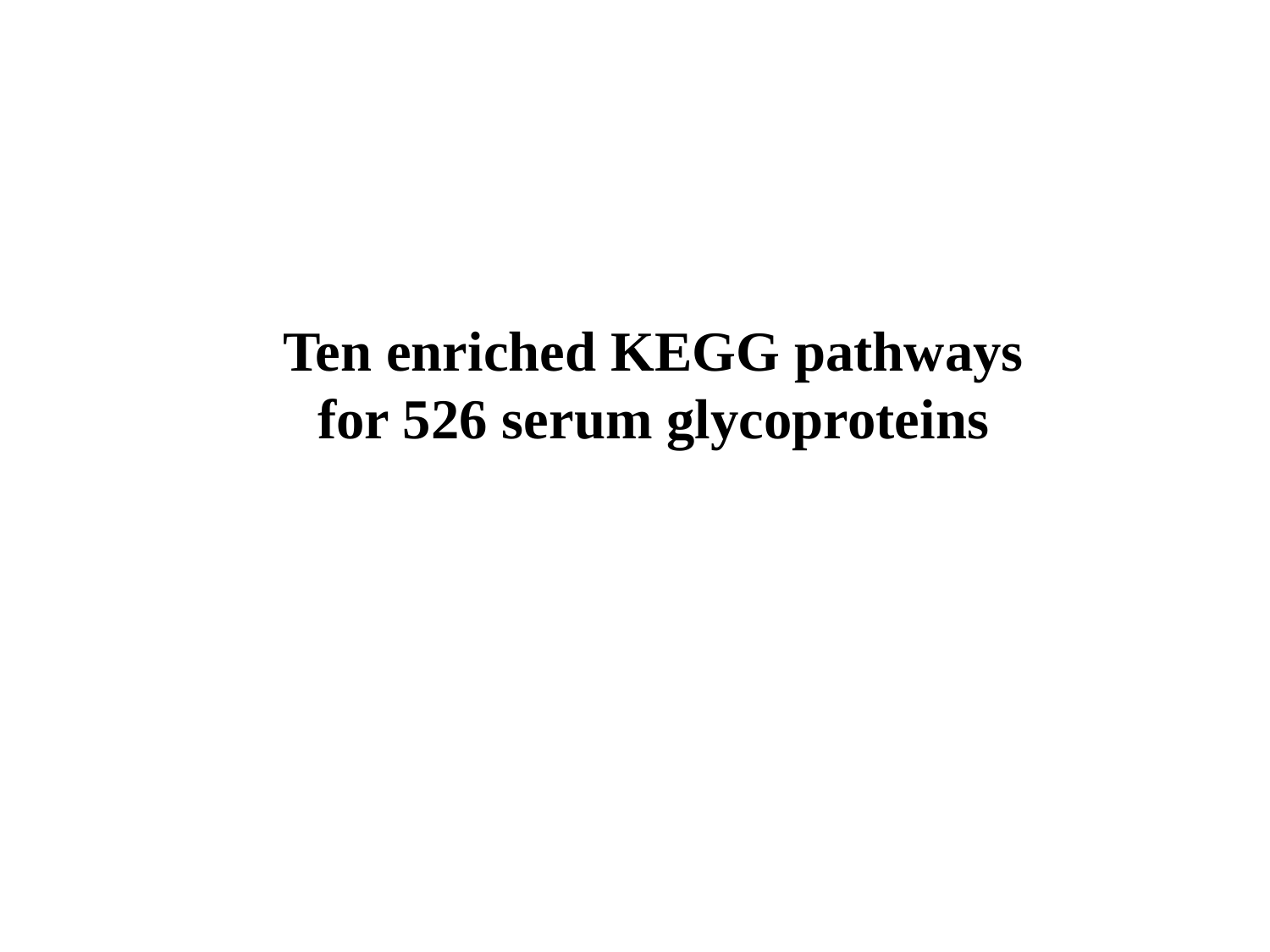

Ten enriched KEGG pathways for 526 serum glycoproteins

## Slide 2
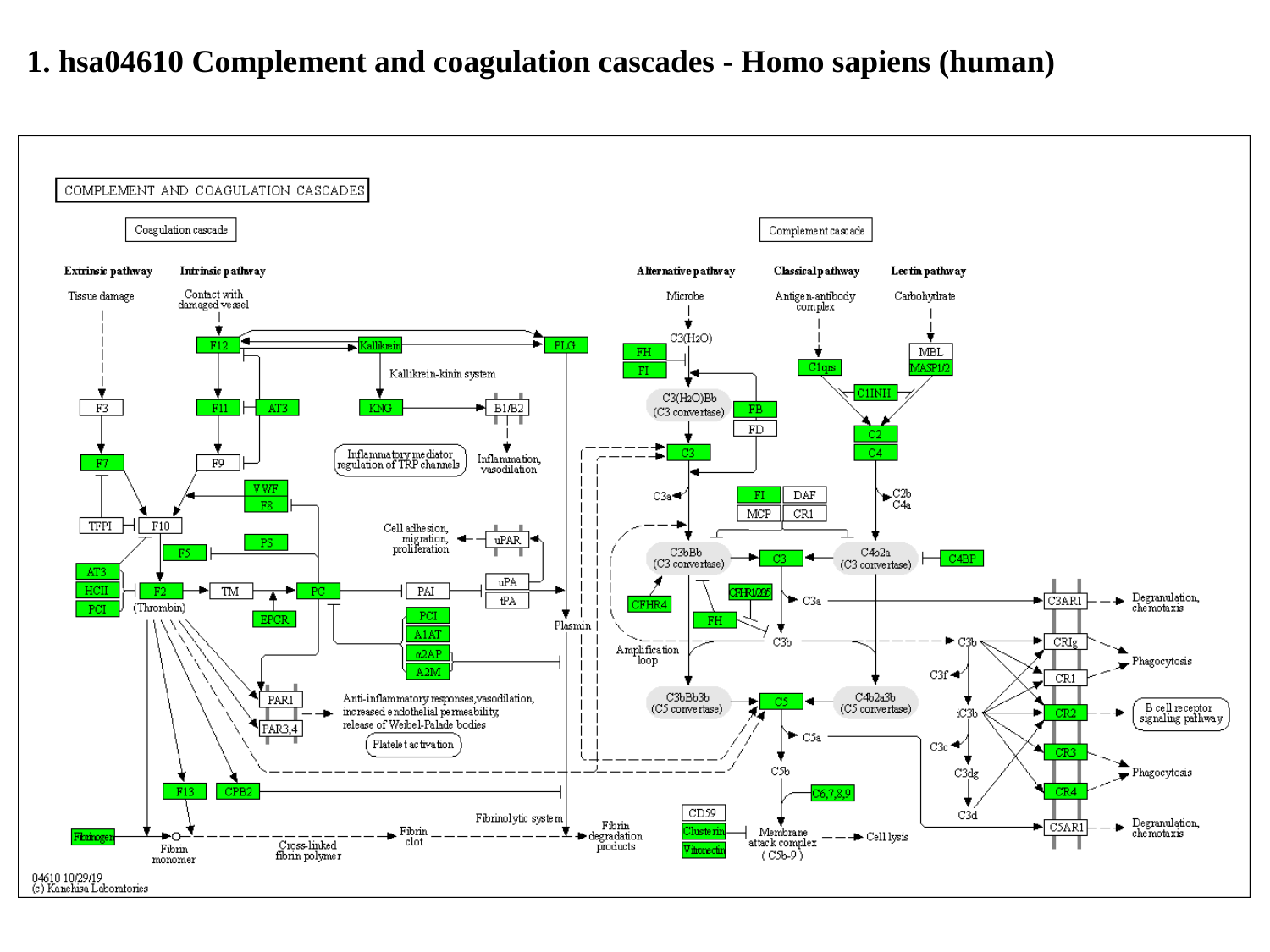

1. hsa04610 Complement and coagulation cascades - Homo sapiens (human)

## Slide 3
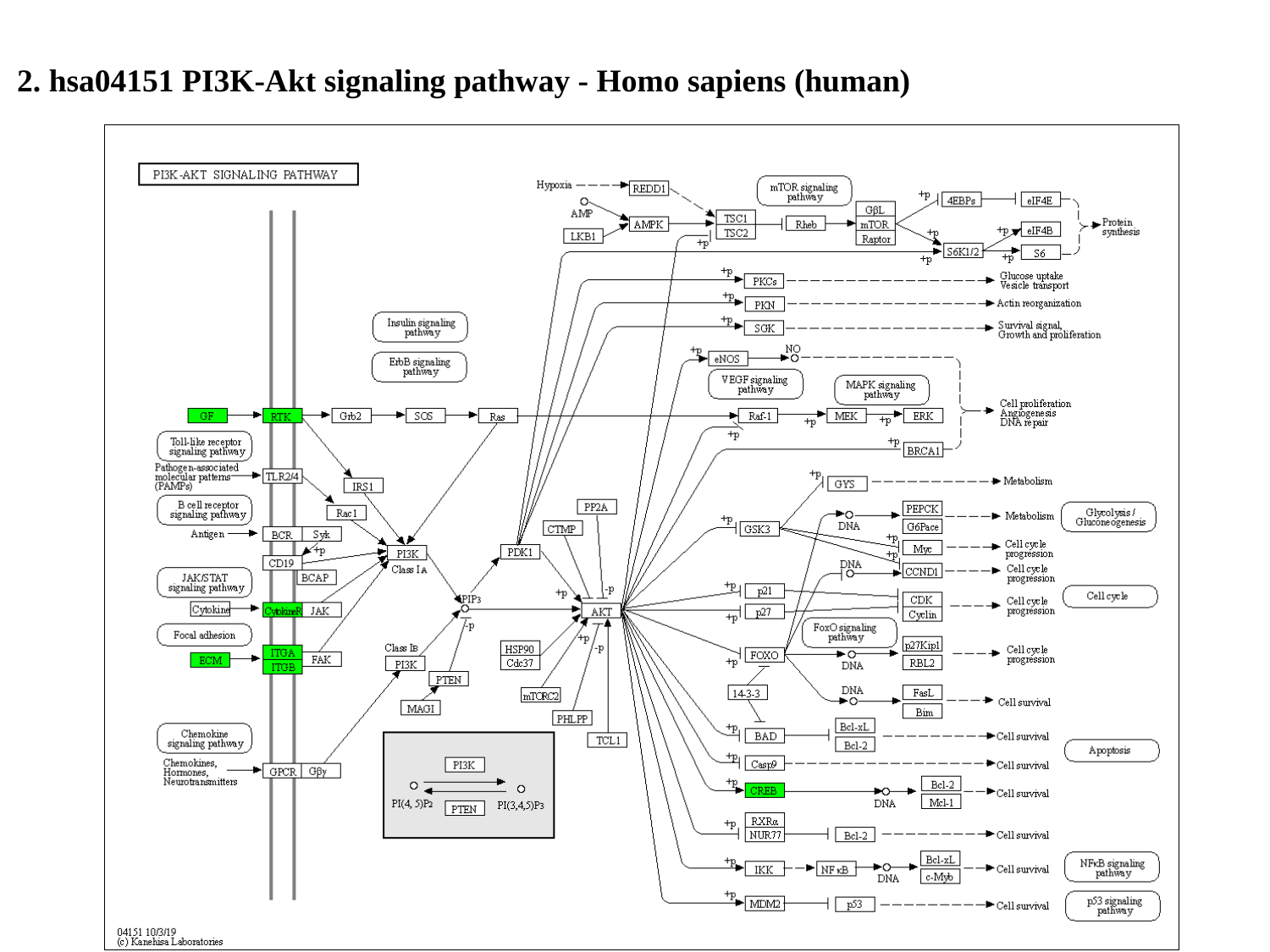

2. hsa04151 PI3K-Akt signaling pathway - Homo sapiens (human)

## Slide 4
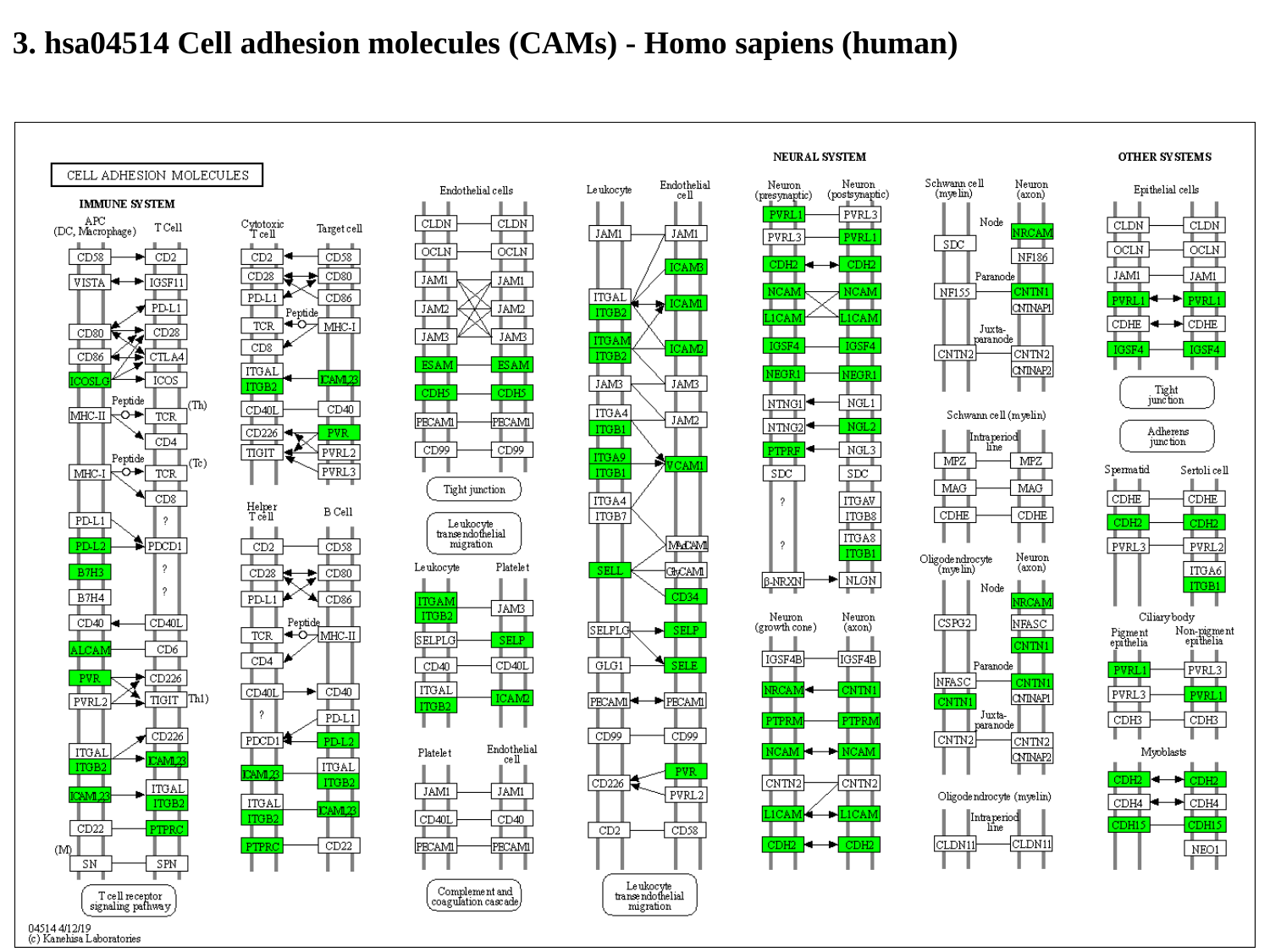

3. hsa04514 Cell adhesion molecules (CAMs) - Homo sapiens (human)

## Slide 5
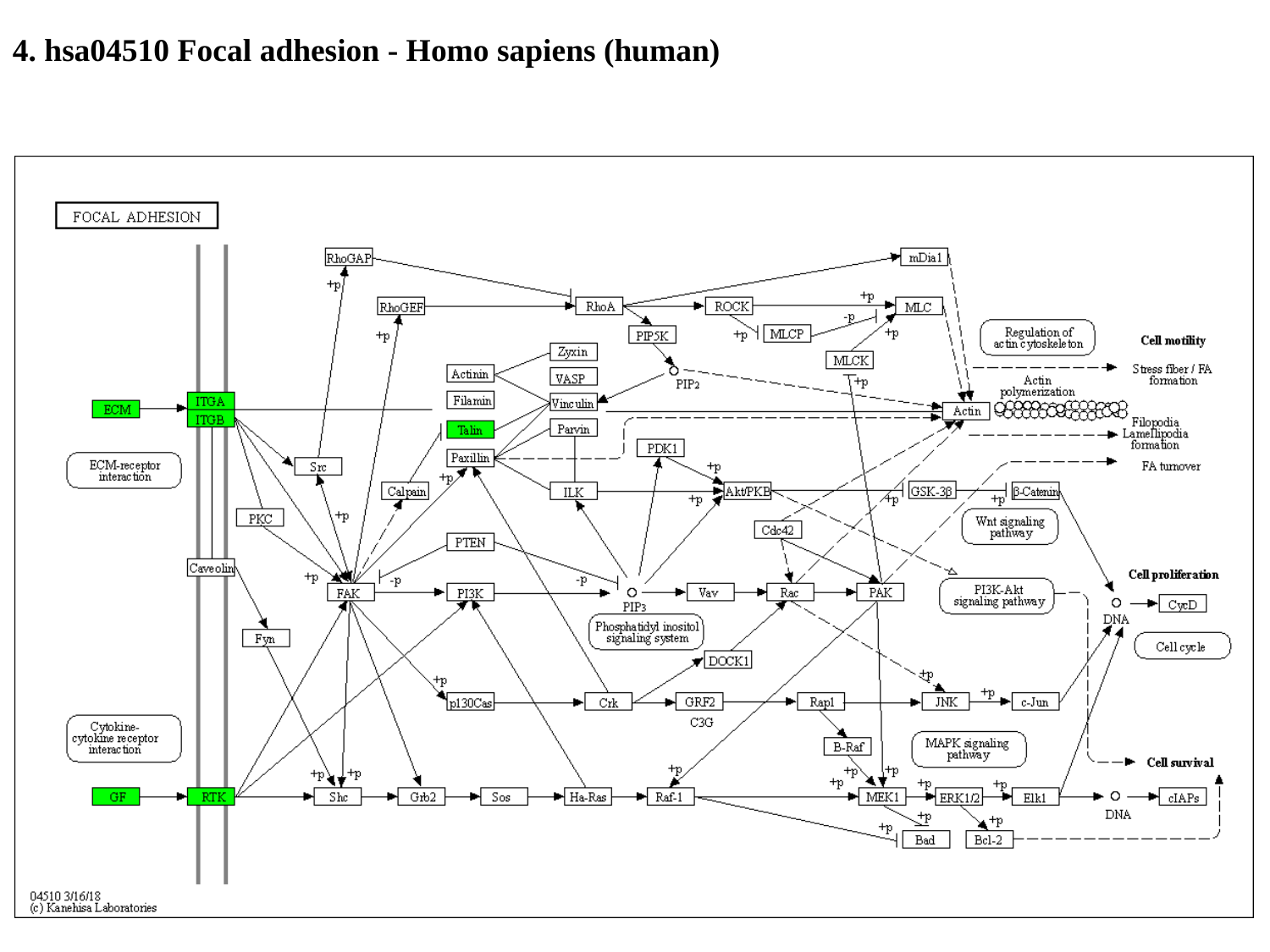

4. hsa04510 Focal adhesion - Homo sapiens (human)

## Slide 6
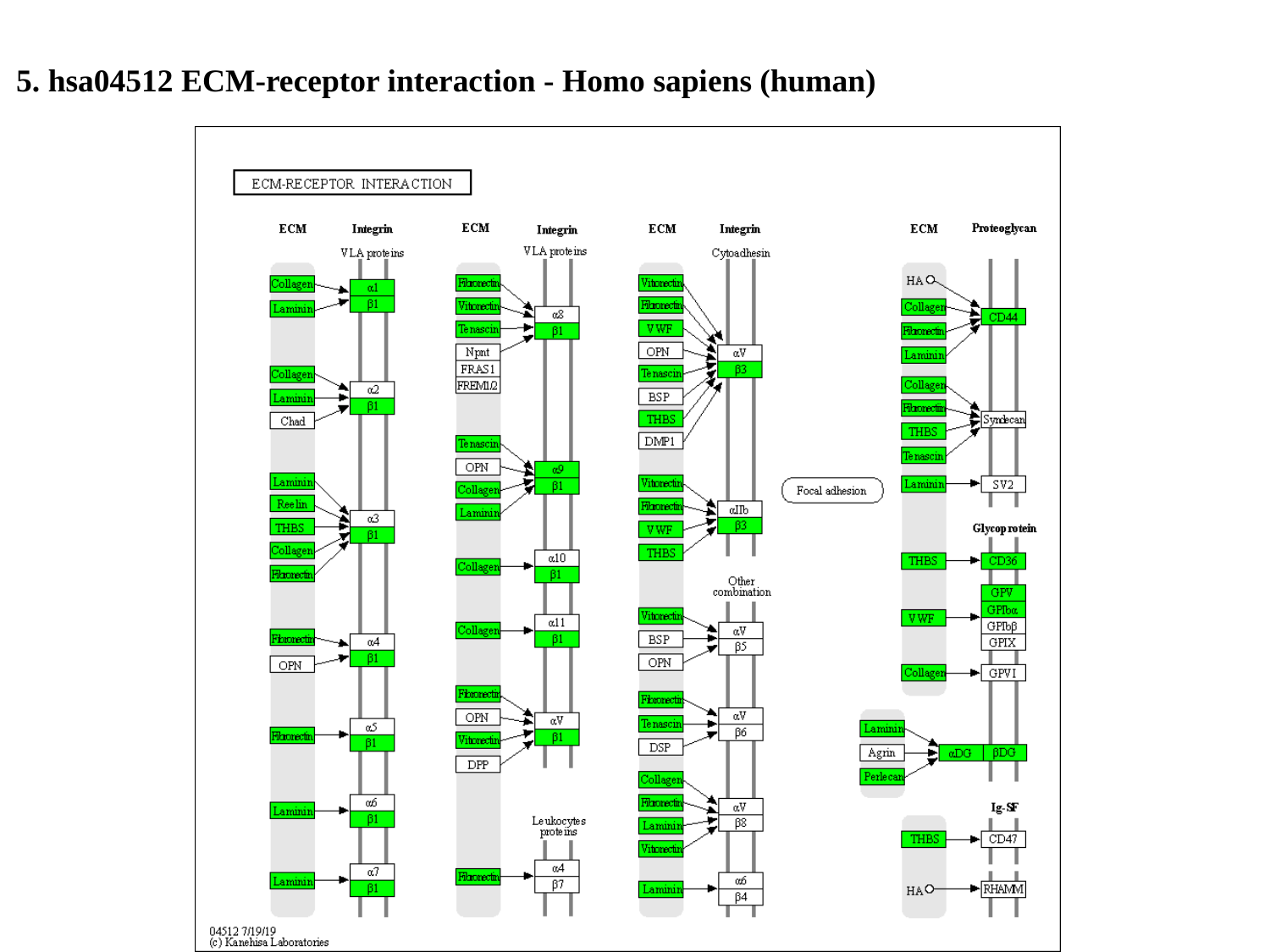

5. hsa04512 ECM-receptor interaction - Homo sapiens (human)

## Slide 7
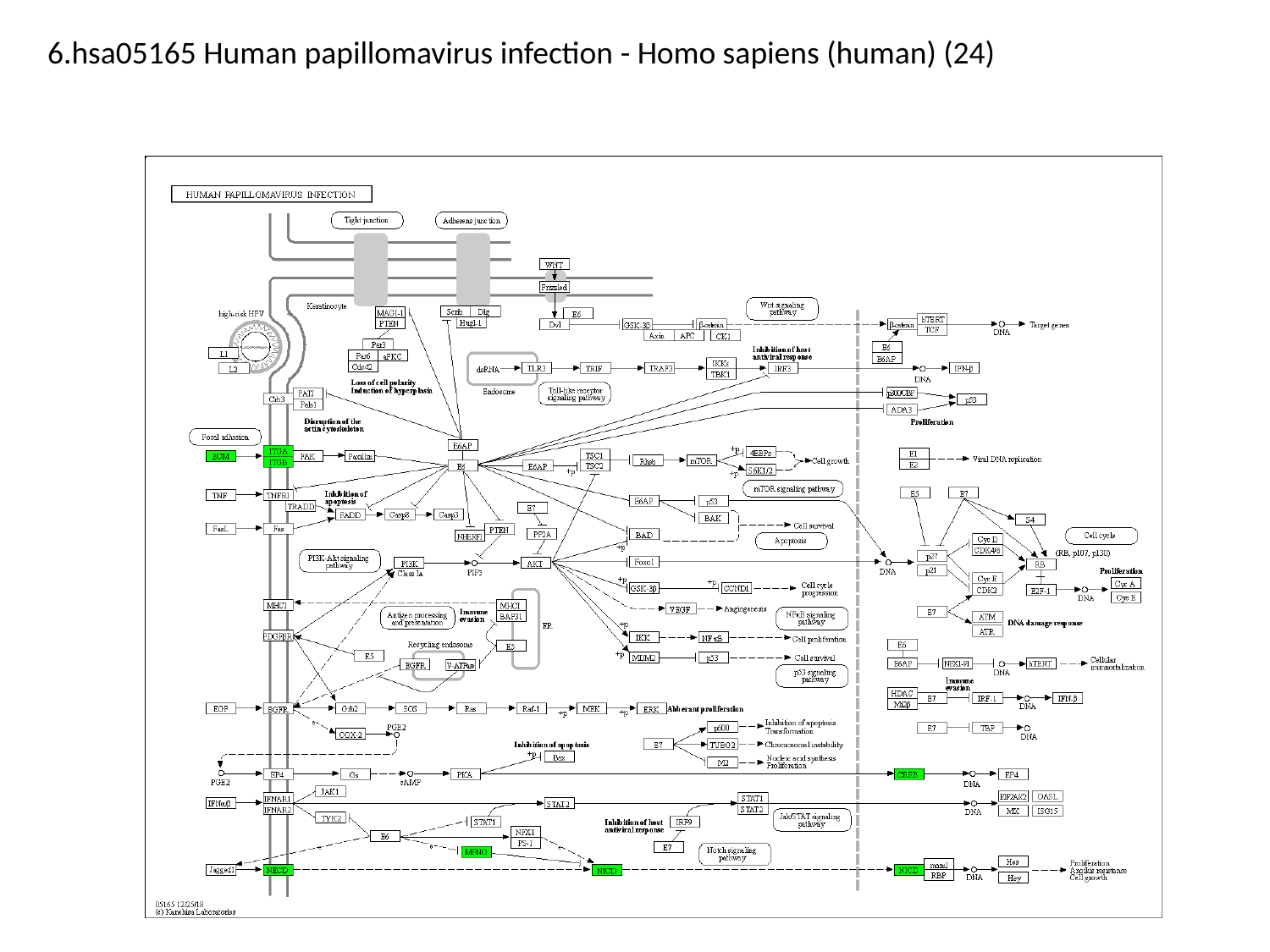

6.hsa05165 Human papillomavirus infection - Homo sapiens (human) (24)

## Slide 8
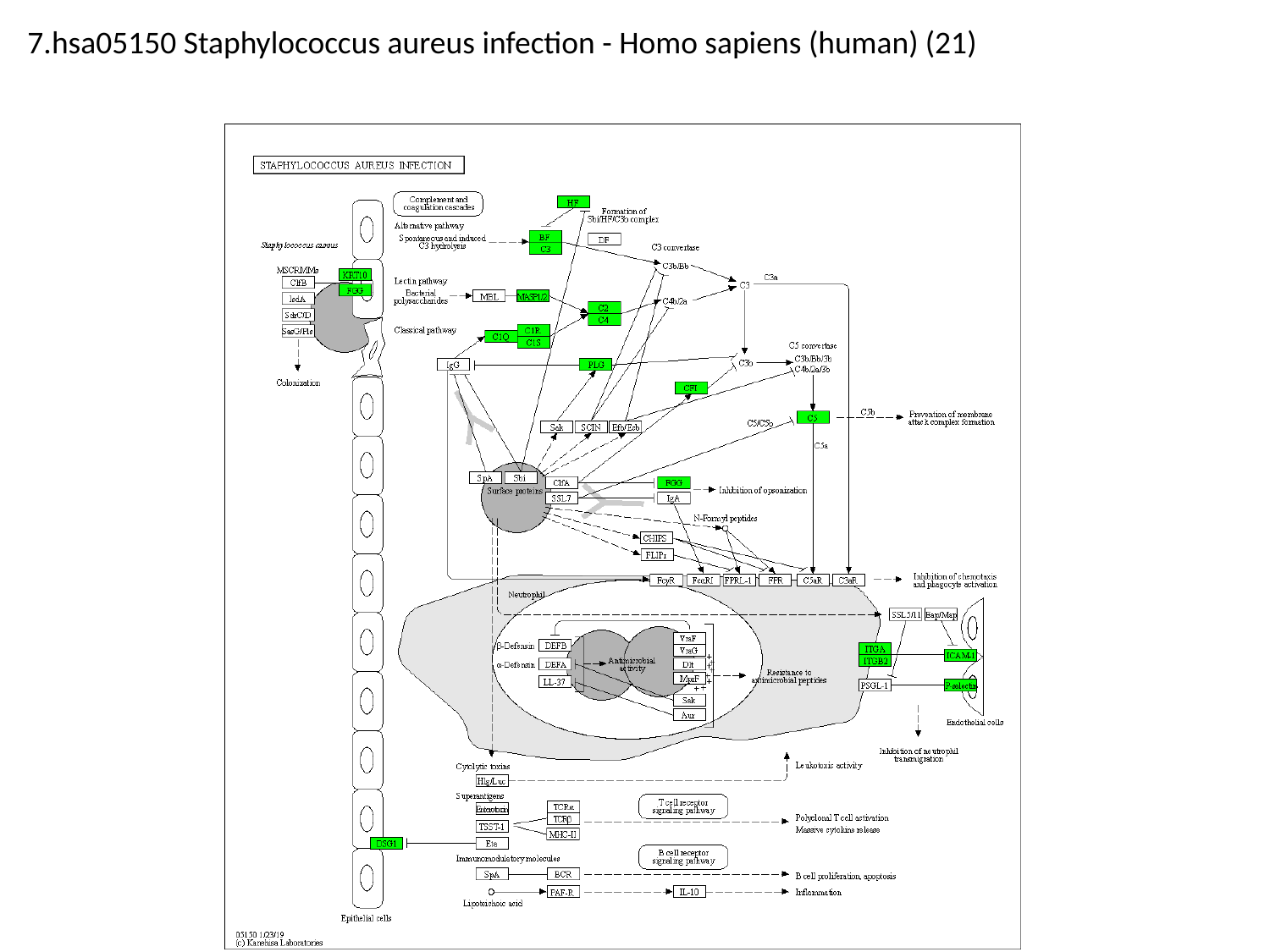

7.hsa05150 Staphylococcus aureus infection - Homo sapiens (human) (21)

## Slide 9
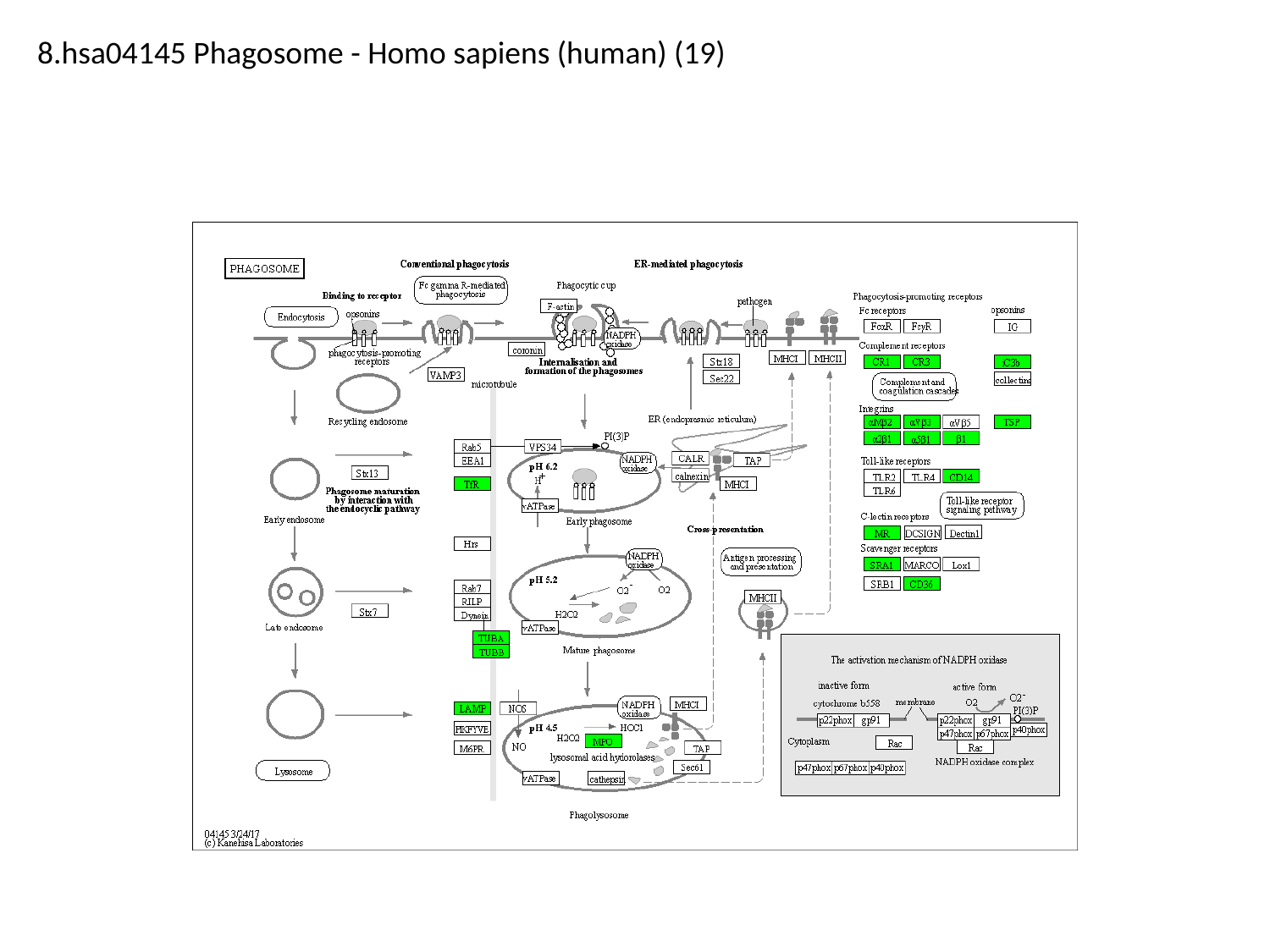

8.hsa04145 Phagosome - Homo sapiens (human) (19)

## Slide 10
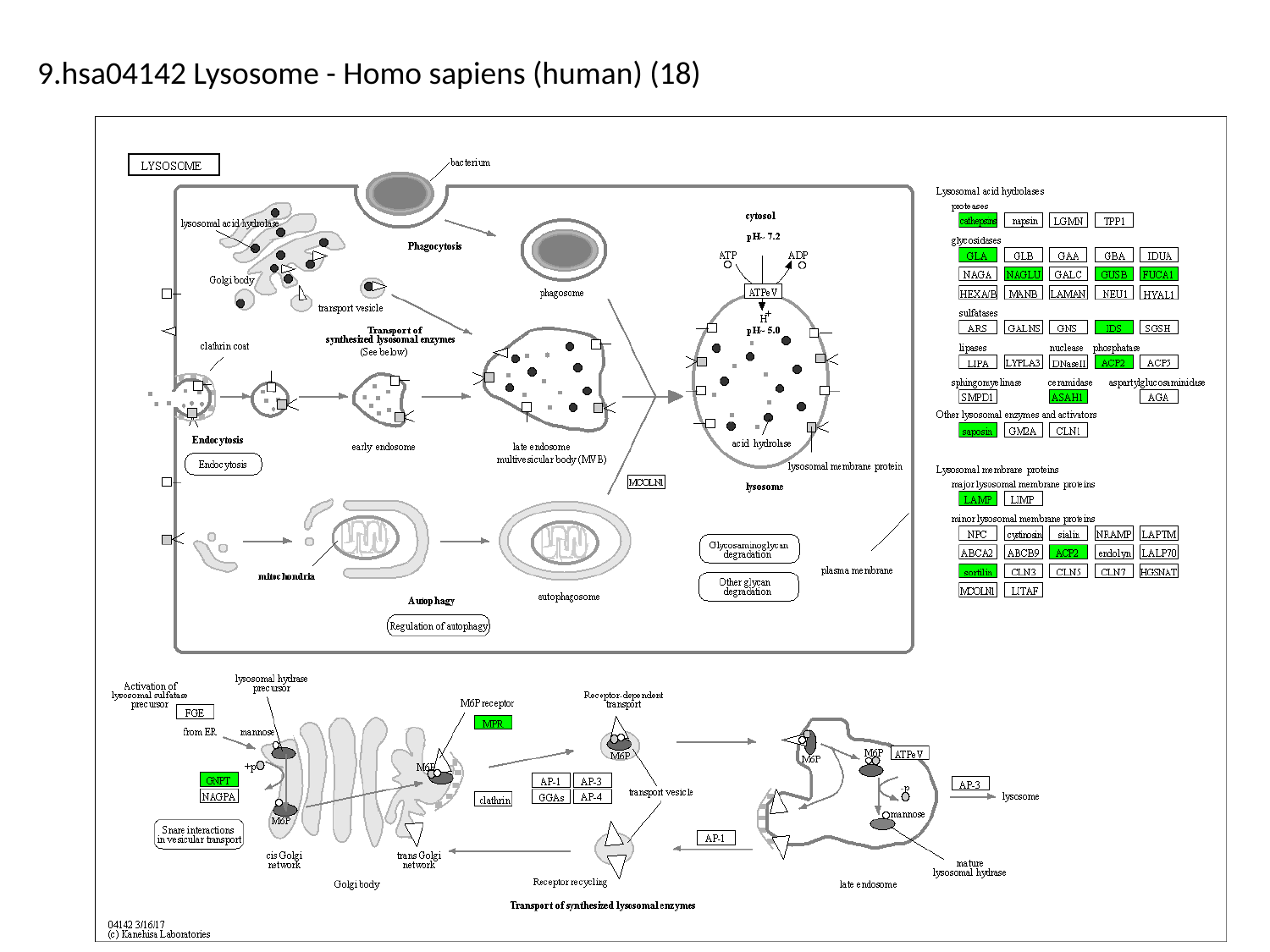

9.hsa04142 Lysosome - Homo sapiens (human) (18)

## Slide 11
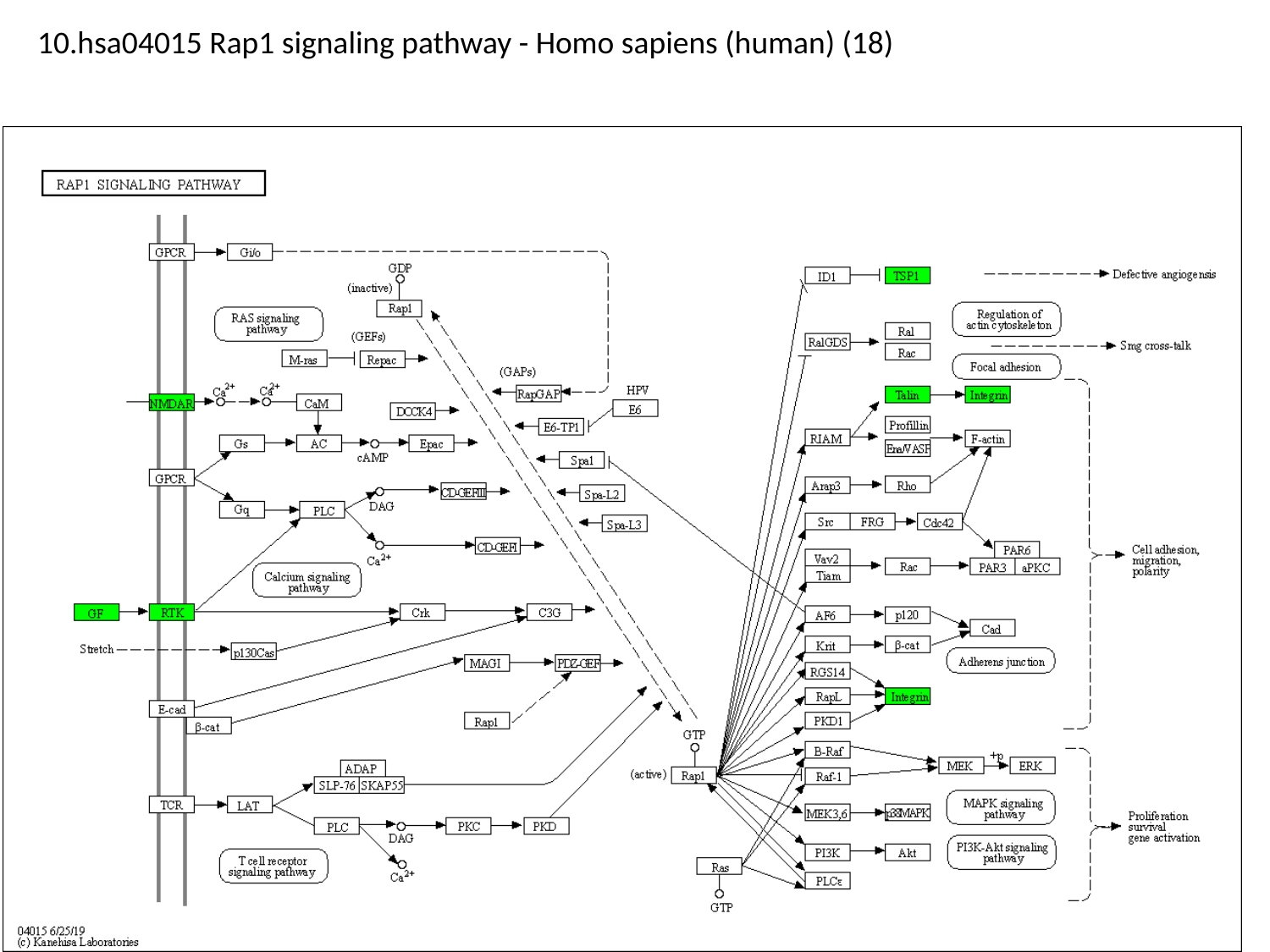

10.hsa04015 Rap1 signaling pathway - Homo sapiens (human) (18)
