# Supplementary material for: Large-scale Identification of N-linked Intact Glycopeptides in Human Serum using HILIC Enrichment and Spectral Library Search
Source: Mol Cell Proteomics. 2020 Feb 26;19(4):672–89. doi: 10.1074/mcp.RA119.001791 (PMC7124471; doi:10.1074/mcp.RA119.001791)

**Supplementary Figure Legends**

**Supplementary Figure 1**. 16 kinds of protein PTMs identified in the serum datasets.

**Supplementary Figure 2**. The complex of human sero-transferrin binding to the pathogenic Neisseria TbpA protein. Its three-dimensional structure (PDB entry 3v8X) is presented with its surface (a, b) and ribbon model (c, d) rendered by PyMOL. Sero-transferrin is colored cyan and TbpA is colored green. The oligosaccharide chains linked to sero-transferrin are colored red and shown as sticks, and the key amino acid residues for iron ion binding, i.e, Arg456, Ala458, Gly459 and Try517 are colored magenta in (a) and (c). In (d), the two canonical and occupied N-glycosites, i.e., N432 and N630 are present as sticks and colored yellow. The uncanonical N-glycosites, i.e., N523 and N637 are present as sticks and colored magenta.

**Supplementary Figure 3**. An incorrect GPSM caused by inaccurate determination of precursor m/z. The precursor m/z of spectrum with scan title UGP_02.20637.20637.3.0.dta in UGP dataset was initially determined as 1207.813 (M1, red arrowhead), and was corrected to 1207.4894 (M0, green arrowhead) after manual check.

**Supplementary Figure 4**. The dimer of human sero-transferrin binding to human transferrin receptor 1. Its three-dimensional structure (PDB entry 1SUV) is presented with its surface (a) and ribbon model (b) rendered by PyMOL. The C-lobes (colored orange and green) and the N-lobes (colored magenta and yellow) of sero-transferrin, and transferrin receptor 1 (gray) is presented in two perpendicular directions. The two N-glycosite of sero-transferrin, i.e., N432 and N630 are colored blue in the left C-lobe and colored red in the right C-lobe. The two identified N-glycosites N251 and N727 of transferrin receptor 1 are colored cyan.

**Supplementary Figure 5**. Four mass spectra from four LC-MS/MS runs identified as the same glycopeptide GLTFQQNASSMCVPDQDTAIR with glycan Hex5HexNAc5Fuc1. Intensive oxonium ion peaks are colored blue. Peptide ions are colored orange (y ions) and green (b ions), and Y ions are colored purple.

**Supplementary Figure 6**. Shift of retention time of one glycopeptide in replicate LC runs and idopt cutoff setting. The retention time (panel a) and idotp value (panel b) of the precursor m/z 1519.95 in Figure 7a are presented in four LC runs in UGP.

**Supplementary Figure 7.** Comparison of GPSMs, glycoproteins, glycosites and N-Glycans obtained using four sets of searching parameters in UGP and FGP datasets. Pink ellipse represents tryptic digestion with three prevalent variable PTMs; Purple ellipse represents semi-tryptic digestion with three prevalent variable PTMs; blue ellipse represents tryptic digestion with the specified 16 variable PTMs; yellow ellipse represents semi-tryptic digestion with the specified 16 variable PTMs.

**Supplementary Figure 8.** Difference of the most intense isotopic peak in glycopeptide and its de-glycosylated counterparts. (a) Distribution of M0-M5 as the most intense isotopic peak in glycosite-containing peptides and glycopeptides. (b) Relationship of M+H of precursor ion and their most intense isotopic peaks in de-glycopeptide (DG, colored orange) and glycosylated peptide (GP, colored cyan).

**Supplementary Figure 9.** Co-elution of glycopeptide precursor ions on MS1 leads to mixed spectra that complicates determination of precursor m/z. (a) Precursor m/z of two query MS/MS spectra recorded by the instrument on their most recent MS1 spectrum. The two peaks are labeled with their recorded m/z and colored magenta and blue, respectively. (b) Elution profiles of the two precursors’ isotopic peaks. (c, d) GPSM results of the two query MS/MS spectra by activating co-elution in pParse. (e, f) Matching of intense peaks on the two query spectra according to the GPSM results.

**Supplementary Figure 10.** Scan number 11178 in UGP Run_01 identified as two GPSMs. (a) Identities of the two GPSMs are presented. Hex, hexose including mannose and galactose. Fuc, fucose. HexNAc, N-acetylglucosamine. NANA, N-acetylneuraminic acid. The glycosites are colored green. (b) Matched peaks are labeled with their m/z values, fragments of GPSM1 are colored blue, and fragments of GPSM2 are colored purple.

**Supplementary Figure 11.** A case of co-eluted precursor ions identified as two glycopeptides with the same peptide backbone but different glycans. (a) Isotopic peaks of the two precursor ions on the most recent MS1 spectrum. (b) Ion matches were shown on the MS/MS spectrum. (c) Identities of the two GPSMs with their glycans and formula presented. Hex, hexose including mannose and galactose. Fuc, fucose. HexNAc, N-acetylglucosamine. NANA, N-acetylneuraminic acid. The glycosite was labeled with underline. (d, e) Theoretical isotopic distributions of the two precursor ions. M+H was depicted.

**Supplementary Figure 12**. Comparison of different spectra processing strategies by deletion/remaining of 25 oxonium ions from query spectra, as well as addition of distinct subset of Y ions into library spectra generated from UDGP. (a) Schematic presenting of six N-glycan core related Y ions. (b) Distribution of the summed relative intensity of 25 oxonium ions among spectra in UGP, data was shown in mean+SD (n=4 replicate runs). (c) UGP dataset result. (d) OVCAR-3 dataset result.

**Supplementary Figure 13.** Comparison of GPSM identities in UGP and FGP by adjusting (a) the value of parameter theta, (b) the threshold number of peptide ions matched and (c) the relative intensities of added Y ions during library construction. (d) The optimal values of these parameters in the two datasets are presented.

**Supplementary Figure 1**

**
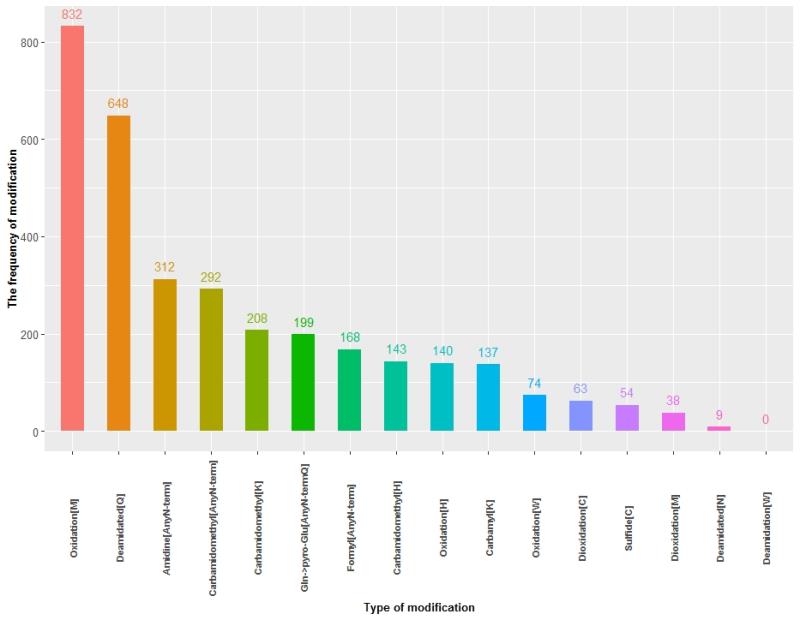
**

**Supplementary Figure 2**


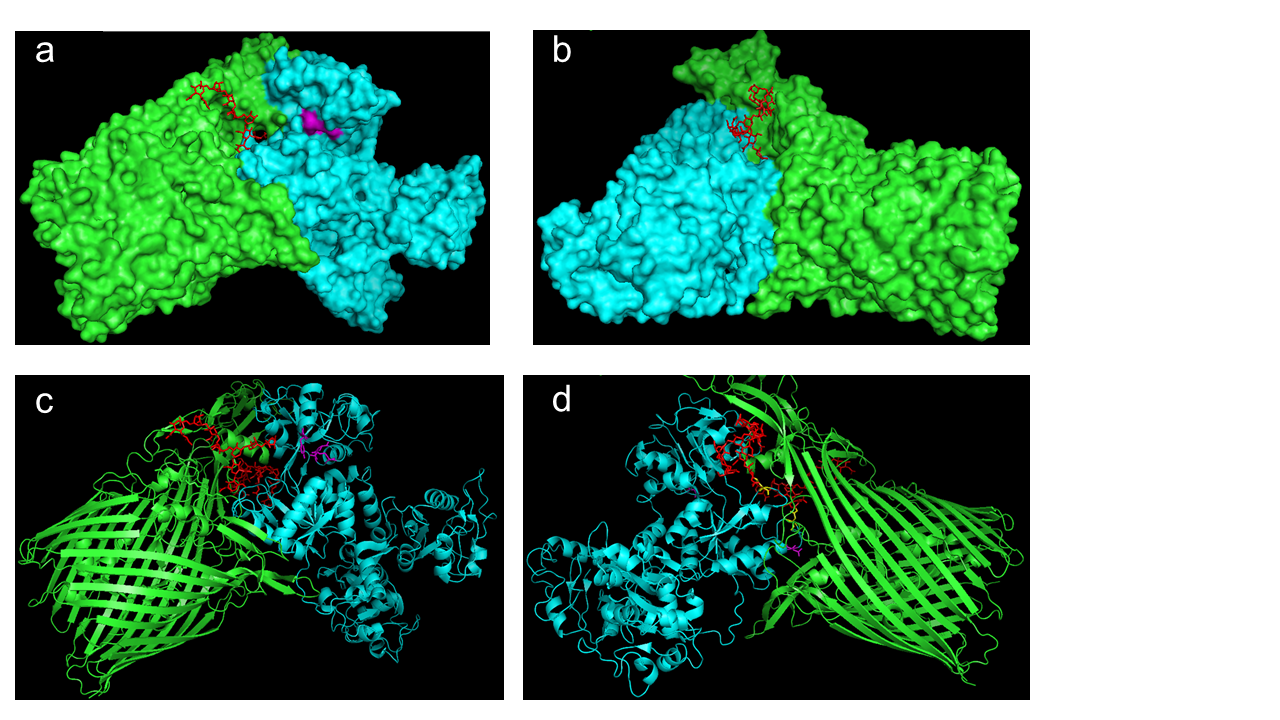


**Supplementary Figure 3**

**Supplementary Figure 4**


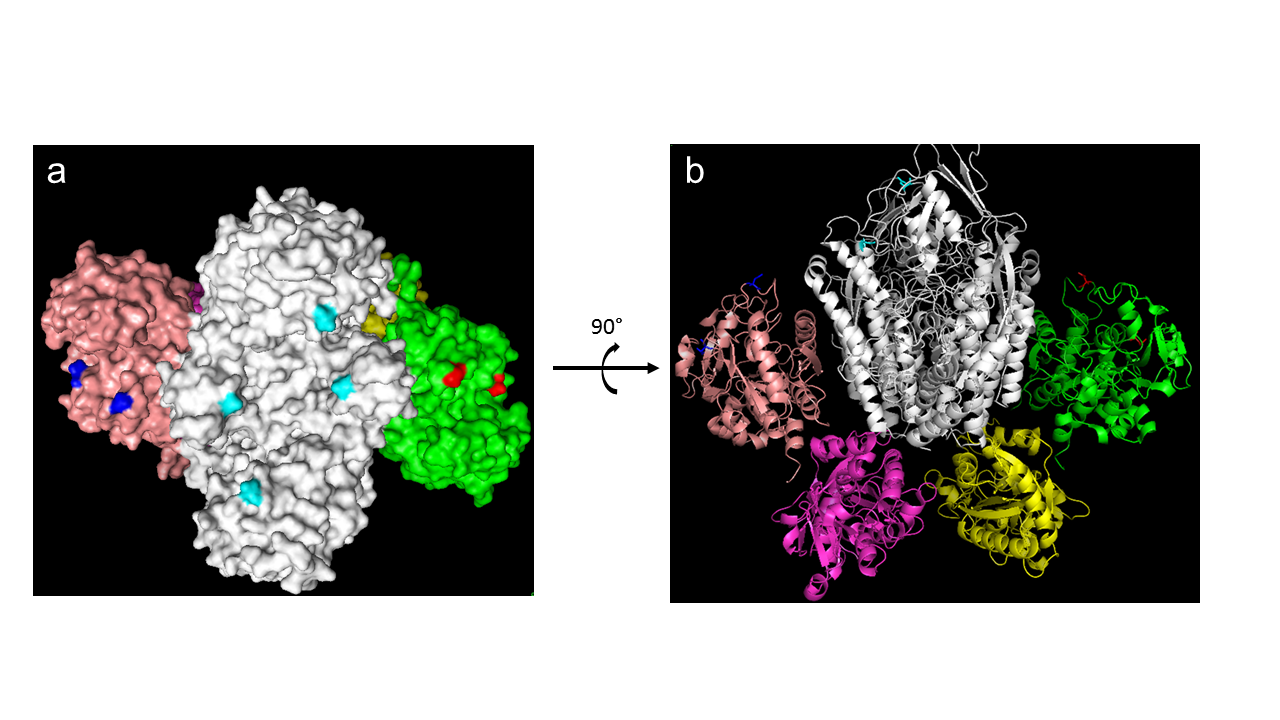


**Supplementary Figure 5**


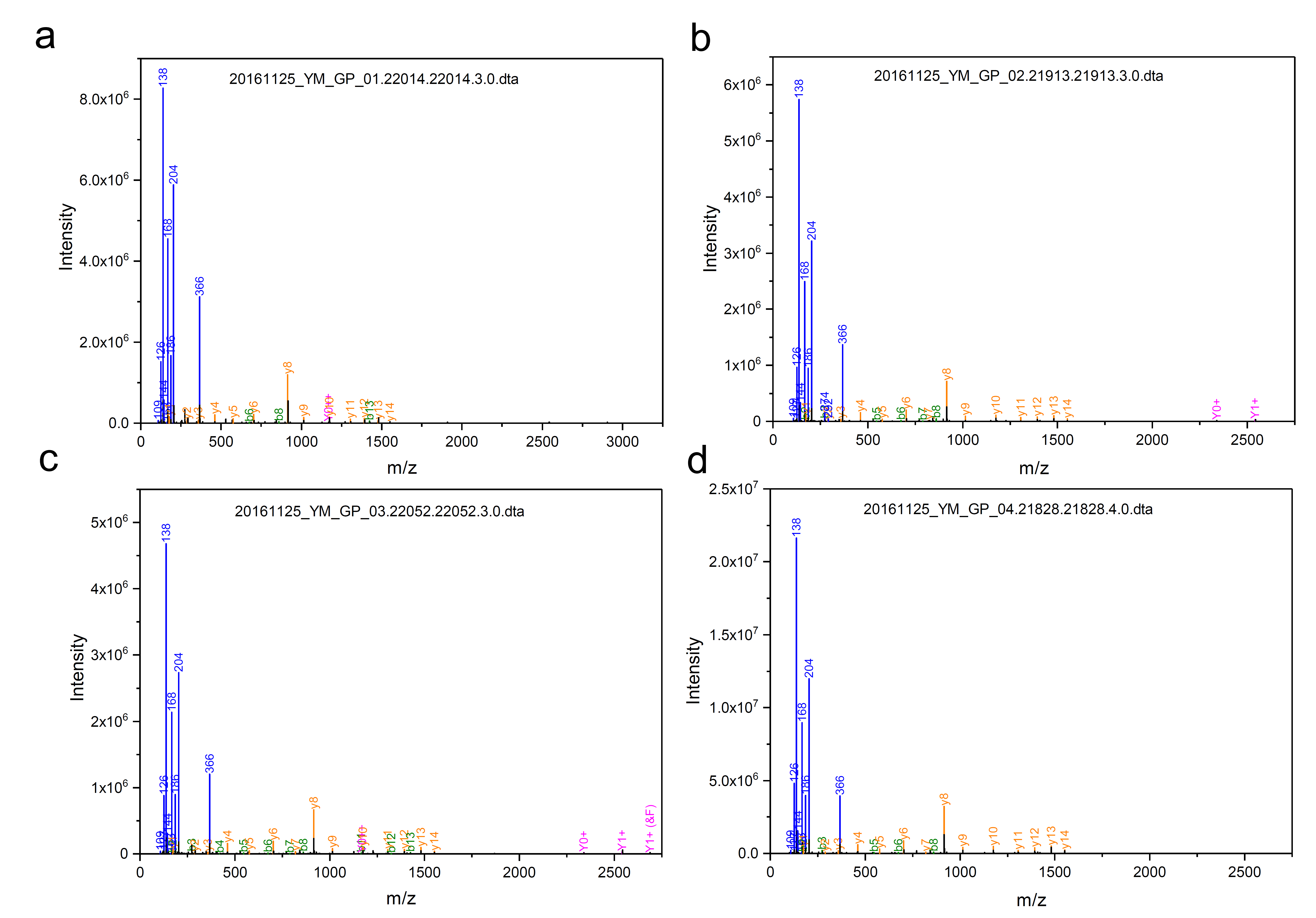


UGP_04, scan #21828

UGP_03, scan #22052

UGP_02, scan #21913

UGP_01, scan #22014

**Supplementary Figure 6**


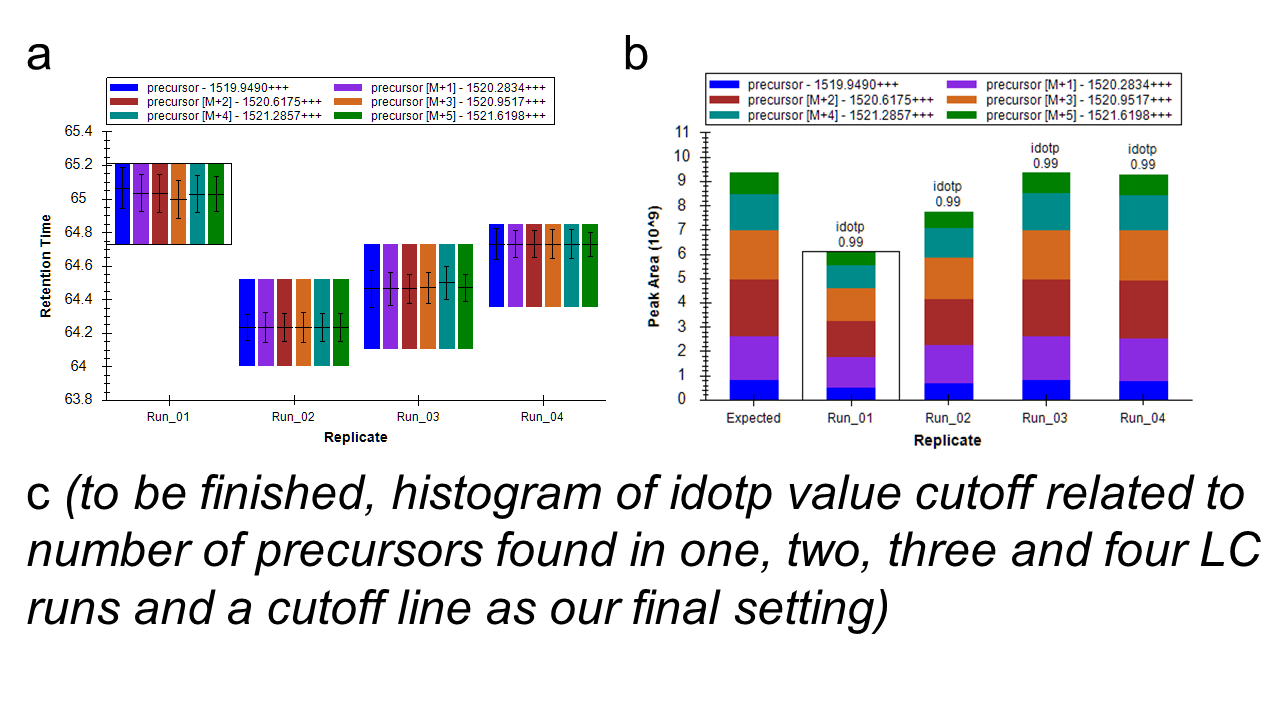
 **Supplementary Figure 7**


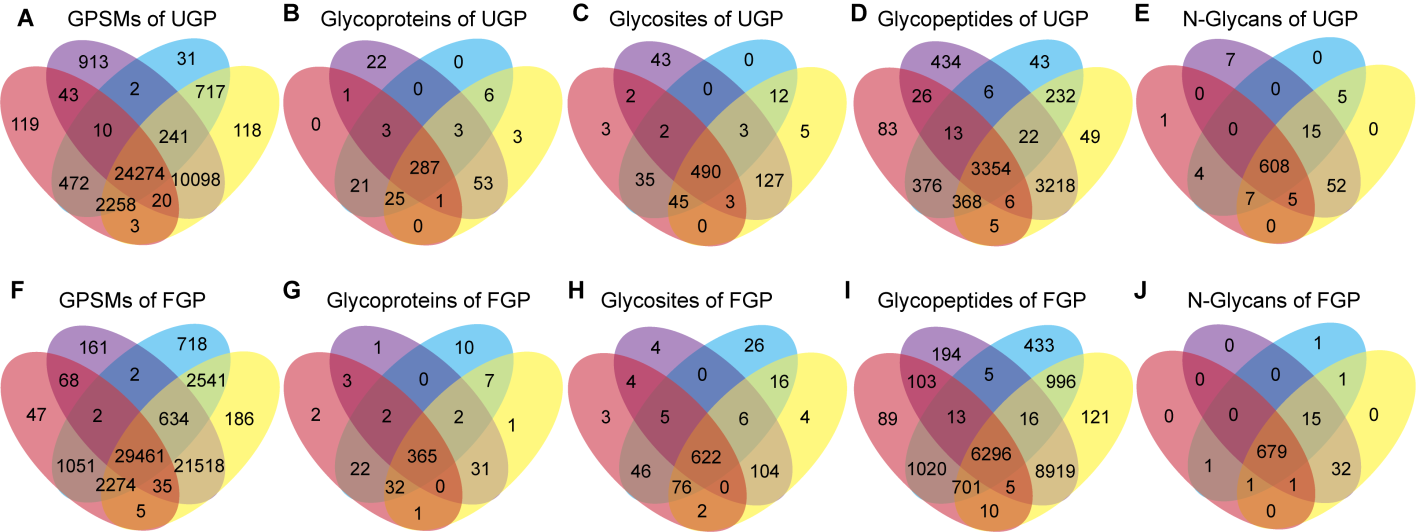


**Supplementary Figure 8**


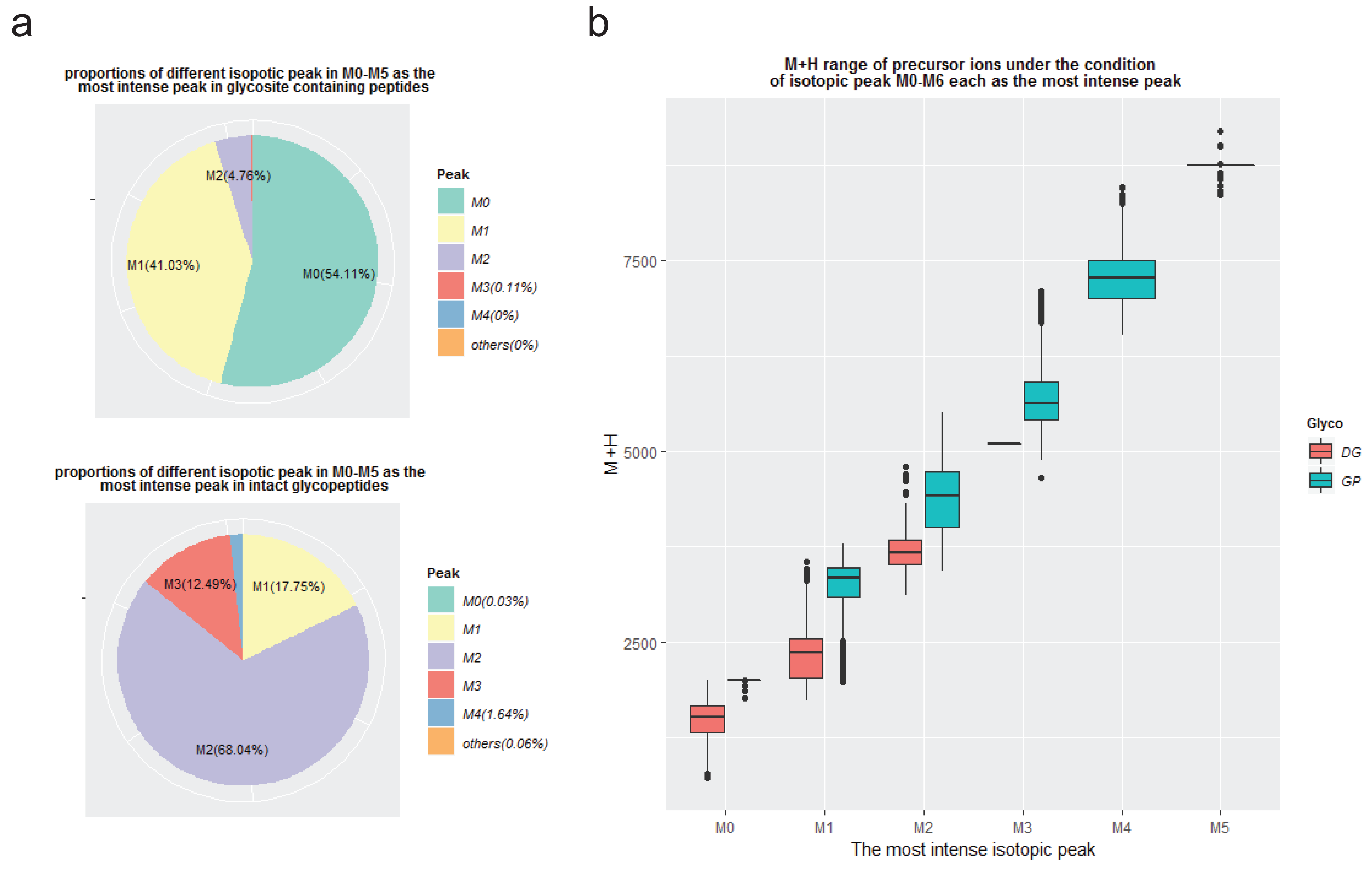


**Supplementary Figure 9**


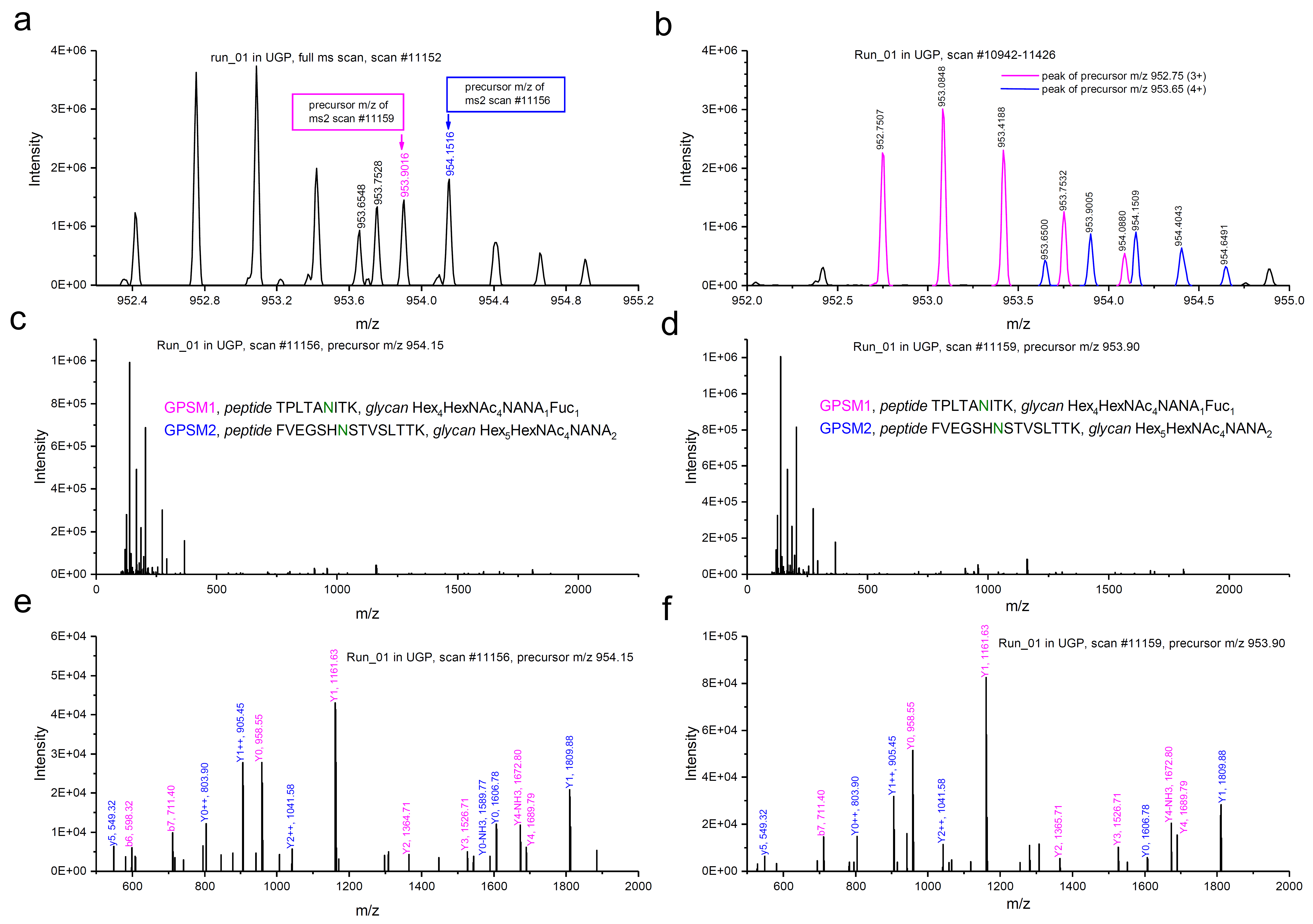


**Supplementary Figure 10**





**Supplementary Figure 11**





**Supplementary Figure 12**





**Supplementary Figure 13**


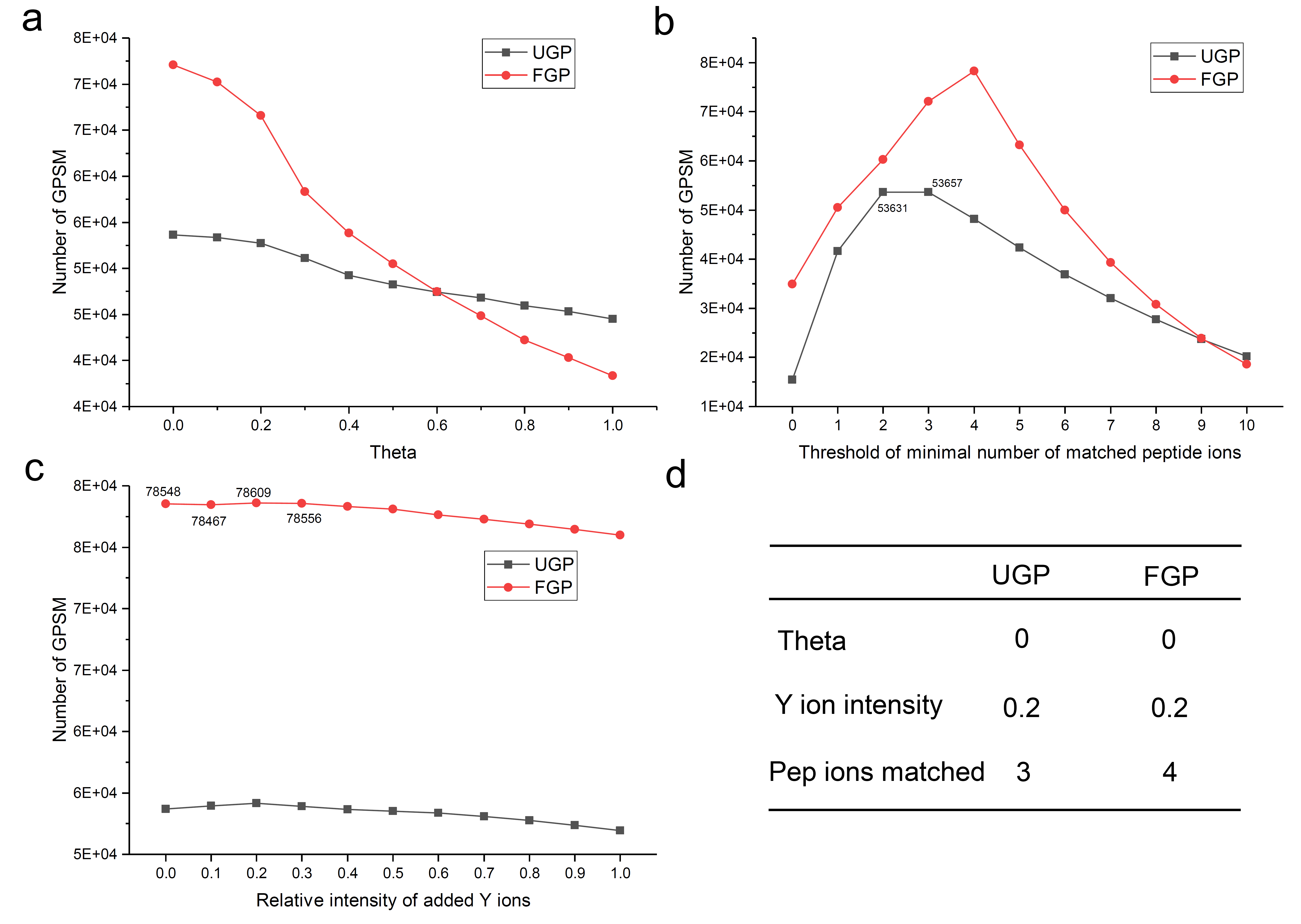

Supplement: Supplementary Figures [file 156056_1_supp_471921_q5c98v.docx]
