# Supplementary material for: Large-scale Identification of N-linked Intact Glycopeptides in Human Serum using HILIC Enrichment and Spectral Library Search
Source: Mol Cell Proteomics. 2020 Feb 26;19(4):672–89. doi: 10.1074/mcp.RA119.001791 (PMC7124471; doi:10.1074/mcp.RA119.001791)
Supplement: Supplementary Document 2 [file 156056_1_supp_471970_q5hz4b.pdf]

reactome

Pathway Analysis Report

This report contains the pathway analysis results for the submitted sample ''. Analysis was performed against Reactome version 71 on 08/02/2020. The web link to these results is:

https://reactome.org/PathwayBrowser/#/ANALYSIS=MjAyMDAyMDgxODM0MzJfMjQ4NjA%3D

Please keep in mind that analysis results are temporarily stored on our server. The storage period depends on usage of the service but is at least 7 days. As a result, please note that this URL is only valid for a limited time period and it might have expired.

Table of Contents

1. Introduction
2. Properties
3. Genome-wide overview
4. Most significant pathways
5. Pathways details
6. Identifiers found
7. Identifiers not found

1. Introduction

Reactome is a curated database of pathways and reactions in human biology. Reactions can be considered as pathway 'steps'. Reactome defines a 'reaction' as any event in biology that changes the state of a biological molecule. Binding, activation, translocation, degradation and classical biochemical events involving a catalyst are all reactions. Information in the database is authored by expert biologists, entered and maintained by Reactome's team of curators and editorial staff. Reactome content frequently cross-references other resources e.g. NCBI, Ensembl, UniProt, KEGG (Gene and Compound), ChEBI, PubMed and GO. Orthologous reactions inferred from annotation for Homo sapiens are available for 17 non-human species including mouse, rat, chicken, puffer fish, worm, fly, yeast, rice, and Arabidopsis. Pathways are represented by simple diagrams following an SBGN-like format.

Reactome's annotated data describe reactions possible if all annotated proteins and small molecules were present and active simultaneously in a cell. By overlaying an experimental dataset on these annotations, a user can perform a pathway over-representation analysis. By overlaying quantitative expression data or time series, a user can visualize the extent of change in affected pathways and its progression. A binomial test is used to calculate the probability shown for each result, and the p-values are corrected for the multiple testing (Benjamini–Hochberg procedure) that arises from evaluating the submitted list of identifiers against every pathway.

To learn more about our Pathway Analysis, please have a look at our relevant publications:

Fabregat A, Sidiropoulos K, Garapati P, Gillespie M, Hausmann K, Haw R, … D'Eustachio P (2016). The reactome pathway knowledgebase. Nucleic Acids Research, 44(D1), D481–D487. https://doi.org/10.1093/nar/gkv1351.

Fabregat A, Sidiropoulos K, Viteri G, Forner O, Marin-Garcia P, Arnau V, … Hermjakob H (2017). Reactome pathway analysis: a high-performance in-memory approach. BMC Bioinformatics, 18.

2. Properties

- This is an **overrepresentation** analysis: A statistical (hypergeometric distribution) test that determines whether certain Reactome pathways are over-represented (enriched) in the submitted data. It answers the question ‘Does my list contain more proteins for pathway X than would be expected by chance?’ This test produces a probability score, which is corrected for false discovery rate using the Benjamani-Hochberg method.
- 404 out of 526 identifiers in the sample were found in Reactome, where 717 pathways were hit by at least one of them.
- All non-human identifiers have been converted to their human equivalent.
- This report is filtered to show only results for species 'Homo sapiens' and resource 'UniProt'.
- The unique ID for this analysis (token) is MjAyMDAyMDgxODM0MzJfMjQ4NjA%3D. This ID is valid for at least 7 days in Reactome’s server. Use it to access Reactome services with your data.

3. Genome-wide overview

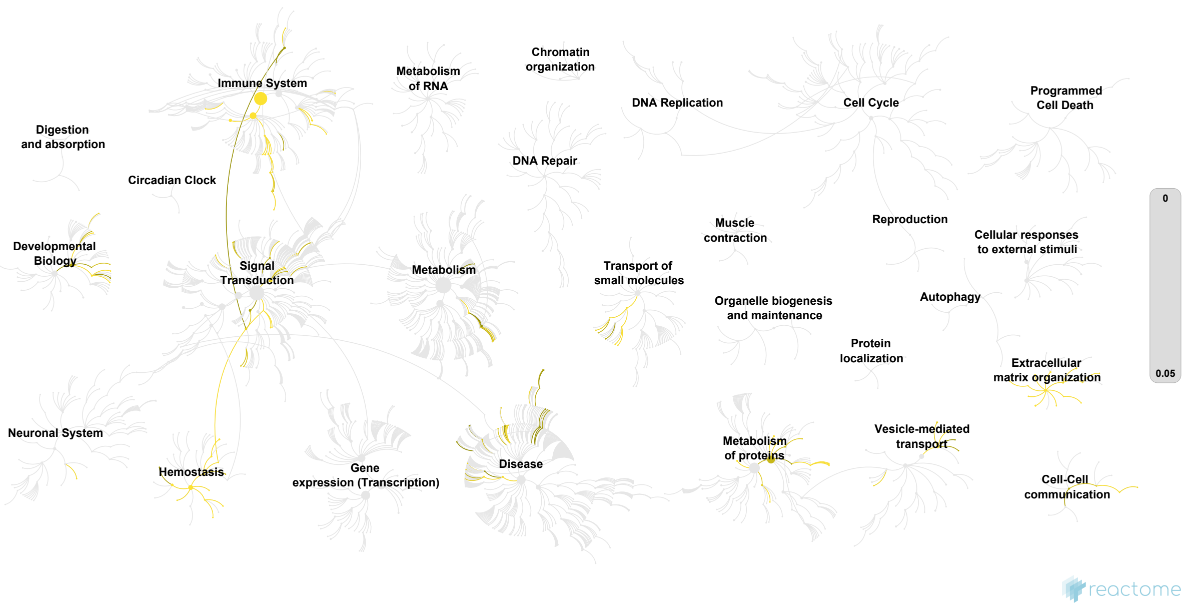

This figure shows a genome-wide overview of the results of your pathway analysis. Reactome pathways are arranged in a hierarchy. The center of each of the circular "bursts" is the root of one top-level pathway, for example "DNA Repair". Each step away from the center represents the next level lower in the pathway hierarchy. The color code denotes over-representation of that pathway in your input dataset. Light grey signifies pathways which are not significantly over-represented.

4. Most significant pathways

The following table shows the 25 most relevant pathways sorted by p-value.

Pathway name	Entities				Reactions	
	found	ratio	p-value	FDR*	found	ratio
Post-translational protein phosphorylation	38 / 107	0.009	1.11e-16	8.10e-15	1 / 1	8.01e-05
Regulation of Complement cascade	38 / 135	0.012	1.11e-16	8.10e-15	42 / 42	0.003
Complement cascade	41 / 146	0.013	1.11e-16	8.10e-15	71 / 71	0.006
Formation of Fibrin Clot (Clotting Cascade)	25 / 39	0.003	1.11e-16	8.10e-15	54 / 57	0.005
Regulation of Insulin-like Growth Factor (IGF) transport and uptake by Insulin-like Growth Factor Binding Proteins (IGFBPs)	41 / 124	0.011	1.11e-16	8.10e-15	9 / 14	0.001
Platelet degranulation	46 / 128	0.011	1.11e-16	8.10e-15	7 / 11	8.81e-04
Extracellular matrix organization	65 / 301	0.027	1.11e-16	8.10e-15	188 / 318	0.025
Response to elevated platelet cytosolic Ca2+	46 / 133	0.012	1.11e-16	8.10e-15	7 / 14	0.001
Hemostasis	92 / 723	0.064	1.11e-16	8.10e-15	162 / 327	0.026
Platelet activation, signaling and aggregation	51 / 262	0.023	1.11e-16	8.10e-15	40 / 114	0.009
Integrin cell surface interactions	28 / 85	0.007	3.55e-15	2.38e-13	52 / 54	0.004
ECM proteoglycans	26 / 76	0.007	1.47e-14	8.94e-13	19 / 23	0.002
Neutrophil degranulation	66 / 480	0.042	2.11e-14	1.18e-12	10 / 10	8.01e-04
Intrinsic Pathway of Fibrin Clot Formation	16 / 22	0.002	2.89e-14	1.50e-12	19 / 20	0.002
Innate Immune System	116 / 1,186	0.104	4.42e-14	2.17e-12	178 / 696	0.056
Common Pathway of Fibrin Clot Formation	15 / 22	0.002	4.56e-13	2.10e-11	28 / 29	0.002
Immune System	170 / 2,373	0.209	4.42e-09	1.90e-07	367 / 1,597	0.128
Non-integrin membrane-ECM interactions	16 / 59	0.005	4.18e-08	1.67e-06	13 / 22	0.002
Cell surface interactions at the vascular wall	34 / 246	0.022	5.06e-08	1.92e-06	43 / 64	0.005
Activation of C3 and C5	6 / 7	6.17e-04	1.36e-06	4.90e-05	3 / 3	2.40e-04
Terminal pathway of complement	6 / 8	7.05e-04	2.91e-06	1.02e-04	5 / 5	4.00e-04
Post-translational modification: synthesis of GPI-anchored proteins	17 / 94	0.008	4.03e-06	1.33e-04	1 / 16	0.001
Other semaphorin interactions	8 / 19	0.002	4.65e-06	1.49e-04	8 / 9	7.21e-04
Initial triggering of complement	18 / 111	0.01	9.19e-06	2.47e-04	21 / 21	0.002

Pathway name	Entities				Reactions	
	found	ratio	p-value	FDR*	found	ratio
p130Cas linkage to MAPK signaling for integrins	7 / 15	0.001	9.51e-06	2.47e-04	3 / 3	2.40e-04

* False Discovery Rate

5. Pathways details

For every pathway of the most significant pathways, we present its diagram, as well as a short summary, its bibliography and the list of inputs found in it.

1. Post-translational protein phosphorylation (R-HSA-8957275)

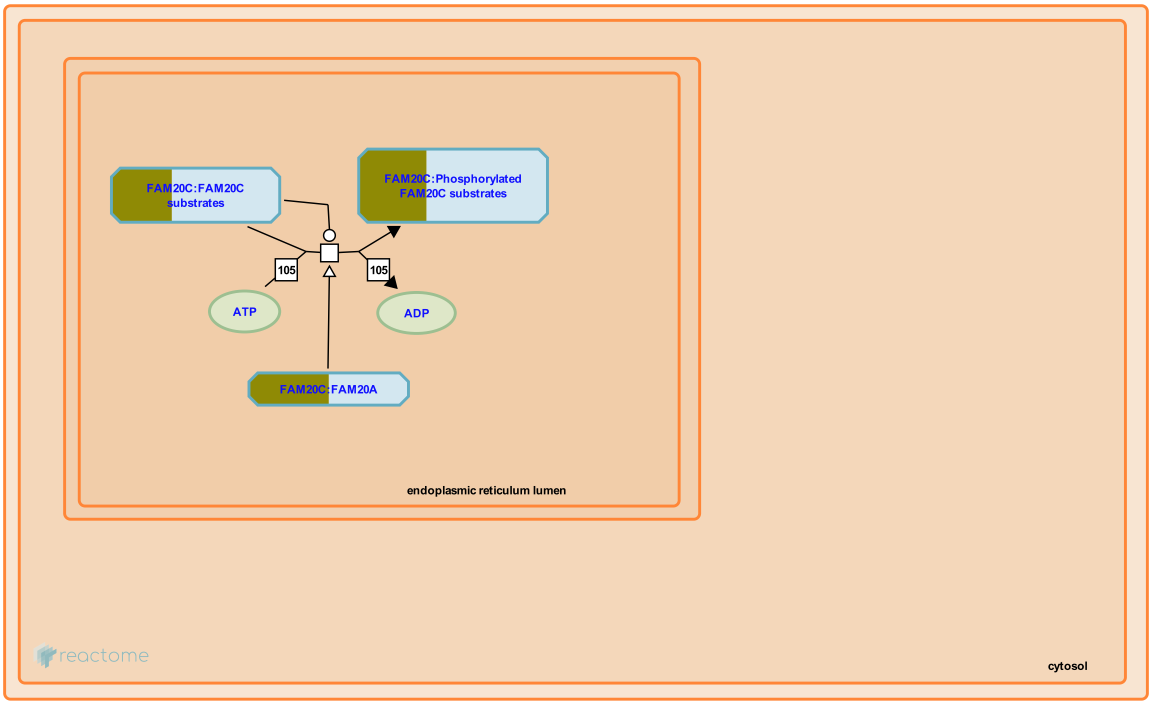

Secretory pathway kinases phosphorylate a diverse array of substrates involved in many physiological processes.

References

Sreelatha A, Kinch LN & Tagliabracci VS (2015). The secretory pathway kinases. Biochim. Biophys. Acta, 1854, 1687-93.

Edit history

Date	Action	Author
2016-12-08	Authored	Jupe S
2017-01-23	Reviewed	Wiley SE
2017-01-24	Edited	Jupe S
2017-01-24	Created	Jupe S
2019-11-21	Modified	Weiser D

Entities found in this pathway (38)

Input	UniProt Id	Input	UniProt Id	Input	UniProt Id
A1AT_HUMAN	P01009	ADA10_HUMAN	O14672	ALBU_HUMAN	P02768
ANT3_HUMAN	P01008	APOB_HUMAN	P04114	CADH2_HUMAN	P19022

Input	UniProt Id	Input	UniProt Id	Input	UniProt Id
CERU_HUMAN	P00450	CO3_HUMAN	P01024	CO4A_HUMAN	P0C0L4
FA20C_HUMAN	Q8IXL6	FA5_HUMAN	P12259	FBN1_HUMAN	P35555
FETA_HUMAN	P02771	FETUA_HUMAN	P02765	FIBA_HUMAN	P02671
FIBG_HUMAN	P02679	FINC_HUMAN	P02751	FSTL1_HUMAN	Q12841
GOLM1_HUMAN	Q8NBJ4	HEP2_HUMAN	P05546	IBP3_HUMAN	P17936
ITIH2_HUMAN	P19823	KNG1_HUMAN	P01042	LAMB2_HUMAN	P55268
LAMC1_HUMAN	P11047	LTBP1_HUMAN	Q14766	MSLN_HUMAN	Q13421
PCSK9_HUMAN	Q8NBP7	PROC_HUMAN	P04070	PRS23_HUMAN	O95084
QSOX1_HUMAN	O00391	SPRL1_HUMAN	Q14515	T132A_HUMAN	Q24JP5
TENA_HUMAN	P24821	TGON2_HUMAN	O43493	TIMP1_HUMAN	P01033
TRFE_HUMAN	P02787	ZPI_HUMAN	Q9UK55		

2. Regulation of Complement cascade (R-HSA-977606)

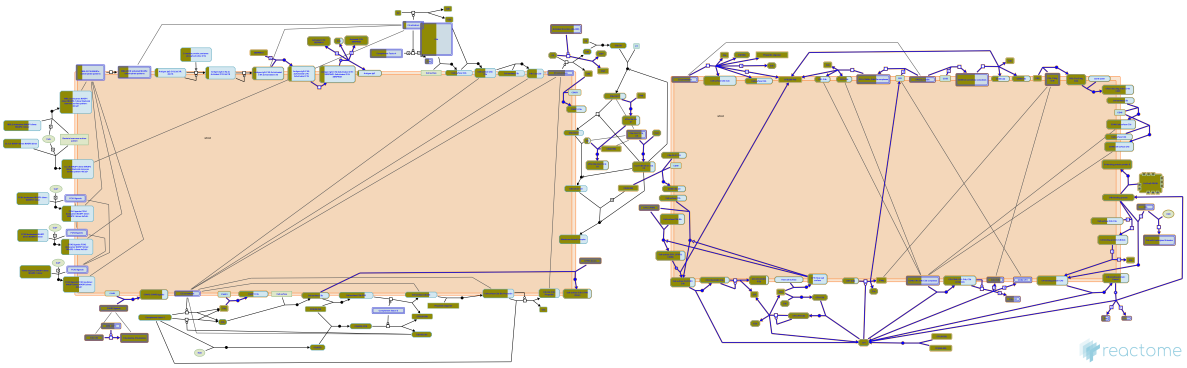

Cellular compartments: plasma membrane, extracellular region.

Two inherent features of complement activation make its regulation very important:

1. There is an inherent positive feedback loop because the product of C3 activation forms part of an enzyme that causes more C3 activation.

2. There is continuous low-level activation of the alternative pathway (see Spontaneous hydrolysis of C3 thioester).

Complement cascade activation is regulated by a family of related proteins termed the regulators of complement activation (RCA). These are expressed on healthy host cells. Most pathogens do not express RCA proteins on their surface, but many have found ways to evade the complement system by stably binding the RCA that circulates in human plasma (Lambris et al. 2008); trapping RCA is by far the most widely employed strategy for avoiding the complement response. RCA recruitment is common in bacteria such as E. coli and streptococci (Kraiczy & Wurzner 2006) and has also been described for viruses, fungi and parasites. RCA deposition and the complement system also have an important role in tissue homeostasis, clearing dead cells and debris, and preventing damage from oxidative stress (Weismann et al. 2011).

RCA proteins control complement activation in two different ways; by promoting the irreversible dissociation (decay acceleration) of complement convertases and by acting as cofactors for Complement factor I (CFI)-mediated cleavage of C3b and C4b.

Decay accelerating factor (DAF, CD55), Complement factor H (FH), Membrane Cofactor Protein (MCP) and Complement receptor 1 (CR1) are composed of arrays of tandem globular domains termed CCPs (complement control protein repeats) or SCRs (short consensus repeats). CR1, MCP and FH are cofactors for the CFI-mediated cleavage of C3b, generating iC3b. CR1 and MCP are also cofactors for C4b cleavage.

C4BP is an additional cofactor for the CFI-mediated cleavage of C4b.

References

Gasque P (2004). Complement: a unique innate immune sensor for danger signals. Mol Immunol, 41, 1089-98.

Zipfel PF & Skerka C (2009). Complement regulators and inhibitory proteins. Nat Rev Immunol, 9, 729-40.

Ricklin D, Hajishengallis G, Yang K & Lambris JD (2010). Complement: a key system for immune surveillance and homeostasis. Nat Immunol, 11, 785-97.

The Complement System. Retrieved from http://users.rcn.com/jkimball.ma.ultranet/BiologyPages/C/Complement.html

Edit history

Date	Action	Author
2010-10-20	Created	Jupe S
2010-10-26	Authored	Jupe S
2010-11-01	Edited	Jupe S
2012-02-13	Reviewed	Fraczek LA, Bradley DT
2019-11-21	Modified	Weiser D

Entities found in this pathway (38)

Input	UniProt Id	Input	UniProt Id	Input	UniProt Id
C1QA_HUMAN	P02745	C1QC_HUMAN	P02747	C1R_HUMAN	P00736
C1S_HUMAN	P09871	C4BPA_HUMAN	P04003	C4BPB_HUMAN	P20851
CBPB2_HUMAN	Q96IY4	CFAB_HUMAN	P00751	CFAH_HUMAN	P08603
CFAI_HUMAN	P05156	CLUS_HUMAN	P10909	CO2_HUMAN	P06681
CO3_HUMAN	P01024	CO4A_HUMAN	P0C0L4	CO5_HUMAN	P01031
CO6_HUMAN	P13671	CO8A_HUMAN	P07357	CO8B_HUMAN	P07358
CO9_HUMAN	P02748	CPN2_HUMAN	P22792	CR2_HUMAN	P20023
ELNE_HUMAN	P08246	FHR1_HUMAN	Q03591	FHR2_HUMAN	P36980
FHR3_HUMAN	Q02985	FHR4_HUMAN	Q92496	FHR5_HUMAN	Q9BXR6
HV434_HUMAN	P06331	IC1_HUMAN	P05155	IGHG1_HUMAN	P01857
IGHG2_HUMAN	P01859	IGHG3_HUMAN	P01860	IGHG4_HUMAN	P01861
KV502_HUMAN	P06315	PROP_HUMAN	P27918	PROS_HUMAN	P07225
THRB_HUMAN	P00734	VTNC_HUMAN	P04004		

3. Complement cascade (R-HSA-166658)

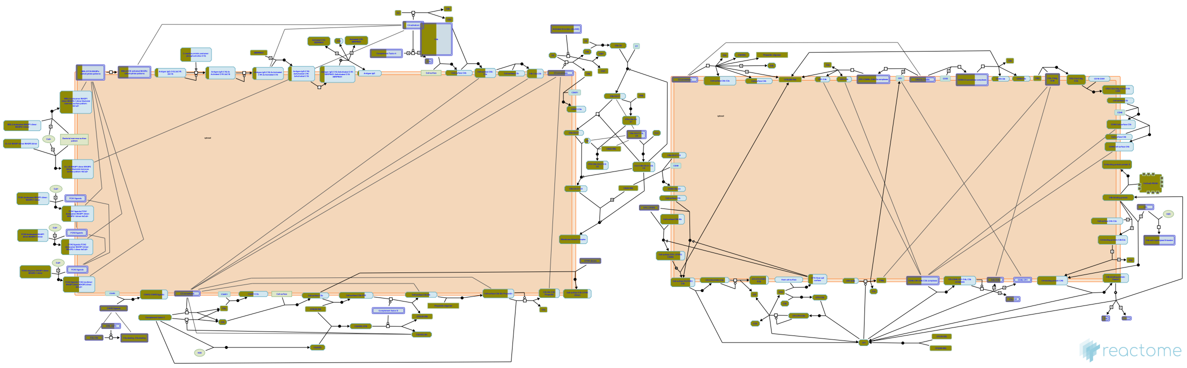

Cellular compartments: extracellular region, plasma membrane.

In the complement cascade, a panel of soluble molecules rapidly and effectively senses a danger or damage and triggers reactions to provide a response that discriminates among foreign intruders, cellular debris, healthy and altered host cells (Ricklin D et al. 2010). Complement proteins circulate in the blood stream in functionally inactive states. When triggered the complement cascade generates enzymatically active molecules (such as C3/C5 convertases) and biological effectors: opsonins (C3b, C3d and C4b), anaphylatoxins (C3a and C5a), and C5b, which initiates assembly of the lytic membrane attack complex (MAC). Three branches lead to complement activation: the classical, lectin and alternative pathways (Kang YH et al. 2009; Ricklin D et al. 2010). The classical pathway is initiated by C1 complex binding to immune complexes, pentraxins or other targets such as apoptotic cells leading to cleavage of C4 and C2 components and formation of the classical C3 convertase, C4bC2a. The lectin pathway is activated by binding of mannan-binding lectin (MBL) to repetitive carbohydrate residues, or by binding of ficolins to carbohydrate or acetylated groups on target surfaces. MBL and ficolins interact with MBL-associated serine proteases (MASP) leading to cleavage of C4 and C2 and formation of the classical C3 convertase, C4bC2a. The alternative pathway is spontaneously activated by the hydrolysis of the internal thioester group of C3 to give C3(H2O). Alternative pathway activation involves interaction of C3(H2O) and/or previously generated C3b with factor B, which is cleaved by factor D to generate the alternative C3 convertases C3(H2O)Bb and/or C3bBb. All three pathways merge at the proteolytic cleavage of component C3 by C3 convertases to form opsonin C3b and anaphylatoxin C3a. C3b covalently binds to glycoproteins scattered across the target cell surface. This is followed by an amplification reaction that generates additional C3 convertases and deposits more C3b at the local site. C3b can also bind to C3 convertases switching them to C5 convertases, which mediate C5 cleavage leading to MAC formation. Thus, the activation of the complement system leads to several important outcomes:
opsonization of target cells to enhance phagocytosis,
lysis of target cells via membrane attack complex (MAC) assembly on the cell surface,
production of anaphylatoxins C3a/C5a involved in the host inflammatory response,
C5a-mediated leukocyte chemotaxis,
and clearance of antibody-antigen complexes.

The complement system is able to distinguish between pathological and physiological challenges, i.e. the outcomes of complement activation are predetermined by the trigger and are tightly tuned by a combination of initiation events with several regulatory mechanisms. These regulatory mechanisms use soluble (e.g., C4BP, CFI and CFH) and membrane-bound regulators (e.g., CR1, CD46(MCP), CD55(DAF) and CD59) and are coordinated by complement receptors such as CR1, CR2, etc.

In response to microbial infection complement activation results in flagging microorganisms with opsonins for facilitated phagocytosis, formation of MAC on cells such as Gram-negative bacteria leading to cell lysis, and release of C3a and C5a to stimulate downstream immune responses and to attract leukocytes. Most pathogens can be eliminated by these complement-mediated host responses, though some pathogenic microorganisms have developed ways of avoiding complement recognition or blocking host complement attack resulting in greater virulence (Lambris JD et al. 2008; Serruto D et al. 2010).

All three complement pathways (classical, lectin and alternative) have been implicated in clearance of dying cells (Mevorach D et al. 1998; Ogden CA et al. 2001; Gullstrand B et al.2009; Kemper C et al. 2008). Altered surfaces of apoptotic cells are recognized by complement proteins leading to opsonization and subsequent phagocytosis. In contrast to pathogens, apoptotic cells are believed to induce only a limited complement activation by allowing opsonization of altered surfaces but restricting the terminal pathway of MAC formation (Gershov D et al. 2000; Braunschweig A and Jozsi M 2011). Thus, opsonization facilitates clearance of dying cells and cell debris without triggering danger signals and further inflammatory responses (Fraser DA et al. 2007, 2009; Benoit ME et al. 2012). C1q-mediated complement activation by apoptotic cells has been shown in a variety of human cells: keratinocytes, human umbilical vein endothelial cells (HUVEC), Jurkat T lymphoblastoid cells, lung adenocarcinoma cells (Korb LC and Ahearn JM 1997; Mold C and Morris CA 2001; Navratil JS et al. 2001; Nauta AJ et al. 2004). In addition to C1q the opsonization of apoptotic Jurkat T cells with MBL also facilitated clearance of these cells by both dendritic cells (DC) and macrophages (Nauta AJ et al. 2004). Also C3b, iC3b and C4b deposition on apoptotic cells as a consequence of activation of the complement cascade may promote complement-mediated phagocytosis. C1q, MBL and cleavage fragments of C3/C4 can bind to several receptors expressed on macrophages (e.g. cC1qR (calreticulin), CR1, CR3, CR4) suggesting a potential clearance mechanism through this interaction (Mevorach D et al. 1998; Ogden CA et al. 2001). Apoptosis is also associated with an altered expression of complement regulators on the surface of apoptotic cells. CD46 (MCP) bound to the plasma membrane of a healthy cell protects it from complement-mediated attack by preventing deposition of C3b and C4b, and reduced expression of CD46 on dying cells may lead to enhanced opsonization (Elward K et al. 2005). Upregulation of CD55 (DAF) and CD59 on apoptotic cell surfaces may protect damaged cells against complement mediated lysis (Pedersen ED et al. 2007; Iborra A et al. 2003; Hensel F et al. 2001). In addition, fluid-phase complement regulators such as C4BP, CFH may also inhibit lysis of apoptotic cells by limiting complement activation (Trouw LA et al 2007; Braunschweig A and Jozsi M. 2011).

Complement facilitates the clearance of immune complexes (IC) from the circulation (Chevalier J and Kazatchkine MD 1989; Nielsen CH et al. 1997). Erythrocytes bear clusters of complement receptor 1 (CR1 or CD35), which serves as an immune adherence receptor for C3 and/or C4 fragments deposited on IC that are shuttled to liver and spleen, where IC are transferred and processed by tissue macrophages through an Fc receptor-mediated process.

Complement proteins are always present in the blood and a small percentage spontaneously activate. Inappropriate activation leads to host cell damage, so on healthy human cells any complement activation or amplification is strictly regulated by surface-bound regulators that accelerate decay of the convertases (CR1, CD55), act as a cofactor for the factor I (CFI)-mediated degradation of C3b and C4b (CR1, CD46), or prevent the formation of MAC (CD59). Soluble regulators such as C4BP, CFH and FHL1 recognize self surface pattern-like glycosaminoglycans and further impair activation.

Complement components interact with other biological systems. Upon microbial infection complement acts in cooperation with Toll-like receptors (TLRs) to amplify innate host defense. Anaphylatoxin C5a binds C5a receptor (C5aR) resulting in a synergistic enhancement of the TLR and C5aR-mediated proinflammatory cytokine response to infection. This interplay is negatively modulated by co-ligation of TLR and the second C5a receptor, C5L2, suggesting the existence of complex immunomodulatory interactions (Kohl J 2006; Hajishengallis G and Lambris JD 2010). In addition to C5aR and C5L2, complement receptor 3 (CR3) facilitates TLR2 or TLR4 signaling pathways by promoting a recruitment of their sorting adaptor TIRAP (MAL) to the receptor complex (van Bruggen R et al. 2007; Kagan JC and Medzhitov R 2006). Complement may activate platelets or facilitate biochemical and morphological changes in the endothelium potentiating coagulation and contributing to homeostasis in response to injury (Oikonomopoulou K et al. 2012). The interplay of complement and coagulation also involves cleavage of C3 and C5 convertases by coagulation proteases, generating biologically active anaphylatoxins (Amara U et al. 2010). Complement is believed to link the innate response to both humoral and cell-mediated immunity (Toapanta FR and Ross TM 2006; Mongini PK et al. 1997). The majority of published data is based on experiments using mouse as a model organism. Further characterization of the influence of complement on B or T cell activation is required for the human system, since differences between murine models and the human system are not yet fully determined. Complement is also involved in regulation of mobilization and homing of hematopoietic stem/progenitor cells (HSPCs) from bone marrow to the circulation and peripheral tissue in order to accommodate blood cell replenishment (Reca R et al. 2006).

Thus, the complement system orchestrates the host defense by sensing a danger signal and transmitting it into specific cellular responses while extensively communicating with associated biological pathways ranging from immunity and inflammation to homeostasis and development.

N.B. Originally the larger fragment of Complement Factor 2 (C2) was designated C2a. However, complement scientists decided that the smaller of all C fragments should be designated with an 'a', the larger with a 'b', changing the nomenclature for C2. Recent literature may use the updated nomenclature and refer to the larger C2 fragment as C2b, and refer to the classical C3 convertase as C4bC2b. Throughout this pathway Reactome adheres to the original convention to agree with the current (Sep 2013) Uniprot names for C2 fragments.

References

Schmidt BZ & Colten HR (2000). Complement: a critical test of its biological importance. Immunol Rev, 178, 166-76.

Gasque P (2004). Complement: a unique innate immune sensor for danger signals. Mol Immunol, 41, 1089-98.

Nonaka M & Yoshizaki F (2004). Evolution of the complement system. Mol Immunol, 40, 897-902.

Sim RB & Laich A (2000). Serine proteases of the complement system. Biochem Soc Trans, 28, 545-50.

Muller-Eberhard HJ (1988). Molecular organization and function of the complement system. Annu Rev Biochem, 57, 321-47.

Edit history

Date	Action	Author
2004-08-04	Authored	de Bono B

Date	Action	Author
2005-09-14	Created	de Bono B
2006-07-04	Reviewed	D'Eustachio P
2010-11-11	Revised	Jupe S
2010-11-11	Edited	Jupe S
2019-11-15	Modified	Weiser D

Entities found in this pathway (41)

Input	UniProt Id	Input	UniProt Id	Input	UniProt Id
C1QA_HUMAN	P02745	C1QC_HUMAN	P02747	C1R_HUMAN	P00736
C1S_HUMAN	P09871	C4BPA_HUMAN	P04003	C4BPB_HUMAN	P20851
CBPB2_HUMAN	Q96IY4	CFAB_HUMAN	P00751	CFAH_HUMAN	P08603
CFAI_HUMAN	P05156	CLUS_HUMAN	P10909	CO2_HUMAN	P06681
CO3_HUMAN	P01024	CO4A_HUMAN	P0C0L4	CO5_HUMAN	P01031
CO6_HUMAN	P13671	CO8A_HUMAN	P07357	CO8B_HUMAN	P07358
CO9_HUMAN	P02748	CPN2_HUMAN	P22792	CR2_HUMAN	P20023
ELNE_HUMAN	P08246	FCN3_HUMAN	O75636	FHR1_HUMAN	Q03591
FHR2_HUMAN	P36980	FHR3_HUMAN	Q02985	FHR4_HUMAN	Q92496
FHR5_HUMAN	Q9BXR6	HV434_HUMAN	P06331	IC1_HUMAN	P05155
IGHG1_HUMAN	P01857	IGHG2_HUMAN	P01859	IGHG3_HUMAN	P01860
IGHG4_HUMAN	P01861	KV502_HUMAN	P06315	MASP1_HUMAN	P48740
MASP2_HUMAN	O00187-1	PROP_HUMAN	P27918	PROS_HUMAN	P07225
THRB_HUMAN	P00734	VTNC_HUMAN	P04004		

4. Formation of Fibrin Clot (Clotting Cascade) (R-HSA-140877)

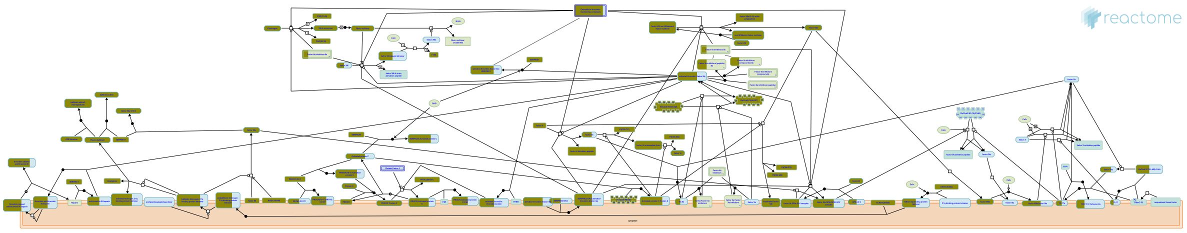

Cellular compartments: extracellular region.

The formation of a fibrin clot at the site of an injury to the wall of a normal blood vessel is an essential part of the process to stop blood loss after vascular injury. The reactions that lead to fibrin clot formation are commonly described as a cascade, in which the product of each step is an enzyme or cofactor needed for following reactions to proceed efficiently. The entire clotting cascade can be divided into three portions, the extrinsic pathway, the intrinsic pathway, and the common pathway. The extrinsic pathway begins with the release of tissue factor at the site of vascular injury and leads to the activation of factor X. The intrinsic pathway provides an alternative mechanism for activation of factor X, starting from the activation of factor XII. The common pathway consists of the steps linking the activation of factor X to the formation of a multimeric, cross-linked fibrin clot. Each of these pathways includes not only a cascade of events that generate the catalytic activities needed for clot formation, but also numerous positive and negative regulatory events.

References

Davie EW, Fujikawa K & Kisiel W (1991). The coagulation cascade: initiation, maintenance, and regulation. Biochemistry, 30, 10363-70.

Mann KG & Butenas S (2003). The dynamics of thrombin formation. Arterioscler Thromb Vasc Biol, 23, 17-25.

Edit history

Date	Action	Author
2004-08-24	Authored	D'Eustachio P
2004-08-24	Created	D'Eustachio P
2019-11-15	Modified	Weiser D

Entities found in this pathway (25)

Input	UniProt Id	Input	UniProt Id	Input	UniProt Id
A2MG_HUMAN	P01023	ANT3_HUMAN	P01008	CD177_HUMAN	Q8N6Q3
EPCR_HUMAN	Q9UNN8	F13B_HUMAN	P05160	FA11_HUMAN	P03951
FA12_HUMAN	P00748	FA5_HUMAN	P12259	FA7_HUMAN	P08709
FA8_HUMAN	P00451	FIBA_HUMAN	P02671	FIBB_HUMAN	P02675
FIBG_HUMAN	P02679	GDN_HUMAN	P07093	GP1BA_HUMAN	P07359
GPV_HUMAN	P40197	HEP2_HUMAN	P05546	IC1_HUMAN	P05155
IPSP_HUMAN	P05154	KLKB1_HUMAN	P03952	KNG1_HUMAN	P01042
PROC_HUMAN	P04070	PROS_HUMAN	P07225	THRB_HUMAN	P00734
VWF_HUMAN	P04275				

5. Regulation of Insulin-like Growth Factor (IGF) transport and uptake by Insulin-like Growth Factor Binding Proteins (IGFBPs) (R-HSA-381426)

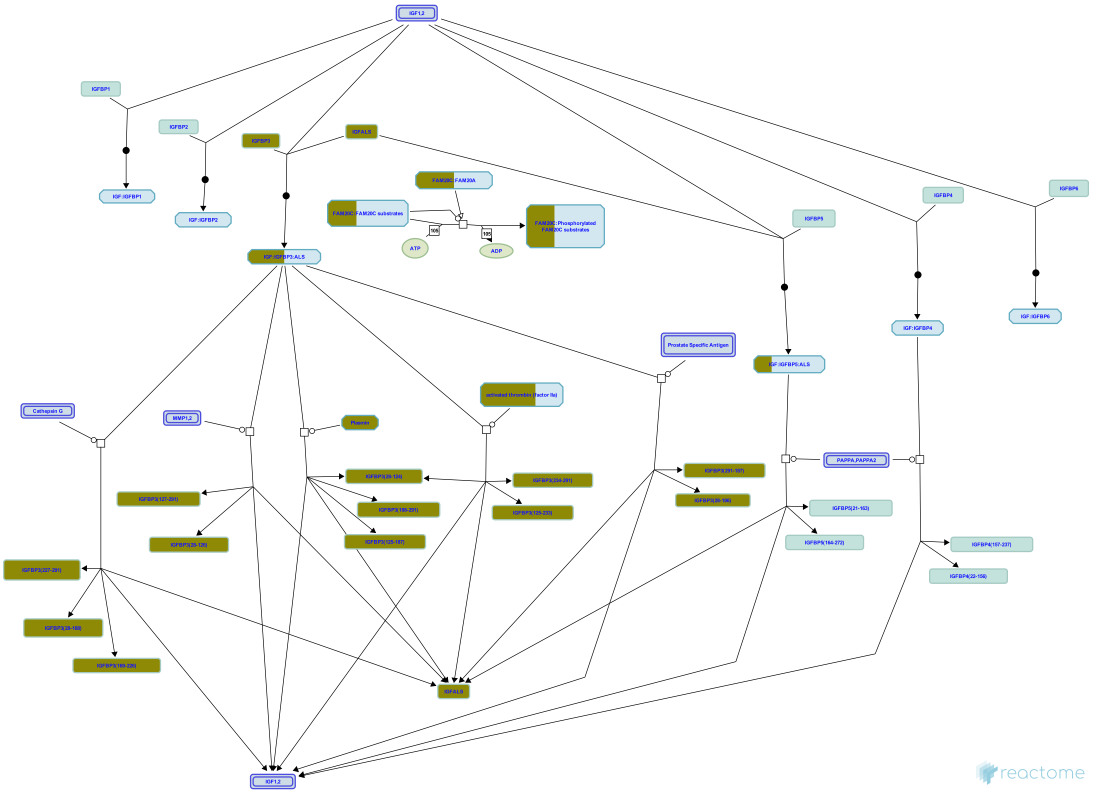

Cellular compartments: extracellular region.

The family of Insulin like Growth Factor Binding Proteins (IGFBPs) share 50% amino acid identity with conserved N terminal and C terminal regions responsible for binding Insulin like Growth Factors I and II (IGF I and IGF II). Most circulating IGFs are in complexes with IGFBPs, which are believed to increase the residence of IGFs in the body, modulate availability of IGFs to target receptors for IGFs, reduce insulin like effects of IGFs, and act as signaling molecules independently of IGFs.
About 75% of circulating IGFs are in 1500 220 KDa complexes with IGFBP3 and ALS. Such complexes are too large to pass the endothelial barrier. The remaining 20 25% of IGFs are bound to other IGFBPs in 40 50 KDa complexes. IGFs are released from IGF:IGFBP complexes by proteolysis of the IGFBP. IGFs become active after release, however IGFs may also have activity when still bound to some IGFBPs.
IGFBP1 is enriched in amniotic fluid and is produced in the liver under control of insulin (insulin suppresses production). IGFBP1 binding stimulates IGF function. It is unknown which if any protease degrades IGFBP1.
IGFBP2 is enriched in cerebrospinal fluid; its binding inhibits IGF function. IGFBP2 is not significantly degraded in circulation.
IGFB3, which binds most IGF in the body is enriched in follicular fluid and found in many other tissues. IGFBP 3 may be cleaved by plasmin, thrombin, Prostate specific Antigen (PSA, KLK3), Matrix Metalloprotease-1 (MMP1), and Matrix Metalloprotease-2 (MMP2). IGFBP3 also binds extracellular matrix and binding lowers its affinity for IGFs. IGFBP3 binding stimulates the effects of IGFs.
IGFBP4 acts to inhibit IGF function and is cleaved by Pregnancy associated Plasma Protein A (PAPPA) to release IGF.
IGFBP5 is enriched in bone matrix; its binding stimulates IGF function. IGFBP5 is cleaved by Pregnancy Associated Plasma Protein A2 (PAPPA2), ADAM9, complement C1s from smooth muscle, and thrombin. Only the cleavage site for PAPPA2 is known.
IGFBP6 is enriched in cerebrospinal fluid. It is unknown which if any protease degrades IGFBP6.

References

Firth SM & Baxter RC (2002). Cellular actions of the insulin-like growth factor binding proteins. Endocr Rev, 23, 824-54.

Hoeflich A, Reisinger R, Lahm H, Kiess W, Blum WF, Kolb HJ, ... Wolf E (2001). Insulin-like growth factor-binding protein 2 in tumorigenesis: protector or promoter?. Cancer Res, 61, 8601-10.

Mohan S & Baylink DJ (2002). IGF-binding proteins are multifunctional and act via IGF-dependent and -independent mechanisms. J Endocrinol, 175, 19-31.

Holly J & Perks C (2006). The role of insulin-like growth factor binding proteins. Neuroendocrinology, 83, 154-60.

Zhou R, Diehl D, Hoeflich A, Lahm H & Wolf E (2003). IGF-binding protein-4: biochemical characteristics and functional consequences. J Endocrinol, 178, 177-93.

Edit history

Date	Action	Author
2008-11-20	Edited	May B, Gopinathrao G
2008-11-20	Created	May B
2008-12-02	Reviewed	Matthews L, D'Eustachio P, Gillespie ME

Date	Action	Author
2019-11-15	Modified	Weiser D

Entities found in this pathway (41)

Input	UniProt Id	Input	UniProt Id	Input	UniProt Id
A1AT_HUMAN	P01009	ADA10_HUMAN	O14672	ALBU_HUMAN	P02768
ALS_HUMAN	P35858	ANT3_HUMAN	P01008	APOB_HUMAN	P04114
CADH2_HUMAN	P19022	CERU_HUMAN	P00450	CO3_HUMAN	P01024
CO4A_HUMAN	P0C0L4	FA20C_HUMAN	Q8IXL6	FA5_HUMAN	P12259
FBN1_HUMAN	P35555	FETA_HUMAN	P02771	FETUA_HUMAN	P02765
FIBA_HUMAN	P02671	FIBG_HUMAN	P02679	FINC_HUMAN	P02751
FSTL1_HUMAN	Q12841	GOLM1_HUMAN	Q8NBJ4	HEP2_HUMAN	P05546
IBP3_HUMAN	P17936	ITIH2_HUMAN	P19823	KNG1_HUMAN	P01042
LAMB2_HUMAN	P55268	LAMC1_HUMAN	P11047	LTBP1_HUMAN	Q14766
MSLN_HUMAN	Q13421	PCSK9_HUMAN	Q8NBP7	PLMN_HUMAN	P00747
PROC_HUMAN	P04070	PRS23_HUMAN	O95084	QSOX1_HUMAN	O00391
SPRL1_HUMAN	Q14515	T132A_HUMAN	Q24JP5	TENA_HUMAN	P24821
TGON2_HUMAN	O43493	THRB_HUMAN	P00734	TIMP1_HUMAN	P01033
TRFE_HUMAN	P02787	ZPI_HUMAN	Q9UK55		

6. Platelet degranulation (R-HSA-114608)

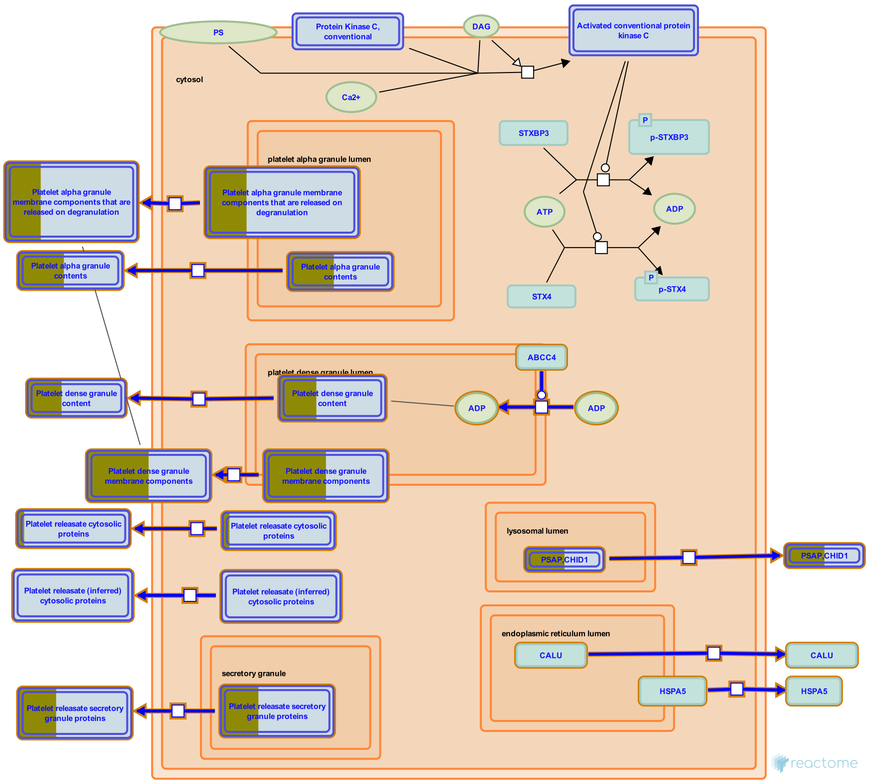

Platelets function as exocytotic cells, secreting a plethora of effector molecules at sites of vascular injury. Platelets contain a number of distinguishable storage granules including alpha granules, dense granules and lysosomes. On activation platelets release a variety of proteins, largely from storage granules but also as the result of apparent cell lysis. These act in an autocrine or paracrine fashion to modulate cell signaling.

Alpha granules contain mainly polypeptides such as fibrinogen, von Willebrand factor, growth factors and protease inhibitors that that supplement thrombin generation at the site of injury. Dense granules contain small molecules, particularly adenosine diphosphate (ADP), adenosine triphosphate (ATP), serotonin and calcium, all recruit platelets to the site of injury.

The molecular mechanism which facilitates granule release involves soluble NSF attachment protein receptors (SNAREs), which assemble into complexes to form a universal membrane fusion apparatus. Although all cells use SNAREs for membrane fusion, different cells possess different SNARE isoforms. Platelets and chromaffin cells use many of the same chaperone proteins to regulate SNARE-mediated secretion (Fitch-Tewfik & Flaumenhaft 2013).

References

Gresele P, Page CP, Fuster V & Vermylen J (2002). *Platelets in thrombotic and non-thrombotic disorders.* , 435-437.

Coppinger JA, Cagney G, Toomey S, Kislinger T, Belton O, McRedmond JP, ... Maguire PB (2004). Characterization of the proteins released from activated platelets leads to localization of novel platelet proteins in human atherosclerotic lesions. Blood, 103, 2096-104.

Edit history

Date	Action	Author
2004-09-25	Created	Farndale R, Pace NP, de Bono B
2019-11-15	Modified	Weiser D

Entities found in this pathway (46)

Input	UniProt Id	Input	UniProt Id	Input	UniProt Id
A1AG1_HUMAN	P02763	A1AG2_HUMAN	P19652	A1AT_HUMAN	P01009
A1BG_HUMAN	P04217	A2AP_HUMAN	P08697	A2MG_HUMAN	P01023
AACT_HUMAN	P01011	ALBU_HUMAN	P02768	APOH_HUMAN	P02749
CATW_HUMAN	P56202	CD109_HUMAN	Q6YHK3	CD36_HUMAN	P16671
CLUS_HUMAN	P10909	ECM1_HUMAN	Q16610	FA5_HUMAN	P12259
FA8_HUMAN	P00451	FETUA_HUMAN	P02765	FIBA_HUMAN	P02671
FIBB_HUMAN	P02675	FIBG_HUMAN	P02679	FINC_HUMAN	P02751
HRG_HUMAN	P04196	IC1_HUMAN	P05155	ISLR_HUMAN	O14498
ITB3_HUMAN	P05106	ITIH3_HUMAN	Q06033	ITIH4_HUMAN	Q14624
KAIN_HUMAN	P29622	KNG1_HUMAN	P01042	LAMP2_HUMAN	P13473
LG3BP_HUMAN	Q08380	LYAM3_HUMAN	P16109	MMRN1_HUMAN	Q13201
PDGFB_HUMAN	P01127	PLMN_HUMAN	P00747	PROS_HUMAN	P07225
QSOX1_HUMAN	O00391	SAP_HUMAN	P07602	SEPP1_HUMAN	P49908
SPRC_HUMAN	P09486	TGFB1_HUMAN	P01137	TIMP1_HUMAN	P01033
TLN1_HUMAN	Q9Y490	TRFE_HUMAN	P02787	TSP1_HUMAN	P07996
VWF_HUMAN	P04275				

7. Extracellular matrix organization (R-HSA-1474244)

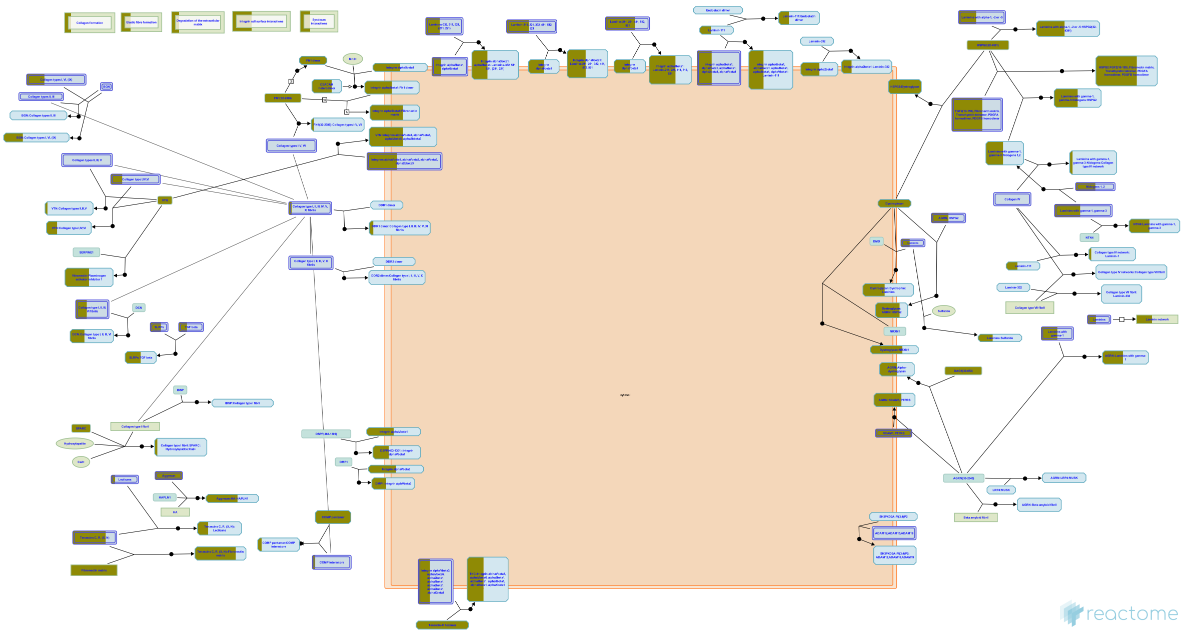

The extracellular matrix is a component of all mammalian tissues, a network consisting largely of the fibrous proteins collagen, elastin and associated-microfibrils, fibronectin and laminins embedded in a viscoelastic gel of anionic proteoglycan polymers. It performs many functions in addition to its structural role; as a major component of the cellular microenvironment it influences cell behaviours such as proliferation, adhesion and migration, and regulates cell differentiation and death (Hynes 2009).

ECM composition is highly heterogeneous and dynamic, being constantly remodeled (Frantz et al. 2010) and modulated, largely by matrix metalloproteinases (MMPs) and growth factors that bind to the ECM influencing the synthesis, crosslinking and degradation of ECM components (Hynes 2009). ECM remodeling is involved in the regulation of cell differentiation processes such as the establishment and maintenance of stem cell niches, branching morphogenesis, angiogenesis, bone remodeling, and wound repair. Redundant mechanisms modulate the expression and function of ECM modifying enzymes. Abnormal ECM dynamics can lead to deregulated cell proliferation and invasion, failure of cell death, and loss of cell differentiation, resulting in congenital defects and pathological processes including tissue fibrosis and cancer.

Collagen is the most abundant fibrous protein within the ECM constituting up to 30% of total protein in multicellular animals. Collagen provides tensile strength. It associates with elastic fibres, composed of elastin and fibrillin microfibrils, which give tissues the ability to recover after stretching. Other ECM proteins such as fibronectin, laminins, and matricellular proteins participate as connectors or linking proteins (Daley et al. 2008).

Chondroitin sulfate, dermatan sulfate and keratan sulfate proteoglycans are structural components associated with collagen fibrils (Scott & Haigh 1985; Scott & Orford 1981), serving to tether the fibril to the surrounding matrix. Decorin belongs to the small leucine-rich repeat proteoglycan family (SLRPs) which also includes biglycan, fibromodulin, lumican and asporin. All appear to be involved in collagen fibril formation and matrix assembly (Ameye & Young 2002).

ECM proteins such as osteonectin (SPARC), osteopontin and thrombospondins -1 and -2, collectively referred to as matricellular proteins (reviewed in Mosher & Adams 2012) appear to modulate cell-matrix interactions. In general they induce de-adhesion, characterized by disruption of focal adhesions and a reorganization of actin stress fibers (Bornstein 2009). Thrombospondin (TS)-1 and -2 bind MMP2. The resulting complex is endocytosed by the low-density lipoprotein receptor-related protein (LRP), clearing MMP2 from the ECM (Yang et al. 2001).

Osteopontin (SPP1, bone sialoprotein-1) interacts with collagen and fibronectin (Mukherjee et al. 1995). It also contains several cell adhesive domains that interact with integrins and CD44.

Aggrecan is the predominant ECM proteoglycan in cartilage (Hardingham & Fosang 1992). Its relatives include versican, neurocan and brevican (Iozzo 1998). In articular cartilage the major non-fibrous macromolecules are aggrecan, hyaluronan and hyaluronan and proteoglycan link protein 1 (HAPLN1). The high negative charge density of these molecules leads to the binding of large amounts of water (Bruckner 2006). Hyaluronan is bound by several large proteoglycans proteoglycans belonging to the hyalectan family that form high-molecular weight aggregates (Roughley 2006), accounting for the turgid nature of cartilage.

The most significant enzymes in ECM remodeling are the Matrix Metalloproteinase (MMP) and A disintegrin and metalloproteinase with thrombospondin motifs (ADAMTS) families (Cawston & Young 2010). Other notable ECM degrading enzymes include plasmin and cathepsin G. Many ECM proteinases are initially present as precursors, activated by proteolytic processing. MMP precursors include an amino prodomain which masks the catalytic Zn-binding motif (Page-McCawet al. 2007). This can be removed by other proteinases, often other MMPs. ECM proteinases can be inactivated by degradation, or blocked by inhibitors. Some of these inhibitors, including alpha2-macroglobulin, alpha1-proteinase inhibitor, and alpha1-chymotrypsin can inhibit a large variety of proteinases (Woessner & Nagase 2000). The tissue inhibitors of metalloproteinases (TIMPs) are potent MMP inhibitors (Brew & Nagase 2010).

References

Frantz C, Stewart KM & Weaver VM (2010). The extracellular matrix at a glance. J Cell Sci, 123, 4195-200.

Lu P, Takai K, Weaver VM & Werb Z (2011). Extracellular matrix degradation and remodeling in development and disease. Cold Spring Harb Perspect Biol, 3.

Bosman FT & Stamenkovic I (2003). Functional structure and composition of the extracellular matrix. J Pathol, 200, 423-8.

Edit history

Date	Action	Author
2011-08-05	Created	Jupe S
2011-09-09	Authored	Jupe S
2012-02-21	Edited	Jupe S
2012-02-28	Reviewed	D'Eustachio P
2013-05-21	Reviewed	Venkatesan N
2013-05-22	Reviewed	Ricard-Blum S

Date	Action	Author
2019-11-15	Modified	Weiser D

Entities found in this pathway (65)

Input	UniProt Id	Input	UniProt Id	Input	UniProt Id
A2MG_HUMAN	P01023	ADA10_HUMAN	O14672	ADAM8_HUMAN	P78325
ASPN_HUMAN	Q9BXN1	ATS2_HUMAN	O95450	BASI_HUMAN	P35613
BMP1_HUMAN	P13497	CD44_HUMAN	P16070	CEAM1_HUMAN	P13688
CEAM8_HUMAN	P31997	CO6A2_HUMAN	P12110	CO6A3_HUMAN	P12111
CO6A6_HUMAN	A6NMZ7	COBA2_HUMAN	P13942	COMP_HUMAN	P49747
DAG1_HUMAN	Q14118	ELNE_HUMAN	P08246	FBLN1_HUMAN	P23142
FBLN4_HUMAN	O95967	FBN1_HUMAN	P35555	FIBA_HUMAN	P02671
FIBB_HUMAN	P02675	FIBG_HUMAN	P02679	FINC_HUMAN	P02751
FMOD_HUMAN	Q06828	ICAM1_HUMAN	P05362	ICAM2_HUMAN	P13598
ICAM3_HUMAN	P32942	ITA1_HUMAN	P56199	ITA9_HUMAN	Q13797
ITAM_HUMAN	P11215	ITB1_HUMAN	P05556	ITB2_HUMAN	P05107
ITB3_HUMAN	P05106	KLKB1_HUMAN	P03952	LAMA2_HUMAN	P24043
LAMA4_HUMAN	Q16363	LAMB2_HUMAN	P55268	LAMC1_HUMAN	P11047
LTBP1_HUMAN	Q14766	LUM_HUMAN	P51884	LYOX_HUMAN	P28300
NCAM1_HUMAN	P13591	NID2_HUMAN	Q14112	PCOC1_HUMAN	Q15113
PDGFB_HUMAN	P01127	PGBM_HUMAN	P98160	PGCA_HUMAN	P16112
PLMN_HUMAN	P00747	PPIB_HUMAN	P23284	PTPRS_HUMAN	Q13332
PXDN_HUMAN	Q92626	SPRC_HUMAN	P09486	TENA_HUMAN	P24821
TENN_HUMAN	Q9UQP3	TENX_HUMAN	P22105	TGFB1_HUMAN	P01137
TIMP1_HUMAN	P01033	TLL1_HUMAN	O43897	TSP1_HUMAN	P07996
TTHY_HUMAN	P02766	VCAM1_HUMAN	P19320	VGFR2_HUMAN	P35968
VTNC_HUMAN	P04004	VWF_HUMAN	P04275		

8. Response to elevated platelet cytosolic Ca2+ (R-HSA-76005)

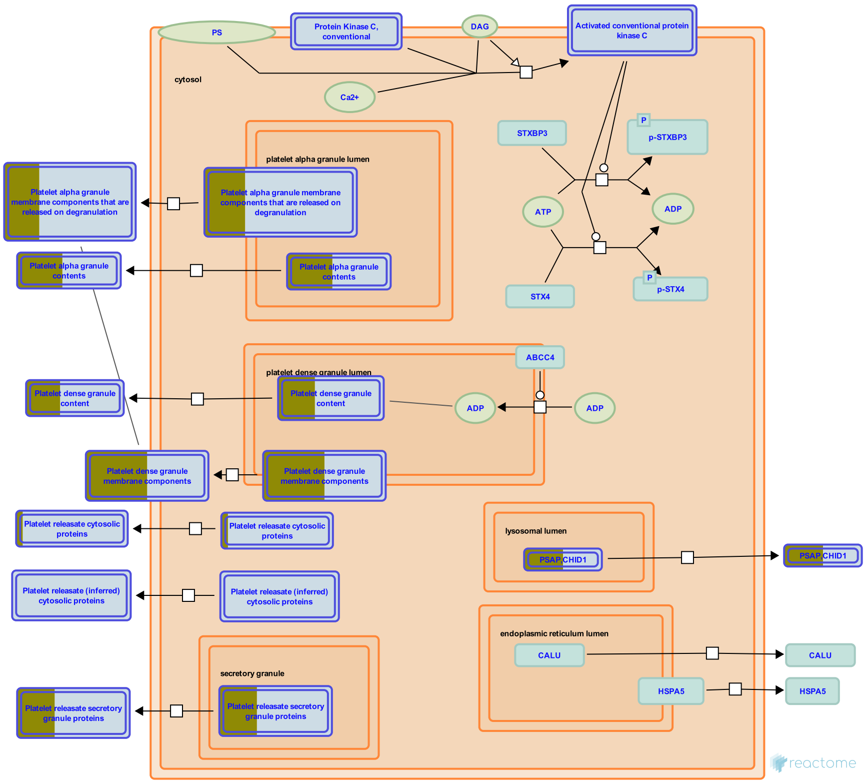

Activation of phospholipase C enzymes results in the generation of second messengers of the phosphatidylinositol pathway. The events resulting from this pathway are a rise in intracellular calcium and activation of Protein Kinase C (PKC). Phospholipase C cleaves the phosphodiester bond in PIP2 to form 1,2 Diacylglycerol (DAG) and 1,4,5-inositol trisphosphate (IP3). IP3 opens Ca2+ channels in the platelet dense tubular system, raising intracellular Ca2+ levels. DAG is a second messenger that regulates a family of Ser/Thr kinases consisting of PKC isozymes (Nishizuka 1995). DAG achieves activation of PKC isozymes by increasing their affinity for phospholipid. Most PKC enzymes are also calcium-dependent, so their activation is in synergy with the rise in intracellular Ca2+. Platelets contain several PKC isoforms that can be activated by DAG and/or Ca2+ (Chang 1997).

References

Walker TR & Watson SP (1993). Synergy between Ca2+ and protein kinase C is the major factor in determining the level of secretion from human platelets. Biochem J, 289, 277-82.

Edit history

Date	Action	Author
2004-08-13	Authored	de Bono B
2004-09-25	Created	Farndale R, Pace NP, de Bono B
2019-11-15	Modified	Weiser D

Entities found in this pathway (46)

Input	UniProt Id	Input	UniProt Id	Input	UniProt Id
A1AG1_HUMAN	P02763	A1AG2_HUMAN	P19652	A1AT_HUMAN	P01009
A1BG_HUMAN	P04217	A2AP_HUMAN	P08697	A2MG_HUMAN	P01023

Input	UniProt Id	Input	UniProt Id	Input	UniProt Id
AACT_HUMAN	P01011	ALBU_HUMAN	P02768	APOH_HUMAN	P02749
CATW_HUMAN	P56202	CD109_HUMAN	Q6YHK3	CD36_HUMAN	P16671
CLUS_HUMAN	P10909	ECM1_HUMAN	Q16610	FA5_HUMAN	P12259
FA8_HUMAN	P00451	FETUA_HUMAN	P02765	FIBA_HUMAN	P02671
FIBB_HUMAN	P02675	FIBG_HUMAN	P02679	FINC_HUMAN	P02751
HRG_HUMAN	P04196	IC1_HUMAN	P05155	ISLR_HUMAN	O14498
ITB3_HUMAN	P05106	ITIH3_HUMAN	Q06033	ITIH4_HUMAN	Q14624
KAIN_HUMAN	P29622	KNG1_HUMAN	P01042	LAMP2_HUMAN	P13473
LG3BP_HUMAN	Q08380	LYAM3_HUMAN	P16109	MMRN1_HUMAN	Q13201
PDGFB_HUMAN	P01127	PLMN_HUMAN	P00747	PROS_HUMAN	P07225
QSOX1_HUMAN	O00391	SAP_HUMAN	P07602	SEPP1_HUMAN	P49908
SPRC_HUMAN	P09486	TGFB1_HUMAN	P01137	TIMP1_HUMAN	P01033
TLN1_HUMAN	Q9Y490	TRFE_HUMAN	P02787	TSP1_HUMAN	P07996
VWF_HUMAN	P04275				

9. Hemostasis (R-HSA-109582)

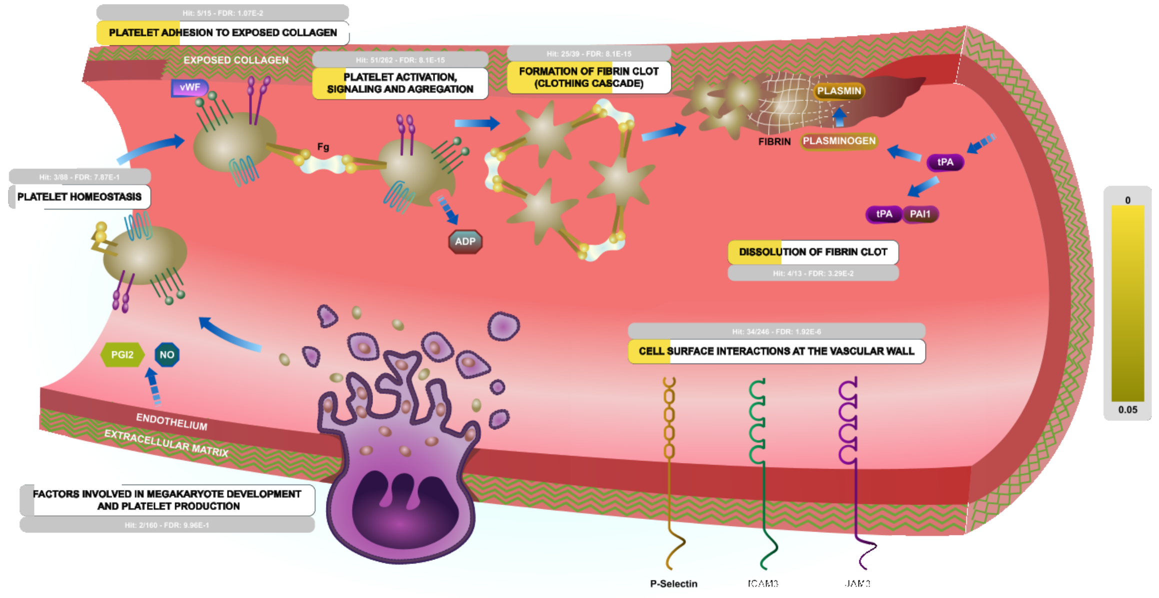

Hemostasis is a physiological response that culminates in the arrest of bleeding from an injured vessel. Under normal conditions the vascular endothelium supports vasodilation, inhibits platelet adhesion and activation, suppresses coagulation, enhances fibrin cleavage and is anti-inflammatory in character. Under acute vascular trauma, vasoconstrictor mechanisms predominate and the endothelium becomes prothrombotic, procoagulatory and proinflammatory in nature. This is achieved by a reduction of endothelial dilating agents: adenosine, NO and prostacyclin; and by the direct action of ADP, serotonin and thromboxane on vascular smooth muscle cells to elicit their contraction (Becker et al. 2000).

The chief trigger for the change in endothelial function that leads to the formation of a haemostatic thrombus is the loss of the endothelial cell barrier between blood and extracellular matrix components (Ruggeri 2002). Circulating platelets identify and discriminate areas of endothelial lesions; here, they adhere to the exposed sub endothelium. Their interaction with the various thrombogenic substrates and locally generated or released agonists results in platelet activation. This process is described as possessing two stages, firstly, adhesion - the initial tethering to a surface, and secondly aggregation - the platelet-platelet cohesion (Savage & Cattaneo et al. 2001).

Three mechansism contribute to the loss of blood following vessel injury. The vessel constricts, reducing the loss of blood. Platelets adhere to the site of injury, become activated and aggregate with fibrinogen into a soft plug that limits blood loss, a process termed primary hemostasis. Proteins and small molecules are released from granules by activated platelets, stimulating the plug formation process. Fibrinogen from plasma forms bridges between activated platelets. These events initiate the clotting cascade (secondary hemostasis). Negatively-charged phospholipids exposed at the site of injury and on activated platelets interact with tissue factor, leading to a cascade of reactions that culminates with the formation of an insoluble fibrin clot.

References

Colman R, Marder V, Clowes A, George J & Goldhaber S (2006). *Hemostasis and Thrombosis: Basic Principles and Clinical Practice* .

Allford S & Machin S (2004). *Haemostasis* , 200a - 200d.

Edit history

Date	Action	Author
2004-01-22	Authored	Farndale R, Pace NP, D'Eustachio P, de Bono B
2004-01-22	Created	Farndale R, Pace NP, D'Eustachio P, de Bono B
2019-11-13	Edited	Joshi-Tope G
2019-11-13	Reviewed	Brummel K, Stafford DW, Rush MG
2019-11-15	Modified	Weiser D

Entities found in this pathway (92)

Input	UniProt Id	Input	UniProt Id	Input	UniProt Id
4F2_HUMAN	P08195	A1AG1_HUMAN	P02763	A1AG2_HUMAN	P19652
A1AT_HUMAN	P01009	A1BG_HUMAN	P04217	A2AP_HUMAN	P08697
A2MG_HUMAN	P01023	AACT_HUMAN	P01011	ALBU_HUMAN	P02768
ANGP1_HUMAN	Q15389	ANT3_HUMAN	P01008	APOB_HUMAN	P04114
APOH_HUMAN	P02749	AT2B2_HUMAN	Q01814	BASI_HUMAN	P35613
CATW_HUMAN	P56202	CD109_HUMAN	Q6YHK3	CD177_HUMAN	Q8N6Q3
CD36_HUMAN	P16671	CD44_HUMAN	P16070	CD48_HUMAN	P09326
CEAM1_HUMAN	P13688	CEAM5_HUMAN	P06731	CEAM8_HUMAN	P31997
CLUS_HUMAN	P10909	ECM1_HUMAN	Q16610	EPCR_HUMAN	Q9UNN8
ESAM_HUMAN	Q96AP7	F13B_HUMAN	P05160	FA11_HUMAN	P03951
FA12_HUMAN	P00748	FA5_HUMAN	P12259	FA7_HUMAN	P08709
FA8_HUMAN	P00451	FETUA_HUMAN	P02765	FIBA_HUMAN	P02671
FIBB_HUMAN	P02675	FIBG_HUMAN	P02679	FINC_HUMAN	P02751
G6B_HUMAN	O95866	GDN_HUMAN	P07093	GP1BA_HUMAN	P07359
GPV_HUMAN	P40197	HEP2_HUMAN	P05546	HG2A_HUMAN	P04233
HRG_HUMAN	P04196	HV434_HUMAN	P06331	IC1_HUMAN	P05155
IGHA1_HUMAN	P01876	IGHA2_HUMAN	P01877	IGHM_HUMAN	P01871
IGJ_HUMAN	P01591	IPSP_HUMAN	P05154	ISLR_HUMAN	O14498
ITA1_HUMAN	P56199	ITAM_HUMAN	P11215	ITB1_HUMAN	P05556
ITB2_HUMAN	P05107	ITB3_HUMAN	P05106	ITIH3_HUMAN	Q06033
ITIH4_HUMAN	Q14624	ITPR2_HUMAN	Q14571	KAIN_HUMAN	P29622
KLKB1_HUMAN	P03952	KNG1_HUMAN	P01042	KV502_HUMAN	P06315
L1CAM_HUMAN	P32004	LAMP2_HUMAN	P13473	LG3BP_HUMAN	Q08380
LYAM1_HUMAN	P14151	LYAM2_HUMAN	P16581	LYAM3_HUMAN	P16109
MERTK_HUMAN	Q12866	MMRN1_HUMAN	Q13201	PDGFB_HUMAN	P01127
PLMN_HUMAN	P00747	PROC_HUMAN	P04070	PROS_HUMAN	P07225
QSOX1_HUMAN	O00391	SAP_HUMAN	P07602	SEPP1_HUMAN	P49908
SPRC_HUMAN	P09486	TBA1C_HUMAN	Q9BQE3	TBB2A_HUMAN	Q13885
TGFB1_HUMAN	P01137	THRB_HUMAN	P00734	TIE2_HUMAN	Q02763
TIMP1_HUMAN	P01033	TLN1_HUMAN	Q9Y490	TRFE_HUMAN	P02787
TSP1_HUMAN	P07996	VWF_HUMAN	P04275		

10. Platelet activation, signaling and aggregation (R-HSA-76002)

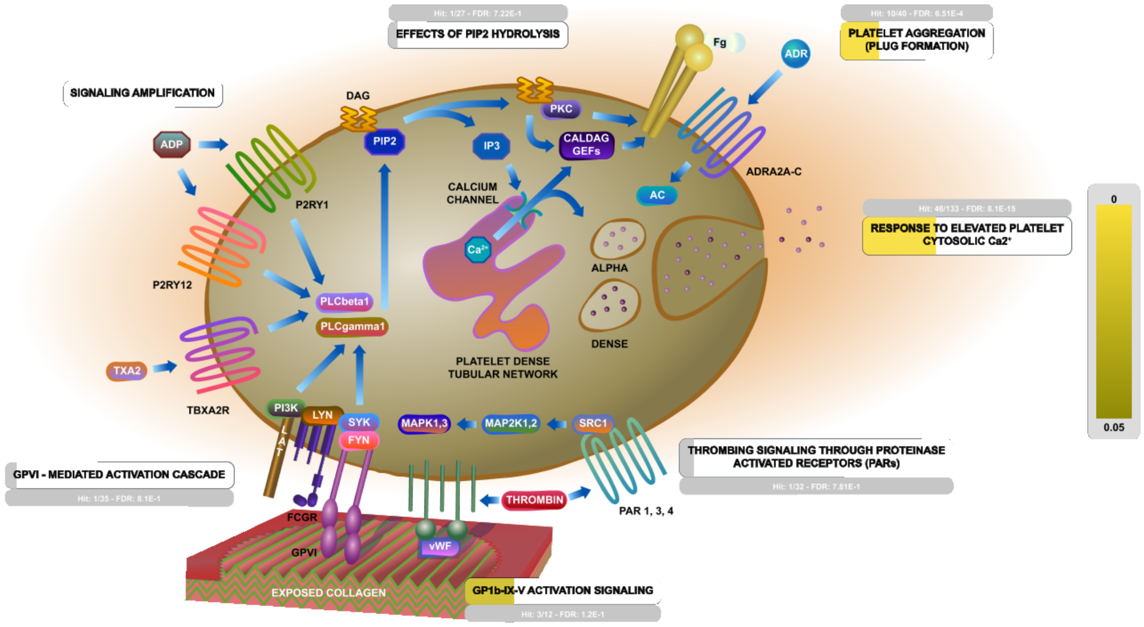

Platelet activation begins with the initial binding of adhesive ligands and of the excitatory platelet agonists (released or generated at the sites of vascular trauma) to cognate receptors on the platelet membrane (Ruggeri 2002). Intracellular signaling reactions then enhance the adhesive and procoagulant properties of tethered platelets or of platelets circulating in the proximity. Once platelets have adhered they degranulate, releasing stored secondary agents such as ADP, ATP, and synthesize thromboxane A2. These amplify the response, activating and recruiting further platelets to the area and promoting platelet aggregation. These amplify the response, activating and recruiting further platelets to the area and promoting platelet aggregation. Adenosine nucleotides signal through P2 purinergic receptors on the platelet membrane. ADP activates P2Y1 and P2Y12, which signal via both the alpha and gamma:beta components of the heterotrimeric G-protein (Hirsch et al. 2001, 2006),
while ATP activates the ionotropic P2X1 receptor (Kunapuli et al. 2003). Activation of these receptors initiates a complex signaling cascade that ultimately results in platelet activation, aggregation and thrombus formation (Kahner et al. 2006).

Integrin AlphaIIbBeta3 is the most abundant platelet receptor, with 40 000 to 80 000 copies per resting platelet, acting as a major receptor for fibrinogen and other adhesive molecules (Wagner et al. 1996). Activation of AlphaIIbBeta3 enhances adhesion and leads to platelet-platelet interactions, and thus aggregation (Philips et al. 1991). GP VI is the most potent collagen receptor initiating signal generation, an ability derived from its interaction with the FcRI gamma chain. This results in the phosphorylation of the gamma-chain by non-receptor tyrosine kinases of the Src family (1). The phosphotyrosine motif is recognized by the SH2 domains of Syk, a tyrosine kinase. This association activates the Syk enzyme, leading to activation (by tyrosine phosphorylation) of PLC gamma2 (2). Thrombin is an important platelet agonist generated on the membrane of stimulated platelets. Thrombin acts via cell surface Protease Activated Receptors (PARs). PARs are G-protein coupled receptors activated by a proteolytic cleavage in an extracellular loop (Vu, 1991) (3). Activated PARs signal via G alpha q (4) and via the beta:gamma component of the G-protein (5). Both stimulate PLC giving rise to PIP2 hydrolysis and consequent activation of PI3K (6). PLCgamma2 activation also gives rise to IP3 (7) which stimulates the IP3 receptor (8) leading to increased intracellular calcium. Platelet activation further results in the scramblase-mediated transport of negatively-charged phospholipids to the platelet surface. These phospholipids provide a catalytic surface (with the charge provided by phosphatidylserine and phosphatidylethanolamine) for the tenase complex (formed by the activated forms of the blood coagulation factors factor VIII and factor I).

References

Wagner CL, Mascelli MA, Neblock DS, Weisman HF, Coller BS & Jordan RE (1996). Analysis of GPIIb/IIIa receptor number by quantification of 7E3 binding to human platelets. Blood, 88, 907-14.

Phillips DR, Charo IF & Scarborough RM (1991). GPIIb-IIIa: the responsive integrin. Cell, 65, 359-62.

Vu TK, Hung DT, Wheaton VI & Coughlin SR (1991). Molecular cloning of a functional thrombin receptor reveals a novel proteolytic mechanism of receptor activation. Cell, 64, 1057-68.

Kahner BN, Shankar H, Murugappan S, Prasad GL & Kunapuli SP (2006). Nucleotide receptor signaling in platelets. J Thromb Haemost, 4, 2317-26.

Kunapuli SP, Dorsam RT, Kim S & Quinton TM (2003). Platelet purinergic receptors. Curr Opin Pharmacol, 3, 175-80.

Edit history

Date	Action	Author
2004-08-13	Authored	de Bono B
2004-09-25	Created	Farndale R, Pace NP, de Bono B
2010-06-07	Revised	Jupe S
2010-06-07	Reviewed	Kunapuli SP
2019-11-15	Modified	Weiser D

Entities found in this pathway (51)

Input	UniProt Id	Input	UniProt Id	Input	UniProt Id
A1AG1_HUMAN	P02763	A1AG2_HUMAN	P19652	A1AT_HUMAN	P01009
A1BG_HUMAN	P04217	A2AP_HUMAN	P08697	A2MG_HUMAN	P01023
AACT_HUMAN	P01011	ALBU_HUMAN	P02768	APOH_HUMAN	P02749
CATW_HUMAN	P56202	CD109_HUMAN	Q6YHK3	CD36_HUMAN	P16671
CLUS_HUMAN	P10909	ECM1_HUMAN	Q16610	FA5_HUMAN	P12259
FA8_HUMAN	P00451	FETUA_HUMAN	P02765	FIBA_HUMAN	P02671
FIBB_HUMAN	P02675	FIBG_HUMAN	P02679	FINC_HUMAN	P02751
G6B_HUMAN	O95866	GP1BA_HUMAN	P07359	GPV_HUMAN	P40197
HRG_HUMAN	P04196	IC1_HUMAN	P05155	ISLR_HUMAN	O14498
ITB3_HUMAN	P05106	ITIH3_HUMAN	Q06033	ITIH4_HUMAN	Q14624
ITPR2_HUMAN	Q14571	KAIN_HUMAN	P29622	KNG1_HUMAN	P01042
LAMP2_HUMAN	P13473	LG3BP_HUMAN	Q08380	LYAM3_HUMAN	P16109
MMRN1_HUMAN	Q13201	PDGFB_HUMAN	P01127	PLMN_HUMAN	P00747
PROS_HUMAN	P07225	QSOX1_HUMAN	O00391	SAP_HUMAN	P07602
SEPP1_HUMAN	P49908	SPRC_HUMAN	P09486	TGFB1_HUMAN	P01137
THRB_HUMAN	P00734	TIMP1_HUMAN	P01033	TLN1_HUMAN	Q9Y490
TRFE_HUMAN	P02787	TSP1_HUMAN	P07996	VWF_HUMAN	P04275

11. Integrin cell surface interactions (R-HSA-216083)

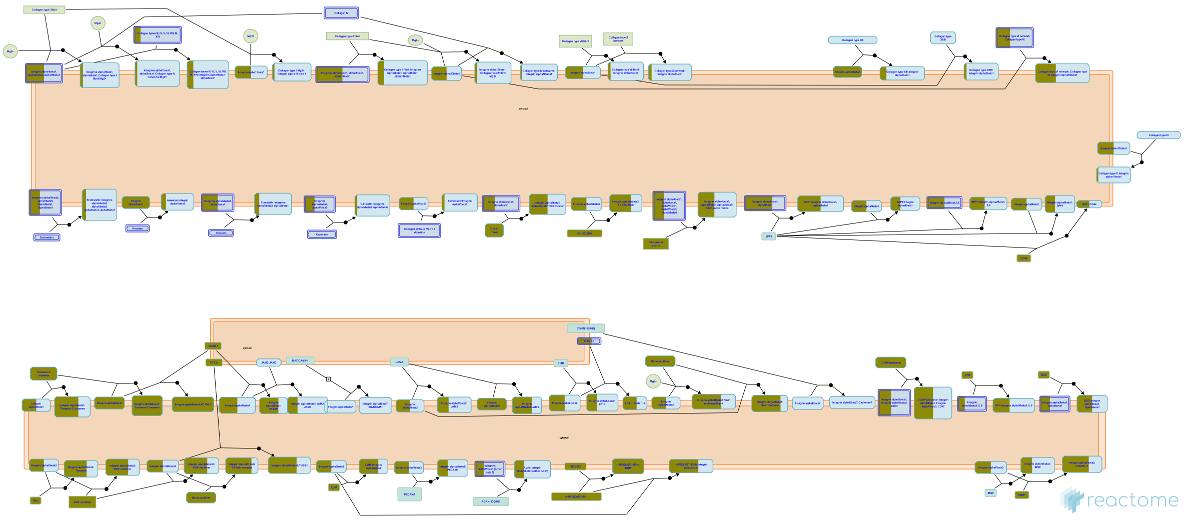

The extracellular matrix (ECM) is a network of macro-molecules that underlies all epithelia and endothelia and that surrounds all connective tissue cells. This matrix provides the mechanical strength and also influences the behavior and differentiation state of cells in contact with it. The ECM are diverse in composition, but they generally comprise a mixture of fibrillar proteins, polysaccharides synthesized, secreted and organized by neighboring cells. Collagens, fibronectin, and laminins are the principal components involved in cell matrix interactions; other components, such as vitronectin, thrombospondin, and osteopontin, although less abundant, are also important adhesive molecules.

Integrins are the receptors that mediate cell adhesion to ECM. Integrins consists of one alpha and one beta subunit forming a noncovalently bound heterodimer. 18 alpha and 8 beta subunits have been identified in humans that combine to form 24 different receptors.

The integrin dimers can be broadly divided into three families consisting of the beta1, beta2/beta7, and beta3/alphaV integrins. beta1 associates with 12 alpha-subunits and can be further divided into RGD-, collagen-, or laminin binding and the related alpha4/alpha9 integrins that recognise both matrix and vascular ligands. beta2/beta7 integrins are restricted to leukocytes and mediate cell-cell rather than cell-matrix interactions, although some recognize fibrinogen. The beta3/alphaV family members are all RGD receptors and comprise aIIbb3, an important receptor on platelets, and the remaining b-subunits, which all associate with alphaV. It is the collagen receptors and leukocyte-specific integrins that contain alpha A-domains.

References

Faull RJ & Ginsberg MH (1996). Inside-out signaling through integrins. J Am Soc Nephrol, 7, 1091-7.

Arnaout MA, Goodman SL & Xiong JP (2002). Coming to grips with integrin binding to ligands. Curr Opin Cell Biol, 14, 641-51.

White DJ, Puranen S, Johnson MS & Heino J (2004). The collagen receptor subfamily of the integrins. Int J Biochem Cell Biol, 36, 1405-10.

Boudreau NJ & Jones PL (1999). Extracellular matrix and integrin signalling: the shape of things to come. Biochem. J., 339, 481-8.

Lal H, Verma SK, Foster DM, Golden HB, Reneau JC, Watson LE, ... Dostal DE (2009). Integrins and proximal signaling mechanisms in cardiovascular disease. Front. Biosci., 14, 2307-34.

Edit history

Date	Action	Author
2008-03-11	Edited	Garapati P V
2008-03-11	Created	Garapati P V
2008-05-07	Reviewed	Hynes R, Humphries MJ, Yamada KM
2008-05-07	Authored	Geiger B, Horwitz AR
2012-08-08	Authored	Jupe S
2013-08-13	Edited	Jupe S
2013-08-13	Reviewed	Ricard-Blum S
2019-11-15	Modified	Weiser D

Entities found in this pathway (28)

Input	UniProt Id	Input	UniProt Id	Input	UniProt Id
BASI_HUMAN	P35613	CD44_HUMAN	P16070	CO6A2_HUMAN	P12110
CO6A3_HUMAN	P12111	CO6A6_HUMAN	A6NMZ7	COMP_HUMAN	P49747
FBN1_HUMAN	P35555	FIBA_HUMAN	P02671	FIBB_HUMAN	P02675
FIBG_HUMAN	P02679	FINC_HUMAN	P02751	ICAM1_HUMAN	P05362
ICAM2_HUMAN	P13598	ICAM3_HUMAN	P32942	ITA1_HUMAN	P56199
ITA9_HUMAN	Q13797	ITAM_HUMAN	P11215	ITB1_HUMAN	P05556
ITB2_HUMAN	P05107	ITB3_HUMAN	P05106	LUM_HUMAN	P51884
PGBM_HUMAN	P98160	TENA_HUMAN	P24821	TSP1_HUMAN	P07996
VCAM1_HUMAN	P19320	VGFR2_HUMAN	P35968	VTNC_HUMAN	P04004
VWF_HUMAN	P04275				

12. ECM proteoglycans (R-HSA-3000178)

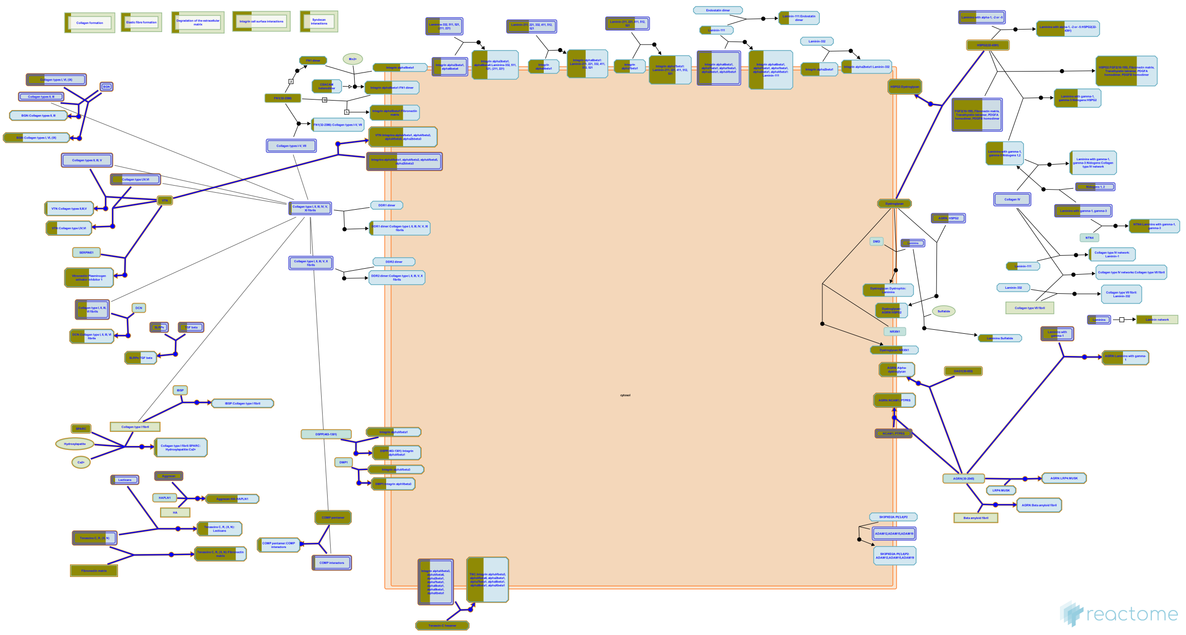

Proteoglycans are major components of the extracellular matrix. In cartilage the matrix constitutes more than 90% of tissue dry weight. Proteoglycans are proteins substituted with glycosaminoglycans (GAGs), linear polysaccharides consisting of a repeating disaccharide, generally of an acetylated amino sugar alternating

with a uronic acid. Most proteoglycans are located in the extracellular

space. Proteoglycans are highly diverse, both in terms of the core proteins and the subtypes of GAG chains, namely chondroitin sulfate (CS), keratan sulfate (KS), dermatan sulfate (DS) and heparan sulfate (HS). Hyaluronan is a non-sulfated GAG whose molecular weight runs into millions of Dalton; in articular cartilage, a single hyaluronan molecule can hold upto 100 aggrecan molecules and these aggregates are stabilized by a link protein.

References

Esko JD, Esko JD, Kimata K, Lindahl U, Varki A, Cummings RD, ... Etzler ME (2009). *Proteoglycans and Sulfated Glycosaminoglycans* .

Kim SH, Turnbull J & Guimond S (2011). Extracellular matrix and cell signalling: the dynamic co-operation of integrin, proteoglycan and growth factor receptor. J. Endocrinol., 209, 139-51.

Hay E (1991). *Cell Biology of Extracellular Matrix* .

Hay E (1991). *Proteoglycans: structure and function, Cell Biology of Extracellular Matrix* .

Edit history

Date	Action	Author
2013-01-10	Authored	Jupe S
2013-01-24	Created	Jupe S
2013-04-26	Edited	Jupe S
2013-05-21	Reviewed	Venkatesan N
2013-05-22	Reviewed	Ricard-Blum S

Date	Action	Author
2019-11-15	Modified	Weiser D

Entities found in this pathway (26)

Input	UniProt Id	Input	UniProt Id	Input	UniProt Id
ASPN_HUMAN	Q9BXN1	CO6A2_HUMAN	P12110	CO6A3_HUMAN	P12111
CO6A6_HUMAN	A6NMZ7	COMP_HUMAN	P49747	DAG1_HUMAN	Q14118
FINC_HUMAN	P02751	FMOD_HUMAN	Q06828	ITA9_HUMAN	Q13797
ITB1_HUMAN	P05556	ITB3_HUMAN	P05106	LAMA2_HUMAN	P24043
LAMA4_HUMAN	Q16363	LAMB2_HUMAN	P55268	LAMC1_HUMAN	P11047
LUM_HUMAN	P51884	NCAM1_HUMAN	P13591	PGBM_HUMAN	P98160
PGCA_HUMAN	P16112	PTPRS_HUMAN	Q13332	SPRC_HUMAN	P09486
TENA_HUMAN	P24821	TENN_HUMAN	Q9UQP3	TENX_HUMAN	P22105
TGFB1_HUMAN	P01137	VTNC_HUMAN	P04004		

13. Neutrophil degranulation (R-HSA-6798695)

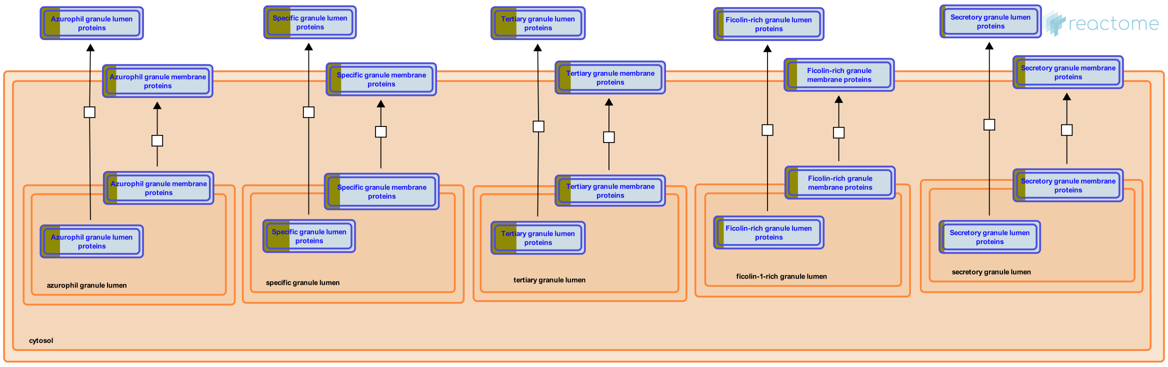

Neutrophils are the most abundant leukocytes (white blood cells), indispensable in defending the body against invading microorganisms. In response to infection, neutrophils leave the circulation and migrate towards the inflammatory focus. They contain several subsets of granules that are mobilized to fuse with the cell membrane or phagosomal membrane, resulting in the exocytosis or exposure of membrane proteins. Traditionally, neutrophil granule constituents are described as antimicrobial or proteolytic, but granules also introduce membrane proteins to the cell surface, changing how the neutrophil responds to its environment (Borregaard et al. 2007). Primed neutrophils actively secrete cytokines and other inflammatory mediators and can present antigens via MHC II, stimulating T-cells (Wright et al. 2010).

Granules form during neutrophil differentiation. Granule subtypes can be distinguished by their content but overlap in structure and composition. The differences are believed to be a consequence of changing protein expression and differential timing of granule formation during the terminal processes of neutrophil differentiation, rather than sorting (Le Cabec et al. 1996).

The classical granule subsets are Azurophil or primary granules (AG), secondary granules (SG) and gelatinase granules (GG). Neutrophils also contain exocytosable storage cell organelles, storage vesicles (SV), formed by endocytosis they contain many cell-surface markers and extracellular, plasma proteins (Borregaard et al. 1992). Ficolin-1-rich granules (FG) are like GGs highly exocytosable but gelatinase-poor (Rorvig et al. 2009).

References

Wright HL, Moots RJ, Bucknall RC & Edwards SW (2010). Neutrophil function in inflammation and inflammatory diseases. Rheumatology (Oxford), 49, 1618-31.

Borregaard N, Sørensen OE & Theilgaard-Mönch K (2007). Neutrophil granules: a library of innate immunity proteins. Trends Immunol., 28, 340-5.

Rørvig S, Østergaard O, Heegaard NH & Borregaard N (2013). Proteome profiling of human neutrophil granule subsets, secretory vesicles, and cell membrane: correlation with transcriptome profiling of neutrophil precursors. J. Leukoc. Biol., 94, 711-21.

Borregaard N, Kjeldsen L, Rygaard K, Bastholm L, Nielsen MH, Sengeløv H, ... Johnsen AH (1992). Stimulus-dependent secretion of plasma proteins from human neutrophils. J. Clin. Invest., 90, 86-96.

Le Cabec V, Cowland JB, Calafat J & Borregaard N (1996). Targeting of proteins to granule subsets is determined by timing and not by sorting: The specific granule protein NGAL is localized to azurophil granules when expressed in HL-60 cells. Proc. Natl. Acad. Sci. U.S.A., 93, 6454-7.

Edit history

Date	Action	Author
2015-09-21	Authored	Jupe S
2015-09-21	Created	Jupe S
2016-06-13	Edited	Jupe S
2016-06-13	Reviewed	Heegaard N
2019-11-15	Modified	Weiser D

Entities found in this pathway (66)

Input	UniProt Id	Input	UniProt Id	Input	UniProt Id
A1AG1_HUMAN	P02763	A1AG2_HUMAN	P19652	A1AT_HUMAN	P01009
A1BG_HUMAN	P04217	A2GL_HUMAN	P02750	AACT_HUMAN	P01011
ADA10_HUMAN	O14672	ADA2_HUMAN	Q9NZK5	ADAM8_HUMAN	P78325
AGAL_HUMAN	P06280	AMPN_HUMAN	P15144	ASAH1_HUMAN	Q13510
AT11B_HUMAN	Q9Y2G3	BGLR_HUMAN	P08236	BST1_HUMAN	Q10588
CANT1_HUMAN	Q8WVQ1	CAP7_HUMAN	P20160	CATC_HUMAN	P53634
CATZ_HUMAN	Q9UBR2	CD14_HUMAN	P08571	CD177_HUMAN	Q8N6Q3
CD36_HUMAN	P16671	CD44_HUMAN	P16070	CD97_HUMAN	P48960
CEAM1_HUMAN	P13688	CEAM8_HUMAN	P31997	CF120_HUMAN	Q7Z4R8
CO3_HUMAN	P01024	CRIS3_HUMAN	P54108	DSG1_HUMAN	Q02413
ELNE_HUMAN	P08246	FETUA_HUMAN	P02765	FGL2_HUMAN	Q14314
FILA2_HUMAN	Q5D862	FOLR3_HUMAN	P41439	FUCO_HUMAN	P04066
GRN_HUMAN	P28799	HPSE_HUMAN	Q9Y251	HPT_HUMAN	P00738
ITAM_HUMAN	P11215	ITB2_HUMAN	P05107	K2C1_HUMAN	P04264
LAMP1_HUMAN	P11279	LAMP2_HUMAN	P13473	LIRA3_HUMAN	Q8N6C8
LYAM1_HUMAN	P14151	MGA_HUMAN	O43451	MPRI_HUMAN	P11717
NGAL_HUMAN	P80188	OLFM4_HUMAN	Q6UX06	OSCAR_HUMAN	Q8IYS5
PERM_HUMAN	P05164	PIGR_HUMAN	P01833	PROP_HUMAN	P27918
PTPRB_HUMAN	P23467	PTPRC_HUMAN	P08575	PTPRJ_HUMAN	Q12913
QPCT_HUMAN	Q16769	QSOX1_HUMAN	O00391	RNAS2_HUMAN	P10153
RNT2_HUMAN	O00584	SAP_HUMAN	P07602	SIRB1_HUMAN	O00241
TRFL_HUMAN	P02788	TTHY_HUMAN	P02766	VNN1_HUMAN	O95497

14. Intrinsic Pathway of Fibrin Clot Formation (R-HSA-140837)

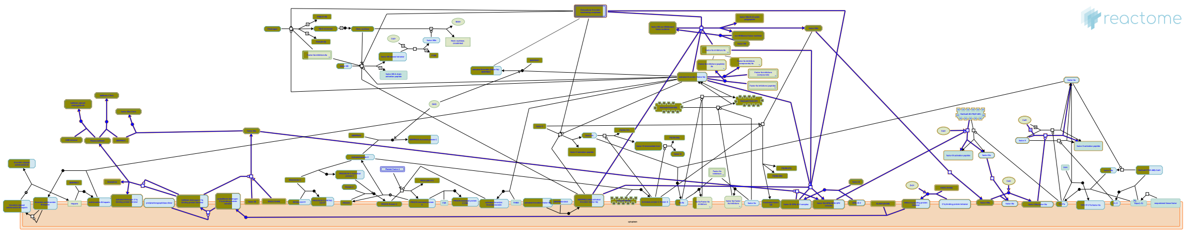

Cellular compartments: extracellular region.

The intrinsic pathway of blood clotting connects interactions among kininogen (high molecular weight kininogen, HK), prekallikrein (PK), and factor XII to the activation of clotting factor X by a series of reactions that is independent of the extrinsic pathway and that is not subject to inhibition by TFPI. It is thus essential for the prolongation of the clotting cascade: while the reactions of the extrinsic pathway appear to be sufficient to initiate clot formation, those of the intrinsic pathway are required to maintain it (Broze 1995; Davie et al. 1991; Monroe et al. 2002). The intrinsic pathway can be divided into three parts: 1) reactions involving interactions of kininogen, prekallikrein, and factor XII, leading to the activation of factor XII, 2) reactions involving factor XI, factor IX, factor VIII, and von Willebrand factor (vWF) leading to the activation of factors VIII and IX, and 3) reactions that inactivate factor XIIa and kallikrein.

Kininogen, prekallikrein, and factor XII were first identified as proteins needed for the rapid formation of clots when whole blood is exposed to negatively charged surfaces in vitro. Studies in vitro have identified several possible sets of interactions, in which small quantities of one or more of these proteins 'autoactivate' and then catalyze the formation of larger quantities of activated factors. Recent work, however, suggests that these factors form complexes on endothelial cell surfaces mediated by C1q binding protein (C1q bp), that the first activation event is the cleavage of prekallikrein by prolylcarboxypeptidase, and that the resulting kallikrein catalyzes the activation of factor XII (Schmaier 2004).

The second group of events, occurs in vivo on the surfaces of activated platelets (although most biochemical characterization of the reactions was originally done with purified proteins in solution). Factor XI binds to the platelet glycoprotein (GP) Ib:IX:V complex, where it can be activated by cleavage either by thrombin (generated by reactions of the common pathway) or by activated factor XII (generated in the first part of the intrinsic pathway). Activated factor XI in turn catalyzes the activation of factor IX. Simultaneously, factor VIII, complexed with vWF, is cleaved by thrombin, activating it and causing its release from vWF. Activated factors VIII and IX form a complex on the platelet surface that very efficiently converts factor X to activated factor X. (Activated factors X and V then form a complex that efficiently activates thrombin.)

While these two groups of events can be viewed as forming a single functional pathway (e.g., Davie et al. 1991), human clinical genetic data cast doubt on this view. Individuals deficient in kininogen, prekallikrein, or factor XII proteins exhibit normal blood clot formation in vivo. In contrast, deficiencies of factor XI can be associated with failure of blood clotting under some conditions, and deficiencies of vWF, factor VIII, or factor IX cause severe abnormalities - von Willebrand disease, hemophilia A, and hemophilia B, respectively. These data suggest that while the second group of events is essential for normal clot formation in vivo, the first group has a different function (e.g., Schmaier 2004).

Finally, reactions neutralize proteins activated in the first part of the intrinsic pathway. Kallikrein forms stable complexes with either C1 inhibitor (C1Inh) or with alpha2-macroglobulin, and factor XIIa forms stable complexes with C1Inh. The relevance of these neutralization events to the regulation of blood clotting is unclear, however. The physiological abnormalities observed in individuals who lack C1Inh appear to be due entirely to abnormalities of complement activation; blood clotting appears to proceed normally. This observation is consistent with the hypothesis, above, that factor XIIa plays a limited role in normal blood clotting under physiological conditions.

These events are outlined in the drawing: black arrows connect the substrates (inputs) and products (outputs) of individual reactions; blue lines connect activated enzymes to the reactions they catalyze.

References

Broze GJ Jr (1995). Tissue factor pathway inhibitor and the revised theory of coagulation. Annu Rev Med, 46, 103-12.

Davie EW, Fujikawa K & Kisiel W (1991). The coagulation cascade: initiation, maintenance, and regulation. Biochemistry, 30, 10363-70.

Monroe DM, Hoffman M & Roberts HR (2002). Platelets and thrombin generation. Arterioscler Thromb Vasc Biol, 22, 1381-9.

Schmaier AH (2004). The physiologic basis of assembly and activation of the plasma kallikrein/kinin system. Thromb Haemost, 91, 1-3.

Edit history

Date	Action	Author
2004-08-24	Authored	D'Eustachio P
2004-08-24	Created	D'Eustachio P
2008-01-11	Reviewed	Rush MG
2019-11-13	Edited	D'Eustachio P
2019-11-21	Modified	Weiser D

Entities found in this pathway (16)

Input	UniProt Id	Input	UniProt Id	Input	UniProt Id
A2MG_HUMAN	P01023	ANT3_HUMAN	P01008	FA11_HUMAN	P03951
FA12_HUMAN	P00748	FA8_HUMAN	P00451	GDN_HUMAN	P07093
GP1BA_HUMAN	P07359	GPV_HUMAN	P40197	HEP2_HUMAN	P05546
IC1_HUMAN	P05155	IPSP_HUMAN	P05154	KLKB1_HUMAN	P03952
KNG1_HUMAN	P01042	PROC_HUMAN	P04070	THRB_HUMAN	P00734
VWF_HUMAN	P04275				

15. Innate Immune System (R-HSA-168249)

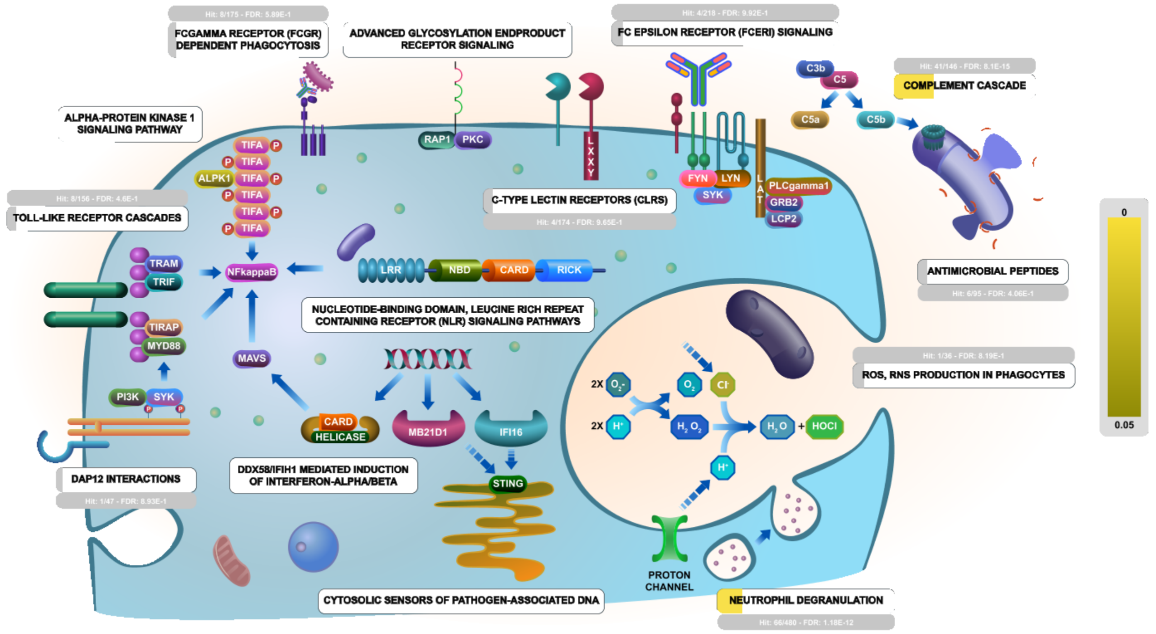

Innate immunity encompases the nonspecific part of immunity tha are part of an individual's natural biologic makeup

References

Edit history

Date	Action	Author
2005-11-12	Created	Gillespie ME
2019-11-15	Modified	Weiser D

Entities found in this pathway (116)

Input	UniProt Id	Input	UniProt Id	Input	UniProt Id
A1AG1_HUMAN	P02763	A1AG2_HUMAN	P19652	A1AT_HUMAN	P01009
A1BG_HUMAN	P04217	A2GL_HUMAN	P02750	AACT_HUMAN	P01011
ADA10_HUMAN	O14672	ADA2_HUMAN	Q9NZK5	ADAM8_HUMAN	P78325
AGAL_HUMAN	P06280	AMPN_HUMAN	P15144	APOB_HUMAN	P04114
ASAH1_HUMAN	Q13510	AT11B_HUMAN	Q9Y2G3	BGLR_HUMAN	P08236
BPIB1_HUMAN	Q8TDL5	BST1_HUMAN	Q10588	C1QA_HUMAN	P02745
C1QC_HUMAN	P02747	C1R_HUMAN	P00736	C1S_HUMAN	P09871
C4BPA_HUMAN	P04003	C4BPB_HUMAN	P20851	CANT1_HUMAN	Q8WVQ1
CAP7_HUMAN	P20160	CATC_HUMAN	P53634	CATZ_HUMAN	Q9UBR2
CBPB2_HUMAN	Q96IY4	CD14_HUMAN	P08571	CD177_HUMAN	Q8N6Q3
CD36_HUMAN	P16671	CD44_HUMAN	P16070	CD97_HUMAN	P48960
CEAM1_HUMAN	P13688	CEAM8_HUMAN	P31997	CF120_HUMAN	Q7Z4R8
CFAB_HUMAN	P00751	CFAH_HUMAN	P08603	CFAI_HUMAN	P05156
CLUS_HUMAN	P10909	CO2_HUMAN	P06681	CO3_HUMAN	P01024
CO4A_HUMAN	P0C0L4	CO5_HUMAN	P01031	CO6_HUMAN	P13671
CO8A_HUMAN	P07357	CO8B_HUMAN	P07358	CO9_HUMAN	P02748
CPN2_HUMAN	P22792	CR2_HUMAN	P20023	CRIS3_HUMAN	P54108

Input	UniProt Id	Input	UniProt Id	Input	UniProt Id
DSG1_HUMAN	Q02413	ELNE_HUMAN	P08246	FCN3_HUMAN	O75636
FETUA_HUMAN	P02765	FGL2_HUMAN	Q14314	FHR1_HUMAN	Q03591
FHR2_HUMAN	P36980	FHR3_HUMAN	Q02985	FHR4_HUMAN	Q92496
FHR5_HUMAN	Q9BXR6	FIBA_HUMAN	P02671	FIBB_HUMAN	P02675
FIBG_HUMAN	P02679	FILA2_HUMAN	Q5D862	FOLR3_HUMAN	P41439
FUCO_HUMAN	P04066	GRN_HUMAN	P28799	HPSE_HUMAN	Q9Y251
HPT_HUMAN	P00738	HV434_HUMAN	P06331	IC1_HUMAN	P05155
ICAM2_HUMAN	P13598	ICAM3_HUMAN	P32942	IGHE_HUMAN	P01854
IGHG1_HUMAN	P01857	IGHG2_HUMAN	P01859	IGHG3_HUMAN	P01860
IGHG4_HUMAN	P01861	ITAM_HUMAN	P11215	ITB2_HUMAN	P05107
ITPR2_HUMAN	Q14571	K2C1_HUMAN	P04264	KV502_HUMAN	P06315
LAMP1_HUMAN	P11279	LAMP2_HUMAN	P13473	LIRA3_HUMAN	Q8N6C8
LYAM1_HUMAN	P14151	MASP1_HUMAN	P48740	MASP2_HUMAN	O00187-1
MGA_HUMAN	O43451	MPRI_HUMAN	P11717	MUC16_HUMAN	Q8WXI7
NGAL_HUMAN	P80188	OLFM4_HUMAN	Q6UX06	OSCAR_HUMAN	Q8IYS5
PERM_HUMAN	P05164	PGRP2_HUMAN	Q96PD5	PIGR_HUMAN	P01833
PLD4_HUMAN	Q96BZ4	PROP_HUMAN	P27918	PROS_HUMAN	P07225
PTPRB_HUMAN	P23467	PTPRC_HUMAN	P08575	PTPRJ_HUMAN	Q12913
QPCT_HUMAN	Q16769	QSOX1_HUMAN	O00391	RNAS2_HUMAN	P10153
RNT2_HUMAN	O00584	SAP_HUMAN	P07602	SIRB1_HUMAN	O00241
THRB_HUMAN	P00734	TRFL_HUMAN	P02788	TTHY_HUMAN	P02766
VNN1_HUMAN	O95497	VTNC_HUMAN	P04004		

16. Common Pathway of Fibrin Clot Formation (R-HSA-140875)

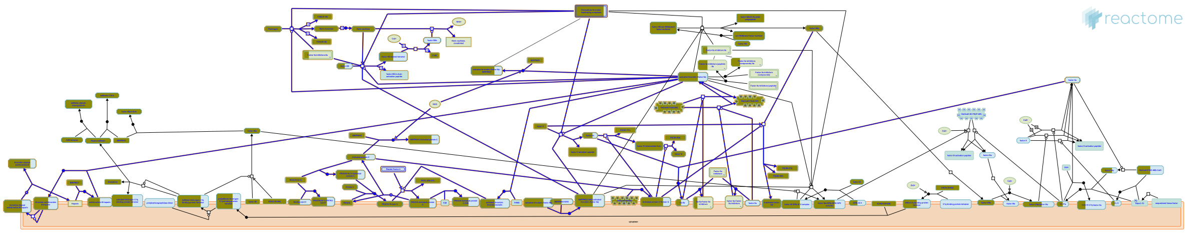

Cellular compartments: extracellular region.

The common pathway consists of the cascade of activation events leading from the formation of activated factor X to the formation of active thrombin, the cleavage of fibrinogen by thrombin, and the formation of cleaved fibrin into a stable multimeric, cross-linked complex. Thrombin also efficiently catalyzes the activation of several factors required earlier in the clotting cascade, thus acting in effect as a positive regulator of clotting. At the same time, thrombin activates protein C, which in turn catalyzes the inactivation of several of these upstream factors, thereby limiting the clotting process. Thrombin can be trapped in stable, inactive complexes with: antithrombin-III (SERPINC1), a circulating blood protein; heparin cofactor II (SERPIND1) which inhibits thrombin in a dermatan sulfate–dependent manner in the arterial vasculature; protein C inhibitor (SERPINA5) that inhibits thrombin in complex with thrombomodulin; and Protease nexin-1 (SERPINE2) that inhibits thrombin at the vessel wall and platelet surface.

The quantitative interplay among these positive and negative modulators is critical to the normal regulation of clotting, facilitating the rapid formation of a protective clot at the site of injury, while limiting and physically confining the process.

These events are outlined in the drawing: black arrows connect the substrates (inputs) and products (outputs) of individual reactions, and blue lines connect output activated enzymes to the other reactions that they catalyze.

References

Davie EW, Fujikawa K & Kisiel W (1991). The coagulation cascade: initiation, maintenance, and regulation. Biochemistry, 30, 10363-70.

Edit history

Date	Action	Author
2004-08-24	Authored	D'Eustachio P
2004-08-24	Created	D'Eustachio P
2019-11-21	Modified	Weiser D

Entities found in this pathway (15)

Input	UniProt Id	Input	UniProt Id	Input	UniProt Id
ANT3_HUMAN	P01008	CD177_HUMAN	Q8N6Q3	EPCR_HUMAN	Q9UNN8
F13B_HUMAN	P05160	FA5_HUMAN	P12259	FA8_HUMAN	P00451
FIBA_HUMAN	P02671	FIBB_HUMAN	P02675	FIBG_HUMAN	P02679
GDN_HUMAN	P07093	HEP2_HUMAN	P05546	IPSP_HUMAN	P05154
PROC_HUMAN	P04070	PROS_HUMAN	P07225	THRB_HUMAN	P00734

17. Immune System (R-HSA-168256)

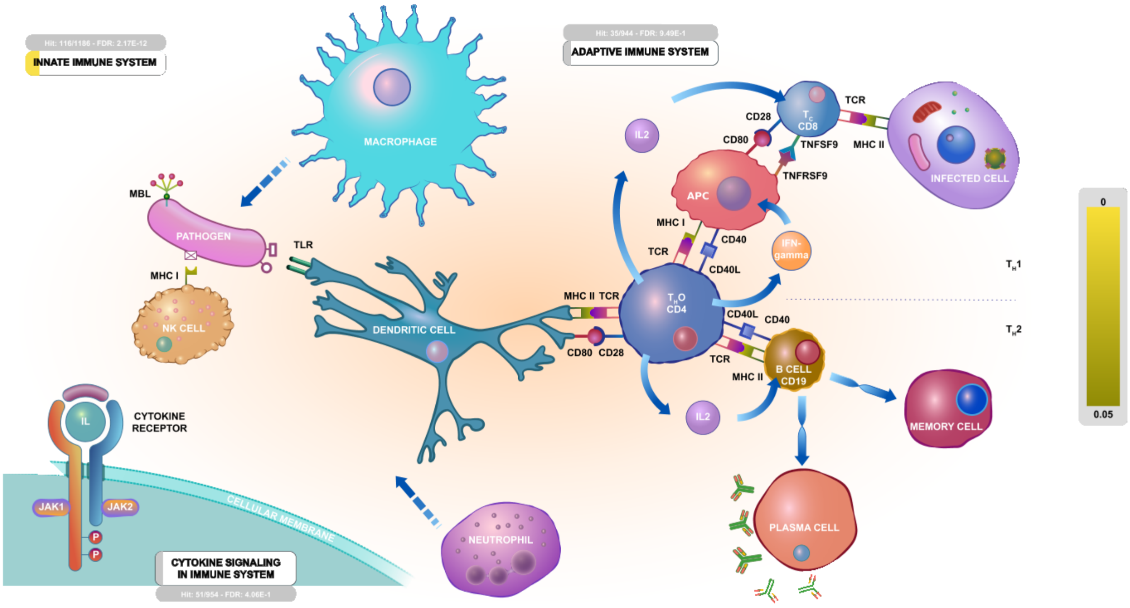

Humans are exposed to millions of potential pathogens daily, through contact, ingestion, and inhalation. Our ability to avoid infection depends on the adaptive immune system and during the first critical hours and days of exposure to a new pathogen, our innate immune system.

References

Edit history

Date	Action	Author
2005-11-12	Created	Gillespie ME
2006-03-30	Authored	Luo F, Ouwehand WH, Gillespie ME, de Bono B
2006-04-19	Reviewed	Zwaginga JJ, D'Eustachio P, Gay NJ, Gale M Jr
2019-11-15	Modified	Weiser D

Entities found in this pathway (164)

Input	UniProt Id	Input	UniProt Id	Input	UniProt Id
1B82_HUMAN	Q29718	1C15_HUMAN	Q07000	1C17_HUMAN	Q95604
A1AG1_HUMAN	P02763	A1AG2_HUMAN	P19652	A1AT_HUMAN	P01009
A1BG_HUMAN	P04217	A2GL_HUMAN	P02750	AACT_HUMAN	P01011
ADA10_HUMAN	O14672	ADA2_HUMAN	Q9NZK5	ADAM8_HUMAN	P78325
AGAL_HUMAN	P06280	AMPN_HUMAN	P15144	ANGP1_HUMAN	Q15389
APOB_HUMAN	P04114	ASAH1_HUMAN	Q13510	AT11B_HUMAN	Q9Y2G3
BGLR_HUMAN	P08236	BPIB1_HUMAN	Q8TDL5	BST1_HUMAN	Q10588
BT2A1_HUMAN	Q7KYR7	C1QA_HUMAN	P02745	C1QC_HUMAN	P02747
C1R_HUMAN	P00736	C1S_HUMAN	P09871	C4BPA_HUMAN	P04003
C4BPB_HUMAN	P20851	CANT1_HUMAN	Q8WVQ1	CAP7_HUMAN	P20160
CATC_HUMAN	P53634	CATF_HUMAN	Q9UBX1	CATZ_HUMAN	Q9UBR2
CBPB2_HUMAN	Q96IY4	CD14_HUMAN	P08571	CD177_HUMAN	Q8N6Q3
CD34_HUMAN	P28906-1	CD36_HUMAN	P16671	CD44_HUMAN	P16070

Input	UniProt Id	Input	UniProt Id	Input	UniProt Id
CD97_HUMAN	P48960	CEAM1_HUMAN	P13688	CEAM8_HUMAN	P31997
CF120_HUMAN	Q7Z4R8	CFAB_HUMAN	P00751	CFAH_HUMAN	P08603
CFAI_HUMAN	P05156	CLUS_HUMAN	P10909	CO2_HUMAN	P06681
CO3_HUMAN	P01024	CO4A_HUMAN	P0C0L4	CO5_HUMAN	P01031
CO6_HUMAN	P13671	CO8A_HUMAN	P07357	CO8B_HUMAN	P07358
CO9_HUMAN	P02748	CPN2_HUMAN	P22792	CR2_HUMAN	P20023
CRIS3_HUMAN	P54108	CSF1R_HUMAN	P07333	DSG1_HUMAN	Q02413
ELNE_HUMAN	P08246	ERAP2_HUMAN	Q6P179	FCN3_HUMAN	O75636
FETUA_HUMAN	P02765	FGFR1_HUMAN	P11362-1, P11362-19	FGFR3_HUMAN	P22607-1, P22607-2
FGL2_HUMAN	Q14314	FHR1_HUMAN	Q03591	FHR2_HUMAN	P36980
FHR3_HUMAN	Q02985	FHR4_HUMAN	Q92496	FHR5_HUMAN	Q9BXR6
FIBA_HUMAN	P02671	FIBB_HUMAN	P02675	FIBG_HUMAN	P02679
FILA2_HUMAN	Q5D862	FINC_HUMAN	P02751	FOLR3_HUMAN	P41439
FUCO_HUMAN	P04066	GFRA2_HUMAN	O00451	GFRA3_HUMAN	O60609
GRN_HUMAN	P28799	HG2A_HUMAN	P04233	HPSE_HUMAN	Q9Y251
HPT_HUMAN	P00738	HV434_HUMAN	P06331	I17RA_HUMAN	Q96F46
I18BP_HUMAN	O95998	IC1_HUMAN	P05155	ICAM1_HUMAN	P05362
ICAM2_HUMAN	P13598	ICAM3_HUMAN	P32942	ICOSL_HUMAN	O75144
IGHE_HUMAN	P01854	IGHG1_HUMAN	P01857	IGHG2_HUMAN	P01859
IGHG3_HUMAN	P01860	IGHG4_HUMAN	P01861	IGHM_HUMAN	P01871
IL1AP_HUMAN	Q9NPH3-1	IL1R2_HUMAN	P27930	IL6RA_HUMAN	P08887, P08887-2
IL6RB_HUMAN	P40189, P40189-1, P40189-2	IL7RA_HUMAN	P16871	ILRL1_HUMAN	Q01638, Q01638-2
ITAM_HUMAN	P11215	ITB1_HUMAN	P05556	ITB2_HUMAN	P05107
ITB3_HUMAN	P05106	ITPR2_HUMAN	Q14571	K2C1_HUMAN	P04264
KIT_HUMAN	P10721	KV502_HUMAN	P06315	LAMP1_HUMAN	P11279
LAMP2_HUMAN	P13473	LIRA3_HUMAN	Q8N6C8	LYAM1_HUMAN	P14151
MASP1_HUMAN	P48740	MASP2_HUMAN	O00187-1	MET_HUMAN	P08581
MGA_HUMAN	O43451	MPRI_HUMAN	P11717	MRC1_HUMAN	P22897
MRC2_HUMAN	Q9UBG0	MUC16_HUMAN	Q8WXI7	NCAM1_HUMAN	P13591
NGAL_HUMAN	P80188	OLFM4_HUMAN	Q6UX06	OSCAR_HUMAN	Q8IYS5
OSMR_HUMAN	Q99650	PD1L2_HUMAN	Q9BQ51	PDGFB_HUMAN	P01127
PERM_HUMAN	P05164	PGRP2_HUMAN	Q96PD5	PIGR_HUMAN	P01833
PLD4_HUMAN	Q96BZ4	PROP_HUMAN	P27918	PROS_HUMAN	P07225
PTPRB_HUMAN	P23467	PTPRC_HUMAN	P08575	PTPRJ_HUMAN	Q12913
PTPRZ_HUMAN	P23471	PVR_HUMAN	P15151	QPCT_HUMAN	Q16769
QSOX1_HUMAN	O00391	RNAS2_HUMAN	P10153	RNT2_HUMAN	O00584
SAP_HUMAN	P07602	SIRB1_HUMAN	O00241	TBA1C_HUMAN	Q9BQE3
TBB2A_HUMAN	Q13885	TGFB1_HUMAN	P01137	THRB_HUMAN	P00734
TIE2_HUMAN	Q02763	TIMP1_HUMAN	P01033	TLN1_HUMAN	Q9Y490
TNR11_HUMAN	Q9Y6Q6	TRFL_HUMAN	P02788	TRML2_HUMAN	Q5T2D2
TTHY_HUMAN	P02766	VCAM1_HUMAN	P19320	VNN1_HUMAN	O95497
VTNC_HUMAN	P04004	VWF_HUMAN	P04275		

18. Non-integrin membrane-ECM interactions (R-HSA-3000171)

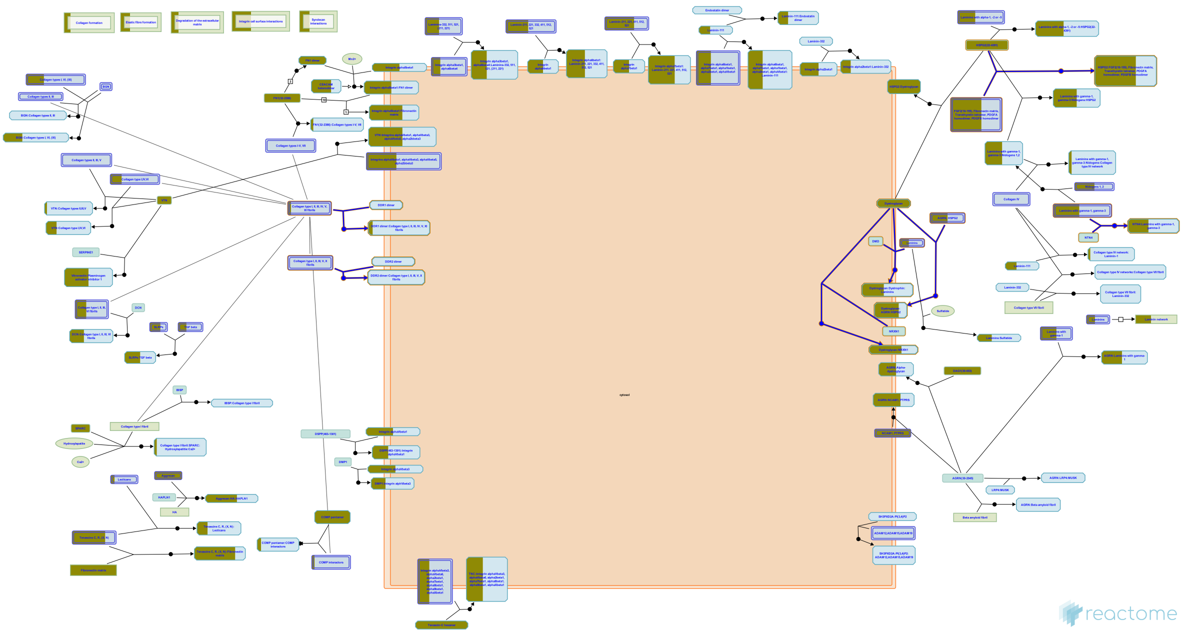

Several non-integrin membrane proteins interact with extracellular matrix proteins. Transmembrane proteoglycans may associate with integrins and growth factor receptors to influence their function, or they can signal independently, often influencing the actin cytoskeleton.

References

Rosso F, Giordano A, Barbarisi M & Barbarisi A (2004). From cell-ECM interactions to tissue engineering. J. Cell. Physiol., 199, 174-80.

Couchman JR (2010). Transmembrane signaling proteoglycans. Annu. Rev. Cell Dev. Biol., 26, 89-114.

Edit history

Date	Action	Author
2012-07-31	Authored	Jupe S
2013-01-24	Created	Jupe S
2013-04-26	Edited	Jupe S
2013-05-22	Reviewed	Ricard-Blum S
2019-11-15	Modified	Weiser D

Entities found in this pathway (16)

Input	UniProt Id	Input	UniProt Id	Input	UniProt Id
COBA2_HUMAN	P13942	DAG1_HUMAN	Q14118	FINC_HUMAN	P02751
ITB1_HUMAN	P05556	ITB3_HUMAN	P05106	LAMA2_HUMAN	P24043
LAMA4_HUMAN	Q16363	LAMB2_HUMAN	P55268	LAMC1_HUMAN	P11047
PDGFB_HUMAN	P01127	PGBM_HUMAN	P98160	TENA_HUMAN	P24821
TGFB1_HUMAN	P01137	TSP1_HUMAN	P07996	TTHY_HUMAN	P02766
VTNC_HUMAN	P04004				

19. Cell surface interactions at the vascular wall (R-HSA-202733)

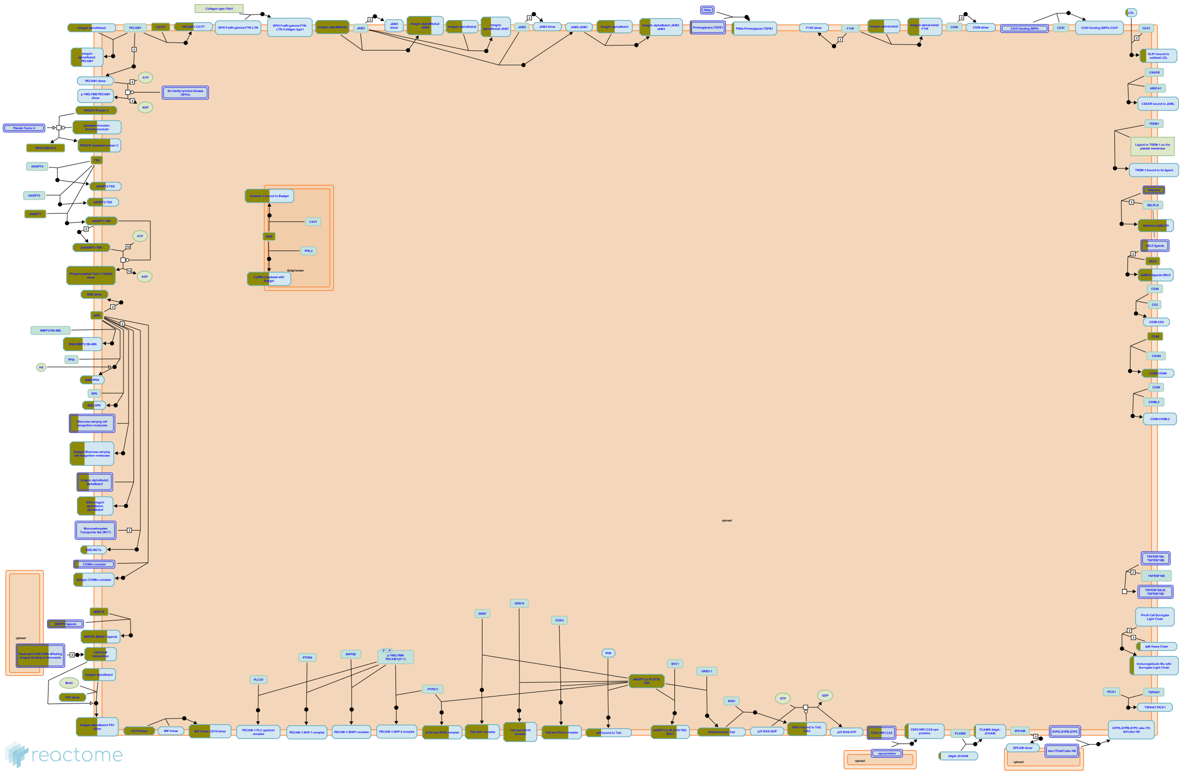

Cellular compartments: plasma membrane.

Leukocyte extravasation is a rigorously controlled process that guides white cell movement from the vascular lumen to sites of tissue inflammation. The powerful adhesive interactions that are required for leukocytes to withstand local flow at the vessel wall is a multistep process mediated by different adhesion molecules. Platelets adhered to injured vessel walls form strong adhesive substrates for leukocytes. For instance, the initial tethering and rolling of leukocytes over the site of injury are mediated by reversible binding of selectins to their cognate cell-surface glycoconjugates.

Endothelial cells are tightly connected through various proteins, which regulate the organization of the junctional complex and bind to cytoskeletal proteins or cytoplasmic interaction partners that allow the transfer of intracellular signals. An important role for these junctional proteins in governing the transendothelial migration of leukocytes under normal or inflammatory conditions has been established.

This pathway describes some of the key interactions that assist in the process of platelet and leukocyte interaction with the endothelium, in response to injury.

References

Jackson SP, Mistry N & Yuan Y (2000). Platelets and the injured vessel wall-- "rolling into action": focus on glycoprotein Ib/V/IX and the platelet cytoskeleton. Trends Cardiovasc Med, 10, 192-7.

Schober A & Weber C (2005). Mechanisms of monocyte recruitment in vascular repair after injury. Antioxid Redox Signal, 7, 1249-57.

Becker BF, Heindl B, Kupatt C & Zahler S (2000). Endothelial function and hemostasis. Z Kardiol, 89, 160-7.

Weber C, Fraemohs L & Dejana E (2007). The role of junctional adhesion molecules in vascular inflammation. Nat Rev Immunol, 7, 467-77.

da Costa Martins P, van den Berk N, Ulfman LH, Koenderman L, Hordijk PL & Zwaginga JJ (2004). Platelet-monocyte complexes support monocyte adhesion to endothelium by enhancing secondary tethering and cluster formation. Arterioscler Thromb Vasc Biol, 24, 193-9.

Edit history

Date	Action	Author
2007-11-11	Created	de Bono B
2007-11-12	Reviewed	Zwaginga JJ
2007-11-12	Authored	Ouwehand WH
2013-11-20	Revised	Shamovsky V
2016-09-05	Revised	Meldal BH
2019-11-15	Modified	Weiser D

Entities found in this pathway (34)

Input	UniProt Id	Input	UniProt Id	Input	UniProt Id
4F2_HUMAN	P08195	ANGP1_HUMAN	Q15389	APOB_HUMAN	P04114
BASI_HUMAN	P35613	CD177_HUMAN	Q8N6Q3	CD44_HUMAN	P16070
CD48_HUMAN	P09326	CEAM1_HUMAN	P13688	CEAM5_HUMAN	P06731
CEAM8_HUMAN	P31997	EPCR_HUMAN	Q9UNN8	ESAM_HUMAN	Q96AP7
FINC_HUMAN	P02751	HG2A_HUMAN	P04233	HV434_HUMAN	P06331
IGHA1_HUMAN	P01876	IGHA2_HUMAN	P01877	IGHM_HUMAN	P01871
IGJ_HUMAN	P01591	ITAM_HUMAN	P11215	ITB1_HUMAN	P05556
ITB2_HUMAN	P05107	ITB3_HUMAN	P05106	KV502_HUMAN	P06315
L1CAM_HUMAN	P32004	LYAM1_HUMAN	P14151	LYAM2_HUMAN	P16581
LYAM3_HUMAN	P16109	MERTK_HUMAN	Q12866	PROC_HUMAN	P04070
PROS_HUMAN	P07225	TGFB1_HUMAN	P01137	THRB_HUMAN	P00734
TIE2_HUMAN	Q02763				

20. Activation of C3 and C5 (R-HSA-174577)

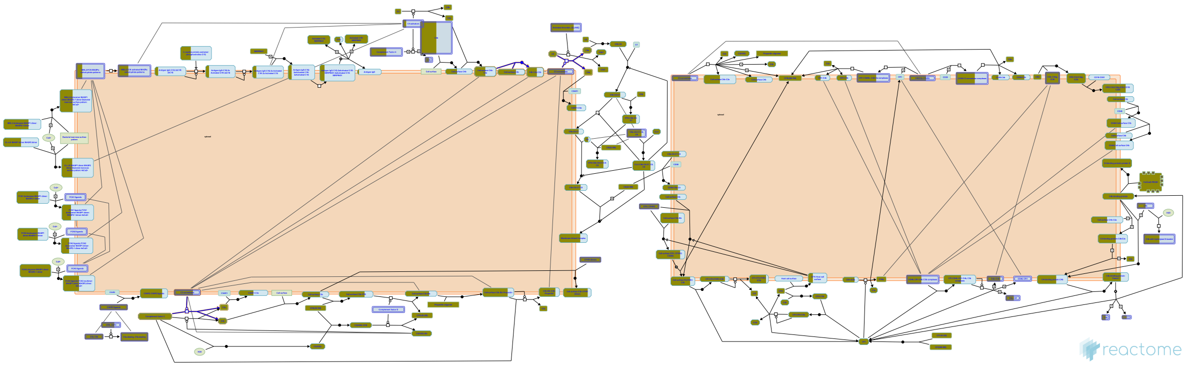

Cellular compartments: extracellular region, plasma membrane.

The 3 pathways of complement activation converge on the cleavage of C3 by C3 convertases. C3 convertase cleaves C3 into C3a and C3b - a central step of complement activation. C3a remains in the fluid phase and acts as an anaphylatoxin, whereas C3b can form additional C3 convertases hastening the production of C3b. Besides, C3b binds to C3 convertases to form C5 convertase, which can act as an opsonin, or is degraded into fragments which cannot form an active convertase.

References

Lesavre PH, Hugli TE, Esser AF & Muller-Eberhard HJ (1979). The alternative pathway C3/C5 convertase: chemical basis of factor B activation. J Immunol, 123, 529-34.

Fujita T, Matsushita M & Endo Y (2004). The lectin-complement pathway--its role in innate immunity and evolution. Immunol Rev, 198, 185-202.

Edit history

Date	Action	Author
2004-08-04	Authored	de Bono B
2006-02-20	Created	de Bono B
2006-07-04	Reviewed	D'Eustachio P
2010-11-17	Edited	Jupe S
2019-11-21	Modified	Weiser D

Entities found in this pathway (6)

Input	UniProt Id	Input	UniProt Id	Input	UniProt Id
CFAB_HUMAN	P00751	CO2_HUMAN	P06681	CO3_HUMAN	P01024
CO4A_HUMAN	P0C0L4	CO5_HUMAN	P01031	PROP_HUMAN	P27918

21. Terminal pathway of complement (R-HSA-166665)

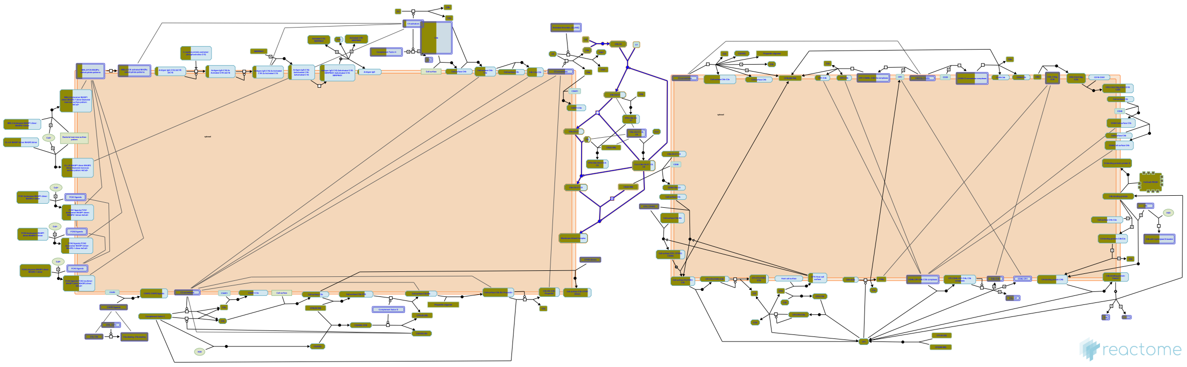

After cleavage of C5, C5b undergoes conformational changes and exposes a binding site for C6. C5b6 binds C7 resulting in the exposure of membrane binding sites and incorporation into target membranes. The membrane-bound C5b-7 complex can then bind C8. C5b-8 acts as a polymerizing agent for C9. The first C9 bound to C5b-8 undergoes major structural changes enabling formation of an elongated molecule and allows binding of additional C9 molecules and insertion of C9 cylinders into the target membrane. The number of C9 molecules varies from 1-12 in the membrane, although polymers containing up to fifteen C9 molecules are also possible.

References

Paul W (2003). *Complement, Fundamental Immunology*, 1077-1104.

Edit history

Date	Action	Author
2004-08-04	Authored	de Bono B
2005-09-14	Created	de Bono B
2006-07-04	Reviewed	D'Eustachio P
2019-11-15	Modified	Weiser D

Entities found in this pathway (6)

Input	UniProt Id	Input	UniProt Id	Input	UniProt Id
CLUS_HUMAN	P10909	CO5_HUMAN	P01031	CO6_HUMAN	P13671
CO8A_HUMAN	P07357	CO8B_HUMAN	P07358	CO9_HUMAN	P02748

22. Post-translational modification: synthesis of GPI-anchored proteins (R-HSA-163125)

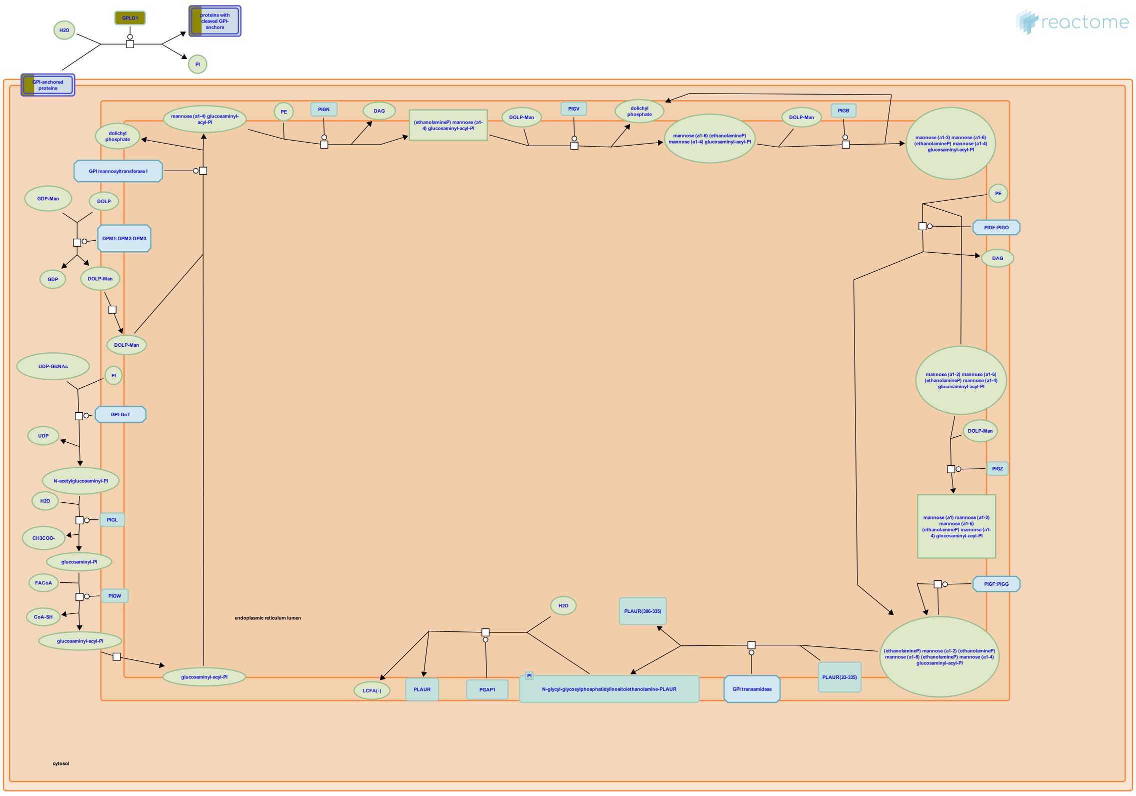

Glycosylphosphatidyl inositol (GPI) acts as a membrane anchor for many cell surface proteins. GPI is synthesized in the endoplasmic reticulum. In humans, a single pathway consisting of eleven reactions appears to be responsible for the synthesis of the major GPI species involved in membrane protein anchoring.

As a nascent protein fated to become GPI-anchored moves into the lumen of the endoplasmic reticulum, it is attacked by a transamidase complex that cleaves it near its carboxy terminus and attaches an acylated GPI moiety. The GPI moiety is deacylated, yielding a protein-GPI conjugate that can be efficiently transported to the Golgi apparatus.

References

Edit history

Date	Action	Author
2005-04-18	Created	D'Eustachio P
2019-11-15	Modified	Weiser D

Entities found in this pathway (17)

Input	UniProt Id	Input	UniProt Id	Input	UniProt Id
BST1_HUMAN	Q10588	CBPM_HUMAN	P14384	CD109_HUMAN	Q6YHK3
CEAM5_HUMAN	P06731	CNTN3_HUMAN	Q9P232	CNTN4_HUMAN	Q8IWV2
LSAMP_HUMAN	Q13449	LYPD3_HUMAN	O95274	MSLN_HUMAN	Q13421
NAR4_HUMAN	Q93070	NEGR1_HUMAN	Q7Z3B1	PHLD_HUMAN	P80108
PPBT_HUMAN	P05186	R4RL2_HUMAN	Q86UN3	RECK_HUMAN	O95980

Input	UniProt Id	Input	UniProt Id	Input	UniProt Id
VNN1_HUMAN	O95497	VNN2_HUMAN	O95498		

23. Other semaphorin interactions (R-HSA-416700)

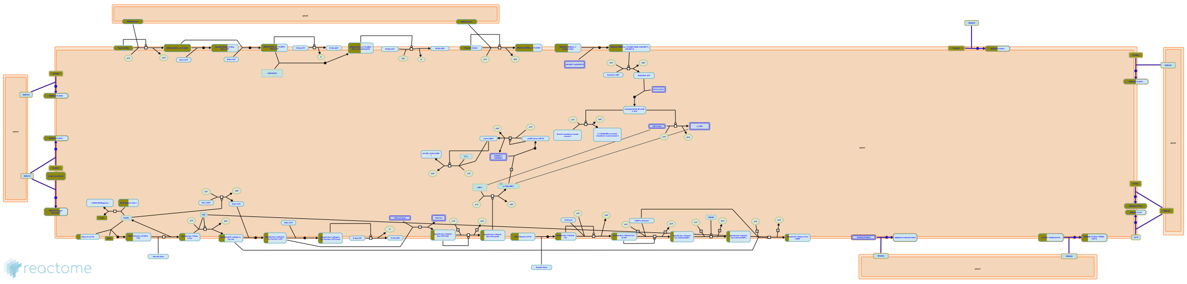

Cellular compartments: plasma membrane.

There are eight classes of semaphorins and four types of plexins. Semaphorin (SEMA) classes 1 and 2 are found in invertebrates and classes 3-7 are vertebrate sempahorines. Sempahorin class 3 is secreted, whereas the other classes are synthesised as transmembrane proteins. Vertebrate plexins (PLXNs) are classified into four subfamilies plexin-A to -D. There are four A-type plexins, three B-type, one C-type and D-type. Interactions between different subfamilies of plexins and semaphorins show differential specificity, which trigger different sets of biological functions. Another level of functional specificity specificity is attained by plexins by coupling with various coreceptors expressed in a cell- or tissue-specific manner, such as neuropilins (NRP), L1CAM, c-MET proto-oncogene, ERB2, CD72 and CD45 (Kruger et al. 2005, Law & Lee 2012).

References

Edit history

Date	Action	Author
2009-03-23	Edited	Garapati P V
2009-03-23	Authored	Garapati P V
2009-03-31	Created	Garapati P V
2009-09-02	Reviewed	Kumanogoh A, Kikutani H
2019-11-15	Modified	Weiser D

Entities found in this pathway (8)

Input	UniProt Id	Input	UniProt Id	Input	UniProt Id
ITA1_HUMAN	P56199	ITB1_HUMAN	P05556	PLXA1_HUMAN	Q9UIW2
PLXB3_HUMAN	Q9ULL4	PLXC1_HUMAN	O60486	PLXD1_HUMAN	Q9Y4D7
PTPRC_HUMAN	P08575	SEM4D_HUMAN	Q92854		

24. Initial triggering of complement (R-HSA-166663)

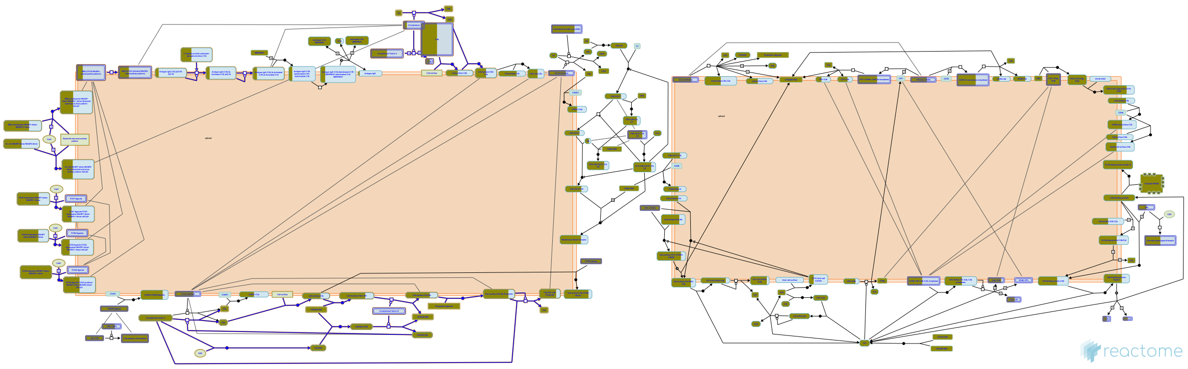

Complement activation is due to a cascade of proteolytic steps, performed by serine protease domains in some of the components. Three different pathways of activation are distinguished triggered by target-bound antibody (the classical pathway); microbial polysaccharide structures (the lectin pathway); or recognition of other "foreign" surface structures (the alternative pathway) by C3b. All three merge in the pivotal activation of C3 and, subsequently, of C5 by highly specific enzymatic complexes, the so-called C3/C5 convertases. A complement system with three C3 activation pathways and a common lytic pathway is found only in jawed vertebrates.

References

Paul W (2003). *Complement, Fundamental Immunology* , 1077-1104.

Edit history

Date	Action	Author
2004-08-04	Authored	de Bono B
2005-09-14	Created	de Bono B
2006-07-04	Reviewed	D'Eustachio P
2010-10-27	Revised	Jupe S
2010-11-17	Edited	Jupe S
2019-11-21	Modified	Weiser D

Entities found in this pathway (18)

Input	UniProt Id	Input	UniProt Id	Input	UniProt Id
C1QA_HUMAN	P02745	C1QC_HUMAN	P02747	C1R_HUMAN	P00736
C1S_HUMAN	P09871	CFAB_HUMAN	P00751	CO2_HUMAN	P06681
CO3_HUMAN	P01024	CO4A_HUMAN	P0C0L4	FCN3_HUMAN	O75636
HV434_HUMAN	P06331	IGHG1_HUMAN	P01857	IGHG2_HUMAN	P01859
IGHG3_HUMAN	P01860	IGHG4_HUMAN	P01861	KV502_HUMAN	P06315
MASP1_HUMAN	P48740	MASP2_HUMAN	O00187-1	PROP_HUMAN	P27918

25. p130Cas linkage to MAPK signaling for integrins (R-HSA-372708)

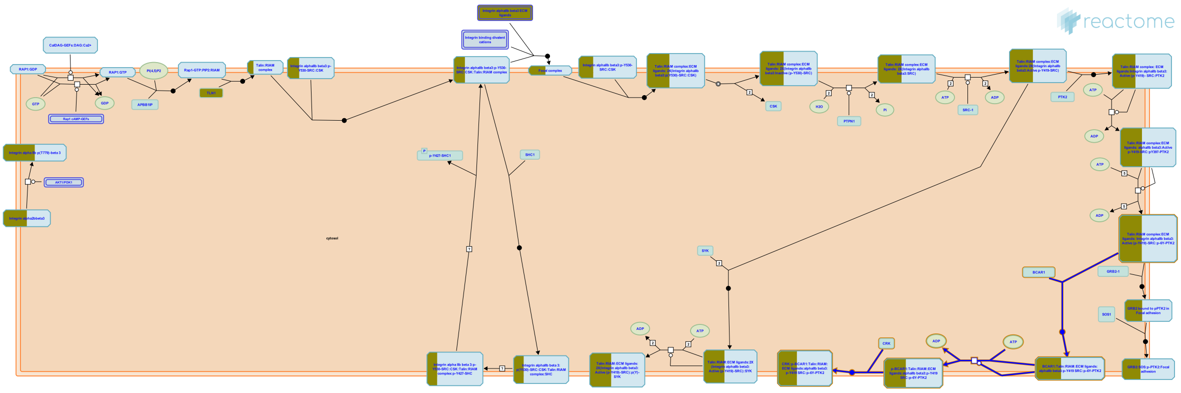

Integrin signaling is linked to the MAP kinase pathway by recruiting p130cas and Crk to the FAK/Src activation complex.

References

Defilippi P, Di Stefano P & Cabodi S (2006). p130Cas: a versatile scaffold in signaling networks. Trends Cell Biol, 16, 257-63.

Edit history

Date	Action	Author
2008-06-16	Edited	Garapati P V
2008-06-16	Authored	Garapati P V
2008-06-27	Created	Garapati P V
2008-09-16	Reviewed	Shattil SJ
2019-11-21	Modified	Weiser D

Entities found in this pathway (7)

Input	UniProt Id	Input	UniProt Id	Input	UniProt Id
FIBA_HUMAN	P02671	FIBB_HUMAN	P02675	FIBG_HUMAN	P02679
FINC_HUMAN	P02751	ITB3_HUMAN	P05106	TLN1_HUMAN	Q9Y490
VWF_HUMAN	P04275				

6. Identifiers found

Below is a list of the input identifiers that have been found or mapped to an equivalent element in Reactome, classified by resource.

Entities (404)

Input	UniProt Id	Input	UniProt Id	Input	UniProt Id
1B82_HUMAN	Q29718	1C15_HUMAN	Q07000	1C17_HUMAN	Q95604
4F2_HUMAN	P08195	A1AG1_HUMAN	P02763	A1AG2_HUMAN	P19652
A1AT_HUMAN	P01009	A1BG_HUMAN	P04217	A2AP_HUMAN	P08697
A2GL_HUMAN	P02750	A2MG_HUMAN	P01023	AACT_HUMAN	P01011
ABCA1_HUMAN	O95477	ACE_HUMAN	P12821	ADA10_HUMAN	O14672
ADA2_HUMAN	Q9NZK5	ADAM8_HUMAN	P78325	AGAL_HUMAN	P06280
AGRE2_HUMAN	Q9UHX3	AGRF5_HUMAN	Q8IZF2	ALBU_HUMAN	P02768
ALS_HUMAN	P35858	AMBP_HUMAN	P02760	AMPE_HUMAN	Q07075
AMPN_HUMAN	P15144	ANAG_HUMAN	P54802	ANGP1_HUMAN	Q15389
ANGT_HUMAN	P01019	ANT3_HUMAN	P01008	ANTR1_HUMAN	Q9H6X2-1, Q9H6X2-2
AOC3_HUMAN	Q16853	APOA_HUMAN	P08519	APOB_HUMAN	P04114
APOC4_HUMAN	P55056	APOD_HUMAN	P05090	APOF_HUMAN	Q13790
APOH_HUMAN	P02749	APOM_HUMAN	O95445	ASAH1_HUMAN	Q13510
ASAH2_HUMAN	Q9NR71	ASGR1_HUMAN	P07306	ASGR2_HUMAN	P07307
ASPN_HUMAN	Q9BXN1	AT11B_HUMAN	Q9Y2G3	AT2B2_HUMAN	Q01814
ATL2_HUMAN	Q86TH1	ATL4_HUMAN	Q6UY14	ATS13_HUMAN	Q76LX8
ATS2_HUMAN	O95450	B3GN2_HUMAN	Q9NY97	B4GA1_HUMAN	O43505
BASI_HUMAN	P35613	BGLR_HUMAN	P08236	BMP1_HUMAN	P13497
BOC_HUMAN	Q9BWV1	BPIB1_HUMAN	Q8TDL5	BST1_HUMAN	Q10588
BT2A1_HUMAN	Q7KYR7	BTD_HUMAN	P43251	C163A_HUMAN	Q86VB7
C1QA_HUMAN	P02745	C1QC_HUMAN	P02747	C1R_HUMAN	P00736
C1S_HUMAN	P09871	C4BPA_HUMAN	P04003	C4BPB_HUMAN	P20851
CA2D1_HUMAN	P54289	CAD13_HUMAN	P55290	CAD15_HUMAN	P55291
CADH2_HUMAN	P19022	CADH5_HUMAN	P33151	CADM1_HUMAN	Q9BY67
CANT1_HUMAN	Q8WVQ1	CAP7_HUMAN	P20160	CATC_HUMAN	P53634
CATF_HUMAN	Q9UBX1	CATW_HUMAN	P56202	CATZ_HUMAN	Q9UBR2
CBG_HUMAN	P08185	CBPB2_HUMAN	Q96IY4	CBPM_HUMAN	P14384
CD109_HUMAN	Q6YHK3	CD14_HUMAN	P08571	CD166_HUMAN	Q13740
CD177_HUMAN	Q8N6Q3	CD34_HUMAN	P28906-1	CD36_HUMAN	P16671
CD44_HUMAN	P16070	CD48_HUMAN	P09326	CD97_HUMAN	P48960
CEAM1_HUMAN	P13688	CEAM5_HUMAN	P06731	CEAM8_HUMAN	P31997
CERU_HUMAN	P00450	CETP_HUMAN	P11597	CF120_HUMAN	Q7Z4R8
CFAB_HUMAN	P00751	CFAH_HUMAN	P08603	CFAI_HUMAN	P05156
CHLE_HUMAN	P06276	CHSTC_HUMAN	Q9NRB3	CLUS_HUMAN	P10909
CNDD3_HUMAN	P42695	CNTN1_HUMAN	Q12860	CNTN3_HUMAN	Q9P232
CNTN4_HUMAN	Q8IWV2	CNTN6_HUMAN	Q9UQ52	CO2_HUMAN	P06681
CO3_HUMAN	P01024	CO4A_HUMAN	P0C0L4	CO5_HUMAN	P01031
CO6A2_HUMAN	P12110	CO6A3_HUMAN	P12111	CO6A6_HUMAN	A6NMZ7
CO6_HUMAN	P13671	CO8A_HUMAN	P07357	CO8B_HUMAN	P07358
CO9_HUMAN	P02748	COBA2_HUMAN	P13942	COMP_HUMAN	P49747

Input	UniProt Id	Input	UniProt Id	Input	UniProt Id
CPN2_HUMAN	P22792	CR2_HUMAN	P20023	CR3L3_HUMAN	Q68CJ9
CRHBP_HUMAN	P24387	CRIS3_HUMAN	P54108	CSF1R_HUMAN	P07333
CSPG4_HUMAN	Q6UVK1	DAG1_HUMAN	Q14118	DHI1_HUMAN	P28845
DOPO_HUMAN	P09172	DPEP2_HUMAN	Q9H4A9	DPP4_HUMAN	P27487
DSC2_HUMAN	Q02487	DSG1_HUMAN	Q02413	DSG2_HUMAN	Q14126
ECE1_HUMAN	P42892	ECM1_HUMAN	Q16610	EFNB2_HUMAN	P52799
ELNE_HUMAN	P08246	EPCR_HUMAN	Q9UNN8	ERAP2_HUMAN	Q6P179
ESAM_HUMAN	Q96AP7	F13B_HUMAN	P05160	FA11_HUMAN	P03951
FA12_HUMAN	P00748	FA20C_HUMAN	Q8IXL6	FA5_HUMAN	P12259
FA7_HUMAN	P08709	FA8_HUMAN	P00451	FBLN1_HUMAN	P23142
FBLN4_HUMAN	O95967	FBN1_HUMAN	P35555	FCN3_HUMAN	O75636
FETA_HUMAN	P02771	FETUA_HUMAN	P02765	FGFR1_HUMAN	P11362-1, P11362-19
FGFR3_HUMAN	P22607-1, P22607-2	FGL2_HUMAN	Q14314	FHR1_HUMAN	Q03591
FHR2_HUMAN	P36980	FHR3_HUMAN	Q02985	FHR4_HUMAN	Q92496
FHR5_HUMAN	Q9BXR6	FIBA_HUMAN	P02671	FIBB_HUMAN	P02675
FIBG_HUMAN	P02679	FILA2_HUMAN	Q5D862	FINC_HUMAN	P02751
FMOD_HUMAN	Q06828	FOLR3_HUMAN	P41439	FSTL1_HUMAN	Q12841
FUCO_HUMAN	P04066	G6B_HUMAN	O95866	GALT5_HUMAN	Q7Z7M9
GDF2_HUMAN	Q9UK05	GDN_HUMAN	P07093	GFRA2_HUMAN	O00451
GFRA3_HUMAN	O60609	GGT7_HUMAN	Q9UJ14	GOLM1_HUMAN	Q8NBJ4
GP1BA_HUMAN	P07359	GPR37_HUMAN	O15354	GPV_HUMAN	P40197
GRN_HUMAN	P28799	HEMO_HUMAN	P02790	HEP2_HUMAN	P05546
HEPS_HUMAN	P05981	HG2A_HUMAN	P04233	HGFA_HUMAN	Q04756
HGFL_HUMAN	P26927	HPSE_HUMAN	Q9Y251	HPT_HUMAN	P00738
HRG_HUMAN	P04196	HV434_HUMAN	P06331	HYOU1_HUMAN	Q9Y4L1
I17RA_HUMAN	Q96F46	I18BP_HUMAN	O95998	IBP3_HUMAN	P17936
IC1_HUMAN	P05155	ICAM1_HUMAN	P05362	ICAM2_HUMAN	P13598
ICAM3_HUMAN	P32942	ICOSL_HUMAN	O75144	IDS_HUMAN	P22304
IGHA1_HUMAN	P01876	IGHA2_HUMAN	P01877	IGHE_HUMAN	P01854
IGHG1_HUMAN	P01857	IGHG2_HUMAN	P01859	IGHG3_HUMAN	P01860
IGHG4_HUMAN	P01861	IGHM_HUMAN	P01871	IGJ_HUMAN	P01591
IHH_HUMAN	Q14623	IL1AP_HUMAN	Q9NPH3-1	IL1R2_HUMAN	P27930
IL6RA_HUMAN	P08887, P08887-2	IL6RB_HUMAN	P40189, P40189-1, P40189-2	IL7RA_HUMAN	P16871
ILRL1_HUMAN	Q01638, Q01638-2	IMPA3_HUMAN	Q9NX62	INHBC_HUMAN	P55103
INHBE_HUMAN	P58166	IPSP_HUMAN	P05154	ISLR_HUMAN	O14498
ITA1_HUMAN	P56199	ITA9_HUMAN	Q13797	ITAM_HUMAN	P11215
ITB1_HUMAN	P05556	ITB2_HUMAN	P05107	ITB3_HUMAN	P05106
ITIH2_HUMAN	P19823	ITIH3_HUMAN	Q06033	ITIH4_HUMAN	Q14624
ITM2B_HUMAN	Q9Y287	ITPR2_HUMAN	Q14571	K1C10_HUMAN	P13645
K2C1_HUMAN	P04264	KAIN_HUMAN	P29622	KCNE3_HUMAN	Q9Y6H6
KIT_HUMAN	P10721	KLKB1_HUMAN	P03952	KNG1_HUMAN	P01042
KV502_HUMAN	P06315	L1CAM_HUMAN	P32004	LAMA2_HUMAN	P24043
LAMA4_HUMAN	Q16363	LAMB2_HUMAN	P55268	LAMC1_HUMAN	P11047
LAMP1_HUMAN	P11279	LAMP2_HUMAN	P13473	LCAT_HUMAN	P04180
LEPR_HUMAN	P48357-1	LG3BP_HUMAN	Q08380	LIRA3_HUMAN	Q8N6C8
LMAN2_HUMAN	Q12907	LRP1_HUMAN	Q07954	LSAMP_HUMAN	Q13449
LTBP1_HUMAN	Q14766	LUM_HUMAN	P51884	LYAM1_HUMAN	P14151

Input	UniProt Id	Input	UniProt Id	Input	UniProt Id
LYAM2_HUMAN	P16581	LYAM3_HUMAN	P16109	LYOX_HUMAN	P28300
LYPD3_HUMAN	O95274	LYVE1_HUMAN	Q9Y5Y7	MA2A2_HUMAN	P49641
MA2B2_HUMAN	Q9Y2E5	MASP1_HUMAN	P48740	MASP2_HUMAN	O00187-1
MERTK_HUMAN	Q12866	MET_HUMAN	P08581	MFNG_HUMAN	O00587
MGA_HUMAN	O43451	MINP1_HUMAN	Q9UNW1	MMRN1_HUMAN	Q13201
MPRI_HUMAN	P11717	MRC1_HUMAN	P22897	MRC2_HUMAN	Q9UBG0
MSLN_HUMAN	Q13421	MSRE_HUMAN	P21757	MTMR7_HUMAN	Q9Y216
MUC16_HUMAN	Q8WXI7	NAR4_HUMAN	Q93070	NCAM1_HUMAN	P13591
NCHL1_HUMAN	O00533	NECT1_HUMAN	Q15223	NEGR1_HUMAN	Q7Z3B1
NELL2_HUMAN	Q99435	NGAL_HUMAN	P80188	NID2_HUMAN	Q14112
NMDE1_HUMAN	Q12879	NNRD_HUMAN	Q8IW45	NOTC1_HUMAN	P46531
NOTC3_HUMAN	Q9UM47	NRCAM_HUMAN	Q92823	NRP1_HUMAN	O14786
NTRK2_HUMAN	Q16620	NTRK3_HUMAN	Q16288	OLFM4_HUMAN	Q6UX06
OSCAR_HUMAN	Q8IYS5	OSMR_HUMAN	Q99650	PCOC1_HUMAN	Q15113
PCSK9_HUMAN	Q8NBP7	PD1L2_HUMAN	Q9BQ51	PDGFB_HUMAN	P01127
PDGFC_HUMAN	Q9NRA1-1	PDLI5_HUMAN	Q96HC4	PERM_HUMAN	P05164
PGBM_HUMAN	P98160	PGCA_HUMAN	P16112	PGRP2_HUMAN	Q96PD5
PHLD_HUMAN	P80108	PIGR_HUMAN	P01833	PKD1_HUMAN	P98161
PLD4_HUMAN	Q96BZ4	PLMN_HUMAN	P00747	PLTP_HUMAN	P55058-1, P55058-2
PLXA1_HUMAN	Q9UIW2	PLXB1_HUMAN	O43157	PLXB3_HUMAN	Q9ULL4
PLXC1_HUMAN	O60486	PLXD1_HUMAN	Q9Y4D7	PON1_HUMAN	P27169
PON3_HUMAN	Q15166	PPBT_HUMAN	P05186	PPIB_HUMAN	P23284
PROC_HUMAN	P04070	PROP_HUMAN	P27918	PROS_HUMAN	P07225
PROZ_HUMAN	P22891	PRP19_HUMAN	Q9UMS4	PRS23_HUMAN	O95084
PTGDS_HUMAN	P41222	PTPRB_HUMAN	P23467	PTPRC_HUMAN	P08575
PTPRF_HUMAN	P10586	PTPRJ_HUMAN	Q12913	PTPRS_HUMAN	Q13332
PTPRZ_HUMAN	P23471	PVR_HUMAN	P15151	PXDN_HUMAN	Q92626
QPCT_HUMAN	Q16769	QSOX1_HUMAN	O00391	R4RL2_HUMAN	Q86UN3
RAMP3_HUMAN	O60896	RECK_HUMAN	O95980	RELN_HUMAN	P78509
RNAS1_HUMAN	P07998	RNAS2_HUMAN	P10153	RNT2_HUMAN	O00584
S39A6_HUMAN	Q13433	S39AE_HUMAN	Q15043	SAMP_HUMAN	P02743
SAP_HUMAN	P07602	SDK2_HUMAN	Q58EX2	SEM4D_HUMAN	Q92854
SEPP1_HUMAN	P49908	SHH_HUMAN	Q15465	SIA10_HUMAN	Q9Y274
SIA8D_HUMAN	Q92187	SIRB1_HUMAN	O00241	SLIK3_HUMAN	O94933
SODE_HUMAN	P08294	SORT_HUMAN	Q99523	SPRC_HUMAN	P09486
SPRL1_HUMAN	Q14515	STAB1_HUMAN	Q9NY15	T132A_HUMAN	Q24JP5
TBA1C_HUMAN	Q9BQE3	TBB2A_HUMAN	Q13885	TENA_HUMAN	P24821
TENN_HUMAN	Q9UQP3	TENX_HUMAN	P22105	TFR1_HUMAN	P02786
TGFB1_HUMAN	P01137	TGON2_HUMAN	O43493	THRB_HUMAN	P00734
TIE2_HUMAN	Q02763	TIMP1_HUMAN	P01033	TLL1_HUMAN	O43897
TLN1_HUMAN	Q9Y490	TNR11_HUMAN	Q9Y6Q6	TNR21_HUMAN	O75509
TPST2_HUMAN	O60704	TRFE_HUMAN	P02787	TRFL_HUMAN	P02788
TRML2_HUMAN	Q5T2D2	TSP1_HUMAN	P07996	TTHY_HUMAN	P02766
TYRO_HUMAN	P14679	UFO_HUMAN	P30530	UROM_HUMAN	P07911
VCAM1_HUMAN	P19320	VGFR2_HUMAN	P35968	VGFR3_HUMAN	P35916
VNN1_HUMAN	O95497	VNN2_HUMAN	O95498	VTDB_HUMAN	P02774
VTNC_HUMAN	P04004	VWF_HUMAN	P04275	ZA2G_HUMAN	P25311
ZFYV9_HUMAN	O95405-1	ZPI_HUMAN	Q9UK55		

7. Identifiers not found

These 122 identifiers were not found neither mapped to any entity in Reactome.

A0A087WYE2_HUMAN	A0A2R8Y7U2_HUMAN	A1ATR_HUMAN	A4GAT_HUMAN	AFAM_HUMAN	AGRA2_HUMAN	AGRA3_HUMAN
AGRG6_HUMAN	AGRL4_HUMAN	ANGL6_HUMAN	APLP1_HUMAN	APMAP_HUMAN	ATF6B_HUMAN	ATRN_HUMAN
C1RL_HUMAN	CBPQ_HUMAN	CC126_HUMAN	CD276_HUMAN	CD5L_HUMAN	CDHR1_HUMAN	CDHR5_HUMAN
CL049_HUMAN	CLC14_HUMAN	CPXM2_HUMAN	CREL1_HUMAN	D42E2_HUMAN	D6RGV2_HUMAN	DCBD2_HUMAN
DIAC_HUMAN	DKK3_HUMAN	E9PJT9_HUMAN	EVI2B_HUMAN	F151A_HUMAN	F198A_HUMAN	F234A_HUMAN
FAT1_HUMAN	FCGBP_HUMAN	FCGRN_HUMAN	FETUB_HUMAN	GGTL2_HUMAN	GNPTA_HUMAN	GNPTG_HUMAN
H0Y4Y9_HUMAN	H0YD63_HUMAN	H0YDE5_HUMAN	HABP2_HUMAN	HECA2_HUMAN	HEG1_HUMAN	HMCN1_HUMAN
HV108_HUMAN	HV5X1_HUMAN	IGDC4_HUMAN	ITIH1_HUMAN	KLK10_HUMAN	LRC19_HUMAN	LRRC4_HUMAN
MEG10_HUMAN	MEGF8_HUMAN	MER34_HUMAN	MICA2_HUMAN	MST1L_HUMAN	MUC18_HUMAN	NBL1_HUMAN
NOE1_HUMAN	NOE2_HUMAN	NOMO1_HUMAN	NPTXR_HUMAN	OAF_HUMAN	OIT3_HUMAN	PCD18_HUMAN
PCYOX_HUMAN	PI16_HUMAN	PKHL1_HUMAN	PLDX1_HUMAN	PLXB2_HUMAN	PODXL_HUMAN	PPAL_HUMAN
PTK7_HUMAN	PTPRG_HUMAN	PTPRM_HUMAN	PXDC2_HUMAN	PZP_HUMAN	QSOX2_HUMAN	ROBO4_HUMAN
SE6L1_HUMAN	SE6L2_HUMAN	SEM3F_HUMAN	SEM4B_HUMAN	SEM4C_HUMAN	SEPR_HUMAN	SFRP4_HUMAN
SIAE_HUMAN	SKT_HUMAN	SRCA_HUMAN	SRTM2_HUMAN	STC1_HUMAN	SUSD1_HUMAN	SVEP1_HUMAN
TDR10_HUMAN	TEN2_HUMAN	THBG_HUMAN	TM2D3_HUMAN	TMM59_HUMAN	TXD15_HUMAN	TYRO3_HUMAN
VSTM1_HUMAN	VTM2A_HUMAN					
