# Supplementary material for: Large-scale Identification of N-linked Intact Glycopeptides in Human Serum using HILIC Enrichment and Spectral Library Search
Source: Mol Cell Proteomics. 2020 Feb 26;19(4):672–89. doi: 10.1074/mcp.RA119.001791 (PMC7124471; doi:10.1074/mcp.RA119.001791)
Supplement: Supplementary Document 3 [file 156056_1_supp_471971_q5c990.pptx]

## Slide 1
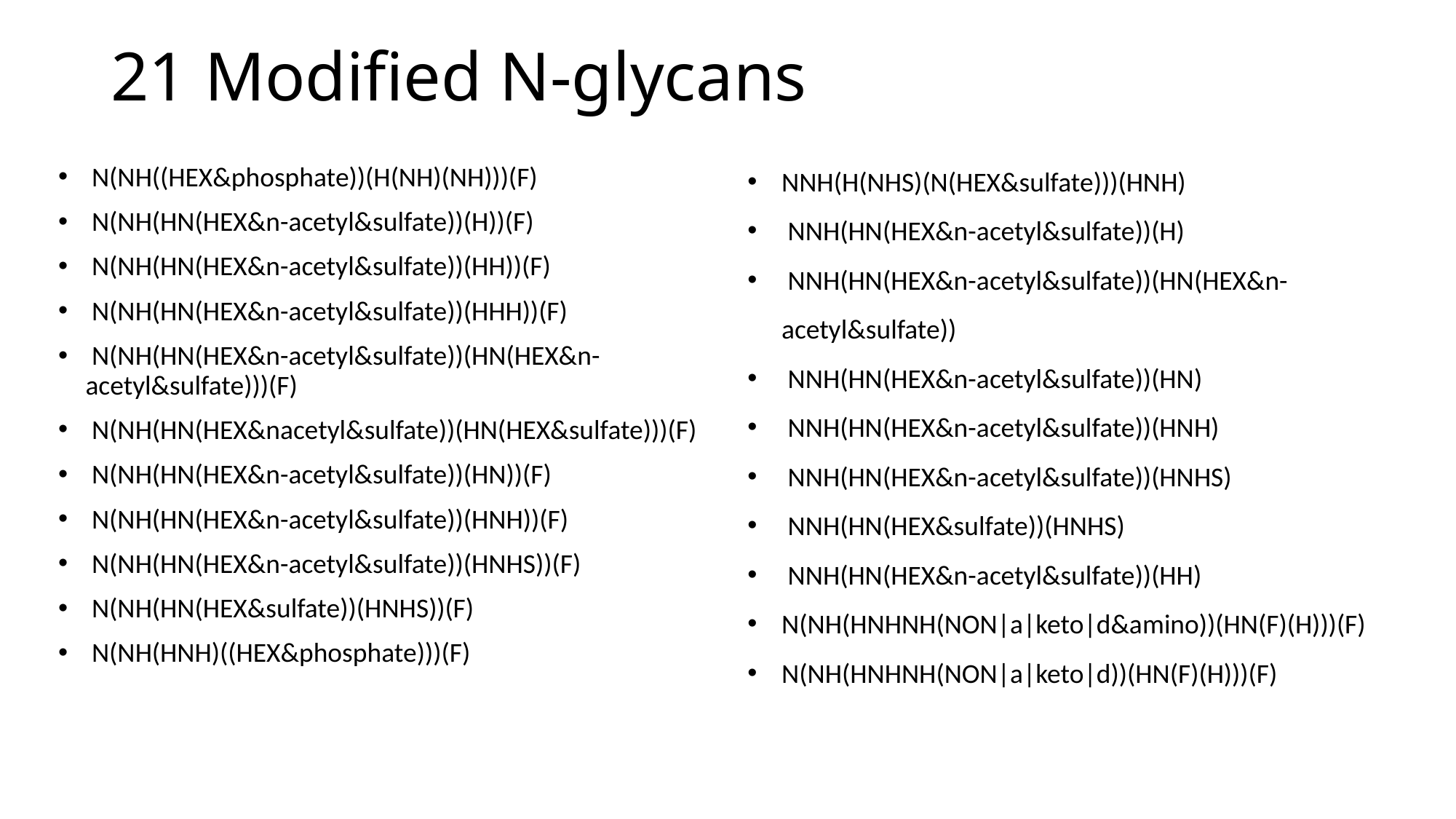

# 21 Modified N-glycans
NNH(H(NHS)(N(HEX&sulfate)))(HNH)
 NNH(HN(HEX&n-acetyl&sulfate))(H)
 NNH(HN(HEX&n-acetyl&sulfate))(HN(HEX&n-acetyl&sulfate))
 NNH(HN(HEX&n-acetyl&sulfate))(HN)
 NNH(HN(HEX&n-acetyl&sulfate))(HNH)
 NNH(HN(HEX&n-acetyl&sulfate))(HNHS)
 NNH(HN(HEX&sulfate))(HNHS)
 NNH(HN(HEX&n-acetyl&sulfate))(HH)
N(NH(HNHNH(NON|a|keto|d&amino))(HN(F)(H)))(F)
N(NH(HNHNH(NON|a|keto|d))(HN(F)(H)))(F)
 N(NH((HEX&phosphate))(H(NH)(NH)))(F)
 N(NH(HN(HEX&n-acetyl&sulfate))(H))(F)
 N(NH(HN(HEX&n-acetyl&sulfate))(HH))(F)
 N(NH(HN(HEX&n-acetyl&sulfate))(HHH))(F)
 N(NH(HN(HEX&n-acetyl&sulfate))(HN(HEX&n-acetyl&sulfate)))(F)
 N(NH(HN(HEX&nacetyl&sulfate))(HN(HEX&sulfate)))(F)
 N(NH(HN(HEX&n-acetyl&sulfate))(HN))(F)
 N(NH(HN(HEX&n-acetyl&sulfate))(HNH))(F)
 N(NH(HN(HEX&n-acetyl&sulfate))(HNHS))(F)
 N(NH(HN(HEX&sulfate))(HNHS))(F)
 N(NH(HNH)((HEX&phosphate)))(F)

## Slide 2
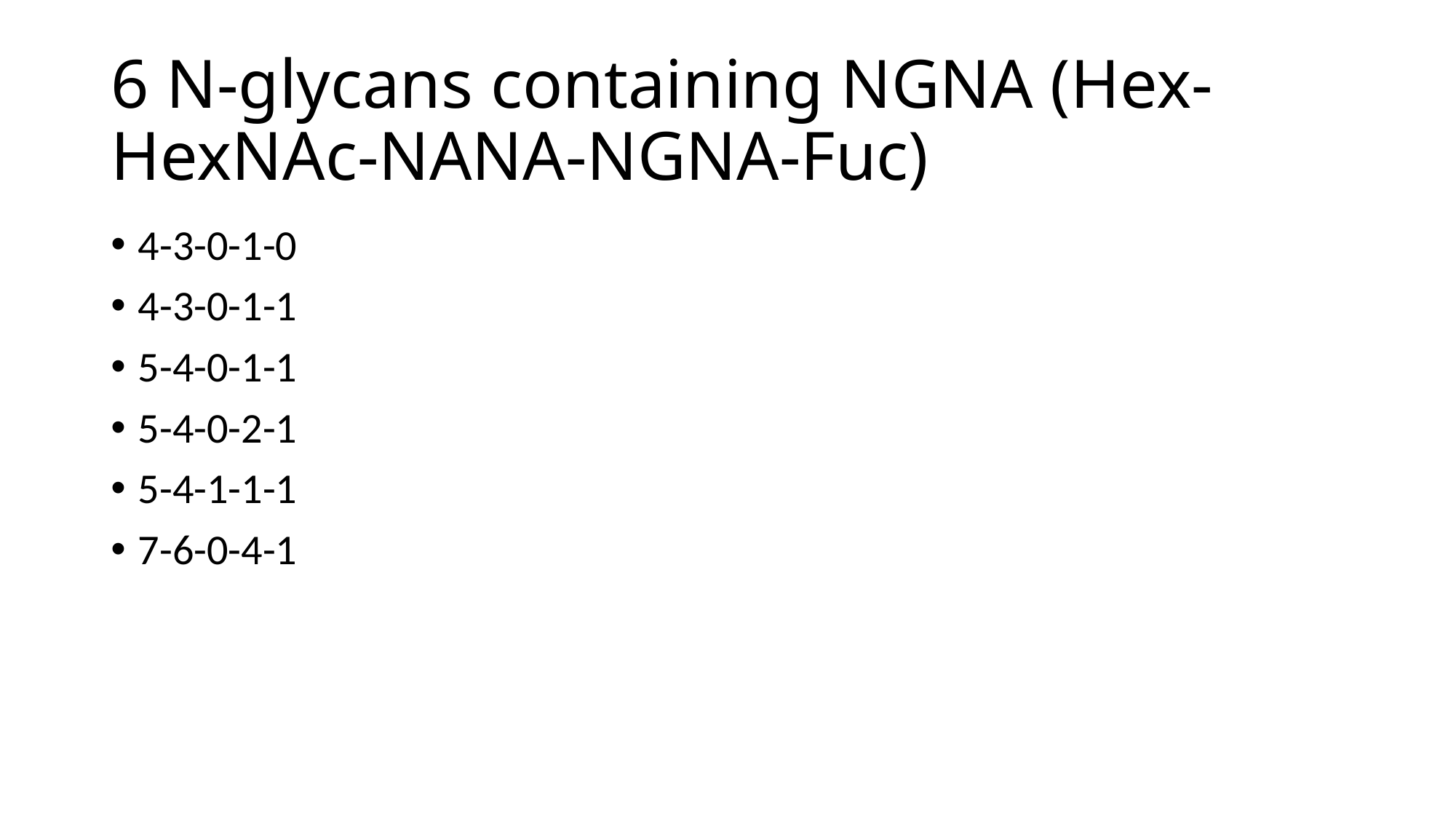

# 6 N-glycans containing NGNA (Hex-HexNAc-NANA-NGNA-Fuc)
4-3-0-1-0
4-3-0-1-1
5-4-0-1-1
5-4-0-2-1
5-4-1-1-1
7-6-0-4-1

## Slide 3
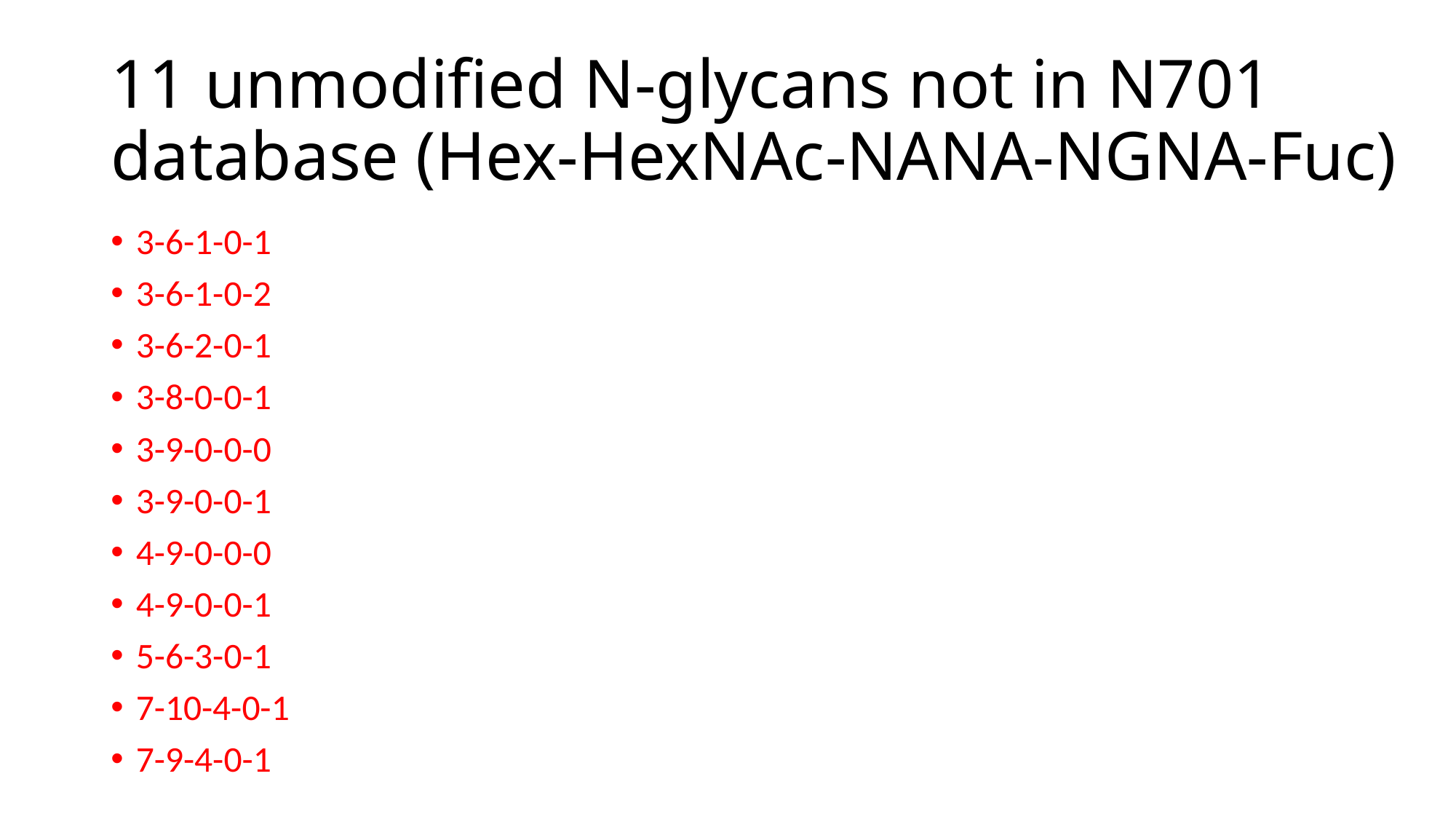

# 11 unmodified N-glycans not in N701 database (Hex-HexNAc-NANA-NGNA-Fuc)
3-6-1-0-1
3-6-1-0-2
3-6-2-0-1
3-8-0-0-1
3-9-0-0-0
3-9-0-0-1
4-9-0-0-0
4-9-0-0-1
5-6-3-0-1
7-10-4-0-1
7-9-4-0-1

## Slide 4
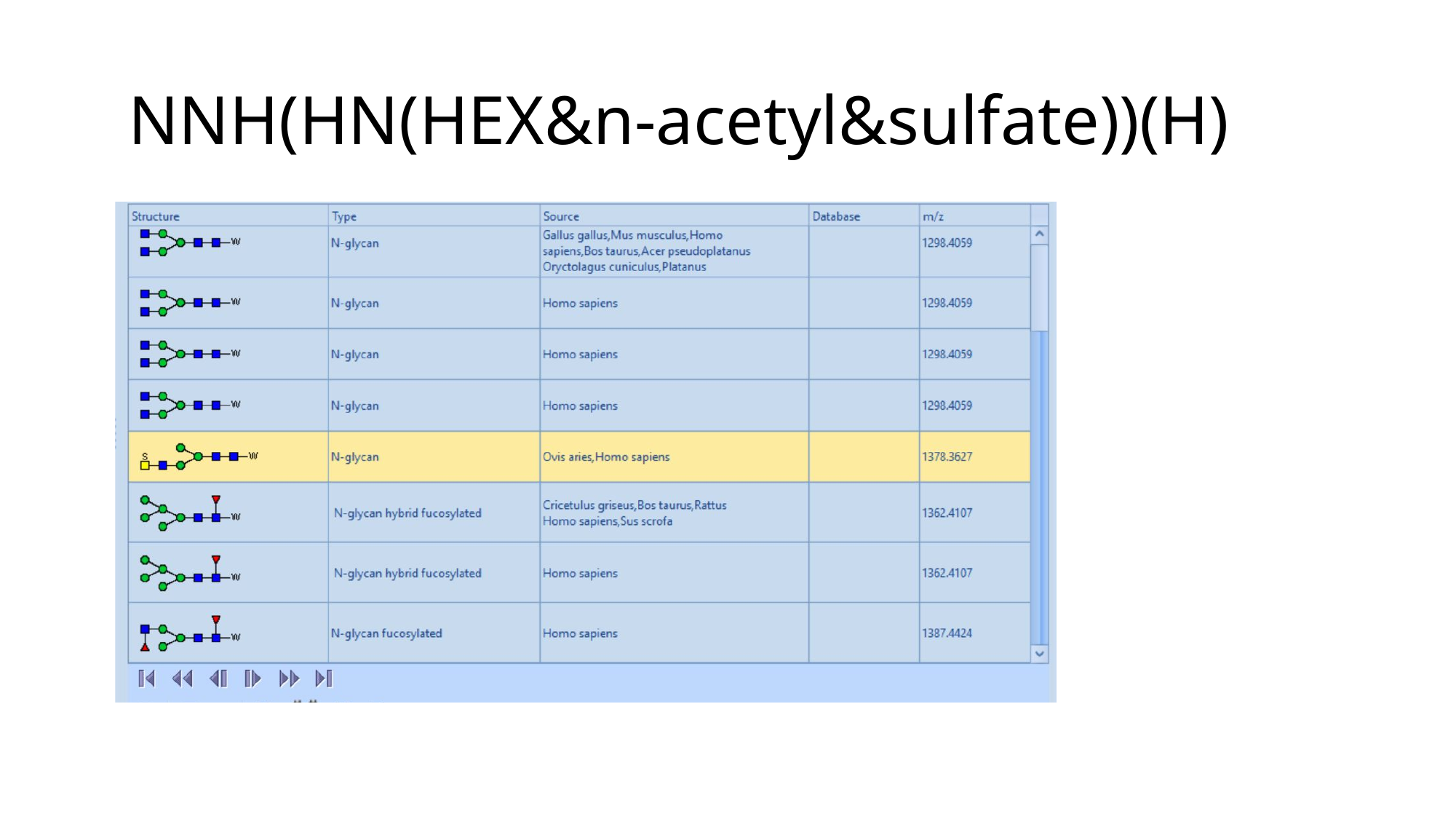

# NNH(HN(HEX&n-acetyl&sulfate))(H)

## Slide 5
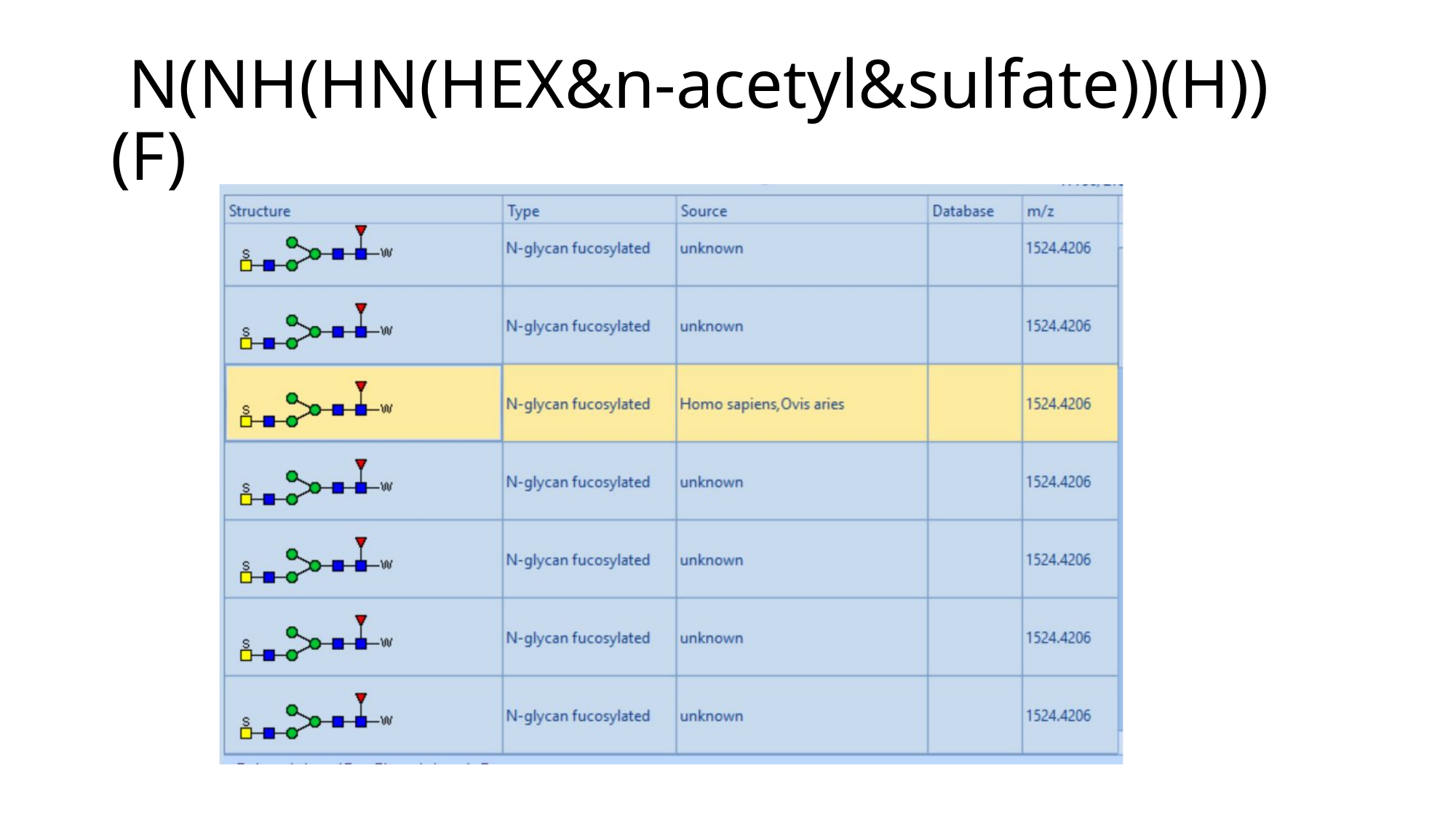

# N(NH(HN(HEX&n-acetyl&sulfate))(H))(F)

## Slide 6
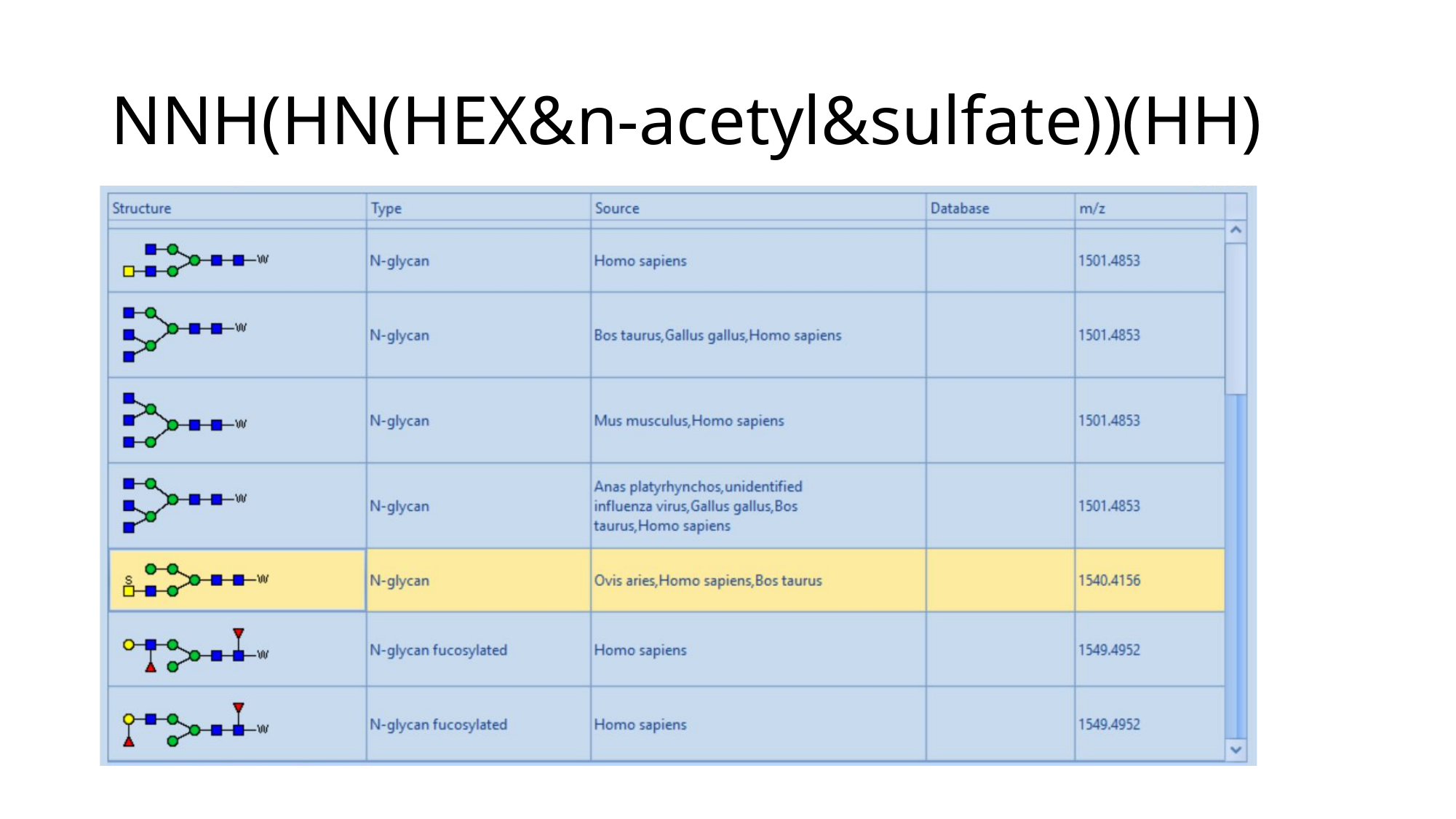

# NNH(HN(HEX&n-acetyl&sulfate))(HH)

## Slide 7
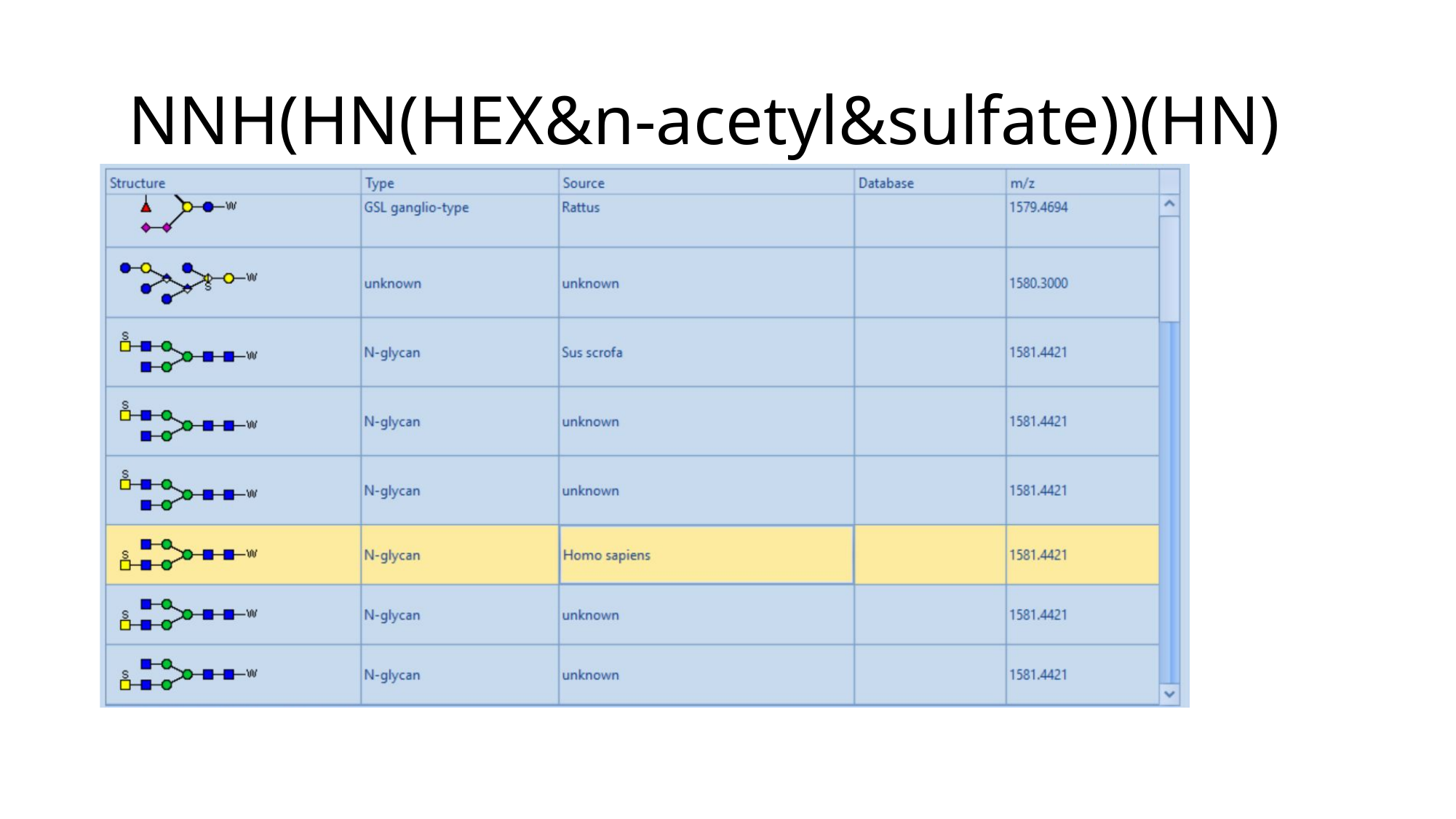

# NNH(HN(HEX&n-acetyl&sulfate))(HN)

## Slide 8
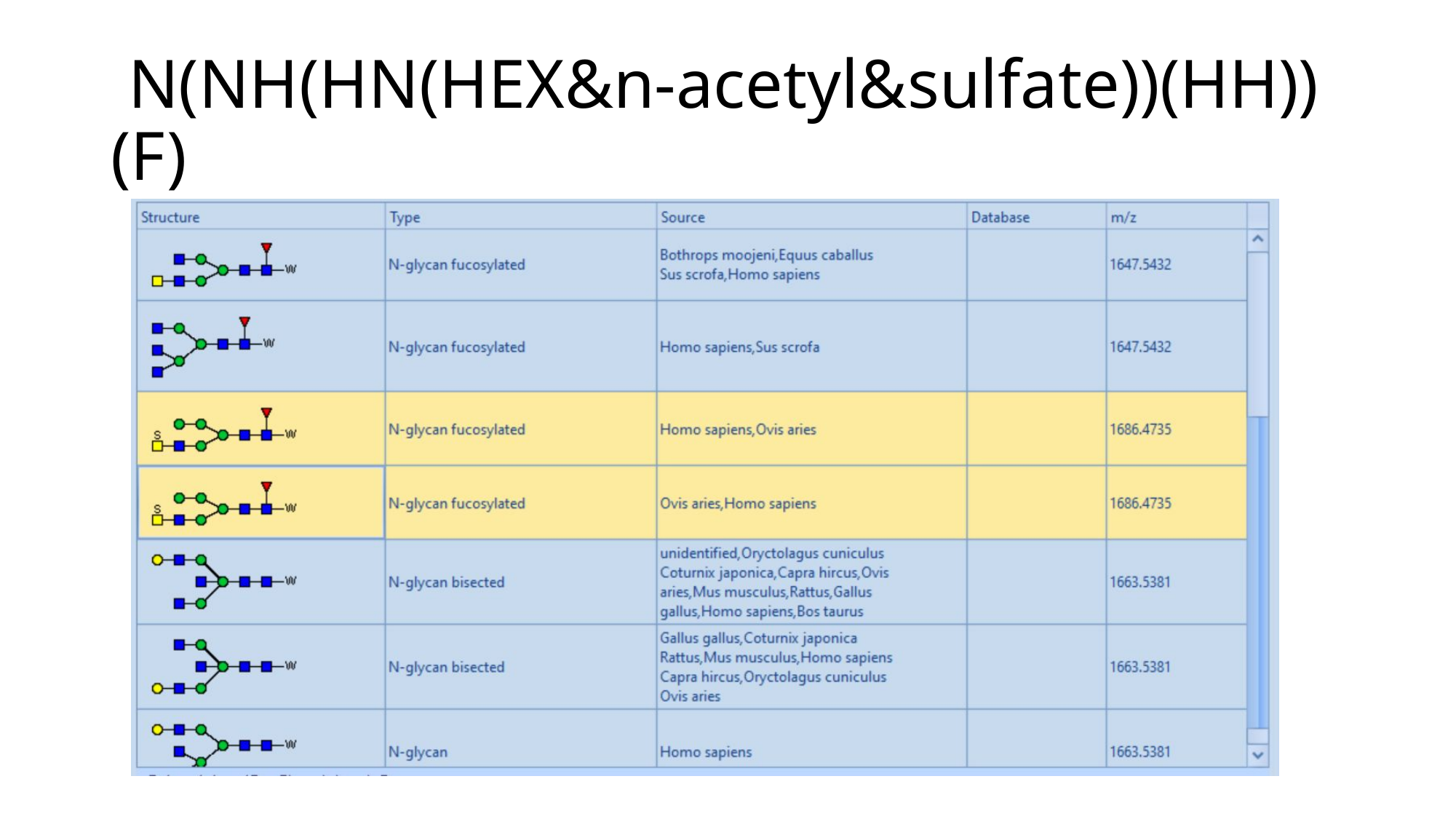

# N(NH(HN(HEX&n-acetyl&sulfate))(HH))(F)

## Slide 9
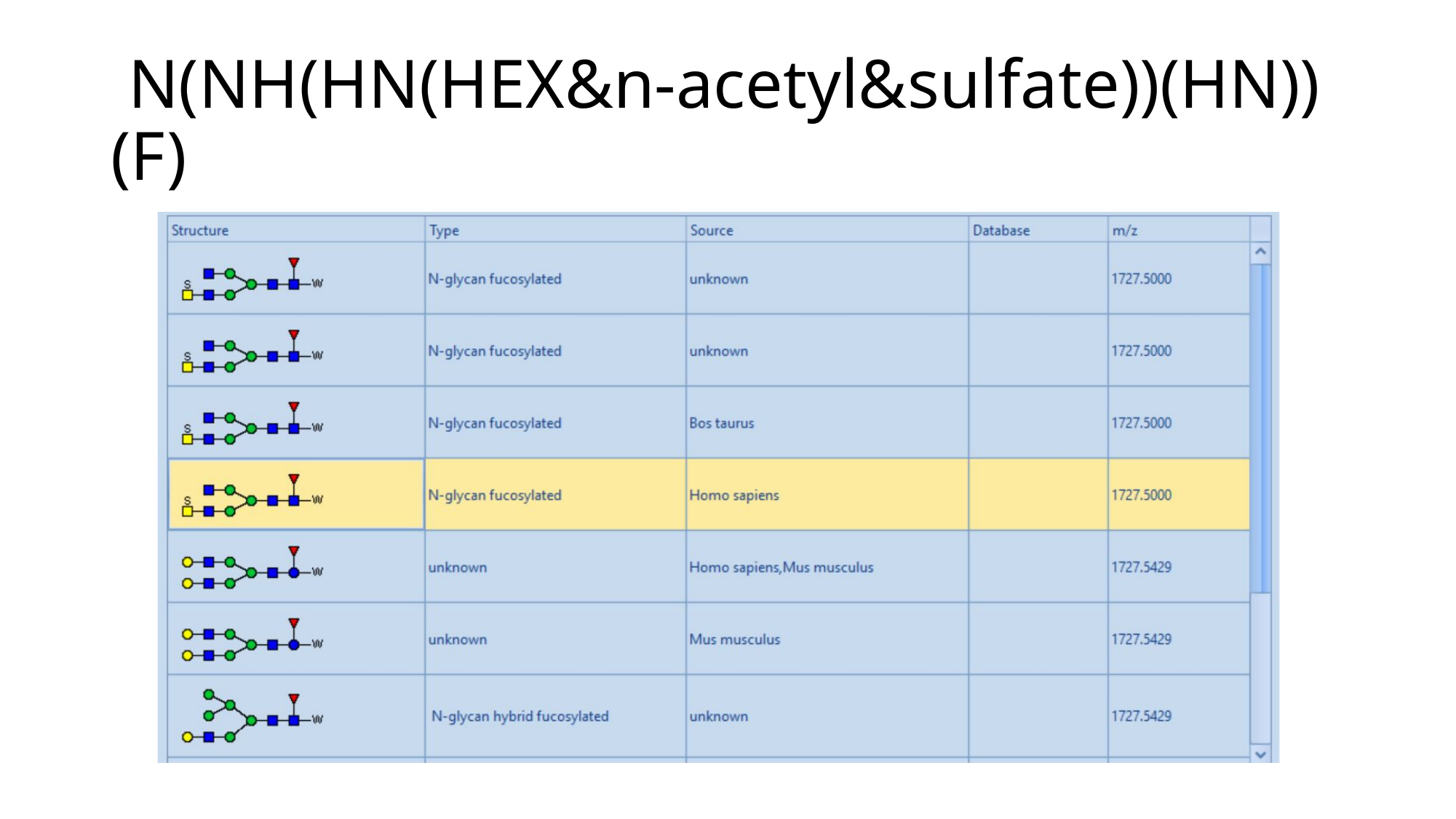

# N(NH(HN(HEX&n-acetyl&sulfate))(HN))(F)

## Slide 10
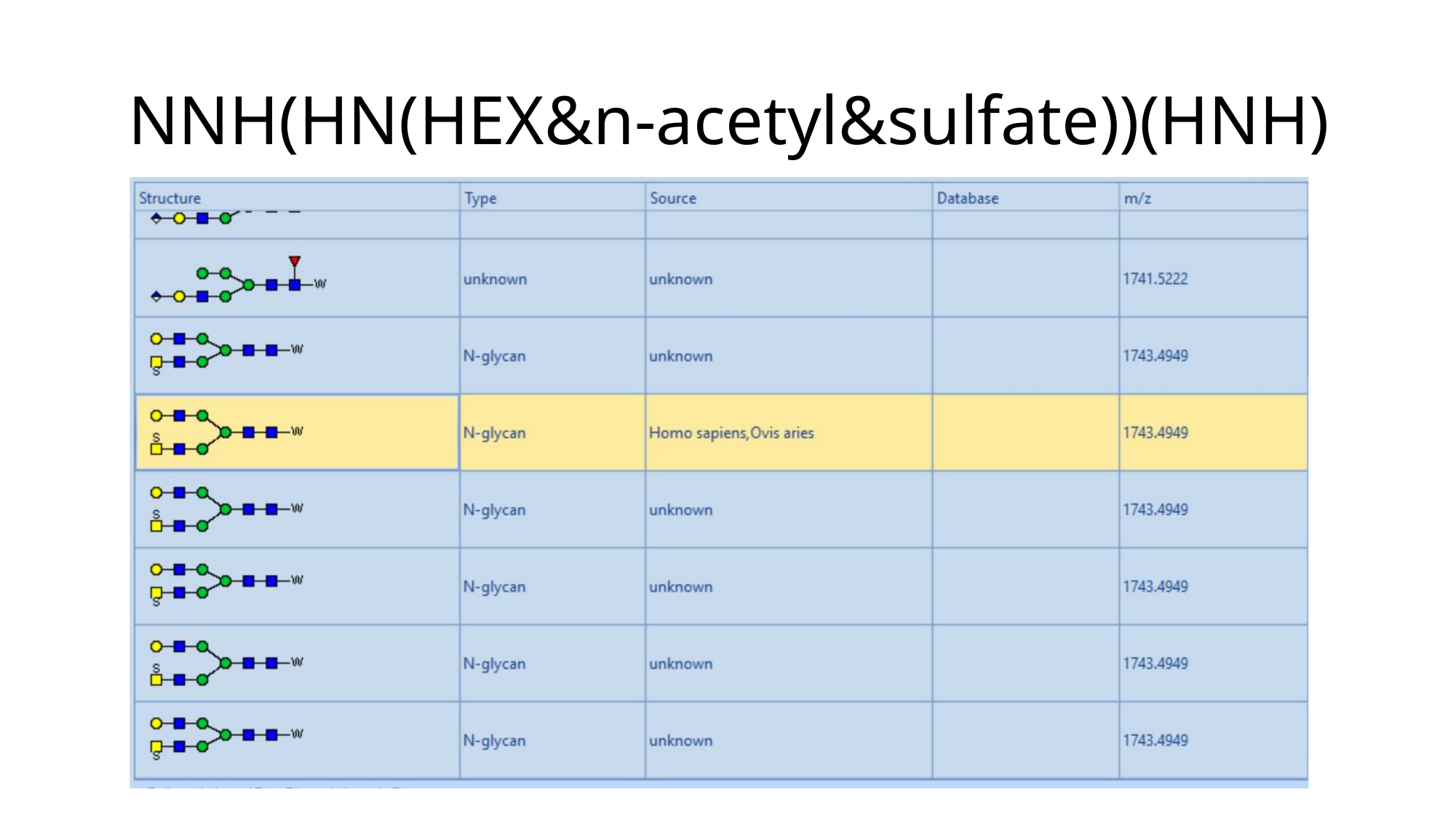

# NNH(HN(HEX&n-acetyl&sulfate))(HNH)

## Slide 11
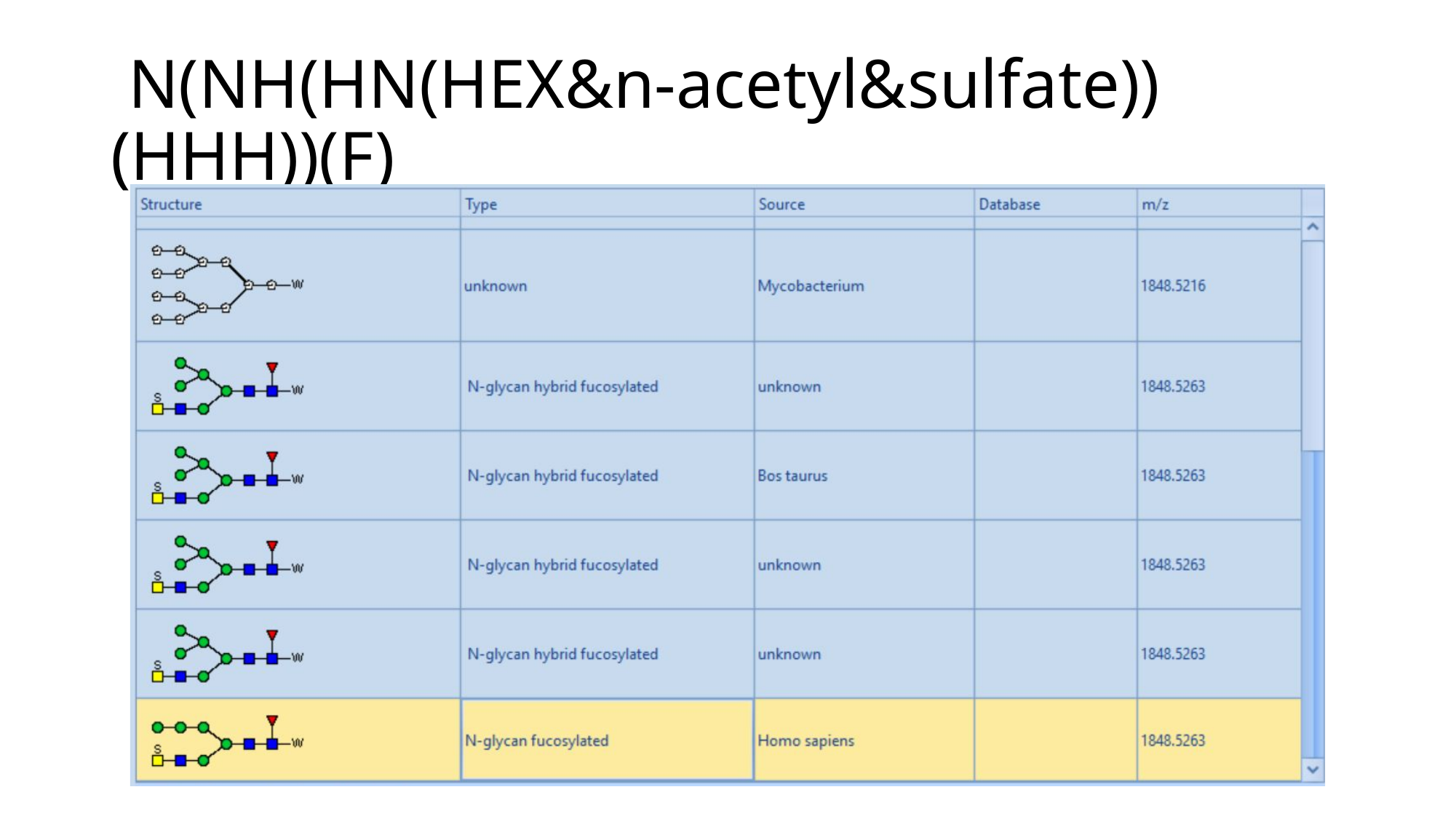

# N(NH(HN(HEX&n-acetyl&sulfate))(HHH))(F)

## Slide 12
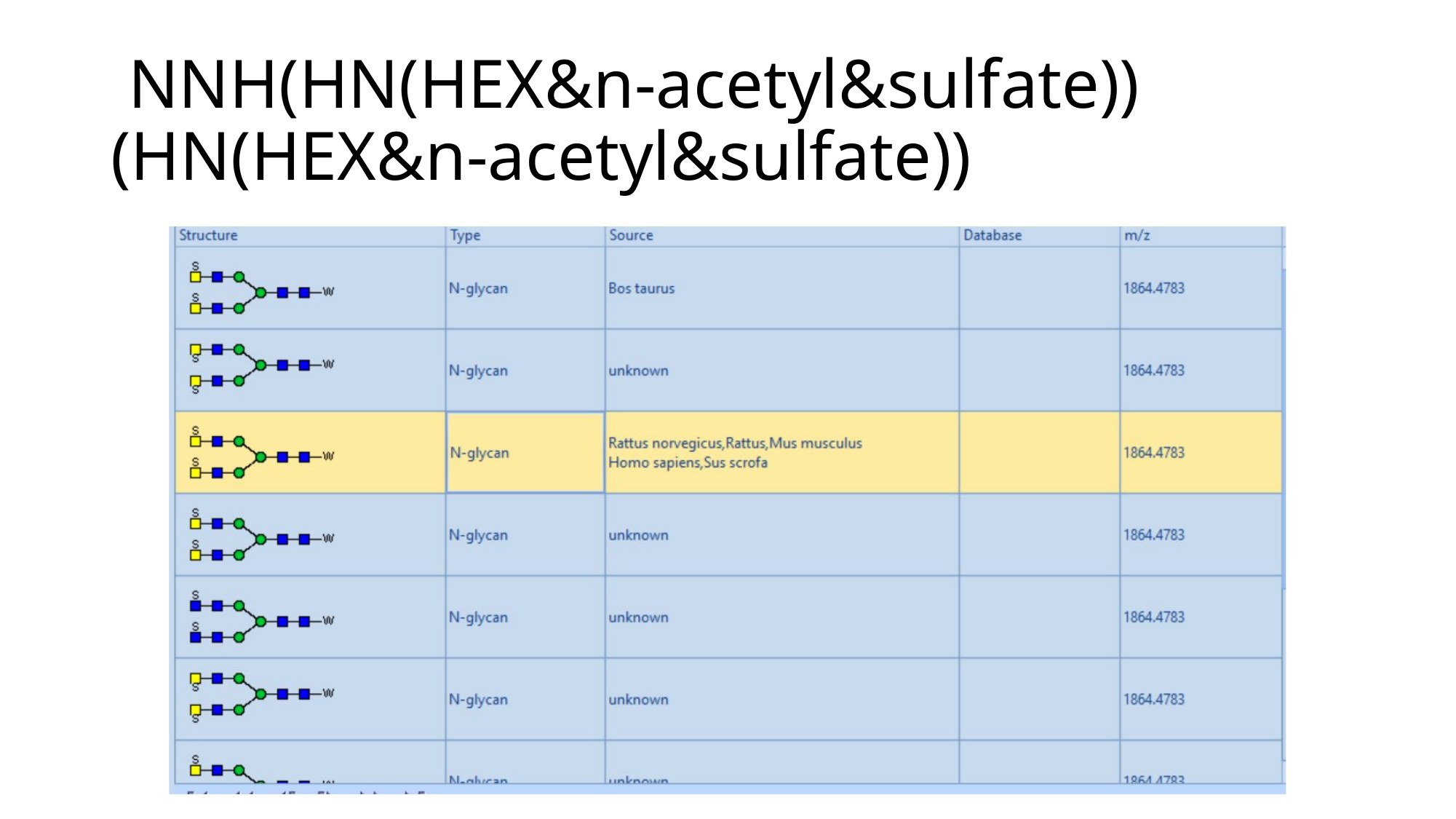

# NNH(HN(HEX&n-acetyl&sulfate))(HN(HEX&n-acetyl&sulfate))

## Slide 13
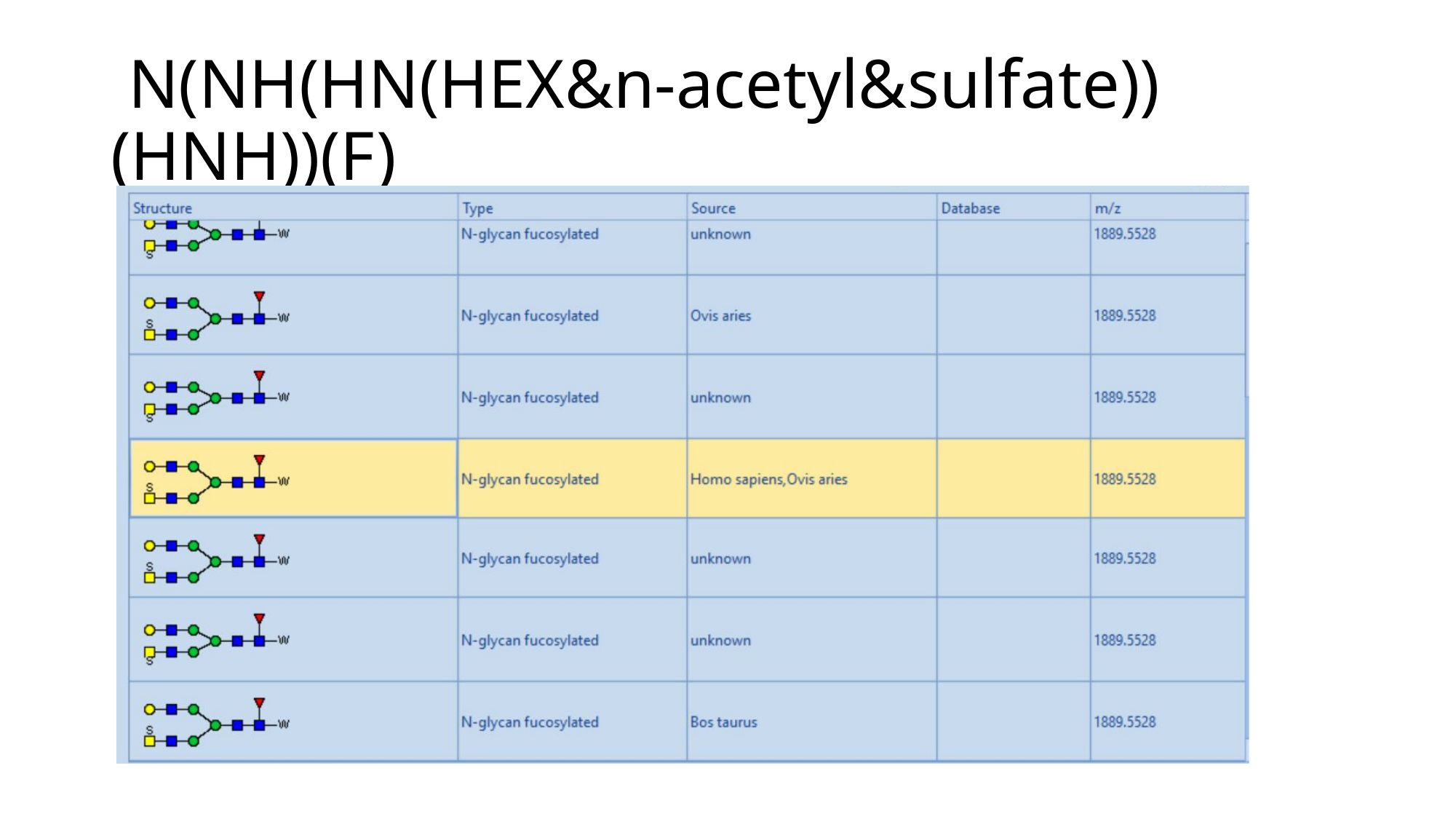

# N(NH(HN(HEX&n-acetyl&sulfate))(HNH))(F)

## Slide 14
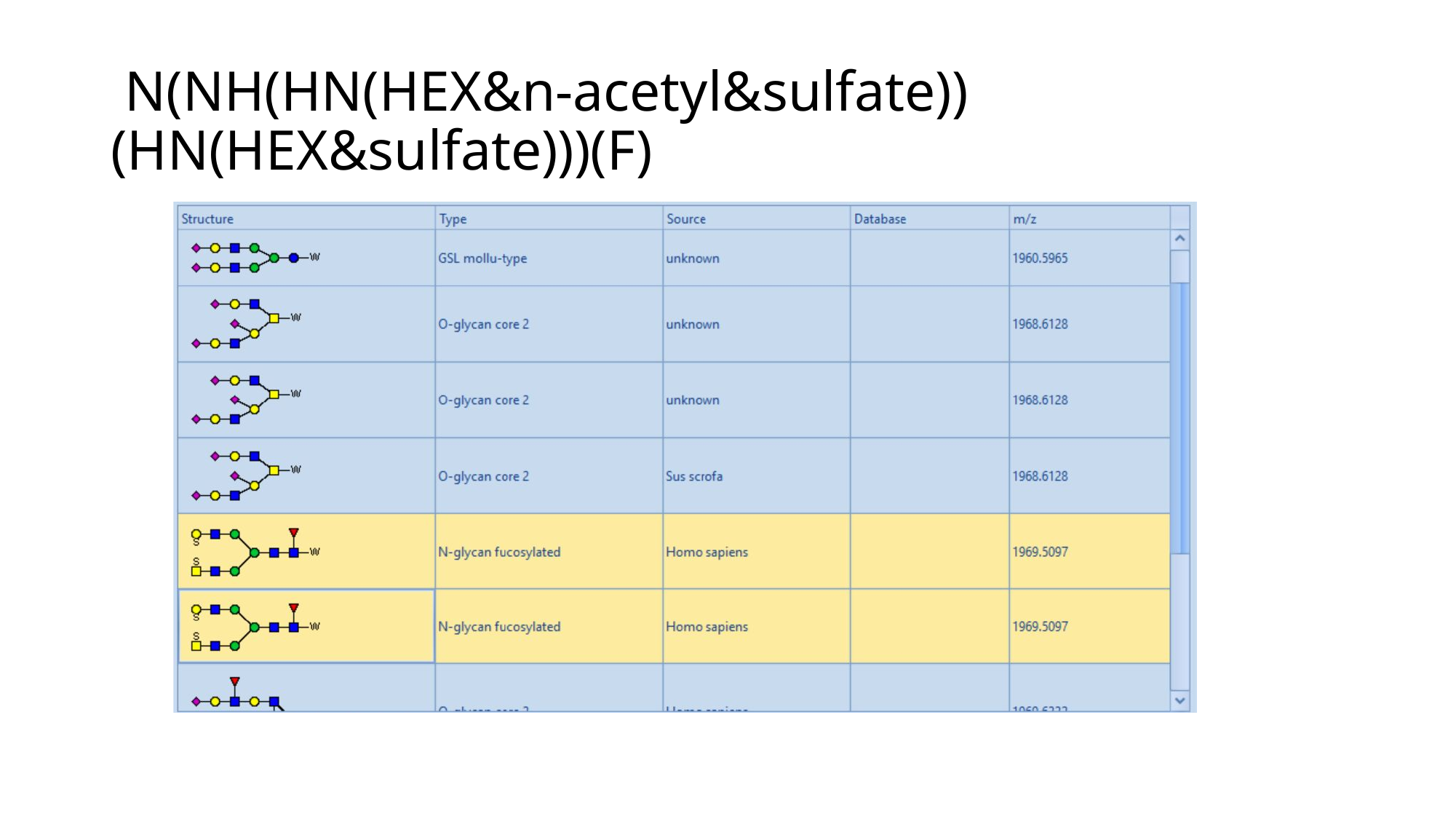

# N(NH(HN(HEX&n-acetyl&sulfate))(HN(HEX&sulfate)))(F)

## Slide 15
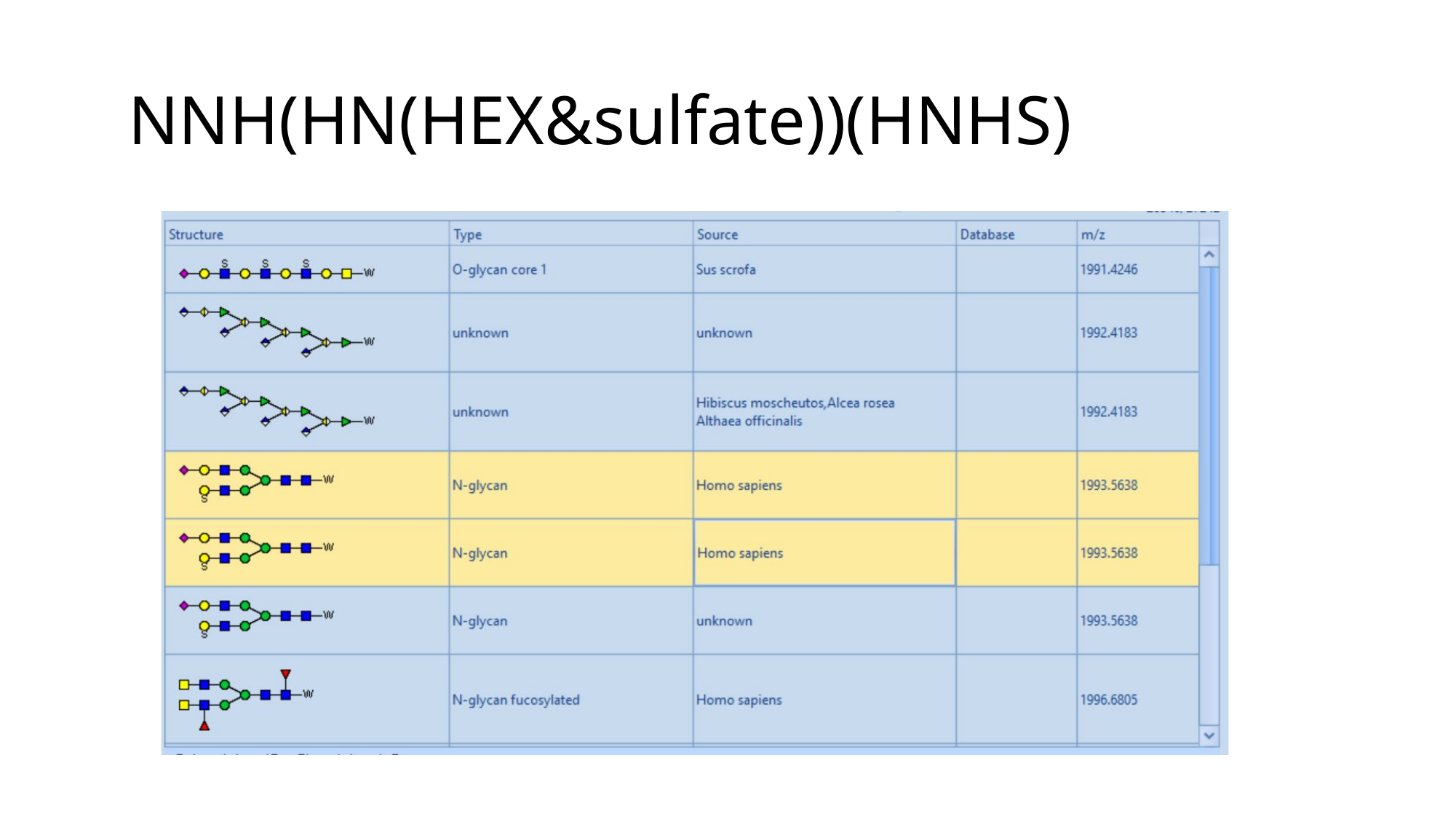

# NNH(HN(HEX&sulfate))(HNHS)

## Slide 16
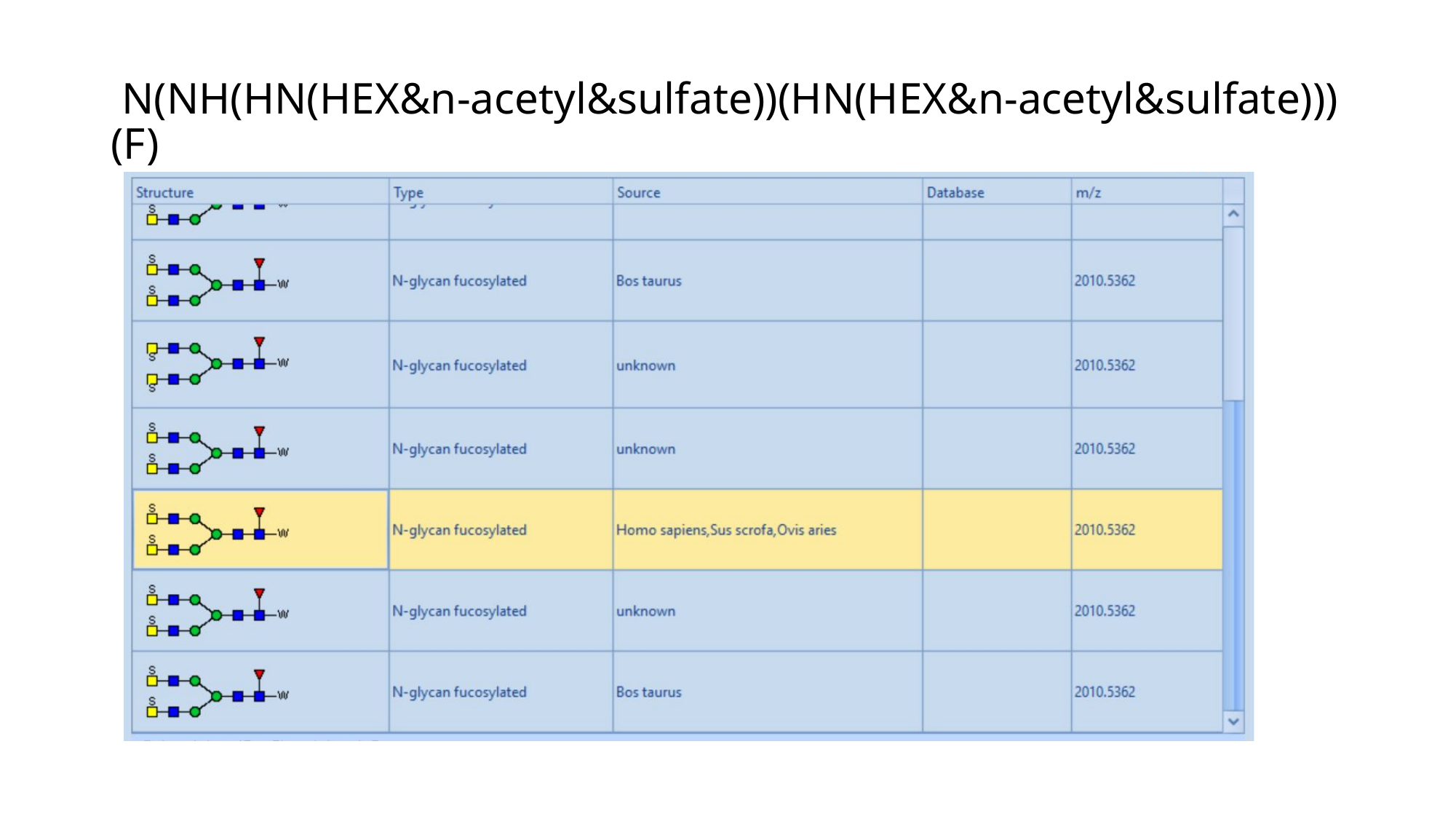

# N(NH(HN(HEX&n-acetyl&sulfate))(HN(HEX&n-acetyl&sulfate)))(F)

## Slide 17
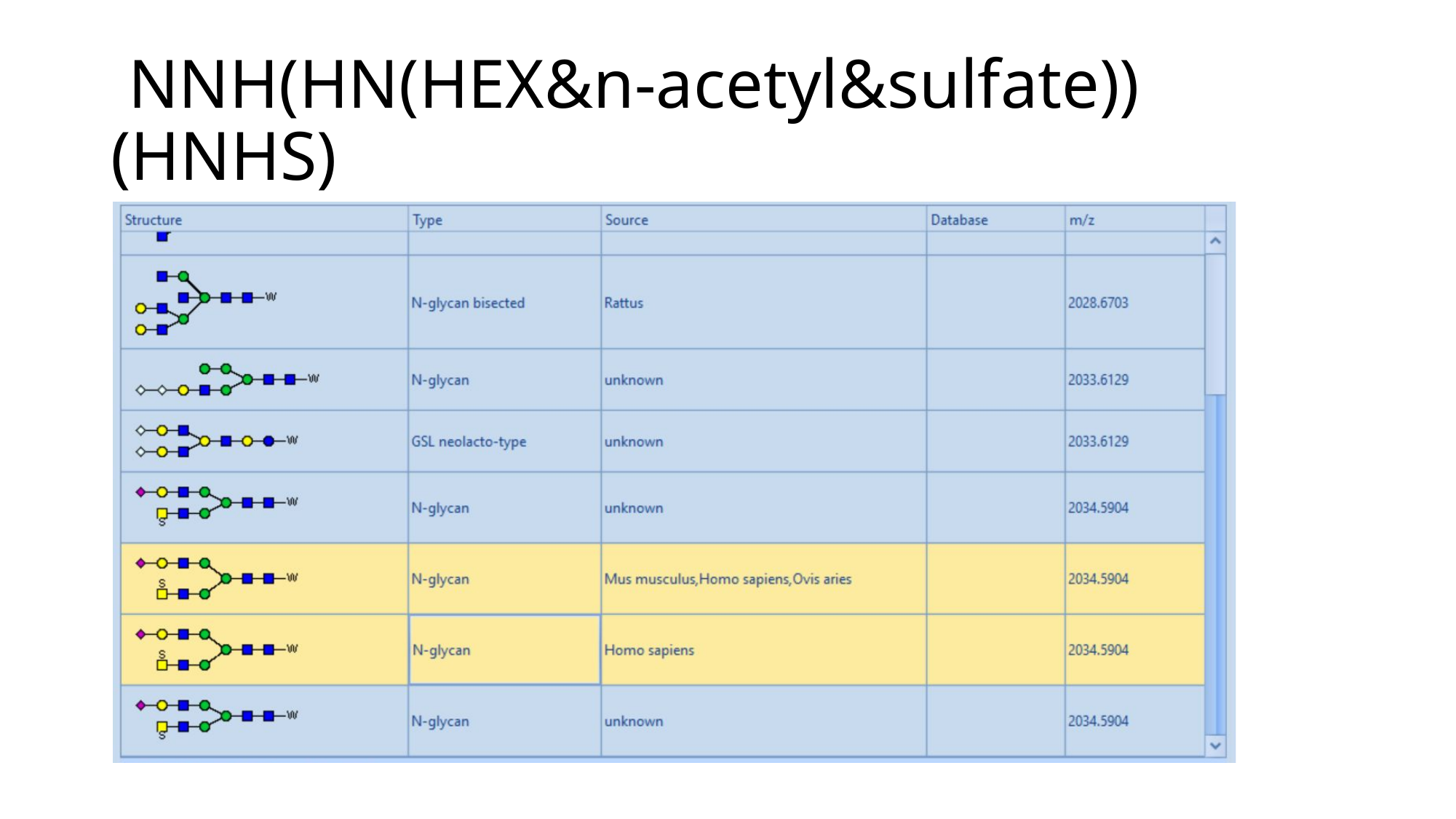

# NNH(HN(HEX&n-acetyl&sulfate))(HNHS)

## Slide 18
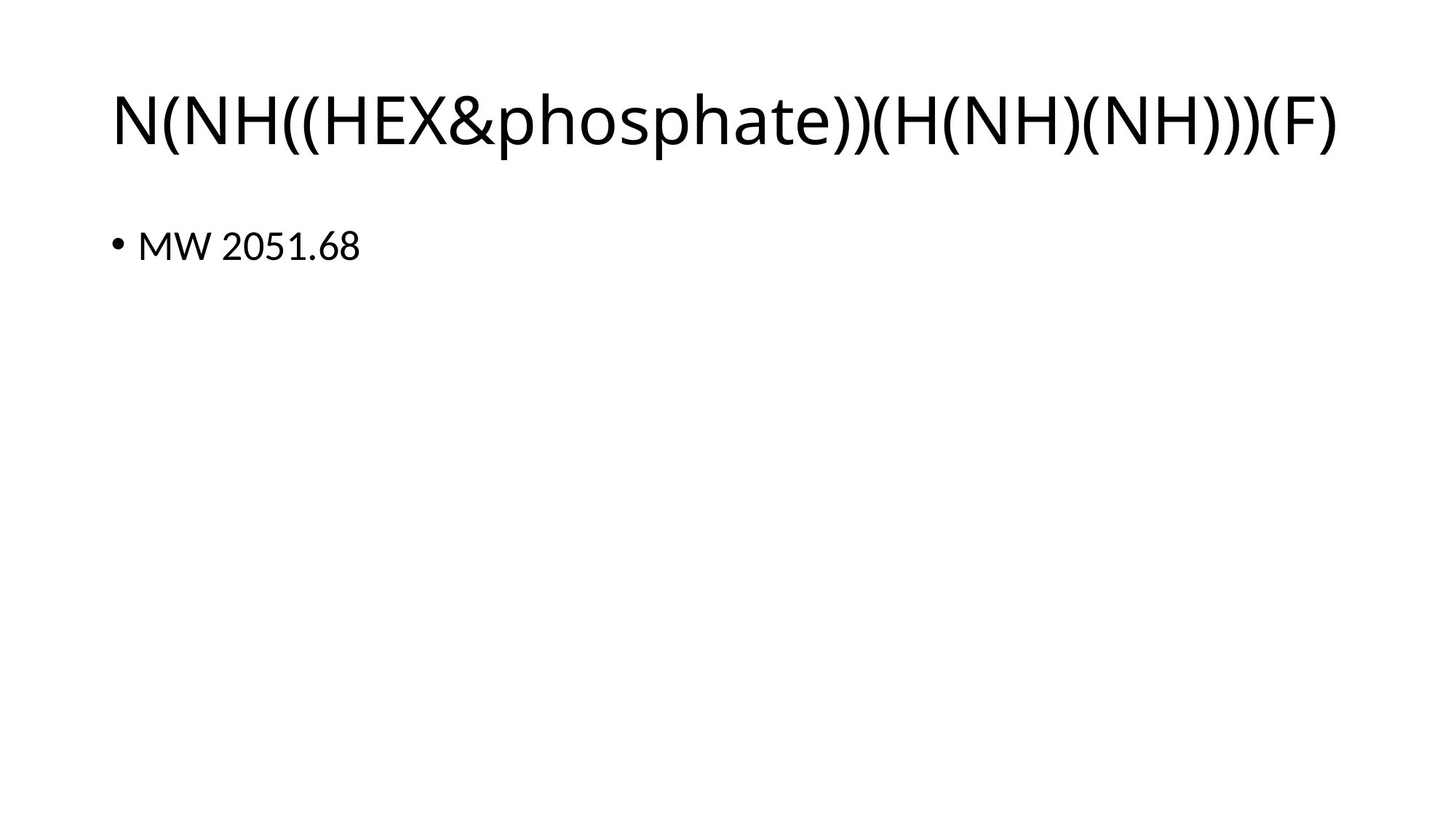

# N(NH((HEX&phosphate))(H(NH)(NH)))(F)
MW 2051.68

## Slide 19
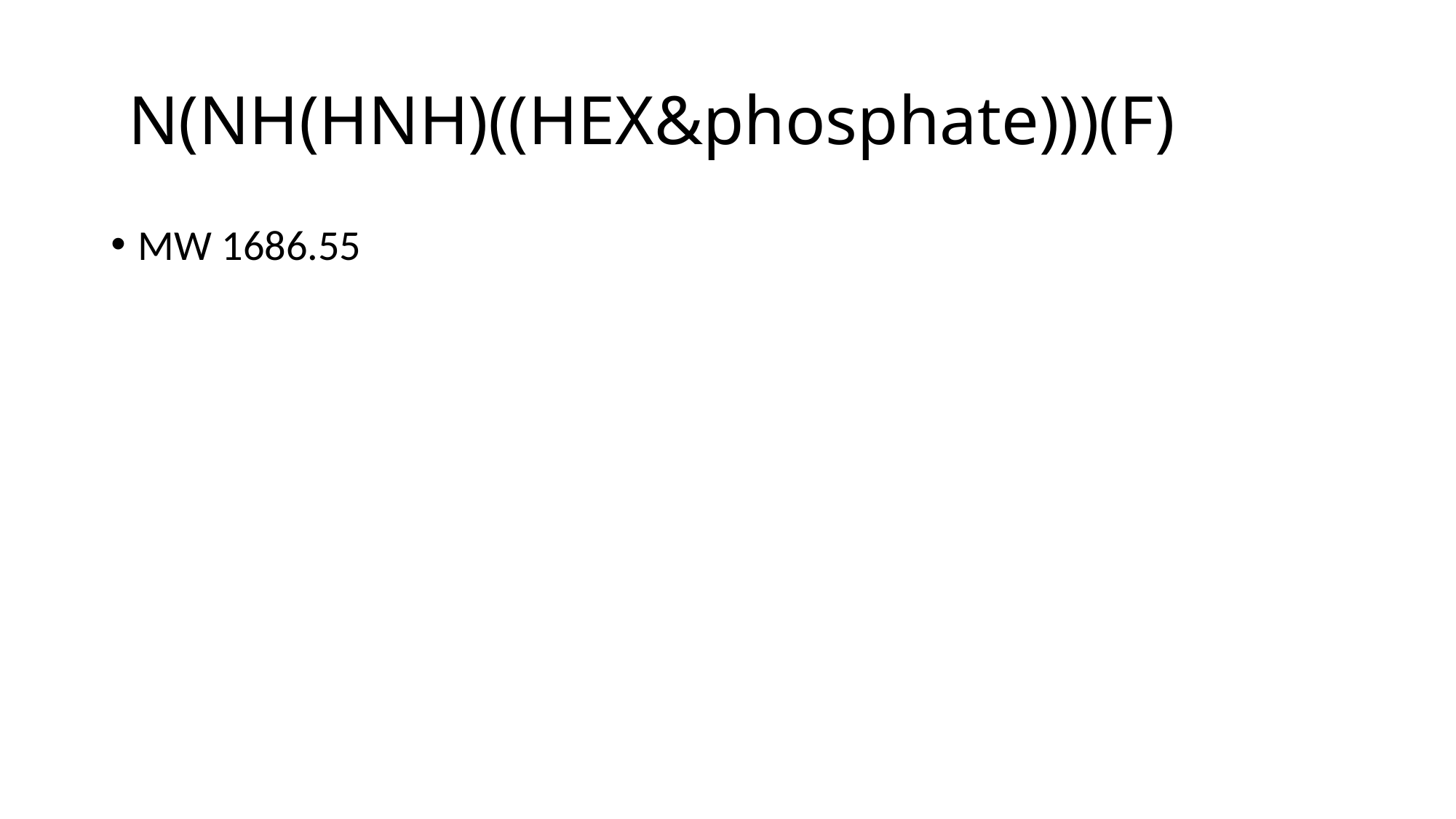

# N(NH(HNH)((HEX&phosphate)))(F)
MW 1686.55

## Slide 20
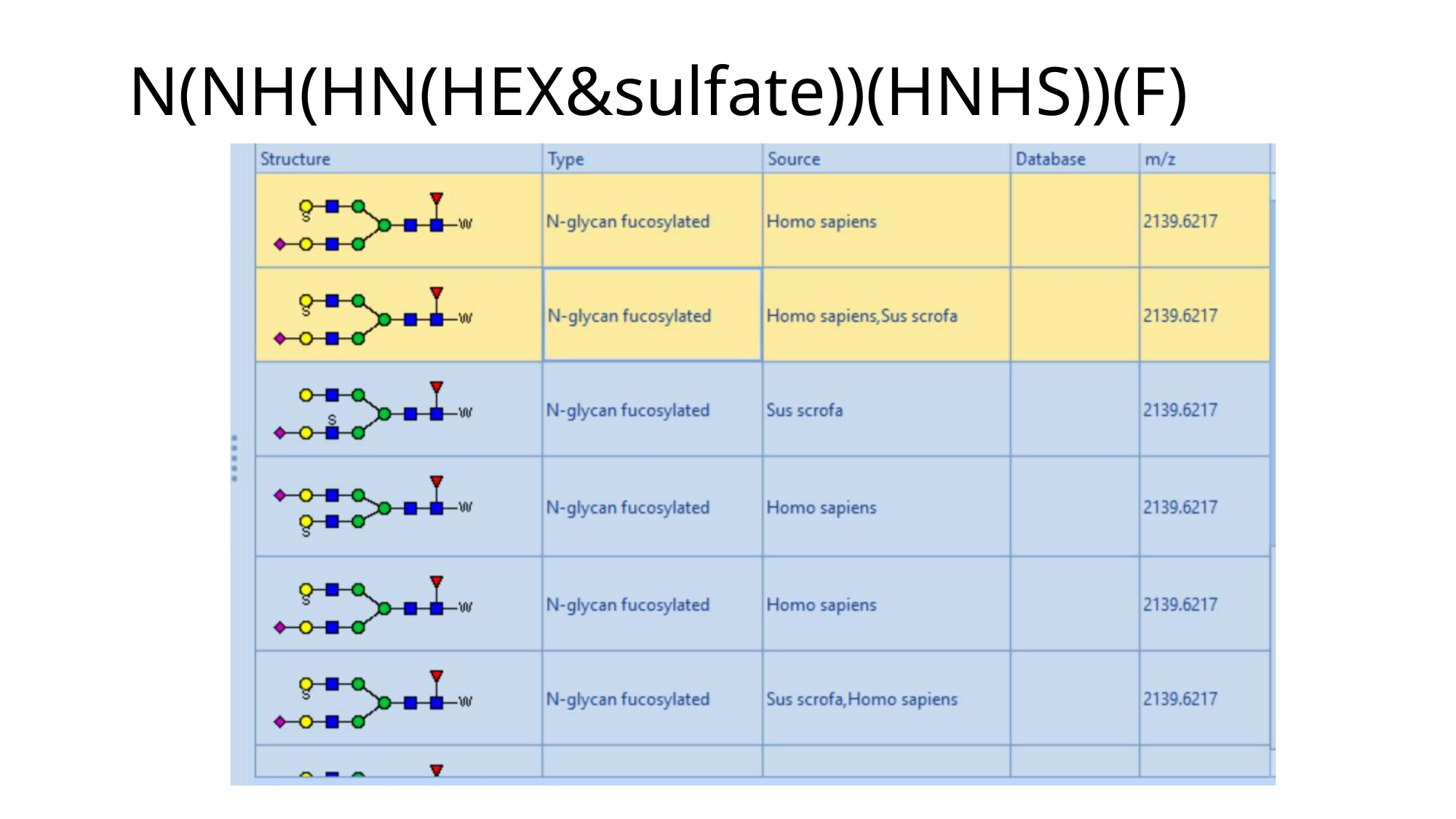

# N(NH(HN(HEX&sulfate))(HNHS))(F)

## Slide 21
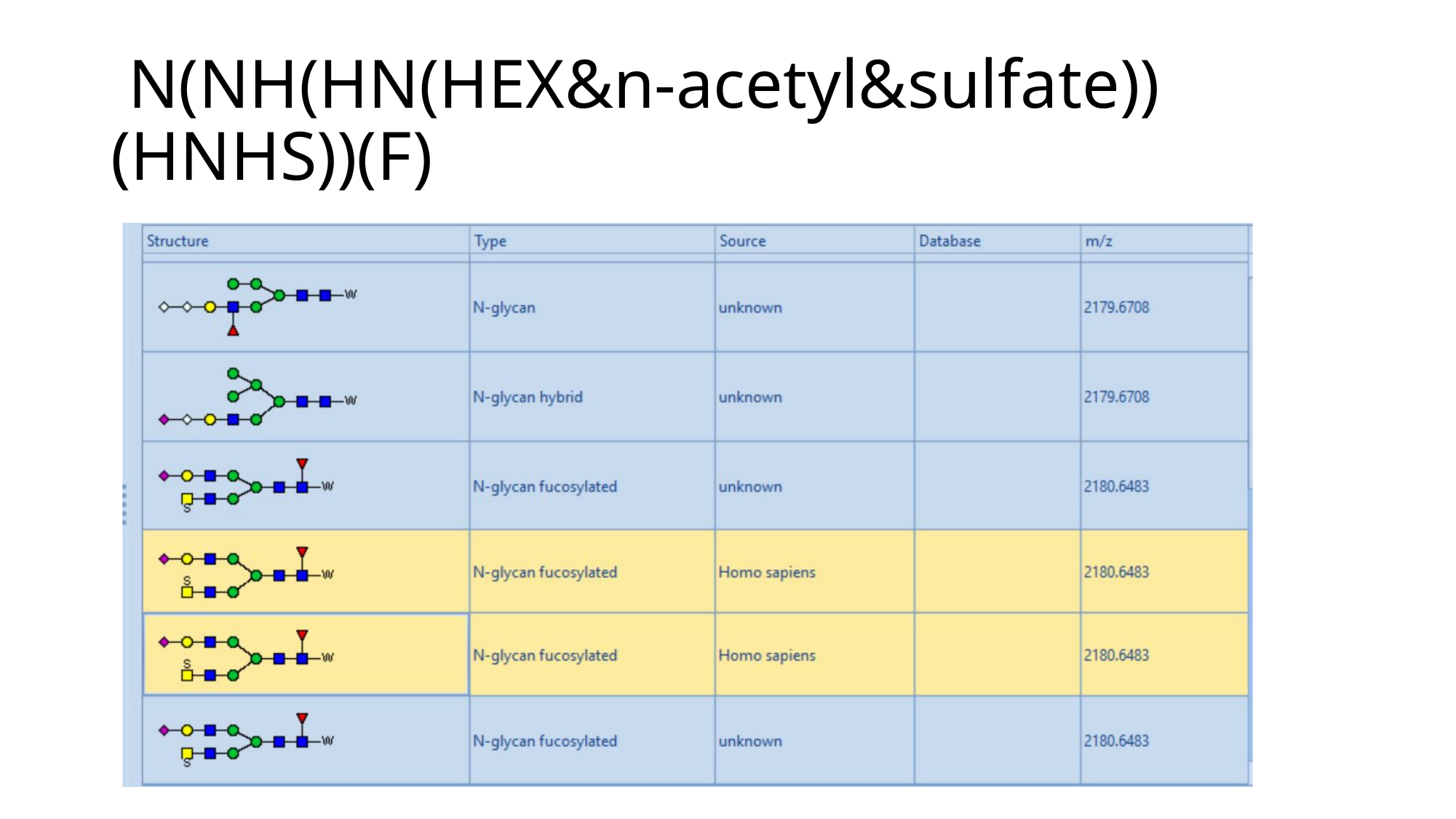

# N(NH(HN(HEX&n-acetyl&sulfate))(HNHS))(F)

## Slide 22
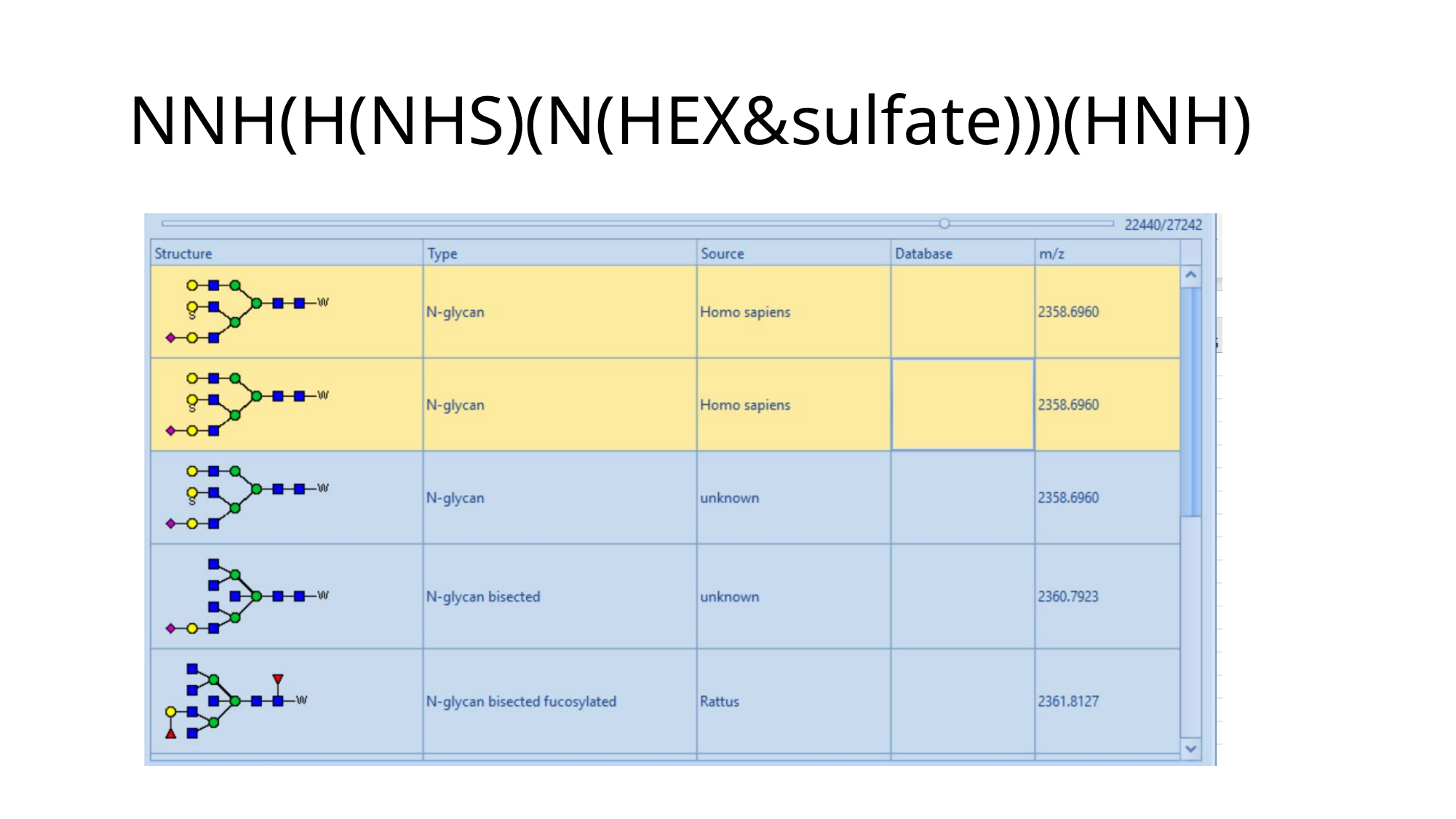

# NNH(H(NHS)(N(HEX&sulfate)))(HNH)

## Slide 23
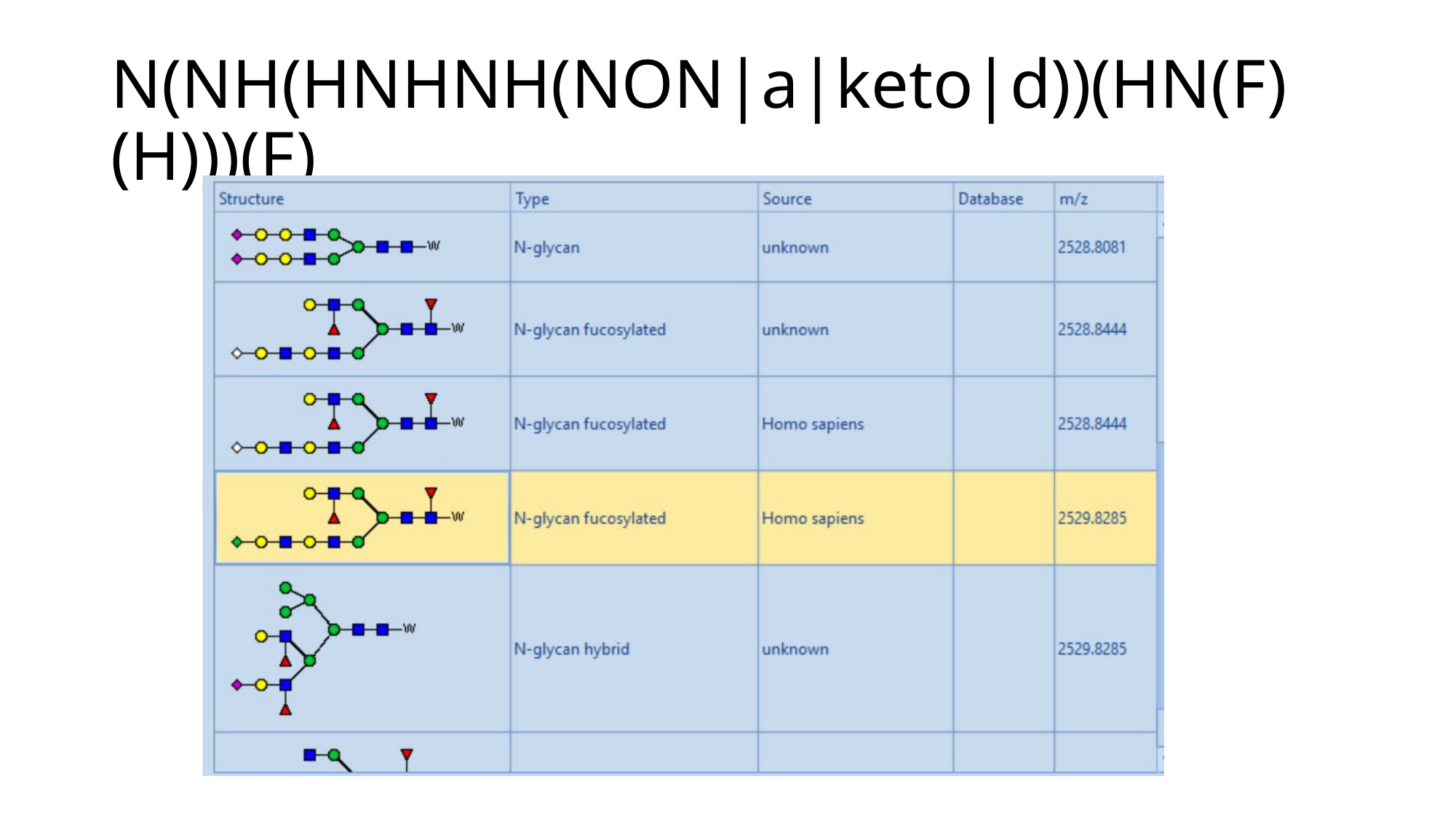

# N(NH(HNHNH(NON|a|keto|d))(HN(F)(H)))(F)

## Slide 24
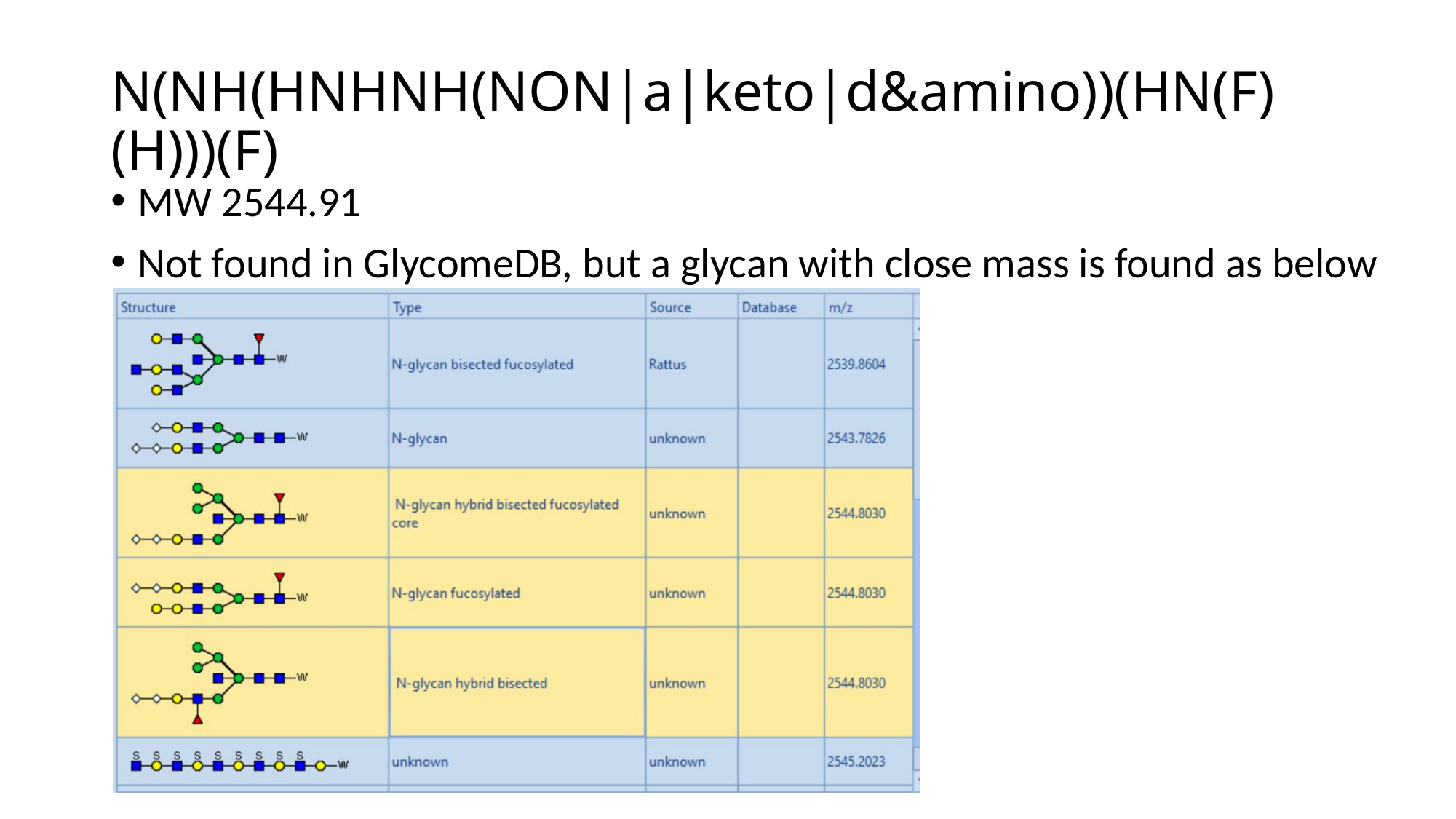

# N(NH(HNHNH(NON|a|keto|d&amino))(HN(F)(H)))(F)
MW 2544.91
Not found in GlycomeDB, but a glycan with close mass is found as below

## Slide 25
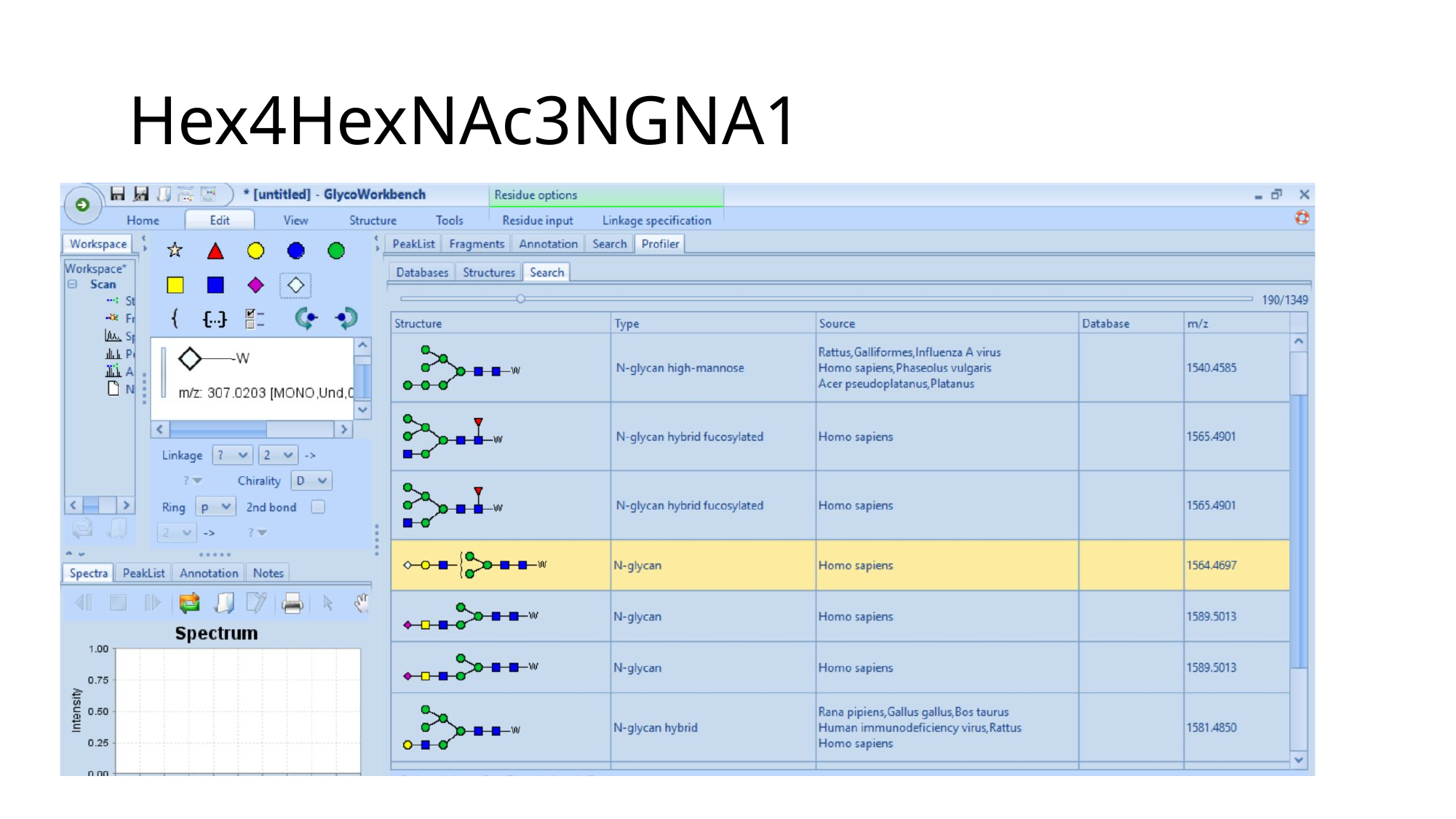

# Hex4HexNAc3NGNA1

## Slide 26
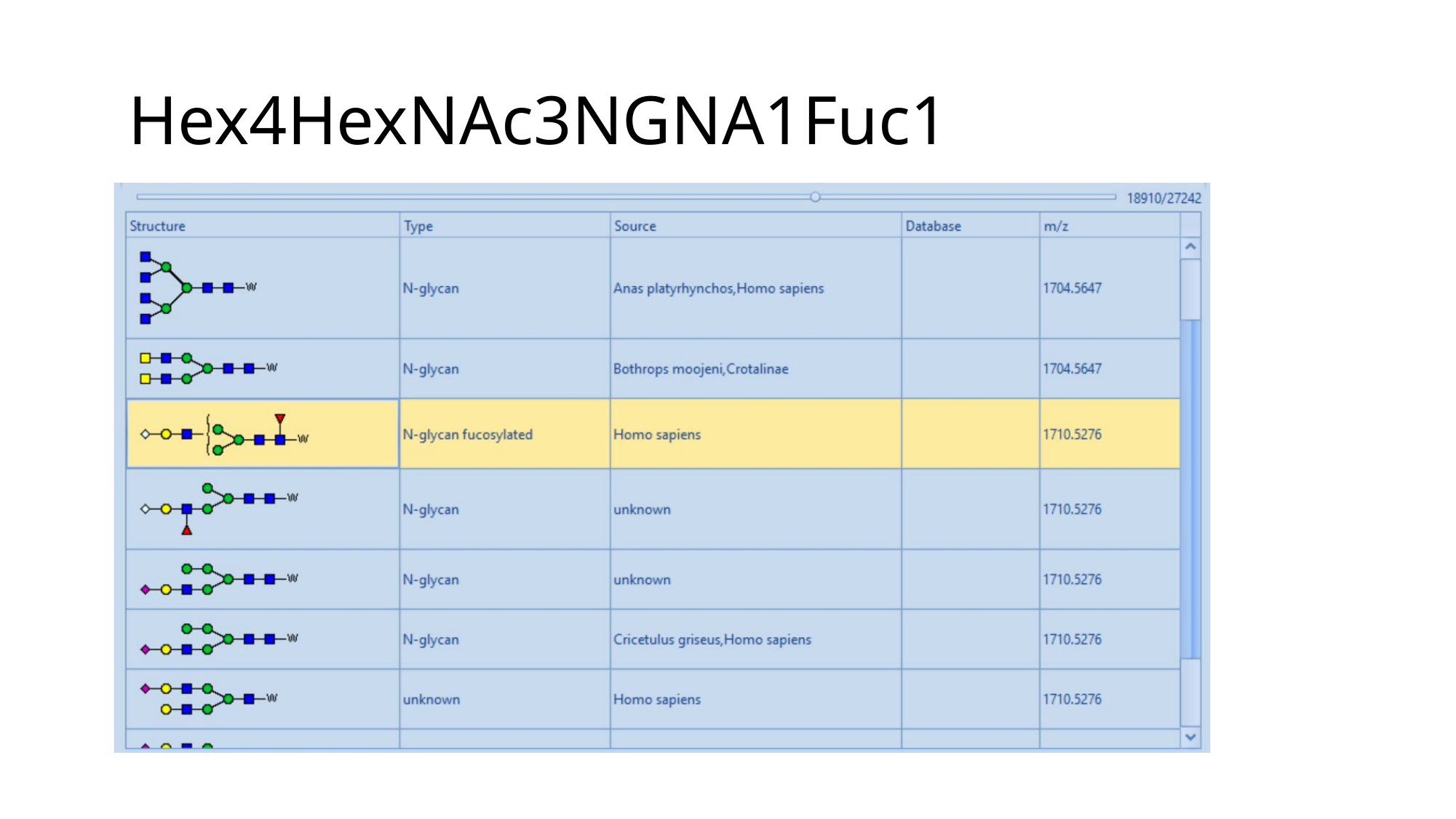

# Hex4HexNAc3NGNA1Fuc1

## Slide 27
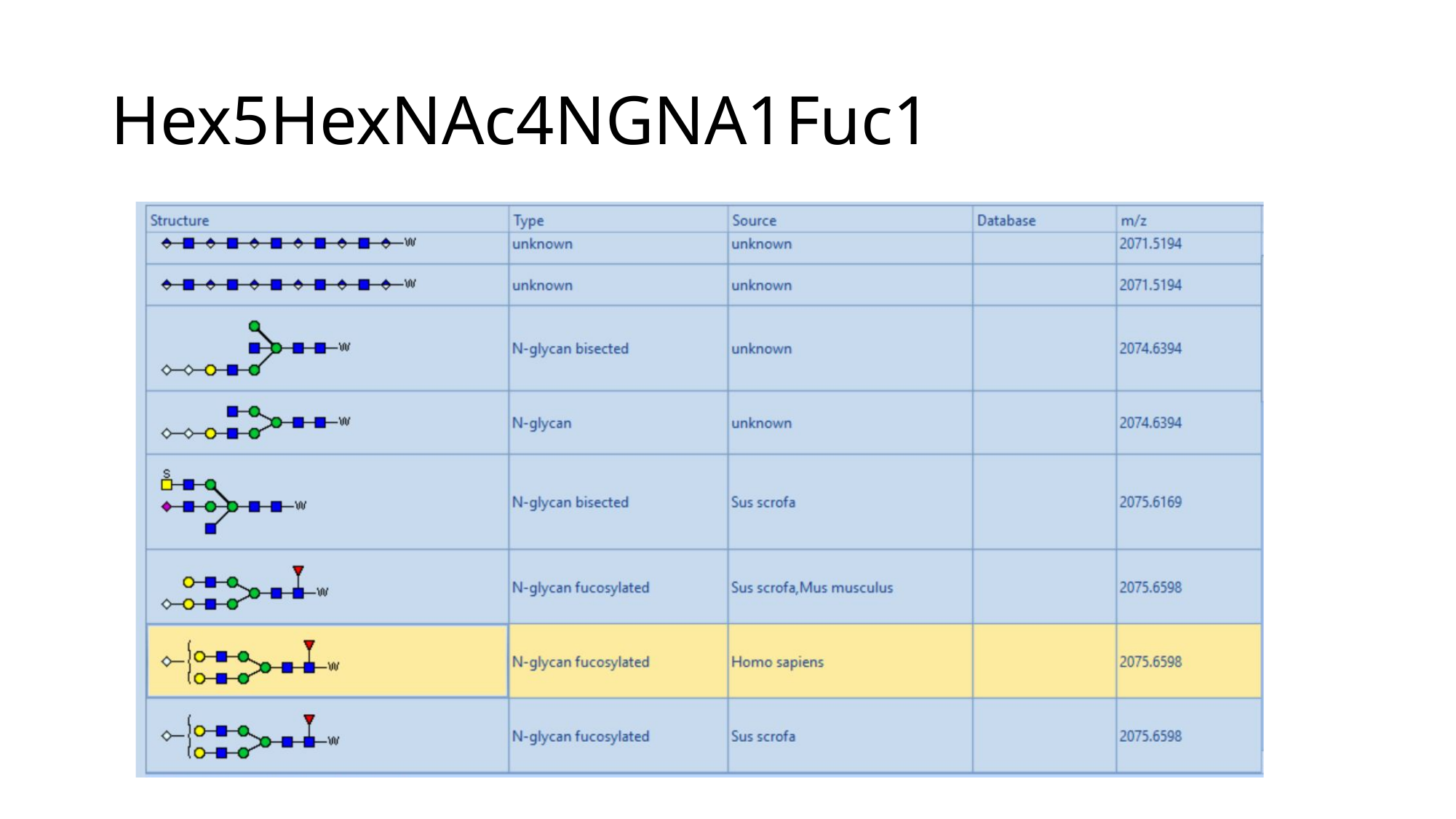

# Hex5HexNAc4NGNA1Fuc1

## Slide 28
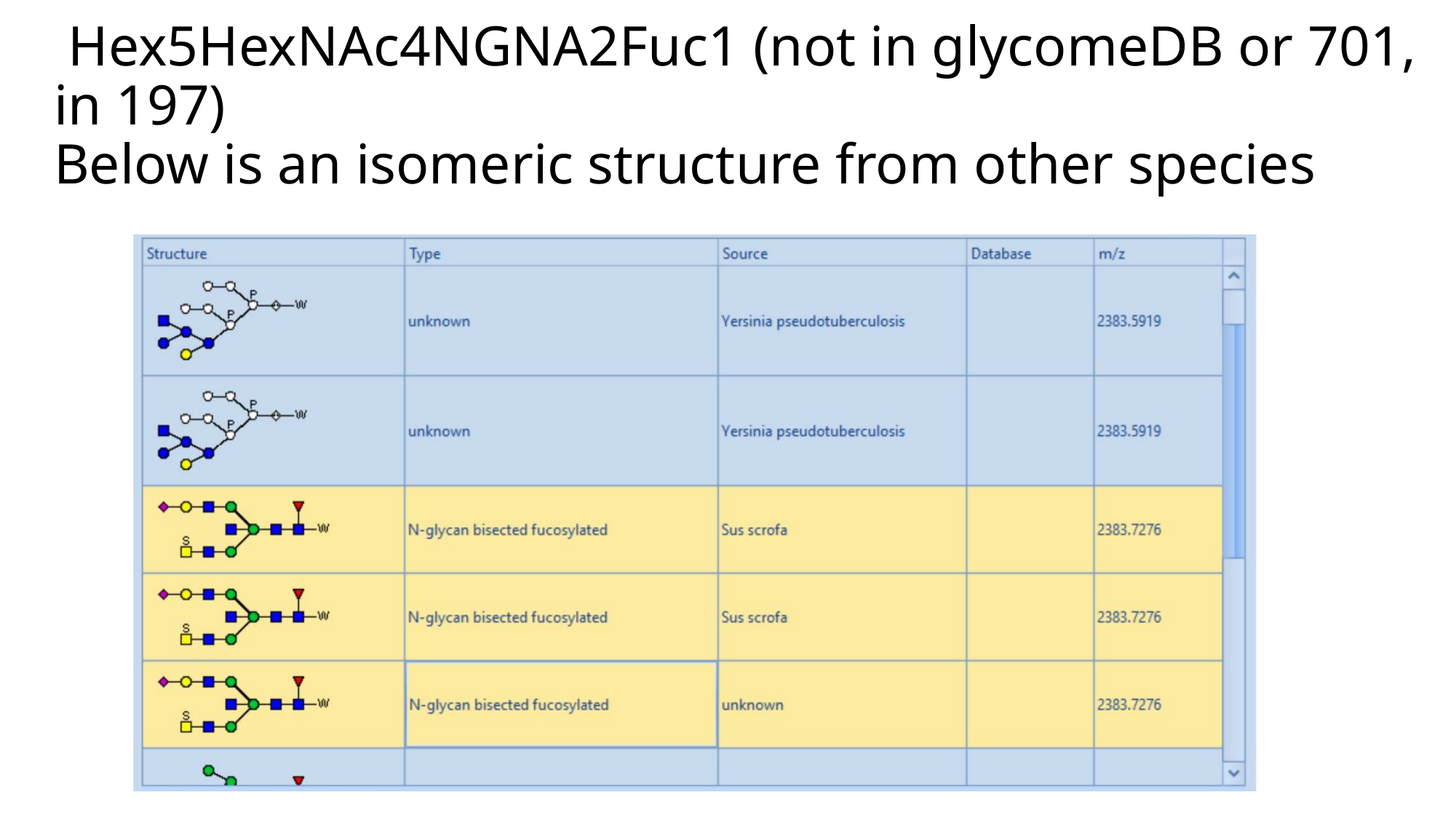

# Hex5HexNAc4NGNA2Fuc1 (not in glycomeDB or 701, in 197) Below is an isomeric structure from other species

## Slide 29
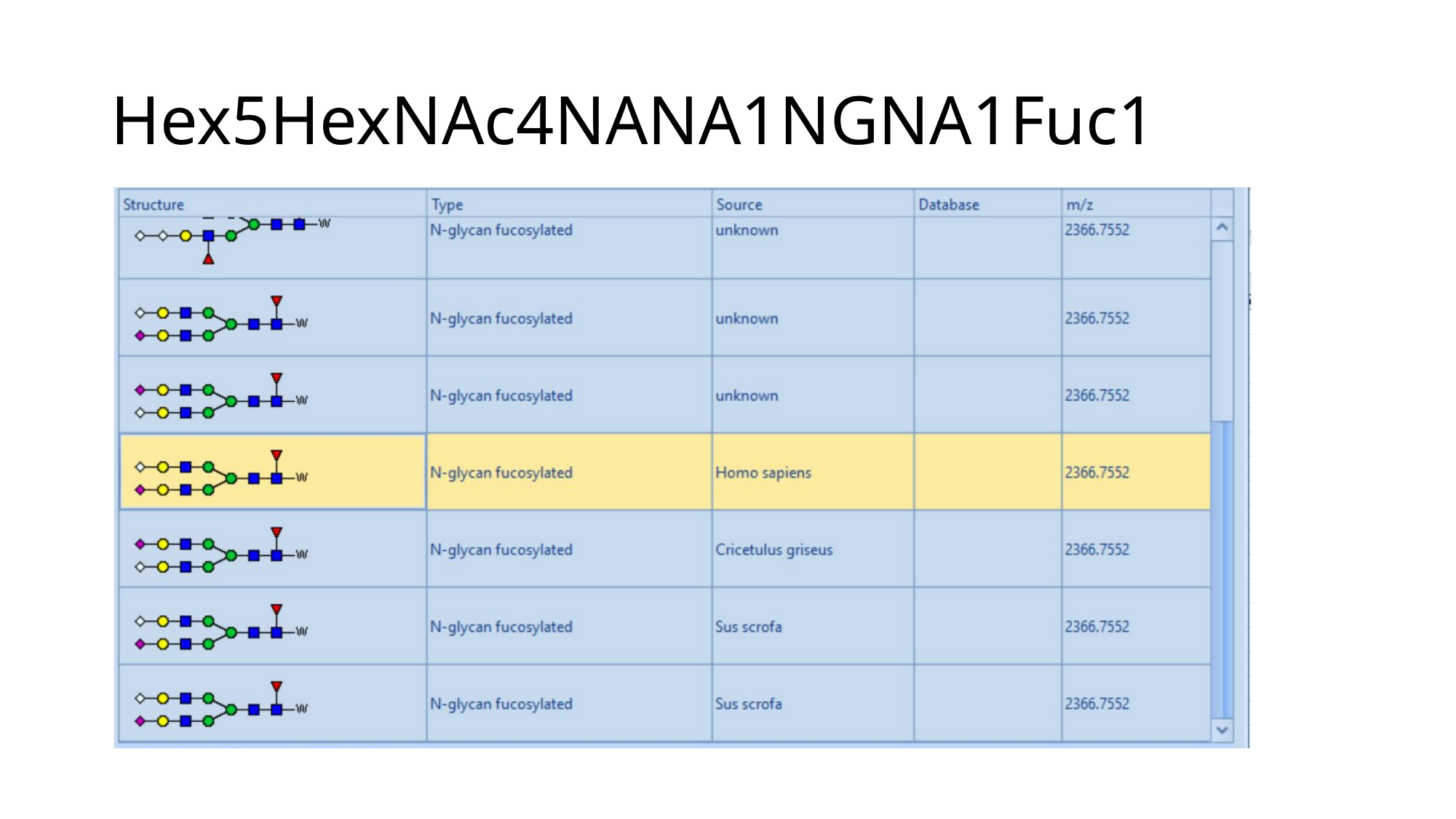

# Hex5HexNAc4NANA1NGNA1Fuc1

## Slide 30
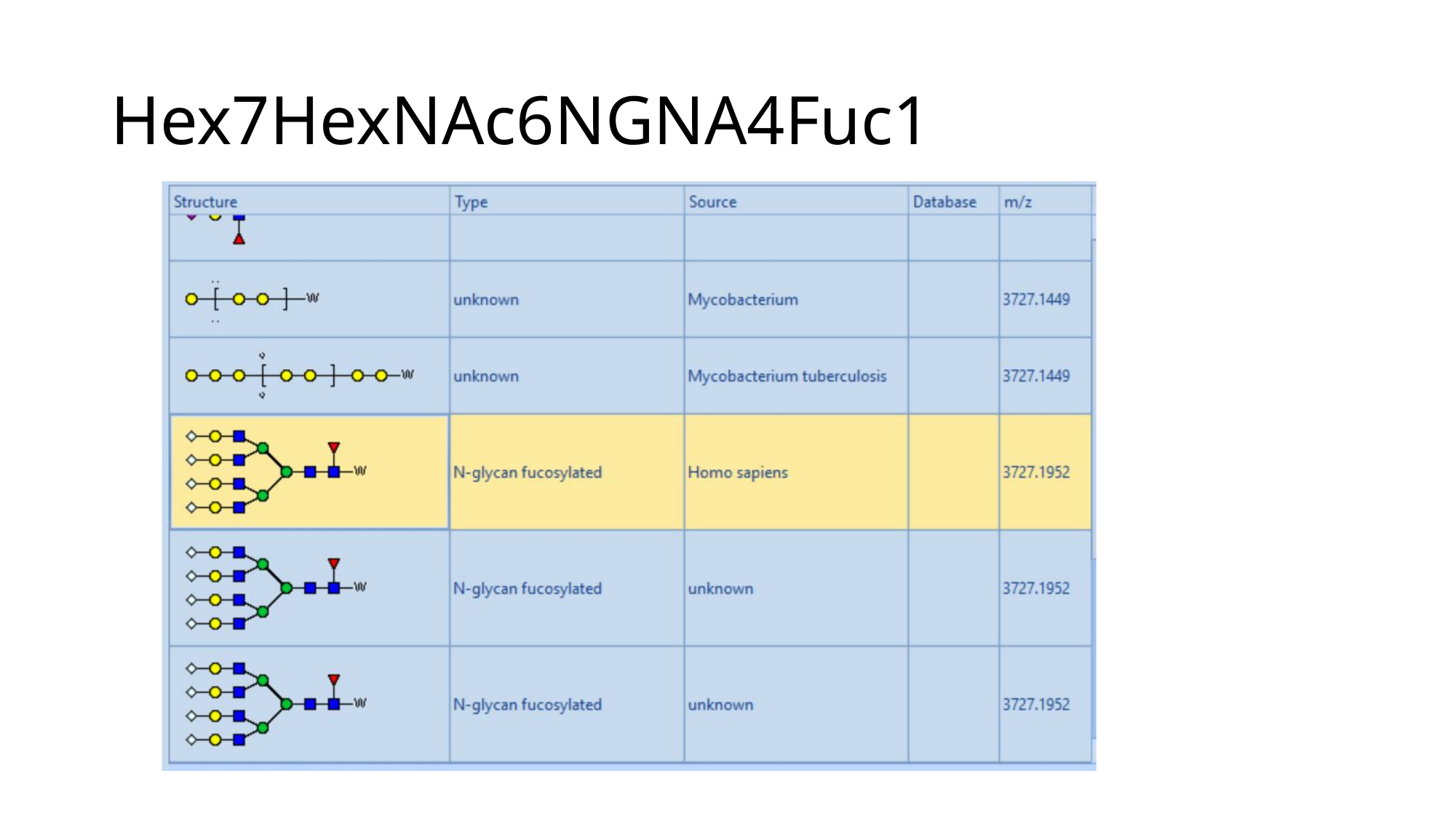

# Hex7HexNAc6NGNA4Fuc1

## Slide 31
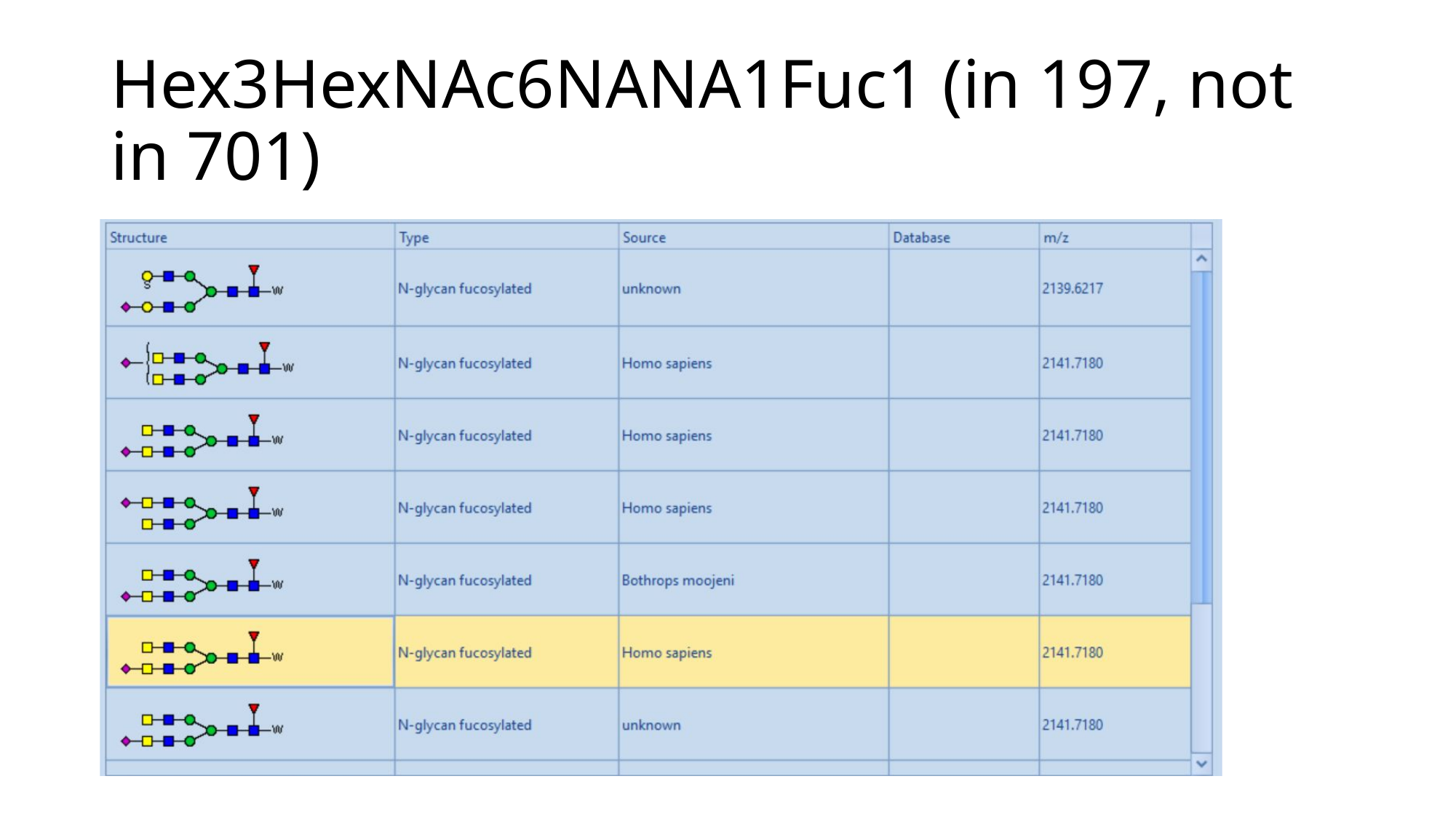

# Hex3HexNAc6NANA1Fuc1 (in 197, not in 701)

## Slide 32
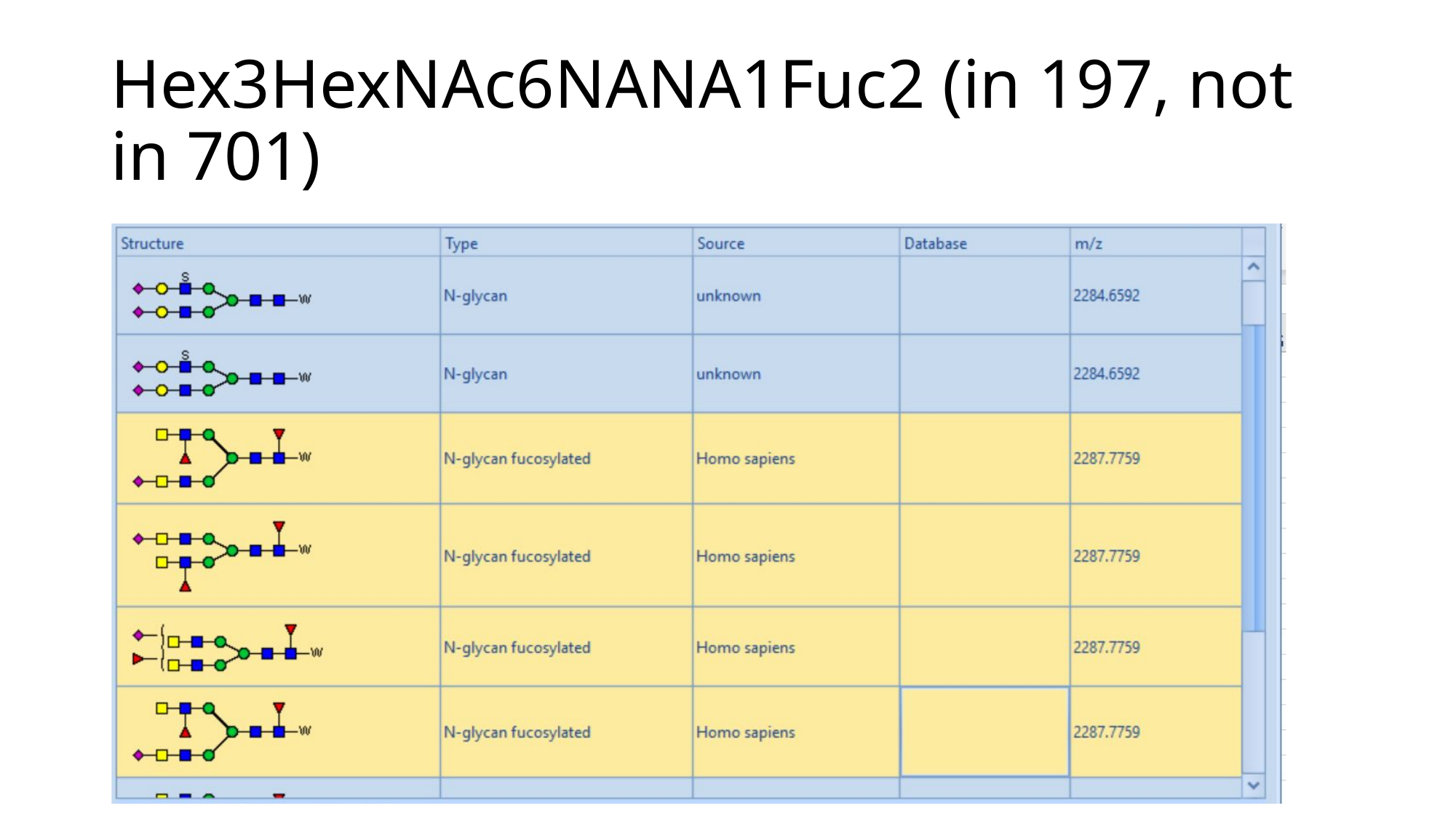

# Hex3HexNAc6NANA1Fuc2 (in 197, not in 701)

## Slide 33
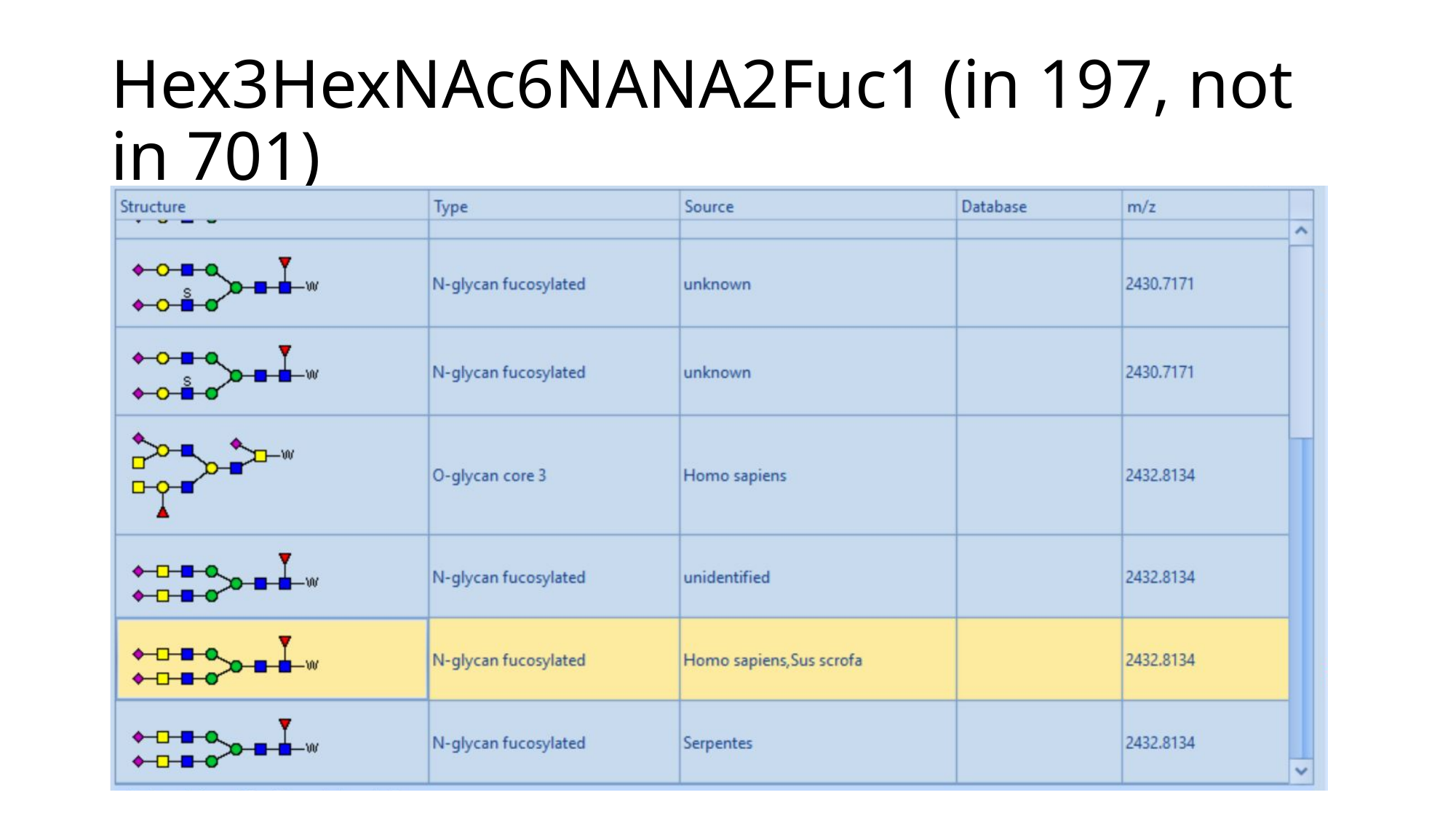

# Hex3HexNAc6NANA2Fuc1 (in 197, not in 701)

## Slide 34
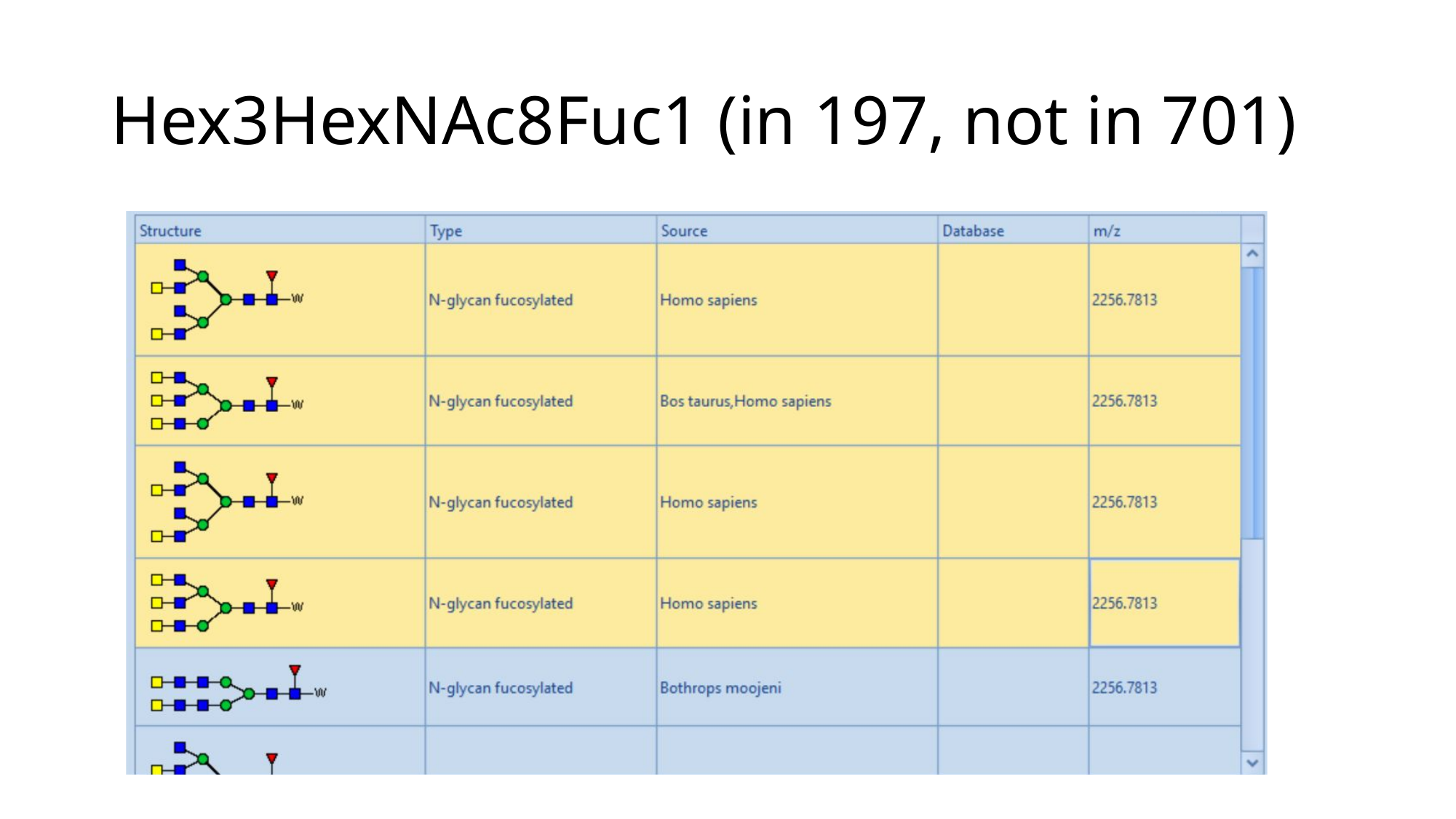

# Hex3HexNAc8Fuc1 (in 197, not in 701)

## Slide 35
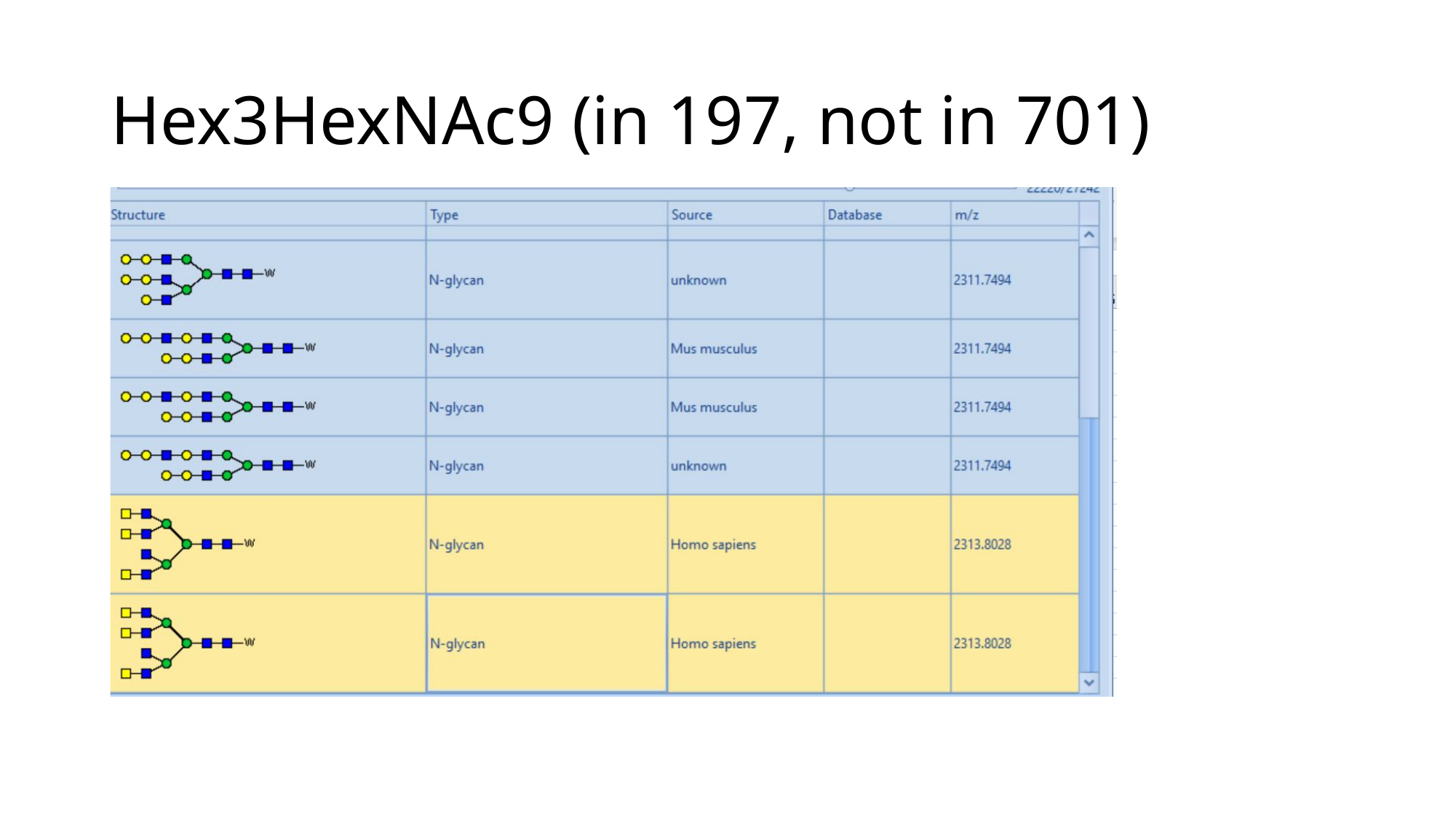

# Hex3HexNAc9 (in 197, not in 701)

## Slide 36
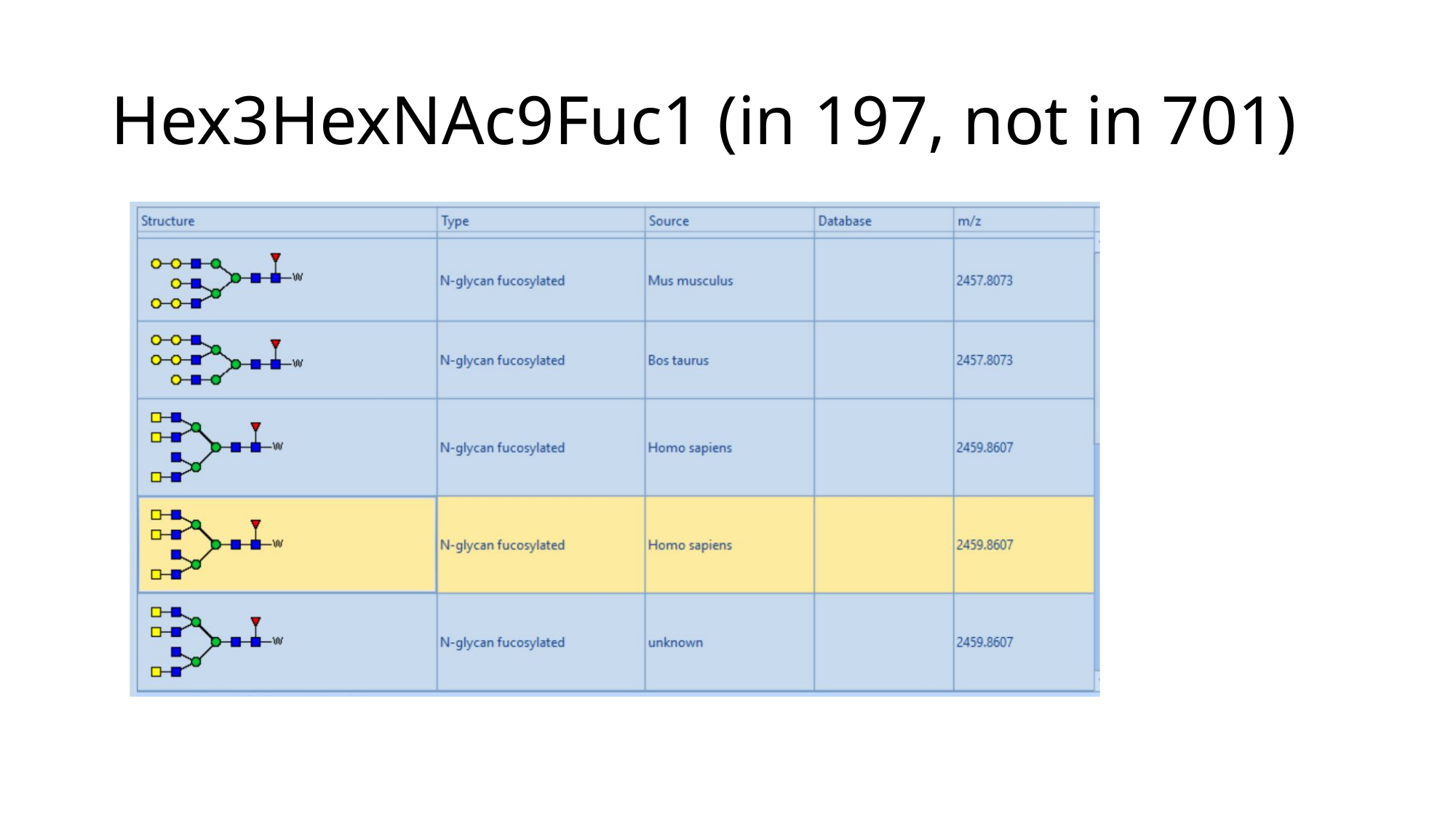

# Hex3HexNAc9Fuc1 (in 197, not in 701)

## Slide 37
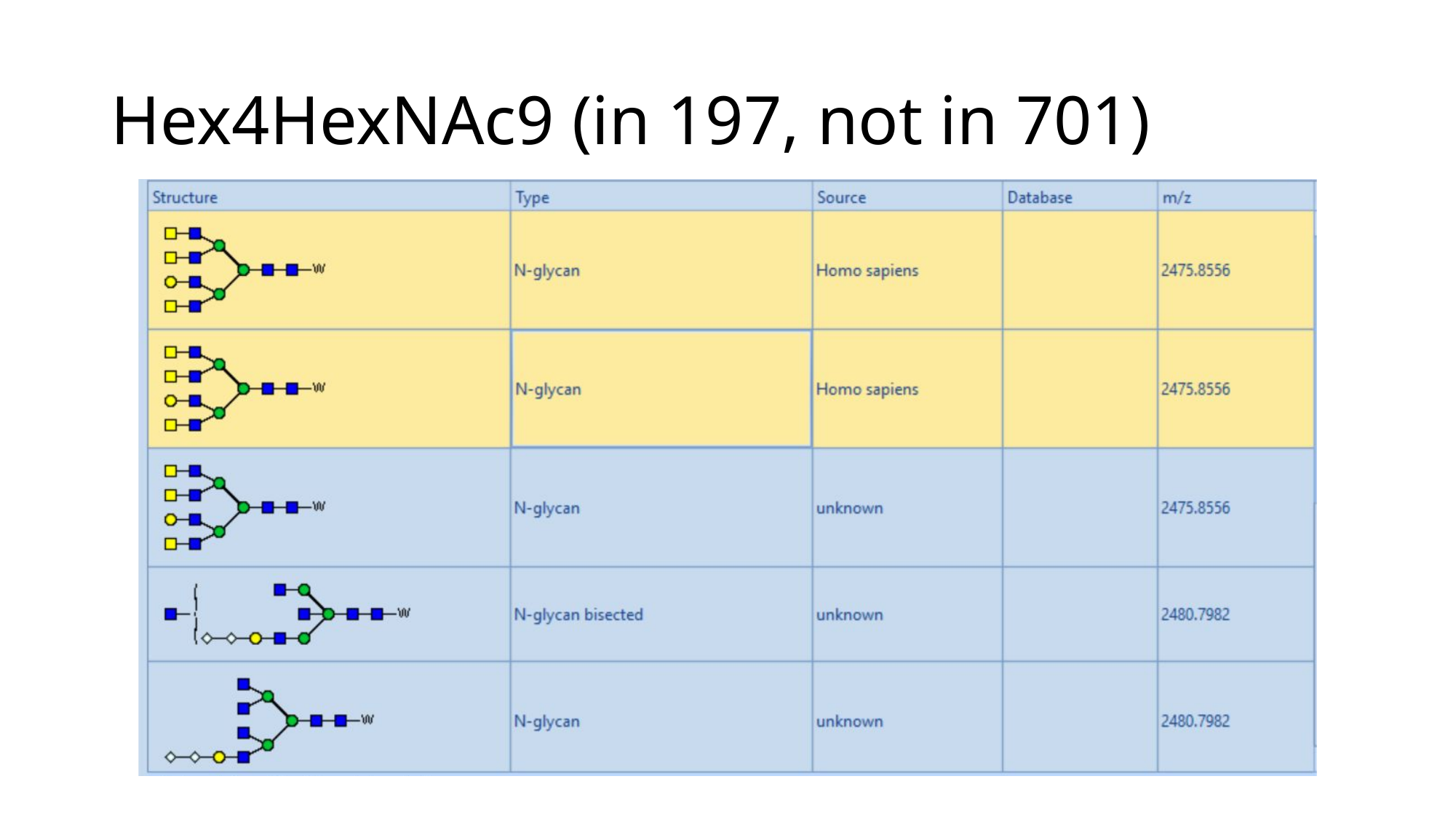

# Hex4HexNAc9 (in 197, not in 701)

## Slide 38
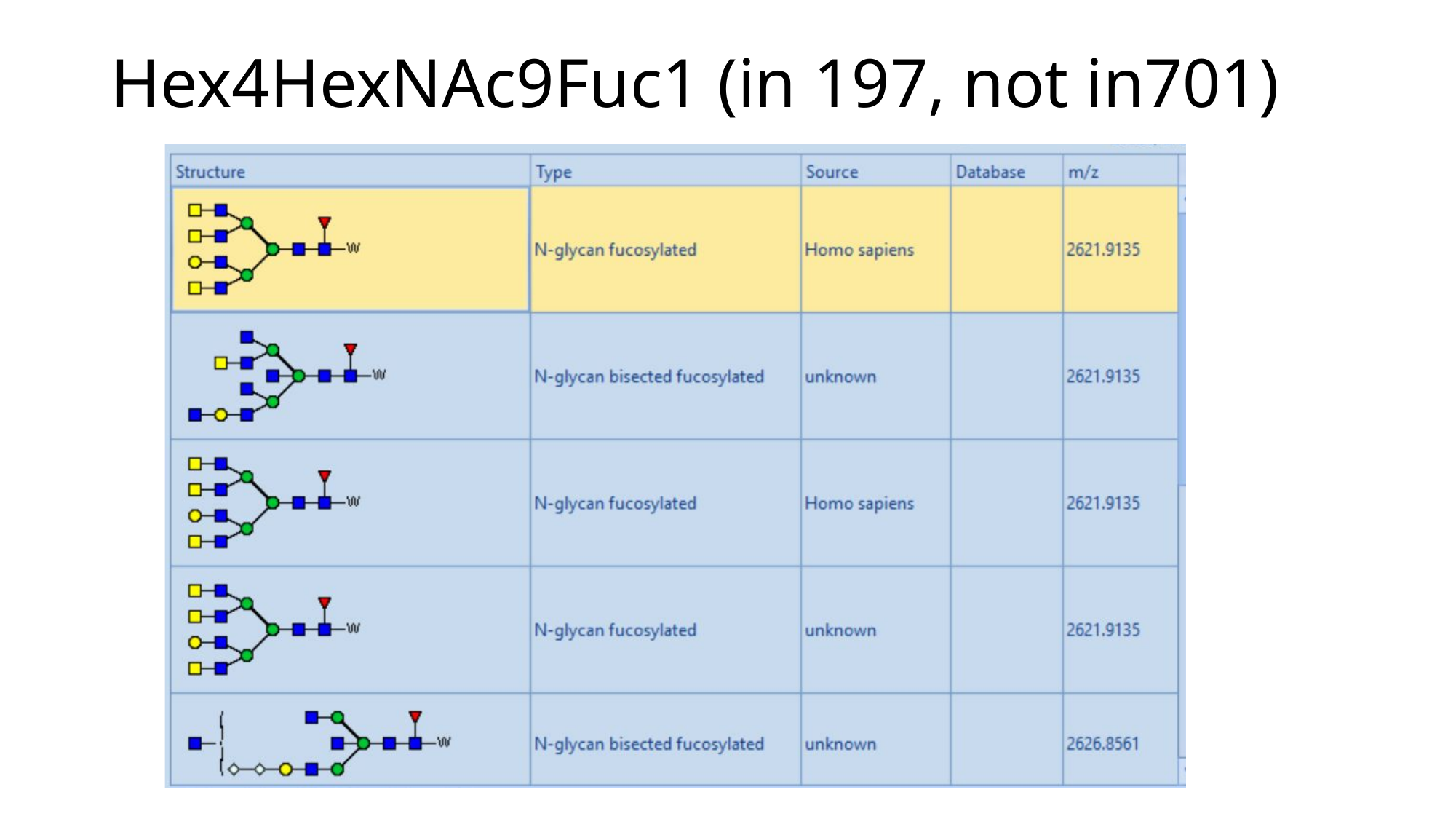

# Hex4HexNAc9Fuc1 (in 197, not in701)

## Slide 39
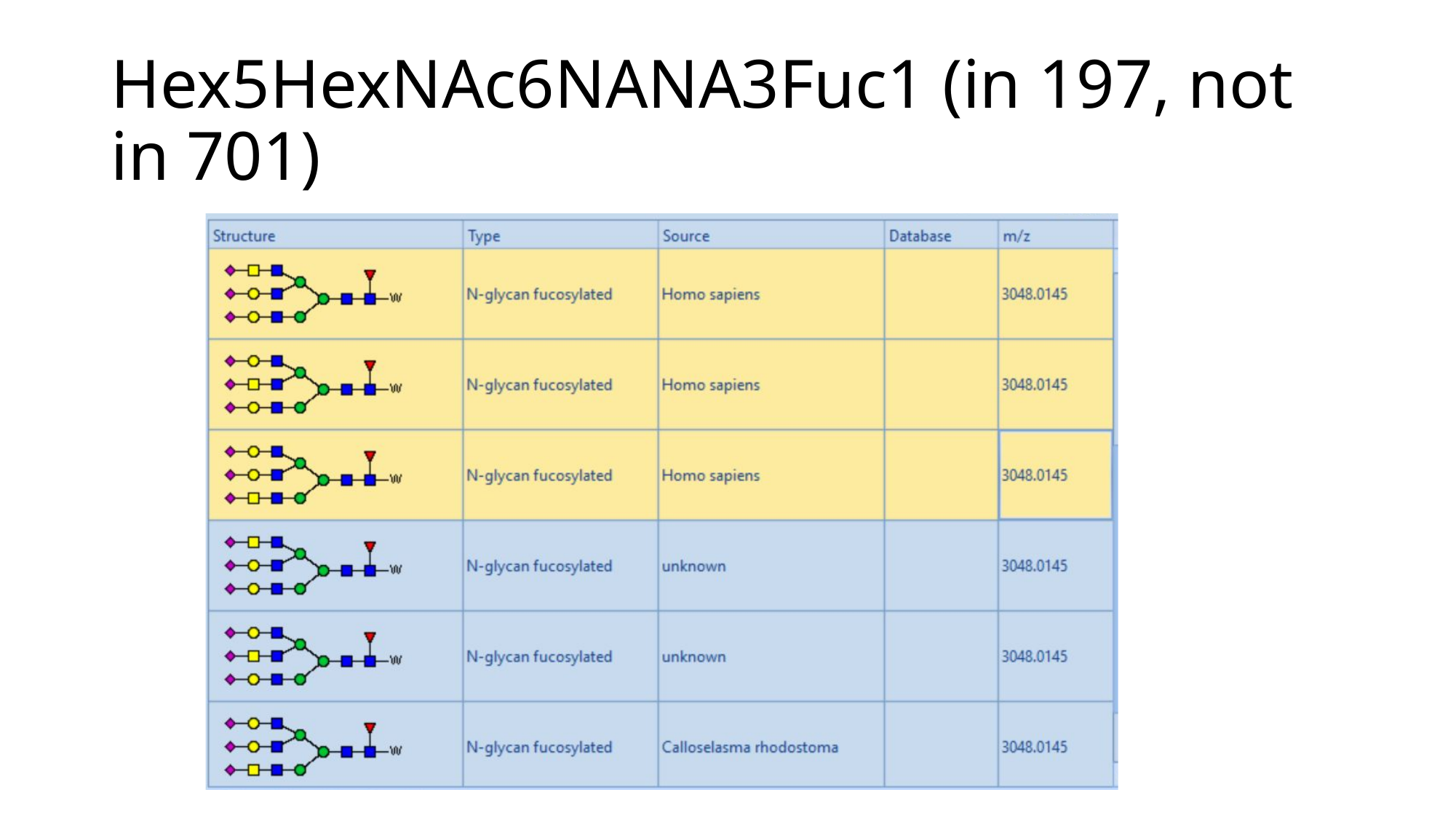

# Hex5HexNAc6NANA3Fuc1 (in 197, not in 701)

## Slide 40
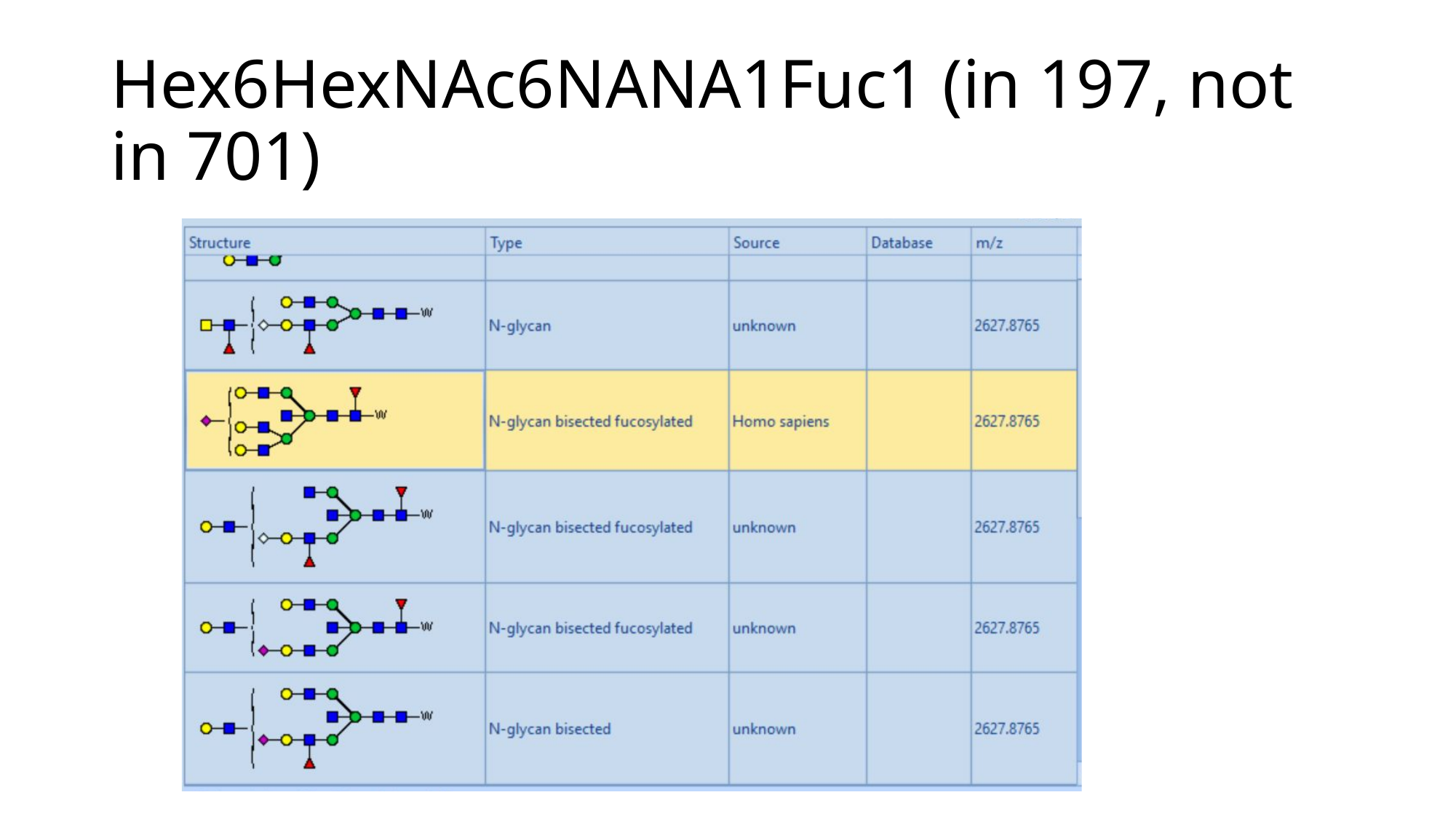

# Hex6HexNAc6NANA1Fuc1 (in 197, not in 701)

## Slide 41
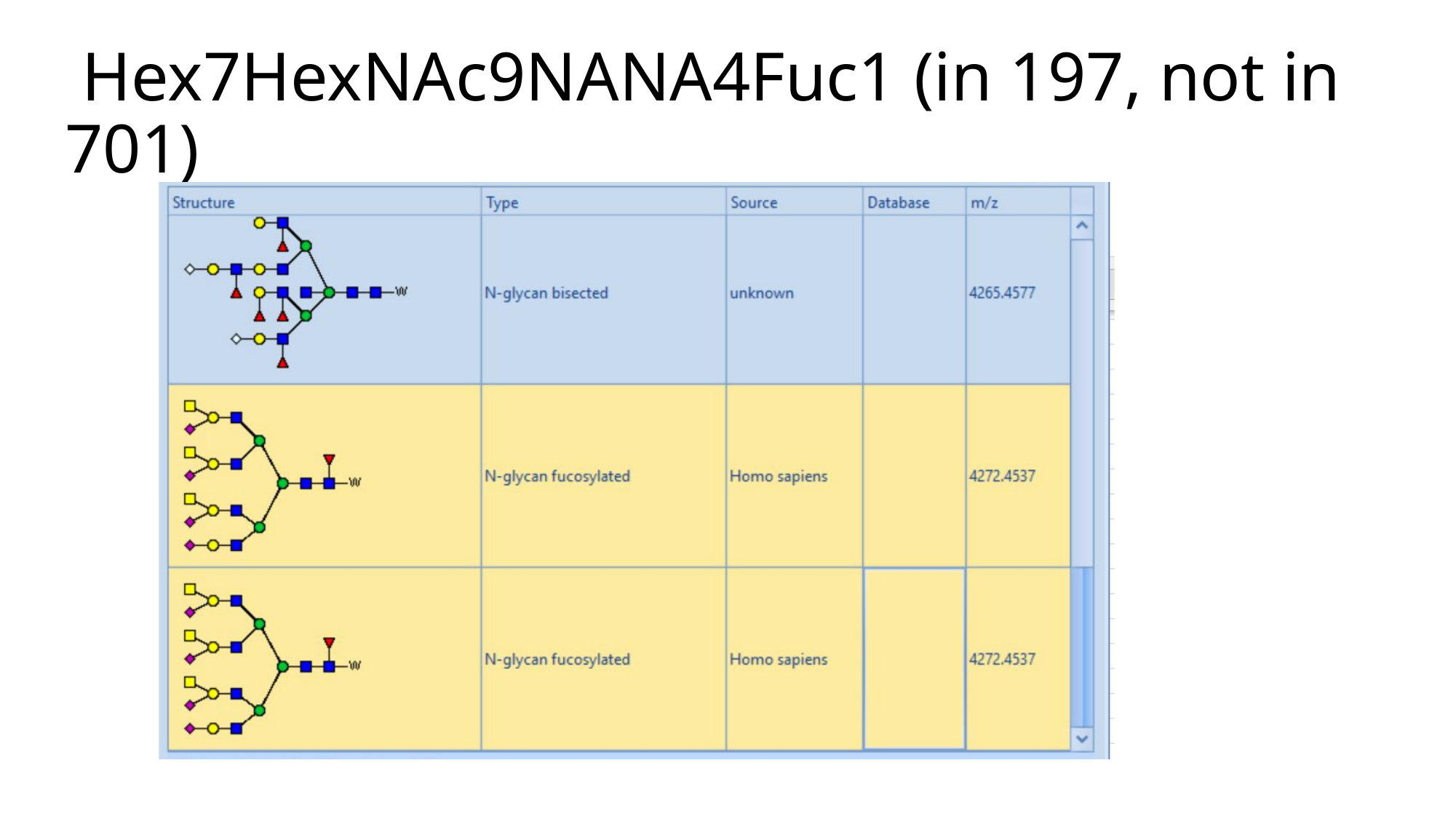

# Hex7HexNAc9NANA4Fuc1 (in 197, not in 701)

## Slide 42
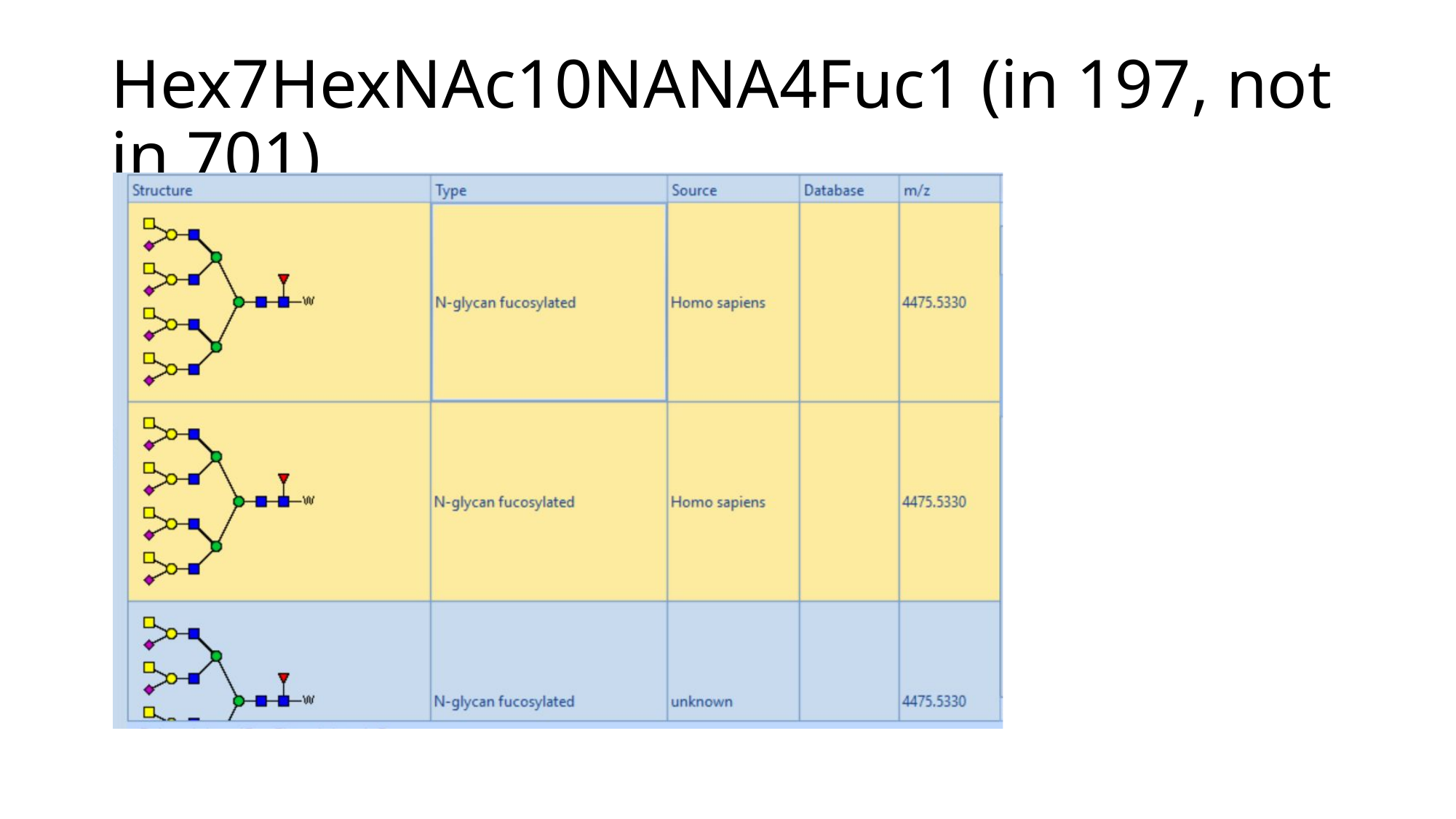

# Hex7HexNAc10NANA4Fuc1 (in 197, not in 701)
